# Arachnid Fauna (Araneae and Opiliones) from the Castro Verde Special Protection Area, southern Portugal

**DOI:** 10.3897/BDJ.11.e110415

**Published:** 2023-12-06

**Authors:** José A. Barrientos, Carlos E. Prieto, Sílvia Pina, Sérgio S Henriques, Pedro Sousa, Stefan Schindler, Luís Reino, Pedro Beja, Joana Santana

**Affiliations:** 1 c/ Balmes, 181, 3º, 2ª. 08006, Barcelona, Spain c/ Balmes, 181, 3º, 2ª. 08006 Barcelona Spain; 2 Departamento de Zoología y Biología Celular Animal, Facultad de Ciencia y Tecnología, Universidad del País Vasco (UPV/EHU). Apdo. 644, 48080, Bilbao, Spain Departamento de Zoología y Biología Celular Animal, Facultad de Ciencia y Tecnología, Universidad del País Vasco (UPV/EHU). Apdo. 644, 48080 Bilbao Spain; 3 CIBIO, Centro de Investigação em Biodiversidade e Recursos Genéticos, InBIO Laboratório Associado, Campus de Vairão, Universidade do Porto, 4485-661, Vairão, Portugal CIBIO, Centro de Investigação em Biodiversidade e Recursos Genéticos, InBIO Laboratório Associado, Campus de Vairão, Universidade do Porto, 4485-661 Vairão Portugal; 4 CIBIO, Centro de Investigação em Biodiversidade e Recursos Genéticos, InBIO Laboratório Associado, Instituto Superior de Agronomia, Universidade de Lisboa, Tapada da Ajuda, 1349-017, Lisboa, Portugal CIBIO, Centro de Investigação em Biodiversidade e Recursos Genéticos, InBIO Laboratório Associado, Instituto Superior de Agronomia, Universidade de Lisboa, Tapada da Ajuda, 1349-017 Lisboa Portugal; 5 Global Center for Species Survival, Indianapolis Zoo, Indianapolis, Indiana, United States of America Global Center for Species Survival, Indianapolis Zoo Indianapolis, Indiana United States of America; 6 International Union for Conservation of Nature (IUCN), Species Survival Commission (SSC), Spider and Scorpion Specialist Group, Gland, Switzerland International Union for Conservation of Nature (IUCN), Species Survival Commission (SSC), Spider and Scorpion Specialist Group Gland Switzerland; 7 Community Ecology and Conservation, Faculty of Environmental Sciences, Community Ecology and Conservation Research Group, Kamýcká 129, CZ-165 00, Prague, Czech Republic Community Ecology and Conservation, Faculty of Environmental Sciences, Community Ecology and Conservation Research Group, Kamýcká 129, CZ-165 00 Prague Czech Republic; 8 BIOPOLIS Program in Genomics, Biodiversity and Land Planning, CIBIO, Campus de Vairão, 4485-661, Vairão, Portugal BIOPOLIS Program in Genomics, Biodiversity and Land Planning, CIBIO, Campus de Vairão, 4485-661 Vairão Portugal

**Keywords:** Alentejo, biodiversity, faunistic, grassland, open farmland, open habitats, Arachnida, taxonomy

## Abstract

**Background:**

With the increasing recognition of the significance of arachnid conservation, it is crucial to allocate greater efforts towards implementing targeted monitoring programmes. Despite recent studies, our understanding of arachnid populations in Portugal remains limited. This study serves as the initial inventory of arachnids (Araneae and Opiliones) within the Castro Verde Special Protection Area (SPA) located in Beja, southern Portugal. The surveys were conducted during the spring of 2012 across 80 open grasslands that were grazed by cattle and sheep.

**New information:**

A total of 71 species of Araneae and two species of Opiliones have been identified. Notably, three spider species, namely *Argennasubnigra*, *Civizelotesibericus* and *Walckenaeriacucullata*, are documented for the first time in Portugal. Additionally, two harvestmen species (*Dasylobusibericus* and *Homalenotusbuchneri*) and 14 spider species (*Cheiracanthiumpennatum*, *Haplodrassusrhodanicus*, *Marinarozelotesminutus*, *Tapinocybaalgirica*, *Agraecinalineata*, *Tibellusmacellus*, *Talaverapetrensis*, *Tetragnathaintermedia*, *Dipoenaumbratilis*, *Enoplognathadiversa*, *Neottiurauncinata*, *Ruborridionmusivum*, *Theridionpinastri* and *Xysticusgrallator*) are recorded for the first time in the Beja District. The occurrence of each documented species within the SPA, including family and species details, is presented, underscoring the significance of the Castro Verde SPA for arachnid conservation. These findings contribute novel insights into the biodiversity of the Castro Verde SPA, emphasising the necessity of incorporating this area into arachnid diversity conservation efforts.

## Introduction

The increasing concern for biodiversity conservation has prompted a rethinking of agricultural management and conservation planning. However, to construct a comprehensive understanding of the impact of agricultural policies, it is essential to study all components of the ecosystem's biodiversity. Several studies (Losey and Vaughan 2006, Schmitz et al. 2017, Schowalter 2017, McCary and Schmitz 2021) emphasise the need to consider the role of invertebrates, given their global dominance in terms of species richness, abundance, biomass and their crucial contributions to ecosystem functioning. However, regional and international conservation planning often neglects invertebrates, with only a few emblematic groups receiving attention. This neglect is particularly evident for small species with limited dispersal abilities and narrow distribution ranges, which constitute the majority of Earth's biota and include many local endemics. Arachnids are particularly endangered by the drastic increase in environmental disturbances worldwide, including pesticide use, deforestation, soil and air pollution, fires and livestock grazing (Cardoso et al. 2020). However, due to the lack of ecological knowledge for the vast majority of species, it is extremely challenging to accurately report on their conservation status (Pinto-da-Rocha et al. 2007). Therefore, it is important to recognise the importance of these overlooked invertebrates and include them in conservation strategies (Pinto-da-Rocha et al. 2007, Cardoso et al. 2011b).

Spiders (Arachnida, Araneae), with over 51,000 described species (World Spider Catalog 2023), play a crucial role in ecosystems as both important food sources for higher trophic levels (e.g. reptiles, birds, mammals) and predators in terrestrial ecosystems. They provide valuable ecosystem services by controlling arthropod pests in agroecosystems, benefitting humans (King and Hardy 2013, Nyffeler and Birkhofer 2017, Michalko et al. 2019). Despite their ecological significance and diversity, spiders are often overlooked in conservation policies compared to other groups (Milano et al. 2021). While Europe is home to 4,154 spider species, only a small fraction has been assessed for extinction risk by the IUCN Red List of Threatened species (IUCN 2021), in contrast to the comprehensive assessments conducted for 97% of European butterflies (van Swaay et al. 2010) and dragonflies (Kalkman et al. 2010). The EU Habitats Directive includes only one out of the 4,154 spider species, while 50 butterfly species out of 496 and 16 dragonfly species out of 143 are listed, highlighting a notable taxonomic bias even within invertebrates (Cardoso 2011,Cardoso et al. 2011b, Milano et al. 2021). Currently, only eight European spider species are listed on the IUCN Red List and the legislation of 19 European countries formally mentions 178 species (Milano et al. 2021). This taxonomic bias in biodiversity conservation is a result of data deficiencies, biased policy, research priorities and lack of public support (Cardoso et al. 2011a). Such inequities have significant implications for scientific research and conservation priorities, stressing the urgent need for more comprehensive assessments of the conservation status of spider species. On the other hand, while harvestmen (Arachnida, Opiliones) exhibit lower diversity compared to other spiders, representing approximately 13% of spider diversity, there are still about 6,673 extant described species (Kury et al. 2023). They are generalist predators although they do not reject scavenging, fungi or plant matter (Pinto-da-Rocha et al. 2007). Currently, no European harvestmen are listed on the IUCN Red List and only 22 species are listed worldwide.

The Mediterranean Basin harbours the highest spider diversity in Europe, yet conservation efforts in this region are insufficient, both in terms of assessments and the establishment of national or regional legislation (Milano et al. 2021). In Spain, for example, with its rich araneofauna comprising 1,386 spider species, only 10 species are listed in the National Red Book (Verdú et al. 2011). In mainland Portugal, there are 829 known spider species (Branco et al. 2019, de Biurrun et al. 2019) and only one species, *Macrothelecalpeiana* (Walckenaer, 1805), is included in the Annex IV of the EU Habitats Directive. In respect to harvestmen, the Iberian harvestmen fauna includes 139 species; currently, only one species (*Maiorerusrandoi* Rambla, 1983 from Canary Islands) is included in the “Catálogo Español de Especies Amenazadas” (Verdú et al. 2011; updated by the Ministerio para la Transición Ecológica y el Reto Demográfico (2023)). In addition, only about 37 species (plus *Ramblinusspinipalpis* (Roewer, 1911) from Madeira Island) are known from Portugal, representing less than 1/20 of the Portuguese spider diversity. These figures highlight the need for further research and to implement measures to protect the diverse arachnid populations in the Mediterranean Basin.

In this study, we present the first systematic inventory of the Arachnid fauna in the Castro Verde Special Protection Area (SPA). The SPA is a Mediterranean High Natural Value open farmland primarily designated for the conservation of steppe birds under the European Union (EU) Birds Directive (79/409/EEC) (Santana et al. 2013). While the biological and conservation importance of birds in the Castro Verde SPA has been well recognised, there is limited knowledge about its value for other groups, particularly arthropods (but see Pina et al. (2017) and Vasconcelos et al. (2019)). Prior to this study, information on arachnids in this area was limited to sporadic captures (Silva 2004). However, in previous works carried out in the Beja District (Baixo Alentejo), we find occasional information on certain species and their distribution in the region. These works, include Bacelar (1933), Bacelar (1935), Bacelar (1940), Machado (1941), Senglet (1972), Ferrández (1986), Ferrández (1991), Ferrández (1996), Méndez (2003), Pekár et al. (2003), Silva (2004), Pekár and Cardoso (2005), Decae et al. (2007), Bosmans et al. (2010), Carvalho et al. (2011), Pekár et al. (2011), Bolzern et al. (2013), Krehenwinkel and Tautz (2013), Lissner and Bosmans (2016). Undoubtedly, the most relevant insights are derived from the studies conducted in the Guadiana River Natural Park (Cardoso 2004,Cardoso et al. 2009) and the subsequent research on the olive groves surrounding Beja (Melic et al. 2016, Barrientos et al. 2020). Therefore, the data we provide here are of the utmost importance for evaluating the overall biodiversity of the Castro Verde SPA. We specifically focus on Arachnida to contribute to the expansion of our understanding on the fauna in Portugal. We present a comprehensive species list along with information on their respective habitats within this important open farmland landscape. Additionally, we compile data on the distribution of each species and provide relevant comments on the significance of the Castro Verde SPA for the conservation of Iberian Arachnida.

## Materials and methods

### Study area

**Figure 1.**
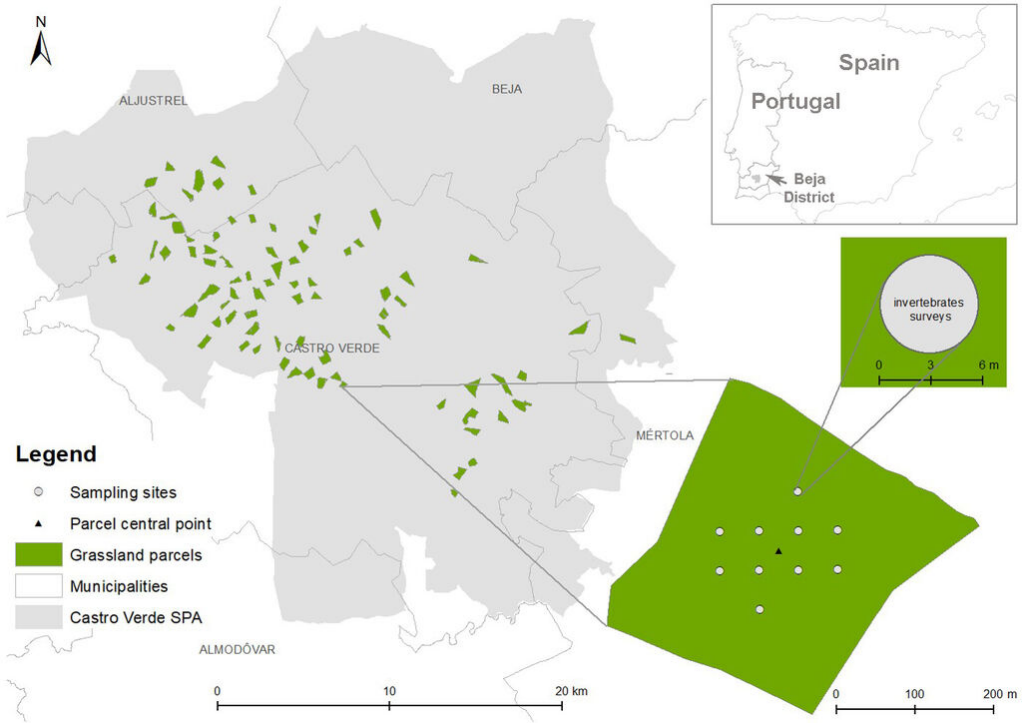
Location of the study area in southern Portugal, highlighting the Castro Verde Special Protection Area (SPA), the municipalities within the Beja District and the 80 grassland parcels. Illustration of the sampling scheme that comprised 10/12 sampling plots (10 in the example) positioned at 50-m intervals around the central point of each parcel. Each sampling plot consisted of a 3-m radius circle, in which invertebrates were collected using a Vortis suction sampler (10 sub-samples of 15 s duration).

The study was conducted in the open farmlands included in the Special Protection Area (SPA) of Castro Verde, southern Portugal (37^o^44'N, 8^o^00'W, Fig. 1). The climate is Mediterranean, with hot summers (30-35ºC on average in July), cold winters (5ºC in January) and with more than 75% of annual rainfall (500-600 mm) located between October and March (Moreira et al. 2005) and the landscape is gently undulated (100-300 m a.s.l.). The Castro Verde SPA was created in 1999 and subsequently enlarged in 2008 to its current size (85,345 ha) and is the most representative grassland landscape in Portugal. The SPA was specially designated for the conservation of steppe birds and their habitats (mainly *Otistarda*, *Tetraxtetrax* and *Falconaumanni*). Most of the area has benefitted from agri-environmental schemes since 1995. The area was traditionally dominated by the rotation of rain-fed grain cereals and fallows typically grazed by sheep at low stocking densities (Ribeiro et al. 2014,Ribeiro et al. 2015), which provide habitat for grassland bird species of conservation concern (Delgado and Moreira 2000, Santana et al. 2013). However, by 2010, this system had shifted to specialised production of either cattle or sheep grazing (Ribeiro et al. 2014, Ribeiro et al. 2015), with declines in cereal and fallow land and increases in forage crops and permanent pastures (Ribeiro et al. 2014, Ribeiro et al. 2015, Santana et al. 2017). Due to historical land-use patterns and legal restrictions associated with the SPA, tree cover is very scarce, with eucalyptus (*Eucalyptus* sp.), oak (*Quercusrotundifolia* and *Q.suber*) and umbrella pine (*Pinuspinea*) plantations located mainly in the north and south of the study area (Moreira et al. 2007, Reino et al. 2009). Pine and oak plantations often have a grassy undergrowth grazed by livestock (Vasconcelos et al. 2019). Shrublands occur mainly in association with rivers and in the south-eastern part of the study area (Moreira et al. 2007, Reino et al. 2009).

### Sampling design

Sampling was conducted in 80 open grassland parcels (Table 1) selected, based on a network of sampling sites established in 2012 to study the effects of grazing regimes on farmland birds (Ramos et al. 2021, Fig. 1). Specifically, parcels were selected following a stratified random procedure with some restrictions. We started by identifying fallows and permanent pastures grazed by either sheep or cattle, based on parcel-level statistical information from 2010 provided by the Portuguese Ministry of Agriculture (Ribeiro et al. 2014). To minimise potentially confounding effects of adjacent land uses and other non-crop elements within parcels, we excluded parcels less than 100 m from shrubland or forested areas, with shrub and tree cover > 5% and with a size <10 ha. Then, we randomly selected 120 parcels and performed fieldwork to confirm land uses and select a balanced proportion of 80 fallow and permanent pastures, grazed by sheep and cattle.

### Spiders and harvestmen surveys

Sampling was conducted during 4 April - 9 May 2012 by JS and SH when the grass was dry (Eschen et al. 2012). The sampling scheme comprised 10/12 sampling plots positioned at 50-m intervals around the central point of each parcel (Fig. 1). Each sampling plot consisted in a 3-m radius circle, in which we collected 10 sub-samples of 15 s duration (total area per sample = 1.94 m^2^) using a Vortis suction sampler (Burkard Manufac-turing Co., Ltd., UK) (Fig. 1). Sub-samples were stored in vials with 70% ethanol. Vegetation height was also estimated for each plot by taking ten measurements at random locations within the plot using a 50 cm ruler and recording the highest point of vegetation projection within 3 cm of the ruler to the nearest half-centimetre (Ramos et al. 2021).

### Sample’s study and identification

Each sample was cleaned at the CIBIO/InBIO laboratory in Lisbon. SP and JS conducted the initial sorting of the samples into major taxonomic groups using binocular microscopes and using relevant general bibliography. Therefore, arachnids constitute only one of several batches of invertebrate fauna collected for each sample. To facilitate the separation of invertebrates from soil debris and vegetation in the samples, a mixture of magnesium sulphate (MgSO_4_) was added to the alcohol, causing the invertebrates to float (Zaborski and Cloyd 2004). Sorted invertebrates were preserved in 70% ethanol with 5% glycerine. Each vial was consistently labelled with the original capture data and a project-wide code: parcel-sampling plot-vial (e.g. 55-9-g).

The taxonomic identification of the Araneae was carried out by José A. Barrientos in his personal office, following the general guidelines: observation and manipulation with binocular microscopy and cold illumination and the help from the relevant bibliography (Simon 1914, Simon 1929, Simon 1932, Simon 1937, Locket and Millidge 1951, Wiehle 1956, Locket and Millidge 1953, Wiehle 1960, Locket et al. 1974, Roberts 1985, Roberts 1987, Roberts 1995, Nentwig et al. 2023). Where necessary, we have also relied on other literature; these references are detailed later in the text. From a nomenclatural perspective, we have followed the World Spider Catalog (2023). Juvenile specimens were identified whenever possible. When adult specimens were present in the sample, juveniles that matched their overall morphology unambiguously were assigned to the adult species. When adult specimens were not present, juveniles were identified to species level only if they presented morphological characters or colour patterns that allowed their identification to a specific species known to occur in the Iberian Peninsula.

The taxonomic identification of the Opiliones was carried out by Carlos E. Prieto at the Zoology Laboratory of the Basque Country University, following classical procedures (observation under binocular stereomicroscope, penis removal and temporal mounting for microscopical study) with the help of the relevant bibliography (Rambla 1967, Martens 1978, Prieto and Merino-Sáinz 2020).

Spider samples are deposited at JAB personal collection (Barcelona, Spain) and the harvestmen samples are deposited in the Zoology Coleccion of the Basque Country University (ZUPV). All the specimens reported in the manuscript are available upon request.

In this study, we provide a checklist of the studied material organised by the alphabetical order of the taxa "Checklist of the Arachnid Fauna (Araneae and Opiliones) from the Castro Verde Special Protection Area, Southern Portugal", which contains some comments on the different species recorded. The current distribution of each species in general and in the Iberian Peninsula is indicated together with a comment when our findings point to an extension of the known distribution range of the species. The number of males (♂♂), females (♀♀) and juveniles (jj) studied is indicated for each species, genus (doubtful species assignment, indet.) or family (doubtful genus assignment, indet.). The data collected for the present study were included in the dataset Arachnid fauna (Araneae and Opiliones) from the Castro Verde Special Protection Area, southern Portugal, which is available through GBIF (Barrientos et al. 2023, Suppl. materials 1, 2). We provide details of all the samples analysed, indicating the sampling plots where they were collected, the date of collection, the number of specimens (♂♂, ♀♀ and jj) and the municipality (and farm) to which they correspond. Then, we provide a table (Table 2) which compiles all previously mentioned arachnid records for the broader Beja District (Aranae: de Biurrun et al. (2019), Barrientos et al. (2020); Opiliones: Rambla (1967), Rambla (1970)) and the species colected in the Castro Verde SPA. The information given facilitates the comparison with the newly-generated data provided for the District.

## Data resources

The data underpinning the analysis reported in this paper are deposited at GBIF, the Global Biodiversity Information Facility, https://doi.org/10.15468/x8jjwh.

## Checklists

### Checklist of the Arachnid Fauna (Araneae and Opiliones) from the Castro Verde Special Protection Area, southern Portugal

#### Araneae

Clerck, 1757

E820D0C4-9E35-5F66-A083-114095649454

#### Agelenidae

C. L. Koch, 1837

3C9DE9AF-C7D3-5923-B579-FE57B7055B0D

#### Eratigena

Bolzern, Burckhardt & Hänggi, 2013

A2F70463-9C33-5841-A5A8-14F924EF891D

#### Eratigena sp.

EE9508CA-AC7A-5FE7-82D4-336B580A7C6B

##### Notes

1 j. indet.

#### Anyphaenidae

Bertkau,1878

89A022EB-07A3-518D-A435-0DB65434013C

#### Anyphaena

Sundevall, 1833

42970722-C032-53B6-95A9-CF89CCAB618F

#### Anyphaena sp.

057E48BE-D2A1-5935-A07A-D070346AA719

##### Distribution

There are no previous records from the SPA of Castro Verde, nor from the District of Beja; this is the first record of the family Anyphaenidae for this Portuguese district.

##### Notes

2 jj. indet.

#### Araneidae

Clerck, 1757

88546454-AA23-5B1A-BE44-3016E34C7E10

#### Araneidae sp.

452C3C48-0FC1-5144-BD26-92AD20B7B98C

##### Notes

484 jj. indet.

#### Araniella

Chamberlin & Ivie, 1942

43BEA78D-274D-5F74-AD4B-75116B58760E

#### Araniella sp.

FB816A2A-4CDC-57D0-9F7A-DC2F68C69B87

##### Distribution

Probably *Araniellacucurbitina* (Clerck, 1757). The genus has been previously recorded for the District of Beja (Barrientos et al. 2020).

##### Notes

9 jj. indet.

#### Hypsosinga

Ausserer, 1871

A2FCD350-A92C-57EF-9C04-AE1EAB89D6B1

#### Hypsosinga albovittata

(Westring, 1851)

3B207219-EFA1-5EF1-A62B-5CEAFD995A1E

https://wsc.nmbe.ch/lsid/urn:lsid:nmbe.ch:spidersp:016183

##### Distribution

Species widespread throughout the Palaearctic Zone. In the Iberian Peninsula, it has been reported for very scattered localities (also in Portugal), so it can be considered a common species. It has been previously cited from the District of Beja (Cardoso et al. 2009). The literature tends to link it to sunny places in areas of low grass and close to the ground (Nentwig et al. 2023). These characteristics coincide with those found in the study area, where it is one of the most abundant species.

##### Notes

5♂♂, 32♀♀, 373 jj.

#### Mangora

O. Pickard-Cambridge, 1889

54453891-C07A-5F44-80DC-D614ED19D1BD

#### Mangora acalypha

(Walckenaer, 1802)

30AE938A-956A-5400-BABB-586C6AF453CA

https://wsc.nmbe.ch/lsid/urn:lsid:nmbe.ch:spidersp:016308

##### Distribution

It is also a very common species throughout the Palaearctic Zone. In the Iberian Peninsula, it has been cited on numerous occasions and from numerous localities that attest to its widespread occurrence (de Biurrun et al. 2019). It has been previously reported for the District of Beja (Barrientos et al. 2020). Like *H.albovittata*, it is common in herbaceous and shrubby environments, building its webs on the vegetation; however, captures in Castro Verde are scarce.

##### Notes

1♂, 1♀

#### Cheiracanthiidae

Wagner,1887

4EDAD934-2544-5E46-8D97-6E3ADF3D6FA1

#### Cheiracanthium

C. L. Koch, 1839

F2C9FF7F-9571-5419-9719-E72A62665744

#### Cheiracanthium pennatum

Simon, 1878

0DCCD1F7-894F-5EDD-9F05-6B920BD7FFDC

https://wsc.nmbe.ch/lsid/urn:lsid:nmbe.ch:spidersp:023399

##### Distribution

Available data place this species in several southern European countries. However, in the Iberian Peninsula, it is only known from three localities: two in Spain and one in Portugal (Santarém; Cardoso (2004)). Now, we report it for the first time in the District of Beja. Like other species of the genus, it usually occupies the upper parts of herbaceous plants, where it builds its typical silk retreats (Urones 1988).

##### Notes

7♂♂, 6♀♀, 9 jj.

#### Clubionidae

Simon, 1878

49B0E98E-DDC3-53DB-886A-C8645EB4F349

#### Porrhoclubiona

Lohmander, 1944

00341FB1-67C8-54E3-B91F-68414B972BDF

#### Porrhoclubiona vegeta

(Simon, 1918)

7390E5BD-00BB-5AE6-ACA9-EBD7DECD823D

https://wsc.nmbe.ch/lsid/urn:lsid:nmbe.ch:spidersp:024816

##### Distribution

Species of Mediterranean distribution (southern Europe and northern Africa), especially frequent in its western part. Recently characterised by Bosmans et al. (2017), who cite it from Beja District.

##### Notes

2♀♀, 14 jj.

#### Dictynidae

O. Pickard-Cambridge, 1871

B526DB34-392D-5D0B-BB11-54A7F1FED5E6

#### Argenna

Simon, 1884

ABF5CAD2-E12B-5465-AE0D-E6251F3F409A

#### Argenna subnigra

(O. Pickard-Cambridge, 1861)

89FC87F6-6D9B-5E6B-960B-072951FF72AB

https://wsc.nmbe.ch/lsid/urn:lsid:nmbe.ch:spidersp:022086

##### Distribution

There are data that place it throughout the Palaearctic area (World Spider Catalog 2023), although most of the information corresponds to European countries. In the Iberian Peninsula, it has only been mentioned from three localities in Spain (Carter 1984, Barrientos 1985, Barrientos et al. 2016). This is the first record for Portugal. It is considered a rare species that lives in sunny areas under stones and plant debris; however, it has been repeatedly captured in the Castro Verde area.

##### Notes

16♀♀, 22 jj.

#### Marilynia

Lehtinen, 1967

817CC1BE-771D-5DCF-B1B0-42845B8424AF

#### Marilynia bicolor

(Simon, 1870)

7F5E5714-28C2-54D6-9093-55F7BAC1217B

https://wsc.nmbe.ch/lsid/urn:lsid:nmbe.ch:spidersp:022497

##### Distribution

Although it is relatively common in the Mediterranean area (Europe and North Africa), it is distributed throughout the Palearctic. It has already been reported for the District of Beja, as well as from other localities in Portugal and Spain (de Biurrun et al. 2019).

##### Notes

1♂, 2♀♀, 5 jj.

It is easily recognisable by its dorsal opisthosomal pigment pattern, but, although it seems to be frequent, it is not particularly abundant.

#### Nigma

Lehtinen, 1967

384B6D32-9B1B-5BCE-ACEC-A40A7D303F3B

#### Nigma puella

(Simon, 1870)

733F23A8-936A-5CDD-BBE5-32A1F3545201

https://wsc.nmbe.ch/lsid/urn:lsid:nmbe.ch:spidersp:022515

##### Distribution

Species typical of the western Palaearctic, frequent in Europe and the Macaronesian area. It has already been reported for several localities in Portugal, including in the District of Beja (Barrientos et al. 2020). It is usually associated with shrubs of a certain size, where it forms abundant populations; herbaceous areas are not a suitable habitat for it (van de Putte 2018).

##### Notes

1♂

#### Dysderidae

C. L. Koch, 1837

5FB69ECE-1160-5C26-A09B-0D39B12461CA

#### Harpactea

Bristowe, 1939

C6A74FD8-5A9E-51D5-AF85-C9F737064A25

#### Harpactea minoccii

Ferrández, 1982

79FC0DA7-42F1-5ABC-9190-0A9980EC82A7

https://wsc.nmbe.ch/lsid/urn:lsid:nmbe.ch:spidersp:004685

##### Distribution

This species can be considered an Iberian endemism. The original description places it in Seville (Ferrández 1982) and it had not been found again until the studies in the Parque Natural do Vale do Guadiana (Cardoso 2004, Cardoso et al. 2009) which provide eight more localities, all of them in the District of Beja. Our finding confirms its presence in this part of Portugal and suggests that *H.minoccii* is distributed in the south-western part of the Iberian Peninsula, although, for the time being, we can consider it a rare species.

##### Notes

1♂

#### Gnaphosidae

Banks,1892

53C6F217-10D6-532E-9639-5DB48111A7F5

#### Gnaphosidae sp.

B710BB98-2AC1-5883-A65A-250F61FE6D9A

##### Notes

51 jj. indet.

#### Aphantaulax

Simon, 1878

7DF0ECBC-7A90-52F0-BC46-EF39EB19A41B

#### Aphantaulax sp.

55567AA1-A82E-51F7-9D08-FB84B6D6B307

##### Distribution

The six specimens captured are assignable to the genus *Aphantaulax* Simon, 1878, but there is no certainty regarding the species. This genus has been previously recorded for the District of Beja (Barrientos et al. 2020).

##### Notes

6 jj. indet.

#### Civizelotes

Senglet, 2012

2ABB318E-5DF7-5FB5-BC89-8170B36C2973

#### Civizelotes civicus

(Simon, 1878)

10FB75AA-7C07-5689-9347-B4E336B27F27

https://wsc.nmbe.ch/lsid/urn:lsid:nmbe.ch:spidersp:028161

##### Distribution

Species native to the western Mediterranean area; it has also been reported for Madeira. In the Iberian Peninsula, it has been mentioned for numerous localities in Spain and Portugal, including in the District of Beja (de Biurrun et al. 2019). It is a species of epideaphic activity but, as in many other Gnaphosidae, its biology is mostly unknown.

##### Notes

1♂, 1 j.

#### Civizelotes ibericus

Senglet, 2012

C3D62818-B749-5E01-A84F-E0AD23CA91BD

https://wsc.nmbe.ch/lsid/urn:lsid:nmbe.ch:spidersp:046162

##### Distribution

Initially described as an Iberian endemism, typical of the southern half of the Iberian Peninsula, it has subsequently been cited from the Province of Álava (Ferrández 1996) and recently from southern France (Oger and Van Keer 2017). This is its first mention in Portugal. The dispersion of existing information suggests that this is due to the lack of suitable prospects.

##### Notes

7♂♂, 2♀♀, 14 jj.

#### Gnaphosa

Latreille, 1804

3F4AAF5B-E89C-5F82-A7CC-7B715DDF3D89

#### Gnaphosa sp.

C6B9605F-758A-5AF7-B51C-4315CE31226A

##### Distribution

Possibly *Gnaphosaartaensis* Simon, 1878, already reported for the District of Beja.

##### Notes

93 jj. Indet.

#### Haplodrassus

Chamberlin, 1922

8A6E2016-7D82-526A-9F6F-6DD6C80F047C

#### Haplodrassus rhodanicus

(Simon, 1914)

6C906B1B-9F88-52B0-861A-F66EE6249EEE

https://wsc.nmbe.ch/lsid/urn:lsid:nmbe.ch:spidersp:026980

##### Distribution

In the recent revision of the genus *Haplodrassus* Chamberlin, 1922, Bosmans et al. (2018) mention this species from several localities in the Iberian Peninsula; one of them in Portugal (Setúbal). This is their second citation for Portugal and the first mention for the District of Beja. The general data on *H.rhodanicus* place it in western Europe, North Africa and the Mediterranean islands. It is a small species that may go unnoticed in some samples.

##### Notes

16♂♂, 6♀♀, 7 jj.

#### Haplodrassus sp.

890A51F9-BBA6-55BD-9170-16A5CC6A1659

##### Notes

27 jj. indet.

#### Leptodrassus

Simon, 1878

550324E8-DCC8-53D7-B66C-33A00A393610

#### Leptodrassus albidus

Simon, 1914

C41B0EF2-82C8-58F7-8E2E-23782C20210D

https://wsc.nmbe.ch/lsid/urn:lsid:nmbe.ch:spidersp:027453

##### Distribution

Cited from the Mediterranean countries of southern Europe and also from the Canary Islands. Known from several localities on the Iberian Peninsula. Previously cited from the Castro Verde area (Silva 2004). It is found in areas near the coast and in sparsely vegetated or fallow crops.

##### Notes

9♂♂, 19 jj.

#### Marinarozelotes

Ponomarev, 2020

31C93FF9-8515-5D5B-9F10-C4D8FD4AD2F8

#### Marinarozelotes minutus

(Crespo, 2010)

AB137110-39EB-56EA-AEF6-0CF226C9EA02

https://wsc.nmbe.ch/lsid/urn:lsid:nmbe.ch:spidersp:043618

##### Distribution

Described in Crespo and Mendes (2010) from two localities in the District of Évora in cork oak forests. It was transferred to the genus *Marinarozelotes* Ponomarev (2020) by Ponomarev and Shmatko (2020). Its presence in Castro Verde, albeit in small numbers, represents an extension of its range (although it is still a Portuguese endemism) and of its habitat, which must now include the steppe-like grasslands of the District of Beja. The lack of information about the species prevented the assessment of its conservation status according to the IUCN criteria (Branco et al. 2019).

##### Notes

4♂♂, 3♀♀, 2 jj.

#### Micaria

Westring, 1851

2C75C2C9-8B37-57EE-B6AD-3250FF7CEE3F

#### Micaria sp.

1702F101-34EE-5F9E-A372-8096E7F6F15F

##### Distribution

Specimens of doubtful specific assignment. The genus *Micaria* Westring, 1851 has numerous species; some are frequent in the Iberian Peninsula and there are several that have been previously reported for the District of Beja.

##### Notes

4 jj. indet.

#### Nomisia

Dalmas, 1921

F393F0D9-8460-52D8-A994-2511617C642F

#### Nomisia exornata

(C. L. Koch, 1839)

3C851D13-87B8-56EC-A0E0-4A593968CE90

https://wsc.nmbe.ch/lsid/urn:lsid:nmbe.ch:spidersp:027627

##### Distribution

Widespread in all the countries of the Mediterranean rim (Europe and Africa), reaching central Asia. It is a common, frequent and abundant species, widely cited throughout the Iberian Peninsula (de Biurrun et al. 2019) and previously mentioned in Castro Verde (Silva 2004) and other places in the District of Beja.

##### Notes

2♂♂, 81 jj.

Numerous specimens have been captured that we have included in this species; but hardly any adults have been captured, probably because their breeding phenology in Castro Verde does not coincide with the dates when the samples were taken.

#### Setaphis

Simon, 1893

F0CF8AAD-1FD2-50FB-A885-5E6F2AB69870

#### Setaphis carmeli

(O. Pickard-Cambridge, 1872)

B27CB07A-99FE-5458-BBE4-157D871F5721

https://wsc.nmbe.ch/lsid/urn:lsid:nmbe.ch:spidersp:027946

##### Distribution

It is a common species throughout the Mediterranean Basin. It is usually frequent and abundant throughout the Iberian Peninsula. It has already been reported for Castro Verde (Silva 2004) and from other localities in the District of Beja and the rest of Portugal. Although its biology is not known in detail, *S.carmeli* has been seen climbing on vegetation, especially in citrus plantations, in search of prey.

##### Notes

18♂♂, 8♀♀, 46 jj.

#### Zelotes

Gistel, 1848

A011DAF8-C530-5F40-B3A8-1B9490315C98

#### Zelotes fulvopilosus

(Simon, 1878)

CC0B4DF9-3D0B-5C79-A710-130877B94E0A

https://wsc.nmbe.ch/lsid/urn:lsid:nmbe.ch:spidersp:028211

##### Distribution

Data on this species are concentrated in Portugal, Spain and France. In the Iberian Peninsula, it has been mentioned from numerous localities; one of them in the District of Beja (Carvalho et al. 2011). Probably the data we now provide from Castro Verde represent an accidental capture in the pasture area, a vagrant from one of the nearby habitats where a stable population could be found. There are no consistent data to confirm these impressions.

##### Notes

1♂

#### Zelotes sp. (New species)

B41BDEB0-6590-54BD-A37D-B6D544FB69A9

##### Notes

2♂♂

This is a species new to the genus that will be described in a forthcoming article.

#### Zelotes sp.

1BC1569A-4C54-56D1-BC01-7D3B7357DBA2

##### Notes

93 jj. indet.

Specimens of doubtful specific assignment. They may correspond to the genus *Zelotes* Gistel, 1848 and/or *Civizelotes* Senglet, 2012.

#### Linyphiidae

Blackwall, 1859

42087F2C-1B7A-5D21-9E85-1AC1F58135CA

#### Linyphiidae sp.

18FF3FDC-7D85-59EA-BEDD-2B42D1624465

##### Notes

1028 jj. Indet.

#### Agyneta pseudorurestris

Wunderlich, 1980

53CFF3F8-3587-5013-885F-BC866532E225

https://wsc.nmbe.ch/lsid/urn:lsid:nmbe.ch:spidersp:011703

##### Distribution

*A.pseudorurestris* has a typically Mediterranean distribution and is one of the most frequent and abundant species of the genus *Agyneta* Hull, 1911. It is reported for numerous localities in the Iberian Peninsula; also in the District of Beja. It produces small space webs, especially in herbaceous environments, very close to the ground; although it is also found in wooded environments linked to the shrub layer.

##### Notes

248♂♂, 264♀♀, 893 jj.

The juvenile forms are hardly distinguishable from other related species of the same genus; since no adults of other *Agyneta* species have been caught, we have opted to identify all of them as *A.pseudorurestris*.

#### Centromerus

Dahl, 1886

51B10880-40D1-52C4-BA11-E05A5F5BAE53

#### Centromerus phoceorum

Simon, 1929

8D394E9A-A020-5613-BBF8-FD74573955B9

https://wsc.nmbe.ch/lsid/urn:lsid:nmbe.ch:spidersp:039399

##### Distribution

Species localised in the western countries of the Mediterranean area (Portugal, Spain, France, Algeria and Tunisia). There are limited, though scattered, data from the Iberian Peninsula. Most of them are located in Portugal; one of them in the District of Beja (Cardoso 2004).

##### Notes

2♀♀

#### Diplocephalus

Bertkau, 1883

EFB7E984-3074-5478-8A4F-607E465160A9

#### Diplocephalus graecus

(O. Pickard-Cambridge, 1873)

57B236E7-3CCC-59C4-90D7-35AE583CE053

https://wsc.nmbe.ch/lsid/urn:lsid:nmbe.ch:spidersp:010058

##### Distribution

Like the previous species, this is a Mediterranean species, although it is also found in some central European countries. In the Iberian Peninsula, records are concentrated in its southern half and in the coastal areas of the Mediterranean (de Biurrun et al. 2019). It has been mentioned from several Portuguese districts, including Beja.

##### Notes

61♂♂, 51♀♀, 109 jj.

#### Diplocephalus marijae

Bosmans, 2010

569860AD-A123-5751-821A-494FDD7483E0

https://wsc.nmbe.ch/lsid/urn:lsid:nmbe.ch:spidersp:043252

##### Distribution

There are very limited data on this species located on the Iberian Peninsula (Spain and Portugal), the Balearic Islands and Morocco. All data are collected in Bosmans et al. (2010). Data from Portugal are scattered from north to south; several correspond to localities in the District of Beja. Its biology is unknown; it seems to be a scarcely abundant species, but frequent in sunny and warm habitats.

##### Notes

2♂♂, 2♀♀

Possibly some of the juveniles identified as *D.graecus* correspond to this species.

#### Erigone

Audouin, 1826

6E04FC4B-0DDE-50AA-9923-AC195019BDB6

#### Erigone dentipalpis

(Wider, 1834)

7EA8BBE8-DBC4-59BF-A6B5-657F6EFFCA0C

https://wsc.nmbe.ch/lsid/urn:lsid:nmbe.ch:spidersp:010424

##### Distribution

It is a very common species, widespread throughout the Palaearctic Region. There are numerous records in Spain and Portugal (de Biurrun et al. 2019), including two localities in the District of Beja.

##### Notes

1♂

#### Microctenonyx

Dahl, 1886

7CB0D102-86A8-55FB-AB8A-5C86C3ADF28E

#### Microctenonyx subitaneus

(O. Pickard-Cambridge, 1875)

707B02D2-675E-50C4-BFFF-C2BD8C5F34E7

https://wsc.nmbe.ch/lsid/urn:lsid:nmbe.ch:spidersp:011797

##### Distribution

This is a diminutive Linyphiidae, especially localised in the western Palearctic area, but introduced in other countries (Nentwig et al. 2023). It is reported for the District of Beja and other localities in Portugal and Spain. It is frequent, although not very abundant, in soil samples, to which it seems to be closely linked.

##### Notes

2♂♂, 3♀♀, 4 jj.

#### Oedothorax

Bertkau, 1883

CB4439AF-1650-54D0-AFC9-AA12CE583863

#### Oedothorax fuscus

(Blackwall, 1834)

A07DD7EA-675A-51CC-999C-2CE4E56F3134

https://wsc.nmbe.ch/lsid/urn:lsid:nmbe.ch:spidersp:012090

##### Distribution

Generally widespread throughout Europe and North Africa. It is a common species. Already cited from the District of Beja (Cardoso 2004), it is also known from other localities in Portugal and Spain; it has also been mentioned in the Balearic Islands.

##### Notes

2♂♂, 2♀♀

#### Ouedia

Bosmans & Abrous, 1992

F19E0E4D-24E5-50D2-9428-035F24665017

#### Ouedia rufithorax

(Simon, 1881)

D8B4FC88-3705-5A21-9A2F-D977AFF84878

https://wsc.nmbe.ch/lsid/urn:lsid:nmbe.ch:spidersp:012184

##### Distribution

It is found in the westernmost part of the Mediterranean zone. It has been previously reported for the District of Beja.

##### Notes

1♂, 3♀♀

It is a very characteristic species; it is easy to recognise by its dark red colour and some characteristics of its genitalia.

#### Palliduphantes

Saaristo & Tanasevitch, 2001

DF7E406A-BF0E-54D2-81D1-56FD2C2D58C5

#### Palliduphantes stygius

(Simon, 1884)

0D931BAC-09C5-537C-BE6B-9BF1A1200649

https://wsc.nmbe.ch/lsid/urn:lsid:nmbe.ch:spidersp:011312

##### Distribution

It has been reported for several localities in the District of Beja (Cardoso 2004), but it is also known from other parts of Portugal, France and Spain. It produces inconspicuous webs in low vegetation.

##### Notes

1♂

#### Pelecopsis

Simon, 1864

A8810123-05C2-584B-9EC6-8EFD2725CAA5

#### Pelecopsis bucephala

(O. Pickard-Cambridge, 1875)

78E2AF2F-3C6A-540A-9981-A9D202512E60

https://wsc.nmbe.ch/lsid/urn:lsid:nmbe.ch:spidersp:012248

##### Distribution

Available data locate this species in the extreme west of the Mediterranean Area (France, Spain, Portugal, Morocco, Algeria and Mediterranean Islands; Nentwig et al. (2023)). It is reported for various parts of the District of Beja, but is scarcely represented in the Castro Verde area.

##### Notes

2♀♀, 1 j.

#### Pelecopsis inedita

(O. Pickard-Cambridge, 1875)

D66E9CC7-87D6-5683-8D6D-BF1B711D75D3

https://wsc.nmbe.ch/lsid/urn:lsid:nmbe.ch:spidersp:012264

##### Distribution

In general, its range, as the previous species, corresponds to the western countries of the Mediterranean Basin. In the Iberian Peninsula, it is frequent and abundant, being known from numerous localities. It has also been reported for the District of Beja. In the Castro Verde area, the species appears to be more tolerant to the epiphytic habitat conditions than *P.bucephala*, having been collected in far greater numbers than the latter species. Like other *Pelecopsis* Simon, 1864, it forms abundant populations in the soil mulch (mosses, leaf litter and other plant detritus).

##### Notes

30♂♂, 25♀♀, 55 jj.

#### Prinerigone

Millidge, 1988

5C4A24D1-47F4-5592-AA4F-15C98E7B462B

#### Prinerigone vagans

(Audouin, 1826)

65622D4F-B600-5E64-A133-E2D7F3C5751C

https://wsc.nmbe.ch/lsid/urn:lsid:nmbe.ch:spidersp:012437

##### Distribution

Species known from all countries in the Mediterranean belt, but extending into Asia, as far east as China. It has already been cited from the District of Beja in previous works. There are numerous records throughout the Iberian Peninsula (de Biurrun et al. 2019). It is a common species, but not very abundant.

##### Notes

3♂♂, 2♀♀

#### Styloctetor

Simon, 1884

3C214EF7-C39A-58A2-A6F3-F2FAD7916138

#### Styloctetor romanus

(O. Pickard-Cambridge, 1873)

1F2F75F0-53AB-5BE3-AAE6-4E7BCD3525D7

https://wsc.nmbe.ch/lsid/urn:lsid:nmbe.ch:spidersp:012780

##### Distribution

*S.romanus* also has a wide distribution area, from Europe to China; with a known range that extends further north than the Mediterranean latitude, being found in some central European countries and Russia (World Spider Catalog 2023). It has already been recorded from the District of Beja (de Biurrun et al. 2019). In the Iberian Peninsula, it is a common species; it appears frequently in regular surveys of the epiedaphic strata (Roberts 1987), although there is not usually a great abundance of individuals.

##### Notes

1♀, 1 j.

#### Tapinocyba

Simon, 1884

F0919915-D7E6-5FEB-BBC9-F94C2E481C82

#### Tapinocyba algirica

Bosmans, 2007

6E753222-8360-50E2-9FAC-B11041369AD6

https://wsc.nmbe.ch/lsid/urn:lsid:nmbe.ch:spidersp:041833

##### Distribution

This species is distributed across the western Mediterranean area (Spain, Portugal and Algeria). Few localities are known from Iberia (Bosmans et al. 2010, Cárdenas and Barrientos 2011). This is the first record for the District of Beja.

##### Notes

8♂♂, 2♀♀, 2 jj.

#### Tenuiphantes

Saaristo & Tanasevitch, 1996

1FBFB5F3-BD2F-5124-9B26-3485945D493E

#### Tenuiphantes sp.

9536E91C-2C52-53B0-A009-7C5A71C395D4

##### Distribution

Although *Tenuiphantestenuis* (Blackwall, 1852) has already been cited from the District of Beja, there are also other species of the same genus that may be present in Castro Verde, so we cannot confidently assign these juveniles to any of them.

##### Notes

7 jj. indet.

Specimens of doubtful specific assignment.

#### Walckenaeria

Blackwall, 1833

A7757EEB-21E4-502F-8179-DB64AEB6C5D1

#### Walckenaeria cucullata

(C. L. Koch, 1836)

43060D33-1F61-5979-A3F2-0A90C4374779

https://wsc.nmbe.ch/lsid/urn:lsid:nmbe.ch:spidersp:013270

##### Distribution

This species has been recorded throughout Europe. In the Iberian Peninsula, it has only been mentioned from some localities in the northern fringe (Cantabrian and western Pyrenees) (de Biurrun et al. 2019). The data we now offer from Castro Verde are the first record of this species in Portugal. The morphological features of the captured male are unequivocal, suggesting that (although it may be considered a rare species in the southern half of the Iberian Peninsula) there must be scattered populations in intermediate localities, which link with the position of the current citation.

##### Notes

1♂

#### Liocranidae

Simon,1897

7AC8B59F-F90E-5848-9E87-7690B281F68A

#### Agraecina

Simon, 1932

CD08D82A-BADC-58DA-816C-C7C588214285

#### Agraecina lineata

Simon, 1878)

DB6589B8-FCB5-5CFD-BE5A-35C0DBD9FD15

https://wsc.nmbe.ch/lsid/urn:lsid:nmbe.ch:spidersp:024142

##### Distribution

It is a widespread species in the countries bordering the Mediterranean, especially in the western Mediterranean. In Portugal, it has been reported for several localities north of Lisbon and Coimbra (de Biurrun et al. 2019). This is the first record for the District of Beja. It is also known from Morocco and Algeria. It is a medium-sized spider that lives a lapidiform life, which probably makes it difficult to capture and is probably more frequent in Castro Verde than suggested by the data.

##### Notes

1♂

#### Lycosidae

Sundevall,1833

284FFEB3-D73F-570A-9F23-25BAD8441F71

#### Lycosidae sp.

7304A7DD-1317-5423-BA20-BFC923D5462F

##### Notes

188 jj. indet.

#### Alopecosa

Simon, 1885

72CE059C-A95B-569D-8D43-B6ACB34DE102

#### Alopecosa albofasciata

(Brullé, 1832)

A8902524-C337-5084-BAD3-698581CC78C0

https://wsc.nmbe.ch/lsid/urn:lsid:nmbe.ch:spidersp:017375

##### Distribution

It is a common species in the Iberian Peninsula. It has already been reported for the District of Beja. Its general distribution affects the countries of the Mediterranean Basin, from Portugal to central Asia. It forms abundant populations and moves on the ground in search of prey or mating partners. They are common in grassy environments.

##### Notes

3♂♂, 2 jj.

#### Hogna

Simon, 1885

A1729C96-7131-5CCE-86CE-458875A24694

#### Hogna radiata

(Latreille, 1817)

AB08071D-4D5A-5B98-B093-8FEF9F11FC43

https://wsc.nmbe.ch/lsid/urn:lsid:nmbe.ch:spidersp:018101

##### Distribution

Its distribution encompasses all the countries of the Mediterranean Basin, reaching to central Asia. It is one of the most common species in open, steppe and grassland environments in Mediterranean countries. It has been previously reported for the District of Beja. Adults are usually more static and take refuge under large stones for copulation, laying and care of their young; their activity is usually nocturnal, sheltered from their usual predators (generally birds).

##### Notes

16 jj.

In the Iberian fauna, the morphological and pigmentary features allow the juveniles to be identified to this species with little margin for error.

#### Pardosa

C. L. Koch, 1847

915791F7-C4B0-560F-9600-DE90518416DC

#### Pardosa proxima

(C. L. Koch, 1847)

793776B2-A6B1-5B41-8CA6-98DBB35EC84E

https://wsc.nmbe.ch/lsid/urn:lsid:nmbe.ch:spidersp:018870

##### Distribution

It is a Palaearctic species. It has already been reported for the District of Beja. As in the case of the genus *Alopecosa* Simon, 1885, the species of *Pardosa* C. L. Koch, 1847 form abundant populations, very localised in time, which swarm ostensibly in herbaceous environments. *P.proxima* is one of the most common species in the Iberian Peninsula.

##### Notes

5♀♀, 15 jj.

#### Mimetidae

Simon,1881

D6B2B2D6-B21C-5FCE-B671-DC82F262A341

#### Ero

C. L. Koch, 1836

2B65EC8C-3DA8-53EA-92F2-566F98DB1E23

#### Ero aphana

(Walckenaer, 1802)

47446E97-C314-5AD0-BBFD-6507556A0AD4

https://wsc.nmbe.ch/lsid/urn:lsid:nmbe.ch:spidersp:005666

##### Distribution

It is found in the temperate and warm countries of the Palaearctic Region. It has been reported for several localities in Spain and Portugal; also in the District of Beja. It is common on shrubby vegetation, where there are numerous webs of Araneidae (of which they are kleptoparasites); but it is also found in grasslands, especially if there are tall grasses (where some Araneidae also settle). It is considered a common species.

##### Notes

4♂♂, 2♀♀

#### Miturgidae

Simon, 1886

3D1DBA1E-D910-56E1-956D-A4513F9B6676

#### Zora

C. L. Koch, 1847

CD4A0CC7-B07A-565C-A05C-43FF744C8956

#### Zora silvestris

Kulczyński, 1897

B4F69414-4B47-54D7-91FB-ED78F5C3A07C

https://wsc.nmbe.ch/lsid/urn:lsid:nmbe.ch:spidersp:028561

##### Distribution

This species is recorded from all western Palaearctic countries, excluding Russia and Turkey. It has already been recorded from the District of Beja (Cardoso 2004), although it is the only record from Portugal. In Spain, it has only been reported for the Pyrenees of Lleida (Crespo et al. 2018). This disparity in its location may be due to the rarity of the species or to the absence of data attesting to its presence in intermediate localities.

##### Notes

2♀♀

In general, the species of the genus *Zora* C. L. Koch, 1847 have closely-related genitalia and somatic characters, which makes identification very difficult and, with it, increases the number of possible errors. A thorough revision of the species of this genus seems desirable.

#### Nemesiidae

Simon,1889

2B918461-1F2C-5A13-AD18-A743C1124ADD

#### Iberesia

Decae & Cardoso, 2006

3194E635-A00C-5797-AB0D-600C002163AE

#### Iberesia sp.

B23B2925-E1A9-5B03-9152-BA4A0984FDAE

##### Notes

1 j. indet.

Specimen of doubtful specific assignment.

#### Oonopidae

Simon,1890

F3034E69-E66D-5ED6-97C2-9163A04EBFFA

#### Oonops

Templeton, 1835

F1979DAD-7283-5933-908E-CDA207B171CD

#### Oonops tubulatus

Dalmas, 1916

C321D3EB-AA4E-5503-9AD0-F01AAA3366F5

https://wsc.nmbe.ch/lsid/urn:lsid:nmbe.ch:spidersp:005062

##### Distribution

Species reported for western Mediterranean countries (Portugal, Spain, Algeria and Italy). It has been reported for several localities in Portugal, including one in the District of Beja (Cardoso 2004).

##### Notes

4♀♀

The species was characterised by Machado (1941), who provides some figures to facilitate its recognition.

#### Silhouettella

Benoit, 1979

0B6A3F0B-CABE-5828-A806-BF63737A541C

#### Silhouettella loricatula

(Roewer, 1942)

B705AAE8-50E8-5AFF-9A90-4B6067D35881

https://wsc.nmbe.ch/lsid/urn:lsid:nmbe.ch:spidersp:004893

##### Distribution

This species is localised in the countries forming the shores of the Mediterranean, from Portugal to Turkey. It has already been reported for the District of Beja. It is undoubtedly a thermophilic species, which is why it is considered rare in central Europe. However, in warmer, steppe-like areas (including North Africa), it is common amongst plant debris, forming large populations in some cases.

##### Notes

3♂♂

*S.loricatula* is a singular species, small in size, but its opisthosomal protective shields give it an armoured appearance.

#### Oxyopidae

Thorell,1869

1D346036-3D9F-5DA2-92B1-C4285F83BC1A

#### Oxyopes

Latreille, 1806

B0230F60-539D-5DBD-BDD8-44AB7B14362F

#### Oxyopes heterophthalmus

(Latreille, 1804)

E4018C73-0D6A-5A0D-8462-F0840CD32C72

https://wsc.nmbe.ch/lsid/urn:lsid:nmbe.ch:spidersp:019996

##### Distribution

One of the most common species of the genus *Oxyopes* Latreille, 1804 and with a very wide distribution, comprising the entire temperate and warm zone of the Palaearctic Region. It as been cited from numerous localities in Portugal and it has also been mentioned in the District of Beja (Cardoso et al. 2009).

##### Notes

7♂♂, 2♀♀, 222 jj.

*Oxyopesheterophthalmus* is usually distinguished by a very pronounced brown pigmentation that separates it from another of the most common species, *Oxyopeslineatus* Latreille, 1806. The juveniles we have identified correspond to this typology; however, given the variability observed, it is possible that some specimens do not correspond to this species.

#### Oxyopes sp.

A208EA08-CE71-50B0-B3B3-ACD49F6E4C0F

##### Distribution

Possibly *Oxyopeslineatus* Latreille, 1806.

##### Notes

21 jj. indet.

#### Philodromidae

Thorell,1869

F3406899-76DF-5B1D-8BC6-A44AC783B363

#### Pulchellodromus

Wunderlich, 2012

176CFD93-33E9-5E34-91B0-407239E78604

#### Pulchellodromus pulchellus

(Lucas, 1846)

75A91CB8-C85A-5613-B69F-C19F1692E772

https://wsc.nmbe.ch/lsid/urn:lsid:nmbe.ch:spidersp:029922

##### Distribution

Species cited from Mediterranean European countries, from Portugal to Turkey. It has been reported for numerous localities in Spain and Portugal; in the latter, it has also been mentioned from the District of Beja. It usually climbs on vegetation, both herbaceous and shrubby. Its rapid tanatosis reaction and its speed of movement may be the cause of an underestimation in the results of the sampling carried out in Castro Verde.

##### Notes

6♂♂, 1♀, 14 jj.

#### Thanatus

C. L. Koch, 1837

13FB5110-D3D7-5A3F-A6B4-254D93268097

#### Thanatus vulgaris

Simon, 1870

37CF16B1-FADB-5AD8-9F43-48DE309C34CB

https://wsc.nmbe.ch/lsid/urn:lsid:nmbe.ch:spidersp:030073

##### Distribution

Its distribution is wider than the previous species, as it also includes North America. It has already been reported for the District of Beja, as well as from many other localities in Portugal and Spain. It is a very common species in arid places, moving quickly on the ground during sunny hours; juvenile forms tend to climb and move through the vegetation.

##### Notes

2♂♂, 1♀, 155 jj.

#### Tibellus

Simon, 1875

7257BC03-9D7E-5792-8460-4D80A1B6F168

#### Tibellus macellus

Simon, 1875

3562B3B7-803B-53B2-B960-0B5D548E0DA2

https://wsc.nmbe.ch/lsid/urn:lsid:nmbe.ch:spidersp:030100

##### Distribution

It can be considered a European species, although it has not been found in some Nordic countries. There are few records in the Peninsula and only one in Portugal (Vila Real; Machado (1941)). This is the first record for the District of Beja.

##### Notes

1♀

#### Pisauridae

Simon,1890

5BD9DFC8-B223-5AA0-B4B8-33885D82F2F9

#### Pisaura

Simon, 1886

EB35886E-52A2-5775-B748-1DB106259133

#### Pisaura mirabilis

(Clerck, 1757)

BC50539E-7008-5078-84A4-68D250B92923

https://wsc.nmbe.ch/lsid/urn:lsid:nmbe.ch:spidersp:019759

##### Distribution

One of the most common species throughout the Palaearctic Region. Previously reported for the District of Beja. It forms abundant populations on the wet banks of ponds, rivers and streams, moving amongst herbaceous and shrubby vegetation. It is not usually found in dry and arid places, so its presence in Castro Verde depends to a large extent on the humidity of the soil and the position of each plot.

##### Notes

1♀, 2 jj.

#### Salticidae

Blackwall,1841

F9BCBEB9-BB42-5129-A449-437893E31999

#### Chalcoscirtus

Bertkau, 1880

AF294409-3820-577F-B57F-2F9BDCB2A92B

#### Chalcoscirtus infimus

(Simon, 1868)

A51038A0-9477-539D-8766-5529B5382853

https://wsc.nmbe.ch/lsid/urn:lsid:nmbe.ch:spidersp:032664

##### Distribution

It is widespread in the Palaearctic Region, occupying central Europe and the Mediterranean area as far as central Asia. There are many records scattered throughout Spain and Portugal, including several in the District of Beja. *C.infimus* is a diminutive Salticidae that moves at the epiedaphic level and in the lowest strata of vegetation. In Castro Verde, there are stable populations as attested by the data obtained in this survey, which probably shows a pre-breeding stage, given the absence of females and the high number of adult and juvenile males.

##### Notes

43♂♂, 175 jj.

#### Euophrys

C. L. Koch, 1834

EAECE86C-9D0A-5336-90E6-EC38F05B0F19

#### Euophrys herbigrada

(Simon, 1871)

6BAC5A64-7BDC-5DD0-A50B-AD608ED4C012

https://wsc.nmbe.ch/lsid/urn:lsid:nmbe.ch:spidersp:033277

##### Distribution

Found in the western part of the Palaearctic Region, widespread in central European and Mediterranean countries. It is a common species, well represented in Spain and Portugal, with records from the District of Beja (Cardoso 2004). Stable and abundant populations develop in the Castro Verde grasslands.

##### Notes

35♂♂, 18♀♀, 34 jj.

#### Heliophanus

C. L. Koch, 1833

03AA34AB-9033-5433-BAFE-23FC1D5E9842

#### Heliophanus lineiventris

Simon, 1868

17146272-450C-58A6-9026-793979372CCC

https://wsc.nmbe.ch/lsid/urn:lsid:nmbe.ch:spidersp:033823

##### Distribution

Widespread throughout the Palaearctic Region, except for its coldest areas. It has already been reported for the District of Beja (Cardoso et al. 2009, Barrientos et al. 2020), as well as from many other localities in Portugal and Spain Barrientos et al. (2020); this is the first data for the species from the Castro Verde area (de Biurrun et al. 2019).

##### Notes

1♂, 2♀♀, 1 j.

#### Pellenes

Simon, 1876

26A888F7-209C-5CCC-8E12-064950260F74

#### Pellenes nigrociliatus

(Simon, 1875)

24E3B3B0-C592-5498-BE5F-5C7C66A29C92

https://wsc.nmbe.ch/lsid/urn:lsid:nmbe.ch:spidersp:035300

##### Distribution

Native to the north-Mediterranean Basin in the Palaearctic Region, from Portugal to China. There are few records in the Iberian Peninsula, but it has already been recorded from the District of Beja. It is a common species that moves through shrubs and tall grasses.

##### Notes

2♀♀

#### Phlegra

Simon, 1876

A2A16D8C-2F3A-5161-92B9-EA2AB657283B

#### Phlegra bresnieri

(Lucas, 1846)

CD510535-5D80-583E-827C-5F2B6286694B

https://wsc.nmbe.ch/lsid/urn:lsid:nmbe.ch:spidersp:035566

##### Distribution

This is a circum-Mediterranean species, also found in some African countries. In the Iberian Peninsula, it is the most common species of the genus *Phlegra* Simon, 1876. It is known from several localities in Portugal, including some in the District of Beja. It is abundant on herbaceous and shrubby vegetation in sunny and arid areas; it is a species well represented in Castro Verde.

##### Notes

24♂♂, 49♀♀, 3 jj.

#### Salticus

Latreille, 1804

932D3635-D149-5B50-9E8E-D7797A4FD837

#### Salticus propinquus

Lucas, 1846

7BB4CB11-7A06-58C9-9C10-E1C2CE860320

https://wsc.nmbe.ch/lsid/urn:lsid:nmbe.ch:spidersp:036028

##### Distribution

It is found in the countries bordering the Mediterranean, from Portugal to Turkey, both in Europe and North Africa. The data available for the Iberian Peninsula, although scarce, are scattered throughout the country. It has been previously reported for the District of Beja.

##### Notes

1♀

#### Talavera

G. W. Peckham & E. G. Peckham, 1909

FA87C7F3-B310-5517-862B-AD0ADE9DECCD

#### Talavera petrensis

(C. L. Koch, 1837)

D7843CCA-FA58-5E36-A39A-B45A42AF0DD4

https://wsc.nmbe.ch/lsid/urn:lsid:nmbe.ch:spidersp:036451

##### Distribution

It is a typical species of the Palaearctic Region, widespread throughout Europe. There are several citations from Portugal, but this is the first mention in the District of Beja. It is a frequent and abundant species; especially in meadows of a certain altitude and in wooded and shrubby areas, moving over vegetation.

##### Notes

1♂

#### Scytodidae

Blackwall,1864

8532F4C5-C48E-5D93-8EE3-BB4547F2A792

#### Scytodes

Latreille, 1804

2FE173D5-6070-5660-88C6-89262A2BF9F1

#### Scytodes sp.

42CCF7F7-88B6-5738-A31A-783B0691CE86

##### Notes

1j. indet.

Specimen of doubtful specific assignment.

#### Synaphridae

Wunderlich,1986

C99FC466-AB0B-5F73-8E52-26445CA95686

#### Synaphris

Simon, 1894

71F4D5D0-41D1-5D91-B108-C2B69C5F12CF

#### Synaphris saphrynis

Lopardo, Hormiga & Melic, 2007

CF79764B-2D77-5435-BFFF-FAF00BF7FED4

https://wsc.nmbe.ch/lsid/urn:lsid:nmbe.ch:spidersp:039849

##### Distribution

Available data abound in considering this species as an Iberian endemism, although a citation from the Selvagens Islands (Crespo et al. 2009) would considerably extend its range. Recently, Barrientos et al. (2020) cite it in the District of Beja. Our data show that, in certain habitats, *S.saphrynis* can be relatively abundant and also that, due to its small size and possible crypticism, it can go unnoticed in surveys.

##### Notes

42♂♂, 32♀♀, 20 jj.

There is considerable morphological affinity between the described species of the genus *Synaphris* Simon, 1894 whose distribution is centralised in the Mediterranean and Macaronesian Zone; the two species described from Madagascar would deserve special attention in a desirable revision of the family Synaphridae.

#### Tetragnathidae

Menge,1866

C047E20C-5D7C-5FE3-BA2E-CCDE261BBB1B

#### Tetragnatha

Latreille, 1804

8699566F-23D0-52A8-8EE1-916FACFC2B2E

#### Tetragnatha intermedia

Kulczyński, 1891

1FC65DF3-8BDD-5DD3-A545-4EE3778BFEB6

https://wsc.nmbe.ch/lsid/urn:lsid:nmbe.ch:spidersp:014289

##### Distribution

Species localised in Mediterranean countries, mainly in its western area (there are some data from Turkey). It has recently been reported for several localities in Portugal (Morano 2020). This is the first record for the District of Beja.

##### Notes

1♂, 1♀

#### Theridiidae

Sundevall, 1833

1BF22F14-D9B9-5776-9461-9694EE155827

#### Theridiidae sp.

B258F0BA-9484-5D96-BC59-990E94FCB809

##### Notes

7 jj. indet.; Specimens are too small to be assigned confidently to a genus.

#### Asagena

Sundevall, 1833

A63ABEE3-30B5-5837-AFB6-1FB6EFDBA769

#### Asagena phalerata

(Panzer, 1801)

DDF4576F-EEE1-57BC-80BA-89C648D28413

https://wsc.nmbe.ch/lsid/urn:lsid:nmbe.ch:spidersp:008101

##### Distribution

It is a typical species of the Palaearctic Region, abundant and very common in the Iberian fauna. Previously cited from Castro Verde (Silva 2004). It roams the ground in places poor in vegetation.

##### Notes

5♂♂, 23♀♀, 5 jj.

#### Dipoena

Thorell, 1869

EEF9C680-F5D1-5016-AD7B-F8E5775F29D2

#### Dipoena umbratilis

(Simon, 1873)

331B1BE9-05FE-59CF-B93E-8309C2E504CC

https://wsc.nmbe.ch/lsid/urn:lsid:nmbe.ch:spidersp:007505

##### Distribution

Species native to the western Mediterranean (Nentwig et al. 2023). However, there are hardly any data from the Iberian Peninsula, with only three records: two from Granada (Crespo et al. 2018) and one from a cave in Lisbon (Machado 1949). We can provisionally consider it a rare species that is now reported for the first time in the District of Beja.

##### Notes

2♂♂, 4jj.

#### Enoplognatha

Pavesi, 1880

7CE84761-C60A-5DCA-9819-5619ECFEBCD3

#### Enoplognatha diversa

(Blackwall, 1859)

17C4F5BC-29FA-58FA-A45E-5B195A1FDCD4

https://wsc.nmbe.ch/lsid/urn:lsid:nmbe.ch:spidersp:007547

##### Distribution

Nentwig et al. (2023) place this species in the western Mediterranean area; however, there are some data from its easternmost part. In the Iberian Peninsula, the localities where it has been found are mainly in its southern half; however, in Portugal, it has not been mentioned in Baixo Alentejo. This is the first record for the District of Beja. The specimens studied here have been identified following the revision of Bosmans and Van Keer (1999). *E.diversa* can be found in very diverse habitats.

##### Notes

2♂♂, 4♀♀

#### Euryopis

Menge, 1868

3711C4B3-523C-5480-96B6-DE460152F06F

#### Euryopis episinoides

(Walckenaer, 1847)

6CB8D9F7-2CD3-54DE-86EE-BE8C551B3452

https://wsc.nmbe.ch/lsid/urn:lsid:nmbe.ch:spidersp:007701

##### Distribution

*E.episinoides* is a common species easily found in open places and garrigues in the Mediterranean area. Previously cited in the District of Beja, these are the first data for the Castro Verde area.

##### Notes

1♂, 3 jj.

#### Neottiura

Menge, 1868

D2488BB9-8F82-55AD-96F1-FA9FE8EC4E84

#### Neottiura uncinata

(Lucas, 1846)

1B94E2B2-95A5-5F60-87BE-EF6D3090272A

https://wsc.nmbe.ch/lsid/urn:lsid:nmbe.ch:spidersp:007858

##### Distribution

*N.uncinata* is a typically Mediterranean species. There are several records from Portugal, but this is the first mention of it in the District of Beja. In the Castro Verde area, *N.uncinata* is one of the main species of the family Theridiidae, as it is found associated with herbaceous low vegetation habitats.

##### Notes

44♂♂, 67♀♀, 366 jj.

#### Paidiscura

Archer, 1950

3290C889-BCDE-522F-A1AC-06DBABA91951

#### Paidiscura pallens

(Blackwall, 1834)

711E4448-E082-58C3-8122-6FAF49644A50

https://wsc.nmbe.ch/lsid/urn:lsid:nmbe.ch:spidersp:007863

##### Distribution

One of the most frequent and common species of the family Theridiidae in the Palaearctic Region. There are data from practically all European (up to Siberia) and North African countries. Data from the Iberian Peninsula (de Biurrun et al. 2019) are distributed throughout the Iberian Peninsula; it has been previously cited for the District of Beja (Barrientos et al. 2020).

##### Notes

1♂

#### Phylloneta

Archer, 1950

40DB51D9-B059-5D50-A45B-0D51A75F9473

#### Phylloneta impressa

(L. Koch, 1881)

189E12A4-A209-5FD8-BC96-B724FA884FEB

https://wsc.nmbe.ch/lsid/urn:lsid:nmbe.ch:spidersp:008433

##### Distribution

Throughout the western Palaearctic area, *P.impresa* is one of the most frequent and abundant species of the family Theridiidae; it has also been cited from North America, so we can consider it a Holarctic species. Cardoso et al. (2009) and Barrientos et al. (2020) cite it from the District of Beja; this is the first data from the Castro Verde area. Its webs are spread amongst vegetation in shrub and herbaceous strata.

##### Notes

1 j.

#### Ruborridion

Wunderlich, 2011

B305E3DE-149A-544E-880A-9CDD265B296B

#### Ruborridion musivum

(Simon, 1873)

395C0D39-EE1F-5476-BC4E-E918CF1A3E00

https://wsc.nmbe.ch/lsid/urn:lsid:nmbe.ch:spidersp:007861

##### Distribution

It is common in western Europe and has been cited from different localities in Spain and Portugal (de Biurrun et al. 2019). However, this is the first record for the District of Beja. It is a diminutive theridid associated with the herbaceous and shrub layer.

##### Notes

1♀

#### Simitidion

Wunderlich, 1992

81BE02A7-F061-5A42-9ACF-2860A58E65A5

#### Simitidion simile

(C. L. Koch, 1836)

9EC4EE3C-0531-5F6F-9DAB-5404FEA797D4

https://wsc.nmbe.ch/lsid/urn:lsid:nmbe.ch:spidersp:008009

##### Distribution

A common species (Nentwig et al. 2023) throughout the western Palaearctic (Europe and North Africa), especially in Mediterranean countries. Previously cited (Barrientos et al. 2020) for the District of Beja, this is the first data for the Castro Verde area.

##### Notes

1 j.

*S.simile* has a characteristic pigment pattern that appears already in juvenile forms.

#### Steatoda

Sundevall, 1833

05CABC69-A6B4-504B-A7FE-D1D3EFE6B22E

#### Steatoda albomaculata

(De Geer, 1778)

340AFA4C-C5EA-5A88-9D18-A00FDE01A9A9

https://wsc.nmbe.ch/lsid/urn:lsid:nmbe.ch:spidersp:008019

##### Distribution

This is a common and abundant species throughout the Holarctic. There are numerous records throughout the Iberian Peninsula, one of them from Castro Verde (Silva 2004).

##### Notes

7 jj.

Juveniles already show the characteristic pigmentation pattern of the species.

#### Theridion

Walckenaer, 1805

980582C9-01DF-5ACF-ACA4-B27E0A23C80C

#### Theridion pinastri

L. Koch, 1872

A7624D0A-3F76-51BA-9D4E-B8EC4C5266F5

https://wsc.nmbe.ch/lsid/urn:lsid:nmbe.ch:spidersp:008611

##### Distribution

*T.pinastri* is widespread throughout Europe, reaching as far as the eastern Palaearctic zone. There are limited data from Portugal and this is the first record for the District of Beja. It is a frequent species in wooded areas (e.g. pine forests, holm oak forests) and also in herbaceous vegetation and low branches (Nentwig et al. 2023).

##### Notes

4♂♂, 15♀♀, 29 jj.

#### Thomisidae

Sundevall,1833

2172AE1C-17D0-5DE5-820F-0AB6659A7055

#### Thomisidae sp.

BF053EF6-AC08-559F-9108-EEFEBC06EB77

##### Notes

3339 jj indet.

Specimens of doubtful generic assignment. Many juvenile forms of Thomisidae (mainly of the genus *Xysticus* C. L. Koch, 1835 and *Ozyptila* Simon, 1864) move about on vegetation in search of food; there are no diagnostic characters specific to these stages.

#### Bassaniodes

Pocock, 1903

7790F269-2E8C-577A-876F-5194C0A97710

#### Bassaniodes bliteus

(Simon, 1875)

D6D3D9C6-2D55-57E2-A74E-3FC24F73C24D

https://wsc.nmbe.ch/lsid/urn:lsid:nmbe.ch:spidersp:031824

##### Distribution

Data on *B.bliteus* are available from several countries bordering the western Mediterranean. Although we still have few data, we can consider it a common species throughout the Iberian Peninsula. It has already been recorded in the District of Beja (Cardoso 2004) and in the Castro Verde area (Silva 2004). It is one of the smallest species of the genus; it is closely linked to the ground, where it goes unnoticed thanks to its cryptic colouring and its defensive staticity.

##### Notes

2♀♀

The morphological characters of this species have made it fluctuate between several genera (*Xysticus* C. L. Koch, 1835, *Ozyptila* Simon, 1864 and *Bassaniodes* Pocock, 1903).

#### Ozyptila

Simon, 1864

0EEC6BB2-698C-5336-90CE-3F3EAA05DCBE

#### Ozyptila pauxilla

(Simon, 1870)

C429B041-553E-5FB9-BB98-62EF4040759B

https://wsc.nmbe.ch/lsid/urn:lsid:nmbe.ch:spidersp:030891

##### Distribution

The general data place it in all the countries of the western Mediterranean (Europe and North Africa). We therefore assume that this is a common species in the Iberian Peninsula, with a wider distribution than currently known. This species, previously cited from the District of Beja (Bacelar 1940, Cardoso 2004), had not been mentioned from the Castro Verde study area. Like other *Ozyptila* Simon, 1864, it lives a cryptic life, linked to dry and stony soils; perhaps for this reason, it has been cited from few places in the Iberian Peninsula (de Biurrun et al. 2019).

##### Notes

70♂♂, 40♀♀, 52 jj.

#### Runcinia

Simon, 1875

89C2881F-6135-5F05-9AB7-A592330B5EF9

#### Runcinia grammica

(C. L. Koch, 1837)

46C03C8A-BA80-501B-92E3-3523C82FD2D5

https://wsc.nmbe.ch/lsid/urn:lsid:nmbe.ch:spidersp:031065

##### Distribution

It is one of the most common Thomisidae in Europe and North Africa, with a widespread distribution in the Iberian Peninsula (de Biurrun et al. 2019). It has been previously cited in the Castro Verde area (Silva 2004, Cardoso 2004). *R.grammica* forms abundant populations that colonise areas of reeds and other herbaceous plants in areas with a certain humidity.

##### Notes

81 jj.

#### Thomisus

Walckenaer, 1805

48F2FDB1-6AF7-57C8-B893-3DAC47B11ABA

#### Thomisus onustus

Walckenaer, 1805

1C83EFED-285B-50A3-89BE-84889D750373

https://wsc.nmbe.ch/lsid/urn:lsid:nmbe.ch:spidersp:031485

##### Distribution

A common species, widely distributed in Europe and North Africa. It is widespread throughout the Iberian Peninsula (de Biurrun et al. 2019). Although it has not been mentioned from the Castro Verde area, it has been mentioned from the District of Beja; we can assume that it is present throughout the area, especially in areas rich in grasslands.

##### Notes

3♂♂, 1♀, 5 jj.

#### Xysticus

C. L. Koch, 1835

ABD44D80-CEC0-51D0-8526-8CFC5615D807

#### Xysticus grallator

Simon, 1932

7E1F6A1D-6A90-5280-8487-1447AB0C1821

https://wsc.nmbe.ch/lsid/urn:lsid:nmbe.ch:spidersp:031910

##### Distribution

Known from Spain, Portugal and the islands of Corsica and Sardinia. *Xysticusgrallator* has reported from the District of Évora and from some localities in Spain: Cáceres, Ciudad Real, Madrid, Murcia and Alicante (Carrillo et al. 2016); all of them south of the Sistema Central. It has also been mentioned from the islands of Corsica and Sardinia. This is the first record for the District of Beja.

##### Notes

2♀♀

#### Xysticus nubilus

Simon, 1875

03CCE2E4-8B22-53B7-AB62-E3EBA6F2A3AB

https://wsc.nmbe.ch/lsid/urn:lsid:nmbe.ch:spidersp:032006

##### Distribution

Although it has not been reported for the Castro Verde area, it has been reported for the District of Beja (Cardoso 2004). There are numerous records from Portugal and Spain, especially in the south-western quadrant of the Peninsula with Atlantic influence. It has also been reported for other countries on the Mediterranean Basin.

##### Notes

9♀♀, 1 j.

#### Zodariidae

Thorell,1881

5452FE09-5474-5385-B19C-C252CCF316C0

#### Zodarion

Walckenaer, 1826

C04D5452-70E3-5F96-90AC-20C779077DD1

#### Zodarion jozefienae

Bosmans, 1994

7DB17319-5401-5DAD-9C56-6A8127F5CE7D

https://wsc.nmbe.ch/lsid/urn:lsid:nmbe.ch:spidersp:026079

##### Distribution

This species is an Iberian endemic; it has already been cited from the study area (Pekár et al. 2003, Silva 2004). It is common in the District of Beja, where most of the known data are concentrated. However, there are also data from the Algarve and Spain (Cáceres, Ciudad Real, Toledo, Huelva, Málaga and Logroño; de Biurrun et al. (2019)). It can be considered a localised species in the south-western quadrant of the Iberian Peninsula; data from the banks of the Ebro (Logroño: Rioja Alta; Fernández-Pérez (2016)) seem exceptional.

##### Notes

26♂♂, 1 jj.

#### Opiliones

Sundevall, 1833

71066300-4D21-5594-8A97-818EC27CCAF1

#### Phalangiidae

Latreille, 1802

EC38193A-37CD-5F4A-BF90-D6BA572D58ED

#### Dasylobus

Simon, 1878

C205D492-2A3D-5668-9DBE-2FED0C442125

#### Dasylobus ibericus

(Rambla, 1967)

95C256B9-06DE-5602-882F-788D7E65B205

Dentizacheus ibericus Rambla 1967, Rev. Biol., 6 (1-2): 25, fig. 14-16 [Type loc.: Torre de Moncorvo];
Dasylobus ibericus – Prieto 2003, Rev. Ibér. Aracn., 8: 132

##### Distribution

Iberian endemism described from three localities in the Douro River valley (border between Bragança, Viseu and Guarda) and one in the Algarve. The two localities provided are the first records for the Beja District.

##### Notes

2♂♂

The specimen from Monte da Chada (body length: 5.3 mm, femur 1 length: 4.3 mm) agrees with the morphology described by Rambla (1967), but the one from Monte da Albergaria (body length: 3.7 mm, femur 1 length: 3 mm) has poorly developed secondary sexual characters (frontal denticulation, cheliceral protuberances, robustness of femur 1), although the penial and palpal morphology confirm its description.

#### Sclerosomatidae

Simon, 1879

19E58C41-A82E-593E-9166-F54579965982

#### Homalenotus

Koch, 1839

76B01BA6-CE16-526C-BF1B-33E98D1B5AC5

#### Homalenotus buchneri

(Schenkel, 1936)

EE86218F-EDAC-57C6-9D88-51FD60E563A1

Parasclerosoma lusitanicum Roewer 1923, Weberkn. d. Erde: 706, fig. 878 [non *Homalenotuslusitanicus* (Kulczyński 1909)] [Type loc.: Coimbra];
Parasclerosoma buchneri Schenkel 1936, Zool. Anz. 116 (1–2): 24-27 [Type loc.: Ischia];
Homalenotus maroccanus Roewer 1957, Senck. biol., 38 (5/6): 336 [Type loc: Umgebung von Fez];
Homalenotus roeweri Kraus 1959, Mitt. zool. Mus. Berlin 35 (2): 301 [nom. nov. *P.lusitanicum*];
Homalenotus buchneri – Grasshoff 1959, Senck. biol. 40(5/6): 286

##### Distribution

Mediterranean species known from the Apennine Peninsula, the Balearic Islands and the south-western quadrant of the Iberian Peninsula, Coimbra being the only locality cited from Portugal (Roewer (1923) sub *Parasclerosomalusitanicum*). The numerous records provided would be the first citation for the Beja District. Additionally, from the ZUPV collection, we add the following records for the Beja District: [ZUPV-3322: 1♂] Mértola (perimeter), 29SPB167660, 90 m elev., Pedro Cardoso, 29-05-2003; [ZUPV-4505: 1♀] Almodóvar, 29SNB8352, 285 m elev., Luis Crespo, 05-11-2009; [ZUPV-4865: 1♂,2♀♀] Vale de Vargo, 29SPC395055, Emidio Machado, 20-08-2011.

##### Notes

14♂♂, 6♀♀, 57 jj.

#### Leiobunum

C. L. Koch, 1839

4E58ADA9-943D-5CB9-AAC4-141D71BEAF1A

#### Leiobunum sp.

C8352892-AC02-5CB4-A15D-E0D1F01B2463

##### Notes

2 jj. indet.

The extreme immaturity of the two specimens, a recently hatched pullus and another of 1.37 mm body length, prevents their determination.

## Analysis

### General patterns

**Figure 2.**
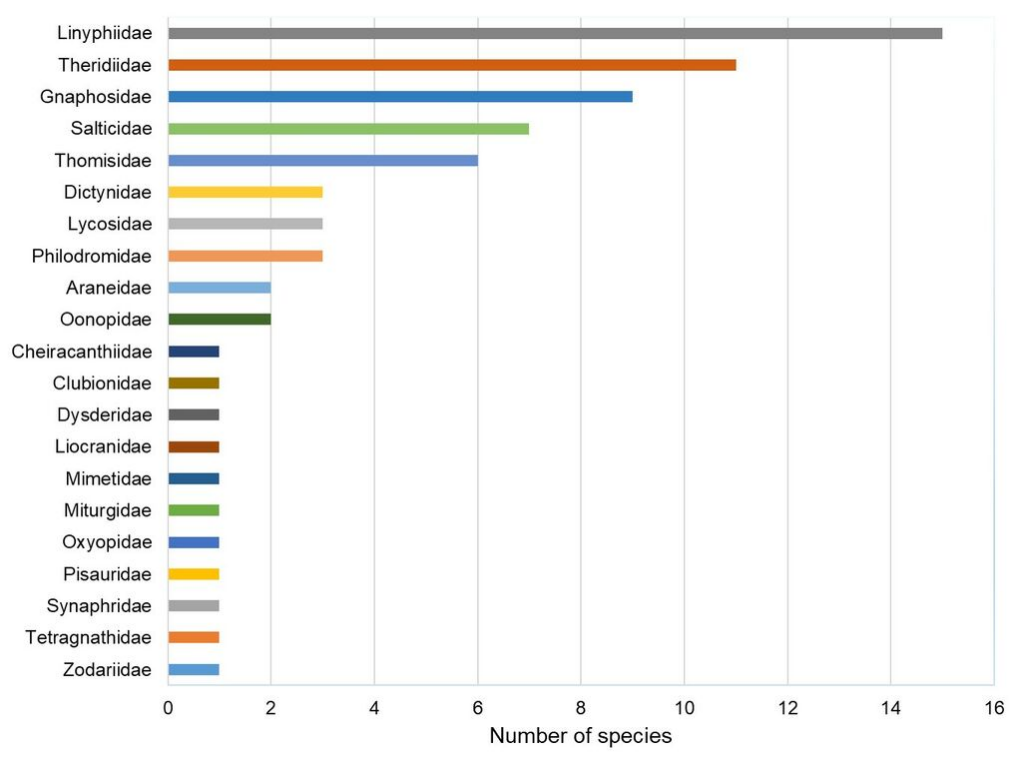
Number of spider species by family.

**Figure 3.**
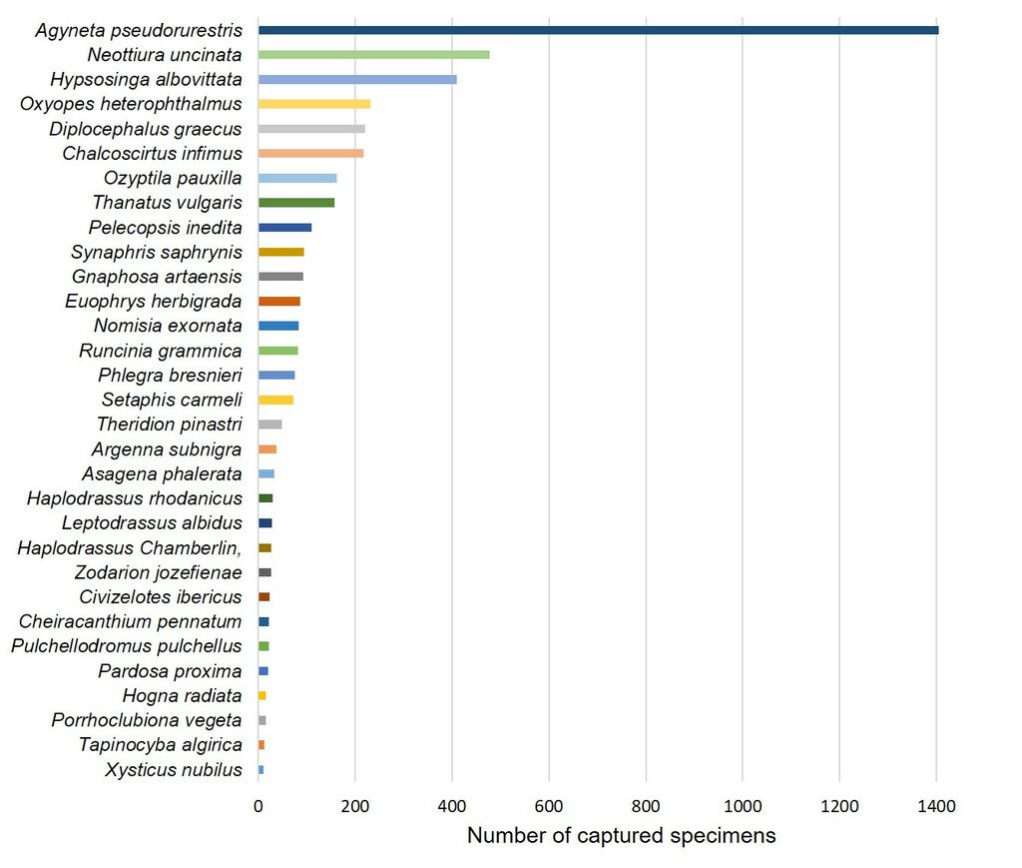
Frequency of the 27 main spider species in the Castro Verde grasslands (≥ 10 specimens captured).

In total, 9,694 spiders were collected in the Castro Verde SPA [768 males (♂♂), 722 females (♀♀) and 8,204 juveniles (jj)], which are distributed amongst 71 nominal species (Barrientos et al. 2023, Suppl. materials 1, 2), belonging to 25 families. Of these, the Linyphiidae, Theridiidae, Gnaphosidae, Salticidae and Thomisidae were the most diverse (with six or more species each); Dictynidae, Lycosidae, Philodromidae, Araneidae and Oonopidae were represented by two to three species, while the other eleven were represented by a single species (Figs 2, 3). Moreover, 81 harvestmen (16♂♂, 6♀♀ and 59jj) distributed amongst two species and three genera belonging to two families were collected (Barrientos et al. 2023, Suppl. materials 1, 2). Additionally 27 pseudoscorpions and eight mites were also collected (Barrientos et al. 2023, Suppl. material 1).

Most of the spider specimens collected were juvenile (84.6%). Morphological identification of immature specimens is very difficult in spiders. Although for most specimens it was possible to identify the family, for many, the identification to genus level was uncertain. For instance, there are 3339 juveniles of Thomisidae whose taxonomic position is doubtful between *Ozyptila* Simon, 1864 and *Xysticus* C. L. Koch, 1835. However, in some cases, juveniles were easily assigned to specific species, even without the presence of adults (although these involved only a few species); obviously, the specific identification of juveniles is reinforced by the presence of adults, suggesting the existence of reproducing populations. After excluding the samples that involved uncertainty, the total number of specimens identified to species level is 4,363 (Barrientos et al. 2023, Suppl. materials 1, 2). With these data, we can make a first assessment of the Castro Verde arachnocenosis.

### Relevant taxonomic and faunal data

From a taxonomic point of view, the most remarkable fact is the appearance of a new species that we include in the genus *Zelotes* Gistel, 1848 that will be described in a future taxonomical note on Iberian Gnaphosidae. From a faunistic perspective, the most remarkable fact is the first record for Portugal of the species *Argennasubnigra* (O.P.-Cambridge, 1861), *Civizelotesibericus* Senglet, 2012 and *Walckenaeriacucullata* (C. L. Koch, 1836). Several species are recorded for the first time for the District of Beja: *Cheiracanthiumpennatum* Simon, 1878, *Haplodrassusrhodanicus* (Simon, 1914), *Marinarozelotesminutus* (Crespo, 2010), *Tapinocybaalgirica* Bosmans, 2007), *Agraecinalineata* (Simon, 1878), *Tibellusmacellus* Simon, 1875, *Talaverapetrensis* (C. L. Koch, 1837), *Tetragnathaintermedia* Kulczyński, 1891, *Dipoenaumbratilis* (Simon, 1873), *Enoplognathadiversa* (Blackwall, 1859), *Neottiurauncinata* (Lucas, 1846), *Ruborridionmusivum* (Simon, 1873), *Theridionpinastri* L. Koch, 1872 and *Xysticusgrallator* Simon, 1932 and one genus: *Anyphaena* Sundevall, 1833. More precise details are given in the checklist.

## Discussion

Our assessment of the Arachnida fauna in open fallows and pastures grazed by sheep and cattle contributes to the knowledge of Arachnida species in Portugal and highlights the importance of Castro Verde SPA for biodiversity conservation. The 71 spider species recorded are distributed amongst 25 of the 49 families known to occur in Portugal. It is also relevant that four of the species recorded in this study are Iberian endemics (Branco et al. 2019) and the range of the strict Portuguese endemism *M.minutus* (Crespo, 2010) was extended to the Beja District (de Biurrun et al. 2019).

The dominant species was a small Linyphiidae, *Agynetapseudorurestris* Wunderlich, 1980, very frequent in the Iberian Peninsula and clearly associated with herbaceous environments (Bosmans 2006). Other Linyphiidae, for example, *Diplocephalusgraecus* (O. Pickard-Cambridge, 1873), *Pelecopsisinedita* (O. Pickard-Cambridge, 1875) and *Tapinocybaalgirica* Bosmans, 2007 were present with clearly lower frequencies. The Theridiidae *Neottiurauncinata* (Lucas, 1846), whose webbing and hunting system differ from those used by the *A.pseudorurestris*, but which shares with it the usual space between the ground and the basal zone of herbaceous plants, was also very abundant. There were also other Theridiidae with much lower frequencies: *Theridionpinastri* L. Koch, 1872 and *Asagenaphalerata* (Panzer, 1801); although, in these two cases, with different strategies and positions in the ecosystem to *N.uncinata*. On the other hand, other species were represented which occupy more varied positions in the spatial structure of the grassland. This was the case of the Araneidae *Hypsosingaalbovittata* (Westring, 1851), the Oxyopidae *Oxyopesheterophthalmus* (Latreille, 1804), the Salticidae *Chalcoscirtusinfimus* (Simon, 1868), Euophrysherbigrada (Simon, 1871) and *Phlegrabresnieri* (Lucas, 1846) and the Cheiracanthiidae *Cheiracanthiumpennatum* Simon, 1878, all of which swarm or settle in the middle or upper part of the vegetation.

There were also some species that tend to move along the ground in search of their prey, taking refuge easily in the irregularities of the ground, at the base of plants or under plant remains. This is the case of another set of captured forms, such as the Thomisidae *Ozyptilapauxilla* (Simon, 1870) and *Xysticusnubilus* Simon, 1875, the Lycosidae *Pardosaproxima* C. L. Koch, 1847, the Clubionidae *Porrhoclubionavegeta* (Simon, 1918), the Philodromidae *Thanatusvulgaris* Simon, 1875 and *Pulchellodromuspulchellus* (Lucas, 1846), as well as several species of Gnaphosidae, *Nomisiaexornata* (C. L. Koch, 1839), *Setaphiscarmeli* (O. Pickard-Cambridge, 1872), *Haplodrassusrhodanicus* (Simon, 1914), *Leptodrassusalbidus* Simon, 1914 and *Civizelotesibericus* Senglet, 2012. The tiny Synaphridae, *Synaphrissaphrynis* Lopardo et al. 2007, an unusual species, unknown until recently and now appearing with considerable frequency on the soil of the Castro Verde grassland, deserves a special mention. In contrast, the Zodariidae *Zodarionjozefienae* Bosmans, 1994 only reaches very low numbers, despite being a myrmecophilous family that is usually more abundant in soil samples.

The number of Opiliones specimens was too low to identify general patterns since data suggest an even greater undersampling due to their nocturnal habits (taking refuge in hidden places during the day) as suggested by the very small number of specimens, less than 1% compared to spiders. Nearly all belonged to *Homalenotusbuchneri*, an edaphic species characteristic of open herbaceous environments.

With the exception of some occasional data in Silva (2004), the data we now offer represent the first faunal information for the SPA of Castro Verde. However, in comparison with the information available for Baixo Alentejo (Beja District), the list represents the addition of 19 species (17 spiders and two harvestmen), so that the provisional balance of diversity in this Portuguese region is 317 species of spiders (Barrientos et al. 2020) and six of harvestmen.

The methodological approach of the study was carried out in a specific period (sampling, lasting a few hours, in the months of April or May), in similar and relatively homogeneous plots, intentionally avoiding the proximity of the forest areas and their possible influence. Invertebrate sampling was undoubtedly aimed at assessing the feeding potential of the different plots for steppe birds. On the other hand, the methodological development relied exclusively on the use of a vortex specially designed for the sampling of invertebrates present in defined and standardisable areas.

One of the utilities of the approaches followed is the easy standardisation of the data (e.g. repetitive replicates, ratio of each sample to a surface unit). Thus, it is possible to convert the absolute frequencies, previously used, into relative frequencies (either for a given plot, a group of plots or for all of them). Additionally and in a parallel way, it is possible to relate the captures to the basic surface unit of each point (0.194 m^2^). Consequently, we can now use these new parameters to approximate population densities and their possible variations. However, this sampling method does not convey an important part of the information required for the biological knowledge of the species that occupy the habitat studied.

In the first place, it does not provide adequate phenological images; it is evident that the sampling period corresponds to one of the most outstanding stages in the annual cycle (Cardoso et al. 2007): the population hatchings, with the massive appearance of juveniles and the mixtures of adults in the reproductive phase. This situation is especially reflected in the frequency of the main species; in particular, for the three most abundant species in the captures: *Agynetapseudorrurestris*, *Neottiurauncinata* and *Hypsosingaalbovittata* (Fig. 3). This study lacks information on the preparatory periods of population hatching, summer resolution and potential autumn resurgence, which are important aspects to consider. The presence of permanent populations cannot be discounted and the dynamics of population fluctuations over time are unknown. Additionally, the existence of other species that may manifest at different times during the annual cycle is yet to be determined.

A second aspect that has been missed in the sampling method used is the stratification of the arachnological fauna. It is impossible to discern which species naturally occupy the epiphytic stratum exclusively, which species climb the grasses temporarily and which species mainly lay their webs and are found at different heights of the herbaceous framework. Future analyses should explore the relationship between vegetation height and land-use type in the frequency of species captured. Specifically, purely epiedaphic species are expected to occupy the lower herbaceous stratum, co-existing with diverse hunting strategies and forming a complex web of trophic relationships. Additionally, other species will predominantly inhabit the middle and upper strata of the grasses, leading to their absence in grazed plots and abundance in fallow areas.

Thirdly, there are no data on the nearby fauna, typical of the surrounding wooded areas (pine woods, holm oak, cork oak, eucalyptus etc.), as well as possible waterlogged areas. This prevents us from offering a contrast between different types of habitat and justifying the occasional and singletons (visiting or accidental species) in the steppe grassland habitat of Castro Verde.

### Conclusion

Arachnids in general and spiders in particular, constitute a part of biodiversity situated at one of the tips of the trophic chains. Arachnids are, therefore, one of the best indicators of the richness and stability of biodiversity in natural habitats (Cardoso et al. 2011a). Thus, precise knowledge of the identity of species and the details of their biology are key to the ecological interpretation of their role in ecosystems. The study carried out at Castro Verde, despite its shortcomings, extends the list of Araneae species known for Portugal (three species added) and Beja District (14 species added). They also allow us to record a new *Zelotes* species, whose provisional locality is limited to the Castro Verde SPA. On the other hand, the species richness recorded (71) increases the interest of the Castro Verde SPA in terms of biodiversity. The diversity of species observed is limited to the steppe-like habitat, dominant in Castro Verde, but excludes consideration of the heterogeneity of the landscape, especially the forest plantations, the study of which would undoubtedly increase the levels of biodiversity. The list of species would probably be more complete if other sampling methods were used at the same time, such as pitfall traps and beating trays and if the study were extended to the whole annual cycle. Overall, this study provides the first information on the Arachnid fauna of Castro Verde and adds new data for the southern part of Portugal; they could serve as a guideline for the management of biodiversity conservation in the SPA of Castro Verde.

## Supplementary Material

781F6EF4-22DC-530E-9C78-2EA57638A704 10.3897/BDJ.11.e110415.suppl1 Supplementary material 1 GBIF event dataset: Raw data on sampling sites and Arachnida specimens collected Data type events, occurrences and measurements in Darwin Core format (event.txt, occurrence.txt, extendedmeasurementorfact.txt tables; all in one zip file) File: oo_927237.zip https://binary.pensoft.net/file/927237 José A. Barrientos, Carlos E. Prieto, Sílvia Pina, Sérgio Henriques, Pedro Sousa, Stefan Schindler, Luís Reino, Pedro Beja, Joana Santana

A4076EA8-391F-533A-AFE0-82138D62ABC7 10.3897/BDJ.11.e110415.suppl2 Supplementary material 2 Samples studied arranged in alphabetical order of the taxa represented Data type occurrences (.pdf file) File: oo_927230.pdf https://binary.pensoft.net/file/927230 José A. Barrientos, Carlos E. Prieto, Sílvia Pina, Sérgio Henriques, Pedro Sousa, Stefan Schindler, Luís Reino, Pedro Beja, Joana Santana

## Figures and Tables

**Table 1.** Sampling parcels identification; farm toponym; municipality; Latitude and Longitude coordinates (decimal degrees); number of sampling points; mean vegetation height (cm); and sampling date.

**Parcel**	**Farm**	**Municipality**	**Latitude**	**Longitude**	**No points**	**Vegetation height**	**Sampling date**
P4	Herdade das Mouras	Castro Verde	37.7745	-8.0233	10	18	15/04/2012
P6	Herdade de A de Neves da Marinha	Castro Verde	37.7145	-8.0462	10	23	24/04/2012
P9	COSA - Herdade de São Marcos	Castro Verde	37.6985	-7.949	10	11	25/04/2012
P11	Monte do Broco e Capitão	Castro Verde	37.7873	-8.0118	10	8	15/04/2012
P14	Lagoa da Mó	Castro Verde	37.7579	-8.0964	10	9	18/04/2012
P15	Lagoa da Mó	Castro Verde	37.7484	-8.0899	10	17	12/04/2012
P19	Herdade do Torrejão	Castro Verde	37.7544	-8.0831	10	8	26/04/2012
P21	Herdade da Barrigoa	Castro Verde	37.7723	-8.1588	10	16	21/04/2012
P23	Monte do Vale das Gretas	Castro Verde	37.6893	-7.97	12	14	03/04/2012
P25	Herdade dos Touris e Rolão	Castro Verde	37.6836	-7.9512	12	12	05/04/2012
P27	Herdade das Sesmarias	Castro Verde	37.707	-8.0663	10	15	24/04/2012
P34	Herdade do Tacanho e Merendeiros	Castro Verde	37.7458	-8.0638	10	7	16/04/2012
P35	Monte do Seixo	Castro Verde	37.6433	-7.962	12	10	04-05/04/2012
P36	Herdade dos Bispos	Castro Verde	37.7397	-8.1345	10	12	11/04/2012
P37	Herdade de São Marcos	Castro Verde	37.6889	-7.9175	12	10	09/04/2012
P43	Monte da Comenda	Castro Verde	37.7676	-8.1859	10	14	21/04/2012
P45	Monte das Fontes Barbas Velho	Castro Verde	37.7732	-8.06	10	12	13/04/2012
P47	Herdade do Almarginho	Castro Verde	37.6823	-7.9299	12	12	09/04/2012
P49	Herdade da Navarra	Castro Verde	37.7119	-8.0733	10	39	25/04/2012
P50	Herdade do Torrejão	Castro Verde	37.7535	-8.0662	10	24	16/04/2012
P51	Herdade do Torrejão	Castro Verde	37.7657	-8.0664	10	6	13/04/2012
P53	Herdade do Reguengo	Castro Verde	37.7337	-8.1174	10	13	20/04/2012
P54	Herdade do Roncanho	Castro Verde	37.7004	-8.0345	10	7	24-25/04/2012
P55	Herdade da Barrigoa	Castro Verde	37.7705	-8.1512	10	18	21/04/2012
P56	Herdade da Barrigoa	Castro Verde	37.7836	-8.1427	10	17	17-18/04/2012
P57	Monte da Azinheira	Castro Verde	37.7655	-7.9464	12	8	06/04/2012
P58	Herdade da Benviúda	Mértola	37.7227	-7.8467	12	6	07/04/2012
P61	Monte da Torre	Aljustrel	37.8079	-8.1286	10	26	05/05/2012
P67	Monte da Chaiça	Aljustrel	37.8065	-8.1157	10	19	12/04/2012
P70	Monte dos Janeiros	Castro Verde	37.7566	-8.1648	10	19	21/04/2012
P72	Cumeada Nova	Castro Verde	37.7876	-8.0504	10	14	13-15/04/2012
P73	Herdade dos Longos	Castro Verde	37.7791	-8.1176	10	21	18-19/04/2012
P78	Herdade de São Marcos	Castro Verde	37.7001	-7.9294	12	7	09/04/2012
P79	Monte da Chada	Castro Verde	37.704	-7.9165	12	9	07/04/2012
P81	Herdade de Reidias	Castro Verde	37.7379	-8.0949	10	9	20/04/2012
P83	Herdade do Álamo	Castro Verde	37.7831	-8.0797	10	28	06/05/2012
P86	Herdade das Mestras	Castro Verde	37.771	-8.1331	10	23	10/05/2012
P87	Herdade das Mestras	Castro Verde	37.7653	-8.1218	10	26	06/05/2012
P88	Herdade da Portela	Castro Verde	37.7226	-8.0775	10	14	25/04/2012
P89	Herdade do Carapetal	Castro Verde	37.788	-8.0934	10	33	06/05/2012
P92	Monte do Carregueiro	Aljustrel	37.8177	-8.1161	10	8	17/04/2012
P93	Herdade da Sobreira	Aljustrel	37.8152	-8.1477	10	15	17/04/2012
P96	Herdade dos Bispos	Castro Verde	37.7312	-8.1481	10	10	11/04/2012
P97	Cumeada Nova	Castro Verde	37.7814	-8.0537	10	12	13/04/2012
P102	Monte da Fonte	Castro Verde	37.7558	-7.9918	10	8	15/04/2012
P104	Herdade dos Touris e Rolão	Castro Verde	37.6756	-7.9494	12	6	04/04/2012
P107	Herdade das Mestras	Castro Verde	37.7651	-8.1105	10	14	18/04/2012
P108	Herdade dos Merendeiros	Castro Verde	37.7285	-8.0073	10	13	10/04/2012
P109	Monte da Achada	Castro Verde	37.7017	-8.0491	10	23	24/04/2012
P110	Monte da Perdigoa	Castro Verde	37.7469	-8.052	10	28	04/05/2012
P111	Monte da Perdigoa	Castro Verde	37.7541	-8.0548	10	6	16/04/2012
P112	Monte do Freire	Castro Verde	37.7195	-8.0913	10	11	20/04/2012
P113	Courela do Monte Novo	Castro Verde	37.7481	-8.0061	10	13	10/04/2012
P114	Herdade das Cuchilhas	Castro Verde	37.7281	-7.8779	12	10	07-08/04/2012
P116	Herdade dos Merendeiros	Castro Verde	37.7374	-8.0074	10	8	10/04/2012
P121	Monte do Tacanho	Castro Verde	37.7236	-8.0623	10	27	16/04/2012
P122	Herdade da Chaiça Velha	Castro Verde	37.7873	-8.107	10	16	12/04/2012
P123	Herdade das Bicadas	Castro Verde	37.7236	-8.1258	10	28	20/04/2012
P124	Herdade das Mestras	Castro Verde	37.7774	-8.1352	10	18	18/04/2012
P125	Herdade da Sobreira	Aljustrel	37.8075	-8.1603	10	24	17/04/2012
P126	Monte do Seixo	Castro Verde	37.6535	-7.9583	12	4	05/04/2012
P127	Herdade dos Montinhos	Castro Verde	37.7429	-8.1124	10	18	11/04/2012
P128	Herdade de Carriça-Viseus	Castro Verde	37.659	-7.9499	12	8	04/04/2012
P129	Herdade da Zibreira	Castro Verde	37.7928	-8.159	10	25	07-08/05/2012
P131	Herdade do Torrejão	Castro Verde	37.7617	-8.0773	10	17	04/05/2012
P132	Monte das Cabeceiras	Castro Verde	37.7705	-8.0808	10	18	06/05/2012
P133	Monte da Albergaria	Castro Verde	37.7439	-7.9964	10	17	26-28/04/2012
PA46	Monte da Chaiça	Aljustrel	37.8029	-8.0934	10	21	09-10/05/2012
PA57	Herdade de Corta Rabos	Aljustrel	37.8007	-8.1355	10	23	08/05/2012
PA166	Herdade dos Brunhachos	Castro Verde	37.7214	-8.0995	10	15	07/05/2012
PA260	Herdade de Reidias	Castro Verde	37.73	-8.0934	10	11	07/05/2012
PA297	Lagoa da Mó	Castro Verde	37.7486	-8.1063	10	32	09/05/2012
PA299	Lagoa da Mó	Castro Verde	37.7556	-8.1046	10	26	08/05/2012
PA349	Herdade das Mestras	Castro Verde	37.7716	-8.122	10	23	10/05/2012
PA388	Monte da Achada	Castro Verde	37.7065	-8.0565	10	16	10/05/2012
PA423	Herdade da Zibreira	Castro Verde	37.7893	-8.1488	10	33	08/05/2012
PA482	Amendoeira Nova	Castro Verde	37.7046	-8.0392	10	8	09/05/2012
PA505	Herdade do Reguengo	Castro Verde	37.7366	-8.1082	10	36	08/05/2012
PA527	Herdade dos Bispos	Castro Verde	37.7498	-8.1221	10	25	07/05/2012
PA999	Herdade dos Pereiros	Castro Verde	37.7703	-8.0307	10	24	09/05/2012

**Table 2.** Arachnid fauna (Araneae and Opiliones) previously mentioned for the Beja District following: (a) de Biurrun et al. (2019); (b) Barrientos et al. (2020); (c) Rambla (1967); and (d) Rambla (1970) and for the Castro Verde Special Protection Area (this study, Barrientos et al. (2023)): NBd - New for Beja District; NPT - New for Portugal. The species collected in this study are highlighted in bold.

**Order / Family**	**Genera**	**Species**	**Notes**
**Araneae Clerck, 1757**			
Agelenidae C. L. Koch, 1837	*Agelena* Walckenaer, 1805	*A.labyrinthica* (Clerck, 1757)	(a)
	*Eratigena* Bolzern, Burckhardt & Hänggi, 2013	*E.atrica* (C. L. Koch, 1843)	(a); (b)
		*E.feminea* (Simon, 1870)	(a); (b)
		*E.montigena* (Simon, 1973)	(a)
		*E.picta* (Simon, 1870)	(a); (b)
	*Lycosoides* Lucas, 1846	*L.coarctata* (Dufour, 1831)	(a); (b)
	*Tegenaria* Latreille, 1804	*T.pagana* C. L. Koch, 1840	(a)
	*Textrix* Sundevall, 1833	*T.caudata* L. Koch, 1872	(a)
Araneidae Clerck, 1757	*Aculepeira* Chamberlin & Ivie, 1942	*A.armida* (Audouin, 1826)	(a)
	*Agalenatea* Archer, 1951	*A.redii* (Scopoli, 1763)	(a)
	*Araneus* Clerck, 1757	*A.diadematus* Clerck, 1757	(b)
	*Argiope* Audouin, 1826	*A.bruennichi* (Scopoli, 1772)	(a)
		*A.lobata* (Pallas, 1772)	(a)
		*A.trifasciata* (Forsskål, 1775)	(a)
	*Cyclosa* Menge, 1866	*C.algerica* Simon, 1885	(a)
		*C.insulana* (Costa, 1834)	(a)
	*Cyrtophora* Simon, 1864	*C.citricola* (Forsskål, 1775)	(a); (b)
	*Gibbaranea* Archer, 1951	*G.bruuni* Lissner, 2016	(a)
	*Hypsosinga* Ausserer, 1871	***H.albovittata* (Westring, 1851)**	**(a); this study**
	*Larinia* Simon, 1874	*L.lineata* (Lucas, 1846)	(b)
	*Larinioides* Caporiacco, 1934	*L.patagiatus* (Clerck, 1757)	(a)
		*L.sclopetarius* (Clerck, 1757)	(a)
	*Leviellus* Wunderlich, 2004	*L.kochi* (Thorell, 1870)	(a)
	Mangora O. Pickard-Cambridge, 1889	***M.acalypha* (Walckenaer, 1802)**	**(a); (b); this study**
	Neoscona Simon, 1864	*N.adianta* (Walckenaer, 1802)	(a)
		*N.subfusca* (C. L. Koch, 1837)	(a); (b)
	Nuctenea Simon, 1864	*N.umbratica* (Clerck, 1757)	(a)
	*Singa* C. L. Koch, 1836	*S.neta* (O. Pickard-Cambridge, 1872)	(a)
Cheiracanthiidae Wagner,1887	*Cheiracanthium* C. L. Koch, 1839	*C.pelasgicum* (C. L. Koch, 1837)	(a)
		***C.pennatum* Simon, 1878**	**This study; NBd**
		*C.striolatum* Simon, 1878	(a)
Clubionidae Simon, 1878	*Clubiona* Latreille, 1804	*C.comta* C. L. Koch, 1839	(a)
	*Porrhoclubiona* Lohmander, 1944	*P.genevensis (L. Koch, 1866)	(a)
		*P.leucaspis (Simon, 1932)	(a)
		*P.vegeta (Simon, 1918)	**(a); (b); this study**
Corinnidae Karsch, 1880	*Castianeira* Keyserling, 1879	*C.badia* (Simon, 1877)	(a)
Dictynidae O. Pickard-Cambridge, 1871	*Archaeodictyna* Caporiacco, 1928	*A.consecuta* (O. Pickard-Cambridge, 1872)	(a); (b)
	*Argenna* Simon, 1884	***A.subnigra* (O. Pickard-Cambridge, 1861)**	**This study; NPT**
	*Brigittea* Lehtinen, 1967	*B.civica* (Lucas, 1850)	(a)
	*Marilynia* Lehtinen, 1967	***M.bicolor* (Simon, 1870)**	**(a); (b); this study**
	*Nigma* Lehtinen, 1967	***N.puella* (Simon, 1870)**	**(a); (b); this study**
	*Scotolathys* Simon, 1884	*S.simplex* Simon, 1884	(a)
Dysderidae C. L. Koch, 1837	*Dysdera* Latreille, 1804	*D.alentejana* Ferrández, 1996	(a); (b)
		*D.fuscipes* Simon, 1882	(a)
		*D.gamarrae* Ferrández, 1984	(a)
	*Harpactea* Bristowe, 1939	***H.minoccii* Ferrández, 1982**	**(a); this study**
		*H.proxima* Ferrández, 1990	(a)
		*H.subiasi* Ferrández, 1990	(a)
Eresidae C. L. Koch, 1845	*Eresus* Walckenaer, 1805	*E.kollari* Rossi, 1846	(a)
Filistatidae Ausserer, 1867	*Filistata* Latreille, 1810	*F.insidiatrix* (Forsskål, 1775)	(a); (b)
	*Pritha* Lehtinen, 1967	*P.pallida* (Kulczynski, 1897)	(a)
Gnaphosidae Banks,1892	*Callilepis* Westring, 1874	*C.concolor* Simon, 1914	(a); (b)
	*Civizelotes* Senglet, 2012	*C.caucasius* (L. Koch, 1866)	(a); (b)
		***C.civicus* (Simon, 1878)**	**(a); this study**
		*C.dentatidens* (Simon, 1914)	(a)
		***C.ibericus* Senglet, 2012**	**This study; NPT**
		*C.medianus* (Denis, 1936)	(a)
	*Drassodes* Westring, 1851	*D.lapidosus* (Walckenaer, 1802)	(a); (b)
		*D.luteomicans* (Simon, 1878)	(a)
		*D.pubescens* (Thorell, 1856)	(a)
		*D.rubidus* (Simon, 1878)	(a)
	*Gnaphosa* Latreille, 1804	*G.alacris* Simon, 1878	(a); (b)
		*G.lucifuga* (Walckenaer, 1802)	(b)
	*Haplodrassus* Chamberlin, 1922	*H.dalmatensis* (L. Koch, 1866)	(a)
		*H.ibericus* Melic, Silva, Barrientos, 2016	(a); (b)
		*H.macellinus* (Thorell, 1871)	(a)
		*H.minor* (O. Pickard-Cambridge, 1879)	(a)
		***H.rhodanicus* (Simon, 1914)**	**This study; NBd**
		*H.rufipes* (Lucas, 1846)	(a)
		*H.signifer* (C. L. Koch, 1839)	(a); (b)
	*Leptodrassex* Murphy, 2007	*L.simoni* (Dalmas, 1919)	(a)
	*Leptodrassus* Simon, 1878	***L.albidus* Simon, 1914**	**(a); (b); this study**
		*L.femineus* (Simon, 1873)	(a)
	*Marinarozelotes* Ponomarev, 2020	*M.bardiae* (Caporiacco, 1928)	(a)
		*M.fuscipes* (L. Koch, 1866)	(a)
		*M.holosericeus* (Simon, 1878)	(a); (b)
		***M.minutus* (Crespo, 2010)**	**This study; NBd**
		*M.mutabilis* (Simon, 1878)	(a)
	*Micaria* Westring, 1851	*M.coarctata* (Lucas, 1846)	(a)
		*M.dives* (Lucas, 1846)	(a)
		*M.formicaria* (Sundevall, 1831)	(a); (b)
		*M.pallipes* (Lucas, 1846)	(b)
		*M.triguttata* Simon, 1884	(a)
	*Nomisia* Dalmas, 1921	*N.excerpta* (O. Pickard-Cambridge, 1872)	(a); (b)
		***N.exornata* (C. L. Koch, 1839)**	**(b); this study**
	*Poecilochroa* Westring, 1874	*P.senilis* (O. Pickard-Cambridge, 1872)	(a); (b)
	*Prodidomus* Hentz, 1847	*P.amaranthinus* (Lucas, 1846)	(a)
	*Pterotricha* Kulczyński, 1903	*P.chazaliae* (Simon, 1895)	(a)
		*P.lesserti* Dalmas, 1921	(a)
		*P.simoni* Dalmas, 1921	(a)
	*Scotophaeus* Simon, 1893	*S.blackwalli* (Thorell, 1871)	(a)
		*S.dolanskyi* Lissner, 2017	(a)
		*S.scutulatus* (L. Koch, 1866)	(b)
		*S.validus* (Lucas, 1846)	(a)
	*Setaphis* Simon, 1893	***S.carmeli* (O. Pickard-Cambridge, 1872)**	**(a); (b); this study**
		*S.parvula* (Lucas, 1846)	(a)
	*Zelominor* Snazell & Murphy, 1997	*Z.algarvensis* Snazell, Murphy, 1997	(a)
	*Zelotes* Gistel, 1848	*Z.aeneus* (Simon, 1878)	(a)
		*Z.callidus* (Simon, 1878)	(a); (b)
		*Z.cornipalpus* Melic, Silva & Barrientos, 2016	(a); (b)
		*Z.criniger* Denis, 1937	(a)
		*Z.egregioides* Senglet, 2011	(b)
		*Z.flagellans* (L. Koch, 1882)	(a); (b)
		***Z.fulvopilosus* (Simon, 1878)**	**(a); (b); this study**
		*Z.gallicus* Simon, 1914	(a)
		*Z.laetus* (O. Pickard-Cambridge, 1872)	(a)
		*Z.lagrecai* Di Franco, 1994	(a)
		*Z.longipes* (L. Koch, 1866)	(a)
		*Z.manius* (Simon, 1878)	(a)
		*Z.pediculatus* Marinaro, 1967	(b)
		*Z.segrex* (Simon, 1878)	(a); (b)
		*Z.spadix* (L. Koch, 1866)	(b)
		*Z.tenuis* (L. Koch, 1866)	(a); (b)
		*Z.thorelli* Simon, 1914	(a); (b)
		*Z.wallacei* Melic, Silva & Barrientos, 2016	(a); (b)
Hahniidae Bertkau, 1878	*Hahnia* C. L. Koch, 1841	*H.nava* (Blackwall, 1841)	(a)
Halonoproctidae Pocock, 1901	*Ummidia* Thorell, 1875	*U.algarve* Decae, 2010	(a)
HersiliidaeThorell, 1870	*Hersiliola* Thorell, 1869	*H.macullulata* (Dufour, 1831)	(a)
LeptonetidaeSimon, 1890	*Teloleptoneta* Ribera, 1988	*T.synthetica* (Machado, 1951)	(a)
Linyphiidae Blackwall, 1859	*Agyneta* Hull, 1911	*A.fuscipalpa* (C. L. Koch, 1836)	(a)
		***A.pseudorurestris* Wunderlich, 1980**	**(a); (b); this study**
	*Araeoncus* Simon, 1884	*A.humilis* (Blackwall, 1841)	(a)
	*Canariphantes* Wunderlich, 1992	*C.zonatus* (Simon, 1884)	(a)
	*Centromerus* Dahl, 1886	*C.minutissimus* Merrett & Powell, 1993	(b)
		***C.phoceorum* Simon, 1929**	**(a); this study**
		*C.prudens* (O. Pickard-Cambridge, 1873)	(a)
		*C.succinus* (Simon, 1884)	(a)
	*Didectoprocnemis* Denis, 1950	*D.cirtensis* (Simon, 1884)	(a)
	*Diplocephalus* Bertkau, 1883	***D.graecus* (O. Pickard-Cambridge, 1873)**	**(a); (b); this study**
		***D.marijae* Bosmans, 2010**	**(a); this study**
	*Erigone* Audouin, 1826	***E.dentipalpis* (Wider, 1834)**	**(a); this study**
	*Erigonoplus* Simon, 1884	*E.depressifrons* (Simon, 1884)	(a)
	*Lessertia* Smith, 1908	*L.dentichelis* (Simon, 1884)	(a)
	*Microctenonyx* Dahl, 1886	***M.subitaneus* (O. Pickard-Cambridge, 1875)**	**(a); (b); this study**
	*Microlinyphia* Gerhardt, 1928	*M.pusilla* (Sundevall, 1830)	(a); (b)
	*Oedothorax* Bertkau, 1883	***Oedothoraxfuscus* (Blackwall, 1834)**	**(a); (b); this study**
	*Ouedia* Bosmans & Abrous, 1992	***O.rufithorax* (Simon, 1881)**	**(a); (b); this study**
	*Palliduphantes* Saaristo & Tanasevitch, 2001	*P.bolivari* (Fage, 1931)	(a)
		***P.stygius* (Simon, 1884)**	**(a); this study**
	*Pelecopsis* Simon, 1864	***P.bucephala* (O. Pickard-Cambridge, 1875)**	**(a); (b); this study**
		***P.inedita* (O. Pickard-Cambridge, 1875)**	**(a); (b); this study**
		*P.susannae* (Simon, 1915)	(a)
	*Prinerigone* Millidge, 1988	***P.vagans* (Audouin, 1826)**	**(a); (b); this study**
	*Sintula* Simon, 1884	*S.furcifer* (Simon, 1911)	(a)
	*Styloctetor* Simon, 1884	***S.romanus* (O. Pickard-Cambridge, 1873)**	**(a); (b); this study**
	*Tapinocyba* Simon, 1884	***T.algirica* Bosmans, 2007**	**This study; NBd**
	*Tenuiphantes* Saaristo & Tanasevitch, 1996	*T.tenuis* (Blackwall, 1852)	(b)
	*Trichopterna* Kulczyński, 1894	*T.cucurbitina* (Simon, 1881)	(a)
	*Walckenaeria* Blackwall, 1833	*W.corniculans* (O. Pickard-Cambridge, 1875)	(a)
		***W.cucullata* (C. L. Koch, 1836)**	**This study; NPT**
		*W.dalmasi* (Simon, 1915)	(a)
Liocranidae Simon,1897	*Agraecina* Simon, 1932	***A.lineata* Simon, 1878)**	**This study; NBd**
	*Agroeca* Westring, 1861	*A.annulipes* Simon, 1878	(b)
	*Liocranum* L. Koch, 1866	*L.majus* Simon, 1878	(a)
	*Mesiotelus* Simon, 1897	*M.mauritanicus* Simon, 1909	(a)
		*M.tenuissimus* (L. Koch, 1866)	(a); (b)
	*Scotina* Menge, 1873	*S.celans* (Blackwall, 1841)	(a)
Lycosidae Sundevall,1833	*Alopecosa* Simon, 1885	*A.accentuata* (Latreille, 1817)	(a)
		***A.albofasciata* (Brullé, 1832)**	**(a); (b); this study**
		*A.simoni* (Thorell, 1872)	(b)
	*Arctosa* C. L. Koch, 1847	*A.cinerea* (Fabricius 1777)	(a)
		*A.lacustris* (Simon, 1876)	(a)
	*Hogna* Simon, 1885	***H.radiata* (Latreille, 1817)**	**(a); (b); this study**
	*Lycosa* Latreille, 1804	*L.hispanica* (Walckenaer, 1837)	(a)
	*Pardosa* C. L. Koch, 1847	*P.hortensis* (Thorell, 187)	(a)
		***P.proxima* (C. L. Koch, 1847)**	**(a); (b); this study**
	*Pirata* Sundevall, 1833	*P.tenuitarsis* Simon, 1876	(a)
	*Trochosa* C. L. Koch, 1847	*T.hispanica* Simon, 1870	(a)
		*T.ruricola* (De Geer, 1778)	(a)
	*Wadicosa* Zyuzin, 1985	*W.fidelis* (O. Pickard-Cambridge, 1972)	(a)
Mimetidae Simon, 1881	*Ero* C. L. Koch, 1836	***E.aphana* (Walckenaer, 1802)**	**(a); (b); this study**
	*Mimetus* Hentz, 1832	*M.laevigatus* (Keyserling, 1863).	(a)
Miturgidae Simon, 1886	*Zora* C. L. Koch, 1847	*Z.manicata* Simon, 1878	(a)
		***Z.silvestris* Kulczyński, 1897**	**(a); this study**
		*Z.spinimana* (Sundevall, 1833)	(a)
Nemesiidae Simon,1889	*Iberesia* Decae & Cardoso, 2006	*I.machadoi* Decae, Cardoso, 2006	(a); (b)
	*Nemesia* Audouin, 1826	*N.athiasi* Franganillo, 1920	(a)
		*N.fagei* Frade & Bacelar, 1931	(a)
		*N.uncinata* Bacelar, 1933	(a); (b)
Oecobiidae Blackwall, 1862	*Oecobius* Lucas, 1846	*O.machadoi* Wunderlich, 1995	(a); (b)
		*O.maculatus* Simon, 1870	(a)
	*Uroctea* Dufour, 1820	*U.durandi* (Latreille, 1809)	(b)
Oonopidae Simon, 1890	*Oonops* Templeton, 1835	***O.tubulatus* Dalmas, 1916**	**(a); this study**
		*O.tubulatus* Dalmas, 1916	(a)
	*Silhouettella* Benoit, 1979	***S.loricatula* (Roewer, 1942)**	**(a); this study**
Oxyopidae Thorell,1869	*Oxyopes* Latreille, 1806	***O.heterophthalmus* (Latreille, 1804)**	**(a); this study**
		*O.lineatus* Latreille, 1806	(a); (b)
		*O.mediterraneus* Levy, 1999	(b)
		*O.nigripalpis* Kulczynski, 1891	(a); (b)
Palpimanidae Thorell, 1870	*Palpimanus* Dufour, 1820	*P.gibbulus* Dufour, 1820	(a); (b)
Philodromidae Thorell, 1869	*Philodromus* Walckenaer, 1826	*P.albidus* Kulczynski, 1911	(b)
		*P.aureolus* (Clerck, 1757)	(a)
		*P.buxi* Simon, 1884	(a)
		*P.cespitum* (Walckenaer, 1802)	(a)
		*P.praedatus* O. Pickard-Cambridge, 1871	(b)
		*P.rufus* Walckenaer, 1826	(a)
	*Pulchellodromus* Wunderlich, 2012	*P.glaucinus* (Simon, 1870)	(a)
		***P.pulchellus* (Lucas, 1846)**	**(a); this study**
		*P.ruficapillus* (Simon, 1885)	(b)
	*Thanatus* C. L. Koch, 1837	*T.atratus* Simon, 1875	(b)
		*T.fabricii* (Audouin, 1826)	(b)
		*T.lineatipes* Simon, 1870	(a)
		***T.vulgaris* Simon, 1870**	**(a); (b); this study**
	*Tibellus* Simon, 1875	***T.macellus* Simon, 1875**	**This study; NBd**
PholcidaeC. L. Koch, 1850	*Holocnemus* Simon, 1873	*H.hispanicus* Wihle, 1933	(a)
		*H.pluchei* (Scopoli, 1763)	(a)
	*Pholcus* Walckenaer, 1805	*P.opilionoides* (Schrank, 1781)	(a)
		*P.phalangioides* (Fuesslin, 1775)	(a)
Phrurolithidae Banks, 1892	*Liophrurillus* Wunderlich, 1992	*L.flavitarsis* (Lucas, 1846)	(a); (b)
Pisauridae Simon, 1890	*Pisaura* Simon, 1886	***Pi.mirabilis* (Clerck, 1757)**	**(a); (b); this study**
Salticidae Blackwall, 1841	*Aelurillus* Simon, 1885	*A.luctuosus* (Lucas, 1846)	(a); (b)
	*Ballus* C. L. Koch, 1850	*B.chalybeius* (Walckenaer, 1802)	(a); (b)
	*Bianor* G. W. Peckham & E. G. Peckham, 1886	*B.albobimaculatus* (Lucas, 1846)	(a)
	*Chalcoscirtus* Bertkau, 1880	***C.infimus* (Simon, 1868)**	**(a); (b); this study**
	*Cyrba* Simon, 1876	*C.algerina* (Lucas, 1846)	(a); (b)
	*Euophrys* C. L. Koch, 1834	*E.frontalis* (Walckenaer, 1802)	(a); (b)
		*E.gambosa* (Simon, 1868)	(a); (b)
		***E.herbigrada* (Simon, 1871)**	**(a); this study**
		*E.nigripalpis* Simon, 1937	(a)
		*E.rufibarbis* (Simon, 1868)	(a)
		*E.sulphurea* (L. Koch, 1867)	(a)
	*Evarcha* Simon, 1902	*E.jucunda* (Lucas, 1846)	(a); (b)
	*Heliophanus* C. L. Koch, 1833	*H.agricola* Wesolowska, 1986	(a)
		*H.cupreus* (Walckenaer, 1802)	(a)
		*H.haymozi* Logunov, 2015	(a)
		***H.lineiventris* Simon, 1868**	**(a); this study**
		*H.melinus* L. Koch, 1867	(b)
	*Icius* Simon, 1876	*I.hamatus* (C. L. Koch, 1846)	(a); (b)
	*Leptorchestes* Thorell, 1870	*L.mutilloides* (Lucas, 1846)	(a)
		*L.peresi* (Simon, 1868)	(a)
	*Macaroeris* Wunderlich, 1992	*M.moebi* (Bösenberg, 1895)	(a)
	*Menemerus* Simon, 1868	*M.semilimbatus* (Hahn, 1829)	(a)
		*M.taeniatus* (L. Koch, 1867)	(b)
	*Neaetha* Simon, 1885	*N.membrosa* (Simon, 1868)	(a); (b)
	*Pellenes* Simon, 1876	*P.arciger* (Walckenaer, 1837)	(b)
		***P.nigrociliatus* (Simon, 1875)**	**(a); this study**
	*Phlegra* Simon, 1876	***P.bresnieri* (Lucas, 1846)**	**(a); (b); this study**
		*P.fasciata* (Hahn, 1826)	(a)
		*P.sierrana* (Simon, 1868)	(a); (b)
	*Pseudeuophrys* Dahl, 1912	*P.erratica* (Walckenaer, 1826)	(a)
		*P.squamifer* (Simon, 1881)	(a)
	*Salticus* Latreille, 1804	*S.confusus* Lucas, 1846	(a)
		***S.propinquus* Lucas, 1846**	**(a); this study**
		*S.scenicus* (Clerck, 1757)	(a); (b)
	*Talavera* G. W. Peckham & E. G. Peckham, 1909	***T.petrensis* (C. L. Koch, 1837)**	**This study; NBd**
	*Thyene* Simon, 1885	*T.imperialis* (Rossi, 1846).	(a)
Scytodidae Blackwall, 1864	*Scytodes* Latreille, 1804	*S.velutina* Heineken & Lowe, 1832	(a); (b)
Segestriidae Simon, 1893	*Ariadna* Audouin, 1826	*A.insidiatrix* Audouin, 1826	(a)
	*Segestria* Latreille, 1804	*S.florentina* (Rossi, 1790)	(b)
Sicariidae Keyserling, 1880	*Loxosceles* Heineken & Lowe, 1832	*L.rufescens* (Dufour, 1820)	(a); (b)
Sparassidae Bertkau, 1872	*Eusparassus* Simon, 1903	*E.dofouri* Simon, 1932	(a)
	*Micrommata* Latreille, 1804	M. ligurina (C. L. Koch, 1845)	(a)
	*Olios* Walckenaer, 1837	*O.argelasius* (Walckenaer, 1806)	(a)
Synaphridae Wunderlich, 1986	*Synaphris* Simon, 1894	***S.saphrynis* Lopardo, Hormiga & Melic, 2007**	**(b); this study**
Tetragnathidae Menge, 1866	*Pachygnatha* Sundevall, 1823	*P.tullgreni* Senglet, 1973	(a)
	*Tetragnatha* Latreille, 1804	*T.extensa* (Linnaeus, 1758)	(a)
		***T.intermedia* Kulczyński, 1891**	**This study; NBd**
		*T.obtusa* C. L. Koch, 1837	(b)
Theridiidae Sundevall, 1833	*Argyrodes* Simon, 1864	*A.argyrodes* (Walckenaer, 1841)	(a)
	*Asagena* Sundevall, 1833	***A.phalerata* (Panzer, 1801)**	**(a); (b); this study**
	*Dipoena* Thorell, 1869	***D.umbratilis* (Simon, 1873)**	**This study; NBd**
	*Enoplognatha* Pavesi, 1880	***E.diversa* (Blackwall, 1859)**	**This study; NBd**
		*E.franzi* Wunderlich, 1995	(b)
		*E.quadripunctata* Simon, 1884	(b)
		*E.thoracica* (Hahn, 1833)	(a)
	*Episinus* Walckenaer, 1809	*E.maculipes* Cavanna, 1876	(a)
		*E.truncatus* Latreille, 1809	(a)
	*Euryopis* Menge, 1868	***E.episinoides* (Walckenaer, 1847)**	**(a); (b); this study**
		*E.quinqueguttata* Thorell, 1875	(b)
	*Kochiura* Archer, 1950	*K.aulica* (C. L. Koch, 1838)	(a); (b)
	*Lasaeola* Simon, 1881	*L.convexa* (Blackwall, 1870)	(a)
		*L.testaceomarginata* Simon, 1881	(a)
	*Latrodectus* Walckenaer, 1805	*L.tredecimguttatus* (Rossi, 1790)	(a)
	*Neottiura* Menge, 1868	*N.curvimana* (Simon, 1914)	(a)
		***N.uncinata* (Lucas, 1846)**	**This study; NBd**
	*Paidiscura* Archer, 1950	***P.pallens* (Blackwall, 1834)**	**(b); this study**
	*Phylloneta* Archer, 1950	***P.impressa* (L. Koch, 1881)**	**(a); (b); this study**
	*Platnickina* Koçak & Kemal, 2008	*P.nigropunctata* (Lucas, 1846)	(a); (b)
	*Robertus* O. Pickard-Cambridge, 1879	*R.arundineti* (O. Pickard-Cambridge, 1871)	(a)
	*Ruborridion* Wunderlich, 2011	***R.musivum* (Simon, 1873)**	**This study; NBd**
	*Simitidion* Wunderlich, 1992	***S.simile* (C. L. Koch, 1836)**	**(a); (b); this study**
	*Steatoda* Sundevall, 1833	***S.albomaculata* (De Geer, 1778)**	**(a); this study**
		*S.nobilis* (Thorell, 1875)	(b)
		*S.triangulosa* (Walckenaer, 1802)	(a)
	*Theridion* Walckenaer, 1805	*T.hannoniae* Denis, 1945	(b)
		*T.hemerobium* Simon, 1914	(a)
		*T.mystaceum* L. Koch, 1870	(a); (b)
		***T.pinastri* L. Koch, 1872**	**This study; NBd**
		*T.varians* Hahn, 1833	(b)
Thomisidae Sundevall, 1833	*Bassaniodes* Pocock, 1903	***B.bliteus* (Simon, 1875)**	**(a); (b); this study**
		*B.bufo* (Dufour, 1820)	(a); (b)
		*B.cribratus* (Simon, 1885)	(a)
		*B.robustus* (Hahn, 1832)	(b)
	*Misumena* Latreille, 1804	*M.vatia* (Clerck, 1757)	(a)
	*Monaeses* Thorell, 1869	*M.paradosus* (Lucas, 1846)	(a)
	*Ozyptila* Simon, 1864	***O.pauxilla* (Simon, 1870)**	**(a); (b); this study**
		*O.simplex* (O. Pickard-Cambridge, 1862)	(a)
	*Runcinia* Simon, 1875	***R.grammica* (C. L. Koch, 1837)**	**(a); (b); this study**
	*Synema* Simon, 1864	*S.globosum* (Fabricius, 1775)	(a); (b)
	*Thomisus* Walckenaer, 1805	***T.onustus* Walckenaer, 1805**	**(a); (b); this study**
	*Tmarus* Simon, 1875	*T.staintoni* (O. Pickard-Cambridge, 1873)	(a); (b)
	*Xysticus* C. L. Koch, 1835	*X.cor* Canestrini, 1873	(b)
		*X.cristatus* (Clerck, 1757)	(a)
		*X.ferrugineus* Menge, 1876	(a)
		***X.grallator* Simon, 1932**	**This study; NBd**
		***X.nubilus* Simon, 1875**	**(a); this study**
Titanoecidae Lehtinen, 1967	*Nurscia* Simon, 1874	*N.albomaculata* (Lucas, 1846)	(b)
		*N.sequerai* (Simon, 1893)	(a)
	*Titanoeca* Thorell, 1870	*T.praefica* Simon, 1870	(b)
		*T.quadriguttata* (Hahn, 1833)	(a)
Trachelidae Simon, 1897	*Metatrachelas* Bosselaers & Bosmans, 2010	*M.rayi* (Simon, 1878)	(a)
Uloboridae Thorell, 1869	*Uloborus* Latreille, 1806	*U.walckenaerius* Latreille, 1806	(b)
Zodariidae Thorell, 1881	*Amphiledorus* Jocqué & Bosmans, 2001	*A.adonis* Jocqué & Bosmans, 2001	(a)
		*A.ungoliantae* Pekár & Cardoso, 2005	(a)
	*Selamia* Simon, 1873	*S.reticulata* (Simon, 1870)	(a); (b)
	*Zodarion* Walckenaer, 1826	*Z.alacre* (Simon, 1870)	(a); (b)
		*Z.alentejanum* Pekár & Carvalho, 2011	(a)
		*Z.bosmansi* Pekar, Cardoso, 2015	(a)
		***Z.jozefienae* Bosmans, 1994**	**(a); (b); this study**
		*Z.merlijni* Bosmans, 1994	(a)
		*Z.styliferum* (Simon, 1870)	(a)
Zoropsidae Bertkau, 1882	*Zoropsis* Simon, 1878	*Z.spinimana* (Dufour, 1820)	(a)
**Opiliones Sundevall, 1833**			
Phalangiidae Latreille, 1802	*Dasylobus* Simon, 1878	***D.ibericus* (Rambla, 1967)**	**This study; NBd**
	*Odiellus* Roewer, 1923	*O.troguloides* (Lucas, 1846)	(c); (d)
Phalangodidae Simon, 1879	*Scotolemon* Lucas, 1860	**S.aff.lespesii Lucas, 1860	(c); (d)
Sclerosomatidae Simon, 1879	*Cosmobunus* Simon, 1879	*C.granarius* (Lucas, 1846)	(c); (d)
	*Gyas* Simon, 1879	*G.titanus* Simon, 1879	(c); (d)
	*Homalenotus* Koch, 1839	***H.buchneri* (Schenkel, 1936)**	**This study; NBd**

* This species belongs to the *genevensis* group; the species of this group have a great morphological affinity, which makes a revision of the samples studied by Cardoso (2004) in the Parque Natural do Vale do Guadiana desirable.

** Rambla (1967) identified the species as *S.lespessi*; however, it is a Pyrenean species and, as such, the identification needs confirmation.
