# Supplementary material for: Arachnid Fauna (Araneae and Opiliones) from the Castro Verde Special Protection Area, southern Portugal
Source: Biodivers Data J. 2023 Dec 6;11:e110415. doi: 10.3897/BDJ.11.e110415 (PMC10719938; doi:10.3897/BDJ.11.e110415)
Supplement: Supplementary material 2 — Samples studied arranged in alphabetical order of the taxa represented [file bdj-11-e110415-s002.pdf]

Supplementary materials for

Arachnid Fauna (Araneae and Opiliones) from the Castro Verde Special Protection Area, Southern Portugal

José A. Barrientos[1], Carlos E. Prieto[2], Sílvia Pina[3,4], Sérgio Henriques[3,4], Pedro Sousa[3], Stefan Schindler[3,4,5], Luís Reino[3,4,6], Pedro Beja[3,4,6], Joana Santana[3,4,6]

1 c/ Balmes, 181, 3º, 2ª. 08006, Barcelona, España

2 Departamento de Zoología y Biología Celular Animal, Facultad de Ciencia y Tecnología, Universidad del País Vasco (UPV/EHU). Apdo. 644, 48080 Bilbao, España

3 CIBIO, Centro de Investigação em Biodiversidade e Recursos Genéticos, InBIO Laboratório Associado, Campus de Vairão, Universidade do Porto, 4485-661 Vairão, Portugal

4 CIBIO, Centro de Investigação em Biodiversidade e Recursos Genéticos, InBIO Laboratório Associado, Instituto Superior de Agronomia, Universidade de Lisboa, Tapada da Ajuda, 1349-017 Lisboa, Portugal

5 Community Ecology and Conservation, Faculty of Environmental Sciences, Community Ecology and Conservation Research Group, Kamýcká 129, CZ-165 00 Prague 6, Czech Republic

6 BIOPOLIS Program in Genomics, Biodiversity and Land Planning, CIBIO, Campus de Vairão, 4485-661 Vairão, Portugal

Corresponding Authors: Joana Santana (joanafsantana@cibio.up.pt); José A. Barrientos (joseantonio.barrientos@uab.es)

Supplementary material 2. Samples studied arranged in alphabetical order of the taxa represented.

<u>Order ARANEAE</u>

Family AGELENIDAE C. L. Koch, 1837

***Eratigena* sp.**

<u>Material examined</u>: Ref. **114**-3-h, 1 j., 08/04/2012. Castro Verde (Herdade das Cuchilhas).

Family ANYPHAENIDAE Bertkau, 1878

***Anyphaena* sp.**

<u>Material examined</u>: Ref. **108**-3-l, 1 j, 10/04/2012, Castro Verde (Herdade dos Merendeiros). Ref. **110**-7-f, 1 j, 04/05/2012, Castro Verde (Monte da Perdigoa).

Family ARANEIDAE Clerck, 1757

Araneidae sp.; (480 jj. indet. *Argiope*, *Neoscona*).

***Araniella* sp.**

<u>Material examined</u>: Ref. **23**-8-c, 1 j, 03/04/2012, Castro Verde (Monte do Vale das Gretas). Ref. **36**-6-h, 1 j, 11/04/2012, Castro Verde (Herdade dos Bispos). Ref. **50**-9-f, 1 j, 16/04/2012, Castro Verde (Herdade do Torrejão). Ref. **96**-2-e, 1 j,

11/04/2012, Castro Verde (Herdade dos Bispos). Ref. **124**-6-j, 1 j, 18/04/2012, Castro Verde (Herdade das Mestras). Ref. **125**-9-h, 1 j, 17/04/2012, Aljustrel (Herdade da Sobreira). Ref. **126**-11-b, 1 j, 05/04/2012, Castro Verde (Monte do Seixo). Ref. **128**-4-b, 1 j, 04/04/2012, Castro Verde (Herdade de Carriça-Viseus). Ref. **A423**-8-b, 1 j, 08/05/2012, Castro Verde (Herdade da Zibreira).

***Hypsosinga albovittata* (Westring, 1851)**

Material examined: Ref. **4**-4-g, 1j, 15/04/2012, Castro Verde (Heredade das Mouras). Ref. **4**-5-j, 1j, 15/04/2012, Castro Verde (Heredade das Mouras). Ref. **4**-11-b, 1j, 15/04/2012, Castro Verde (Heredade das Mouras). Ref. **6**-2-a, 1♀ 1j, 24/04/2012, Castro Verde (Herdade de A de Neves da Marinha). Ref. **6**-7-f, 1♀ 1j, 24/04/2012, Castro Verde (Herdade de A de Neves da Marinha). Ref. **6**-8-h, 1j, 24/04/2012, Castro Verde (Herdade de A de Neves da Marinha). Ref. **9**-2-g, 1j, 25/04/2012, Castro Verde (COSA - Herdade de São Marcos). Ref. **11**-9-d, 1j, 15/04/2012, Castro Verde (Monte do Broco e Capitão). Ref. **11**-11-e, 1j, 15/04/2012, Castro Verde (Monte do Broco e Capitão). Ref. **14**-7-e, 1j, 18/04/2012, Castro Verde (Lagoa da Mó). Ref. **14**-11-c, 1j, 18/04/2012, Castro Verde (Lagoa da Mó). Ref. **15**-2-h, 3jj, 12/04/2012, Castro Verde (Lagoa da Mó). Ref. **15**-3-b, 2jj, 12/04/2012, Castro Verde (Lagoa da Mó). Ref. **15**-6-a, 1♀ 2jj, 12/04/2012, Castro Verde (Lagoa da Mó). Ref. **15**-7-e, 1j, 12/04/2012, Castro Verde (Lagoa da Mó). Ref. **15**-9-a, 2jj, 12/04/2012, Castro Verde (Lagoa da Mó). Ref. **15**-11-c, 1j, 12/04/2012, Castro Verde (Lagoa da Mó). Ref. **19**-11-d, 1j, 26/04/2012, Castro Verde (Herdade do Torrejão). Ref. **21**-2-a, 3jj, 21/04/2012, Castro Verde (Herdade da Barrigoa). Ref. **21**-3-e, 1j, 21/04/2012, Castro Verde (Herdade da Barrigoa). Ref. **21**-5-f, 1j, 21/04/2012, Castro Verde (Herdade da

Barrigoa). Ref. **21**-6-a, 1j, 21/04/2012, Castro Verde (Herdade da Barrigoa). Ref. **21**-9-a, 1j, 21/04/2012, Castro Verde (Herdade da Barrigoa). Ref. **21**-10-a, 1j, 21/04/2012, Castro Verde (Herdade da Barrigoa). Ref. **23**-2-a, 1j, 03/04/2012, Castro Verde (Monte do Vale das Gretas). Ref. **23**-10-k, 1j, 03/04/2012, Castro Verde (Monte do Vale das Gretas). Ref. **25**-6-i, 1j, 05/04/2012, Castro Verde (Herdade dos Touris e Rolão). Ref. **27**-3-d, 1♀, 24/04/2012, Castro Verde (Herdade das Sesmarias). Ref. **27**-4-e, 1j, 24/04/2012, Castro Verde (Herdade das Sesmarias). Ref. **27**-5-d, 1j, 24/04/2012, Castro Verde (Herdade das Sesmarias). Ref. **27**-7-d, 1♀, 24/04/2012, Castro Verde (Herdade das Sesmarias). Ref. **34**-2-f, 1j, 16/04/2012, Castro Verde (Herdade do Tacanho e Merendeiros). Ref. **34**-3-c, 1j, 16/04/2012, Castro Verde (Herdade do Tacanho e Merendeiros). Ref. **34**-8-c, 1♀ 1j, 16/04/2012, Castro Verde (Herdade do Tacanho e Merendeiros). Ref. **34**-10-c, 1j, 16/04/2012, Castro Verde (Herdade do Tacanho e Merendeiros). Ref. **35**-4-d, 1j, 05/04/2012, Castro Verde (Monte do Seixo). Ref. **35**-8-d, 1j, 05/04/2012, Castro Verde (Monte do Seixo). Ref. **35**-10-f, 1♀, 05/04/2012, Castro Verde (Monte do Seixo). Ref. **35**-11-f, 1j, 05/04/2012, Castro Verde (Monte do Seixo). Ref. **35**-12-c, 1♂, 05/04/2012, Castro Verde (Monte do Seixo). Ref. **36**-3-a, 1j, 11/04/2012, Castro Verde (Herdade dos Bispos). Ref. **36**-4-e, 1♀, 11/04/2012, Castro Verde (Herdade dos Bispos). Ref. **36**-5-a, 1j, 11/04/2012, Castro Verde (Herdade dos Bispos). Ref. **36**-7-d, 1j, 11/04/2012, Castro Verde (Herdade dos Bispos). Ref. **36**-8-i, 2jj, 11/04/2012, Castro Verde (Herdade dos Bispos). Ref. **36**-11-d, 2jj, 11/04/2012, Castro Verde (Herdade dos Bispos). Ref. **37**-2-e, 1j, 09/04/2012, Castro Verde (Herdade de São Marcos). Ref. **37**-4-d, 1♀, 09/04/2012, Castro Verde (Herdade de São Marcos). Ref. **37**-8-b, 1j, 09/04/2012, Castro Verde (Herdade de São

Marcos). Ref. **37**-11-c, 1j, 09/04/2012, Castro Verde (Herdade de São Marcos). Ref. **43**-3-c, 2jj, 21/04/2012, Castro Verde (Monte da Comenda). Ref. **43**-4-d, 1j, 21/04/2012, Castro Verde (Monte da Comenda). Ref. **43**-6-b, 2jj, 21/04/2012, Castro Verde (Monte da Comenda). Ref. **43**-10-h, 1j, 21/04/2012, Castro Verde (Monte da Comenda). Ref. **45**-3-b, 1♀ 1j, 13/04/2012, Castro Verde (Monte das Fontes Barbas Velho). Ref. **45**-5-a, 1♀ 2jj, 13/04/2012, Castro Verde (Monte das Fontes Barbas Velho). Ref. **45**-6-b, 2jj, 13/04/2012, Castro Verde (Monte das Fontes Barbas Velho). Ref. **45**-11-a, 2jj, 13/04/2012, Castro Verde (Monte das Fontes Barbas Velho). Ref. **47**-3-d, 5jj, 09/04/2012, Castro Verde (Herdade do Almarginho). Ref. **47**-4-g, 1j, 09/04/2012, Castro Verde (Herdade do Almarginho). Ref. **47**-6-c, 1j, 09/04/2012, Castro Verde (Herdade do Almarginho). Ref. **47**-7-e, 2jj, 09/04/2012, Castro Verde (Herdade do Almarginho). Ref. **47**-8-b, 1j, 09/04/2012, Castro Verde (Herdade do Almarginho). Ref. **47**-10-c, 1j, 09/04/2012, Castro Verde (Herdade do Almarginho). Ref. **49**-4-b, 1j, 25/04/2012, Castro Verde (Herdade da Navarra). Ref. **49**-5-a, 1♂ 1j, 25/04/2012, Castro Verde (Herdade da Navarra). Ref. **49**-7-c, 2jj, 25/04/2012, Castro Verde (Herdade da Navarra). Ref. **50**-2.c, 1j, 16/04/2012, Castro Verde (Herdade do Torrejão). Ref. **50**-3-f, 1♀, 16/04/2012, Castro Verde (Herdade do Torrejão). Ref. **50**-6-c, 1♀, 16/04/2012, Castro Verde (Herdade do Torrejão). Ref. **50**-10-c, 2jj, 16/04/2012, Castro Verde (Herdade do Torrejão). Ref. **53**-2-d, 3jj, 20/04/2012, Castro Verde (Herdade do Reguengo). Ref. **53**-4-b, 1j, 20/04/2012, Castro Verde (Herdade do Reguengo). Ref. **53**-8-c, 2jj, 20/04/2012, Castro Verde (Herdade do Reguengo). Ref. **55**-2-c, 1j, 21/04/2012, Castro Verde (Herdade da Barrigoa). Ref. **55**-4-d, 2jj, 21/04/2012, Castro Verde (Herdade da Barrigoa). Ref. **55**-5-b, 2jj, 21/04/2012, Castro Verde (Herdade da Barrigoa). Ref. **55**-8-b, 1♀ 4jj, 21/04/2012, Castro

Verde (Herdade da Barrigoa). Ref. **55**-9-g, 2jj, 21/04/2012, Castro Verde (Herdade da Barrigoa). Ref. **55**-10-b, 1j, 21/04/2012, Castro Verde (Herdade da Barrigoa). Ref. **55**-11-a, 2jj, 21/04/2012, Castro Verde (Herdade da Barrigoa). Ref. **56**-6-f, 1j, 18/04/2012, Castro Verde (Herdade da Barrigoa). Ref. **56**-7-d, 2jj, 18/04/2012, Castro Verde (Herdade da Barrigoa). Ref. **56**-8-e, 1j, 18/04/2012, Castro Verde (Herdade da Barrigoa). Ref. **56**-9-f, 3jj, 18/04/2012, Castro Verde (Herdade da Barrigoa). Ref. **56**-10-b, 1j, 18/04/2012, Castro Verde (Herdade da Barrigoa). Ref. **57**-2-f, 1j, 06/04/2012, Castro Verde (Monte da Azinheira). Ref. **57**-6-b, 2jj, 06/04/2012, Castro Verde (Monte da Azinheira). Ref. **57**-7-e, 1j, 06/04/2012, Castro Verde (Monte da Azinheira). Ref. **57**-8-b, 1j, 06/04/2012, Castro Verde (Monte da Azinheira). Ref. **57**-9-b, 1j, 06/04/2012, Castro Verde (Monte da Azinheira). Ref. **58**-1-h, 1j, 07/04/2012, Mértola (Herdade da Benviúda). Ref. **58**-2-a, 2jj, 07/04/2012, Mértola (Herdade da Benviúda). Ref. **58**-4-b, 1j, 07/04/2012, Mértola (Herdade da Benviúda). Ref. **58**-7-e, 1j, 07/04/2012, Mértola (Herdade da Benviúda). Ref. **61**-8-b, 1j, 05/05/2012, Aljustrel (Monte da Torre). Ref. **67**-5-j, 1j, 12/04/2012, Aljustrel (Monte da Chaiça). Ref. **67**-9-c, 1j, 12/04/2012, Aljustrel (Monte da Chaiça). Ref. **70**-2-c, 1j, 21/04/2012, Castro Verde (Monte dos Janeiros). Ref. **70**-4-a, 1j, 21/04/2012, Castro Verde (Monte dos Janeiros). Ref. **70**-5-d, 2jj, 21/04/2012, Castro Verde (Monte dos Janeiros). Ref. **70**-8-f, 1j, 21/04/2012, Castro Verde (Monte dos Janeiros). Ref. **70**-9-f, 1j, 21/04/2012, Castro Verde (Monte dos Janeiros). Ref. **70**-10-a, 1♀, 21/04/2012, Castro Verde (Monte dos Janeiros). Ref. **70**-11-g, 1j, 21/04/2012, Castro Verde (Monte dos Janeiros). Ref. **72**-4-c, 1j, 15/04/2012, Castro Verde (Cumeada Nova). Ref. **72**-6-a, 1j, 15/04/2012, Castro Verde (Cumeada Nova). Ref. **72**-8-e, 5jj, 15/04/2012, Castro Verde (Cumeada Nova). Ref. **72**-9-a, 1j, 15/04/2012,

Castro Verde (Cumeada Nova). Ref. **72-10-d**, 1j, 15/04/2012, Castro Verde (Cumeada Nova). Ref. **72**-11-g, 1j, 15/04/2012, Castro Verde (Cumeada Nova). Ref. **73**-4-e, 2jj, 19/04/2012, Castro Verde (Herdade dos Longos). Ref. **73**-5-h, 3jj, 19/04/2012, Castro Verde (Herdade dos Longos). Ref. **73**-6-c, 6jj, 19/04/2012, Castro Verde (Herdade dos Longos). Ref. **73**-8-c, 2jj, 19/04/2012, Castro Verde (Herdade dos Longos). Ref. **73**-9-j, 1j, 19/04/2012, Castro Verde (Herdade dos Longos). Ref. **73**-10-h, 1j, 19/04/2012, Castro Verde (Herdade dos Longos). Ref. **73**-11-c, 1j, 19/04/2012, Castro Verde (Herdade dos Longos). Ref. **78**-2-d, 1j, 09/04/2012, Castro Verde (Herdade de São Marcos). Ref. **78**-4-b, 1j, 09/04/2012, Castro Verde (Herdade de São Marcos). Ref. **78**-5-b, 1j, 09/04/2012, Castro Verde (Herdade de São Marcos). Ref. **79**-2-e, 1j, 07/04/2012, Castro Verde (Monte da Chada). Ref. **79**-3-h, 1♀, 07/04/2012, Castro Verde (Monte da Chada). Ref. **79**-7-g, 1j, 07/04/2012, Castro Verde (Monte da Chada). Ref. **79**-12-e, 1j, 07/04/2012, Castro Verde (Monte da Chada). Ref. **81**-2-h, 1j, 20/04/2012, Castro Verde (Herdade de Reidias). Ref. **81**-7-g, 2jj, 20/04/2012, Castro Verde (Herdade de Reidias). Ref. **83**-2-g, 1j, 06/05/2012, Castro Verde (Herdade do Álamo). Ref. **83**-6-d, 2jj, 06/05/2012, Castro Verde (Herdade do Álamo). Ref. **83**-9-a, 1j, 06/05/2012, Castro Verde (Herdade do Álamo). Ref. **83**-10-f, 1j, 06/05/2012, Castro Verde (Herdade do Álamo). Ref. **86**-2-a, 1j, 10/05/2012, Castro Verde (Herdade das Mestras). Ref. **86**-8-e, 1j, 10/05/2012, Castro Verde (Herdade das Mestras). Ref. **87**-3-h, 3jj, 06/05/2012, Castro Verde (Herdade das Mestras). Ref. **87**-8-c, 1j, 06/05/2012, Castro Verde (Herdade das Mestras). Ref. **88**-2-o, 1♂ 1j, 25/04/2012, Castro Verde (Herdade da Portela). Ref. **88**-5-i, 1j, 25/04/2012, Castro Verde (Herdade da Portela). Ref. **88**-11-j, 1j, 25/04/2012, Castro Verde (Herdade da Portela). Ref. **89**-3-b, 1j, 06/05/2012, Castro Verde (Herdade do

Carapetal). Ref. **89**-4-h, 1j, 06/05/2012, Castro Verde (Herdade do Carapetal). Ref. **89**-5-k, 2jj, 06/05/2012, Castro Verde (Herdade do Carapetal). Ref. **89**-8-a, 2jj, 06/05/2012, Castro Verde (Herdade do Carapetal). Ref. **89**-10-c, 1j, 06/05/2012, Castro Verde (Herdade dos Pereiros). Ref. **89**-11-h, 1j, 06/05/2012, Castro Verde (Herdade do Carapetal). Ref. **92**-10-b, 1♂, 17/04/2012, Aljustrel (Monte do Carregueiro). Ref. **93**-2-a, 4jj, 17/04/2012, Aljustrel (Herdade da Sobreira). Ref. **93**-4-i, 1j, 17/04/2012, Aljustrel (Herdade da Sobreira). Ref. **93**-6-h, 1j, 17/04/2012, Aljustrel (Herdade da Sobreira). Ref. **93**-9-a, 1j, 17/04/2012, Aljustrel (Herdade da Sobreira). Ref. **96**-2-e, 1j, 11/04/2012, Castro Verde (Herdade dos Bispos). Ref. **96**-4-c, 1j, 11/04/2012, Castro Verde (Herdade dos Bispos). Ref. **96**-7-b, 1j, 11/04/2012, Castro Verde (Herdade dos Bispos). Ref. **96**-10-i, 1j, 11/04/2012, Castro Verde (Herdade dos Bispos). Ref. **97**-3-d, 2jj, 13/04/2012, Castro Verde (Cumeada Nova). Ref. **97**-4-c, 2jj, 13/04/2012, Castro Verde (Cumeada Nova). Ref. **97**-5-c, 1j, 13/04/2012, Castro Verde (Cumeada Nova). Ref. **97**-6-h, 1j, 13/04/2012, Castro Verde (Cumeada Nova). Ref. **97**-7-c, 1j, 13/04/2012, Castro Verde (Cumeada Nova). Ref. **97**-8-c, 2jj, 13/04/2012, Castro Verde (Cumeada Nova). Ref. **97**-11-a, 1j, 13/04/2012, Castro Verde (Cumeada Nova). Ref. **102**-10-a, 1♀, 15/04/2012, Castro Verde (Monte da Fonte). Ref. **104**-7-h, 1♀, 04/04/2012, Castro Verde (Herdade dos Touris e Rolão). Ref. **104**-10-e, 1j, 04/04/2012, Castro Verde (Herdade dos Touris e Rolão). Ref. **107**-5-e, 1♀ 5jj, 18/04/2012, Castro Verde (Herdade das Mestras). Ref. **107**-6-d, 2jj, 18/04/2012, Castro Verde (Herdade das Mestras). Ref. **107**-8-b, 1j, 18/04/2012, Castro Verde (Herdade das Mestras). Ref. **107**-10-e, 1j, 18/04/2012, Castro Verde (Herdade das Mestras). Ref. **108**-4-a, 2jj, 10/04/2012, Castro Verde (Herdade dos Merendeiros). Ref. **108**-5-a, 1j, 10/04/2012, Castro Verde

(Herdade dos Merendeiros). Ref. **108**-6-e, 1♀, 10/04/2012, Castro Verde (Herdade dos Merendeiros). Ref. **108**-7-g, 2jj, 10/04/2012, Castro Verde (Herdade dos Merendeiros). Ref. **108**-8-a, 1j, 10/04/2012, Castro Verde (Herdade dos Merendeiros). Ref. **110**-2-j, 2jj, 04/05/2012, Castro Verde (Monte da Perdigoa). Ref. **110**-3-k, 1j, 04/05/2012, Castro Verde (Monte da Perdigoa). Ref. **110**-4-d, 1j, 04/05/2012, Castro Verde (Monte da Perdigoa). Ref. **110**-6-a, 1j, 04/05/2012, Castro Verde (Monte da Perdigoa). Ref. **110**-8-e, 2jj, 04/05/2012, Castro Verde (Monte da Perdigoa). Ref. **110**-9-f, 2♀♀ 2jj, 04/05/2012, Castro Verde (Monte da Perdigoa). Ref. **110**-10-j, 1j, 04/05/2012, Castro Verde (Monte da Perdigoa). Ref. **110**-11-b, 1j, 04/05/2012, Castro Verde (Monte da Perdigoa). Ref. **111**-7-e, 1j, 16/04/2012, Castro Verde (Monte da Perdigoa). Ref. **111**-8-c, 2jj, 16/04/2012, Castro Verde (Monte da Perdigoa). Ref. **112**-7-h, 1j, 20/04/2012, Castro Verde (Monte do Freire). Ref. **112**-8-i, 1♀ 1j, 20/04/2012, Castro Verde (Monte do Freire). Ref. **113**-9-b, 1♀, 10/04/2012, Castro Verde (Courela do Monte Novo). Ref. **113**-10-c, 1♀, 10/04/2012, Castro Verde (Courela do Monte Novo). Ref. **114**-9-h, 2jj, 08/04/2012, Castro Verde (Herdade das Cuchilhas). Ref. **114**-10-a, 1j, 08/04/2012, Castro Verde (Herdade das Cuchilhas). Ref. **116**-7-d, 1j, 10/04/2012, Castro Verde (Herdade dos Merendeiros). Ref. **116**-9-a, 1j, 10/04/2012, Castro Verde (Herdade dos Merendeiros). Ref. **121**-5-g, 1j, 16/04/2012, Castro Verde (Monte do Tacanho). Ref. **121**-11-a, 2jj, 16/04/2012, Castro Verde (Monte do Tacanho). Ref. **122**-3-b, 1j, 12/04/2012, Castro Verde (Herdade da Chaiça Velha). Ref. **122**-7-c, 2jj, 12/04/2012, Castro Verde (Herdade da Chaiça Velha). Ref. **122**-8-d, 3jj, 12/04/2012, Castro Verde (Herdade da Chaiça Velha). Ref. **122**-9-b, 1j, 12/04/2012, Castro Verde (Herdade da Chaiça Velha). Ref. **122**-10-e, 1j, 12/04/2012, Castro Verde (Herdade da

Chaiça Velha). Ref. **122**-11-a, 2jj, 12/04/2012, Castro Verde (Herdade da Chaiça Velha). Ref. **123**-2-c, 1j, 20/04/2012, Castro Verde (Herdade das bicadas). Ref. **123**-5-g, 1j, 20/04/2012, Castro Verde (Herdade das bicadas). Ref. **123**-7-d, 1j, 20/04/2012, Castro Verde (Herdade das bicadas). Ref. **123**-9-b, 1j, 20/04/2012, Castro Verde (Herdade das bicadas). Ref. **124**-2-b, 2jj, 18/04/2012, Castro Verde (Herdade das Mestras). Ref. **124**-3-a, 6jj, 18/04/2012, Castro Verde (Herdade das Mestras). Ref. **124**-5-a, 2jj, 18/04/2012, Castro Verde (Herdade das Mestras). Ref. **124**-6-j, 2jj, 18/04/2012, Castro Verde (Herdade das Mestras). Ref. **124**-8-d, 3jj, 18/04/2012, Castro Verde (Herdade das Mestras). Ref. **124**-9-g, 1j, 18/04/2012, Castro Verde (Herdade das Mestras). Ref. **124**-10-b, 1j, 18/04/2012, Castro Verde (Herdade das Mestras). Ref. **124**-11-f, 2jj, 18/04/2012, Castro Verde (Herdade das Mestras). Ref. **125**-2-b, 2jj, 17/04/2012, Aljustrel (Herdade da Sobreira). Ref. **125**-4-k, 1j, 17/04/2012, Aljustrel (Herdade da Sobreira). Ref. **125**-6-b, 1j, 17/04/2012, Aljustrel (Herdade da Sobreira). Ref. **125**-7-f, 3jj, 17/04/2012, Aljustrel (Herdade da Sobreira). Ref. **125**-8-d, 2jj, 17/04/2012, Aljustrel (Herdade da Sobreira). Ref. **125**-9-h, 2jj, 17/04/2012, Aljustrel (Herdade da Sobreira). Ref. **125**-10-k, 1j, 17/04/2012, Aljustrel (Herdade da Sobreira). Ref. **125**-11-f, 1j, 17/04/2012, Aljustrel (Herdade da Sobreira). Ref. **126**-3-c, 1j, 05/04/2012, Castro Verde (Monte do Seixo). Ref. **127**-2-b, 1j, 11/04/2012, Castro Verde (Herdade dos Montinhos). Ref. **127**-3-d, 1j, 11/04/2012, Castro Verde (Herdade dos Montinhos). Ref. **127**-5-b, 1♀, 11/04/2012, Castro Verde (Herdade dos Montinhos). Ref. **127**-7-i, 1j, 11/04/2012, Castro Verde (Herdade dos Montinhos). Ref. **127**-8-b, 1♂ 4jj, 11/04/2012, Castro Verde (Herdade dos Montinhos). Ref. **127**-9-f, 2jj, 11/04/2012, Castro Verde (Herdade dos Montinhos). Ref. **127**-10-a, 1j, 11/04/2012, Castro Verde (Herdade dos Montinhos). Ref. **127**-

11-e, 1♀, 11/04/2012, Castro Verde (Herdade dos Montinhos). Ref. **128**-2-a, 1j, 04/04/2012, Castro Verde (Herdade de Carriça-Viseus). Ref. **128**-8-f, 2jj, 04/04/2012, Castro Verde (Herdade de Carriça-Viseus). Ref. **129**-6-d, 2jj, 08/05/2012, Castro Verde (Herdade da Zibreira). Ref. **129**-11-g, 1j, 08/05/2012, Castro Verde (Herdade da Zibreira). Ref. **131**-3-c, 1j, 04/05/2012, Castro Verde (Herdade do Torrejão). Ref. **131**-7-d, 1♀, 04/05/2012, Castro Verde (Herdade do Torrejão). Ref. **132**-2-k, 1♀, 06/05/2012, Castro Verde (Monte das Cabeceiras). Ref. **132**-6-c, 1♀ 1j, 06/05/2012, Castro Verde (Monte das Cabeceiras). Ref. **132**-8-a, 2jj, 06/05/2012, Castro Verde (Monte das Cabeceiras). Ref. **132**-11-b, 1j, 06/05/2012, Castro Verde (Monte das Cabeceiras). Ref. **133**-5-c, 1j, 28/04/2012, Castro Verde (Monte da Albergaria). Ref. **133**-7-c, 2jj, 28/04/2012, Castro Verde (Monte da Albergaria). Ref. **A46**-8-c, 1j, 10/05/2012, Aljustrel (Monte da Chaiça). Ref. **A57**-6-c, 1♀, 08/05/2012, Aljustrel (Herdade de Corta Rabos). Ref. **A166**-2-f, 1j, 07/05/2012, Castro Verde (Herdade dos Brunhachos). Ref. **A166**-5-b, 2jj, 07/05/2012, Castro Verde (Herdade dos Brunhachos). Ref. **A260**-6-g, 1j, 07/05/2012, Castro Verde (Herdade de Reidias). Ref. **A260**-7-d, 1♀ 1j, 07/05/2012, Castro Verde (Herdade de Reidias). Ref. **A260**-11-g, 1j, 07/05/2012, Castro Verde (Herdade de Reidias). Ref. **A297**-6-b, 1j, 09/05/2012, Castro Verde (Lagoa da Mó). Ref. **A297**-9-c, 1j, 09/05/2012, Castro Verde (Lagoa da Mó). Ref. **A299**-6-d, 1j, 08/05/2012, Castro Verde (Lagoa da Mó). Ref. **A349**-3-d, 1j, 10/05/2012, Castro Verde (Herdade das Mestras). Ref. **A349**-4-a, 2jj, 10/05/2012, Castro Verde (Herdade das Mestras). Ref. **A349**-10-d, 1j, 10/05/2012, Castro Verde (Herdade das Mestras). Ref. **A423**-2-j, 4jj, 08/05/2012, Castro Verde (Herdade da Zibreira). Ref. **A423**-3-i, 1j, 08/05/2012, Castro Verde (Herdade da Zibreira). Ref. **A423**-5-f, 3jj, 08/05/2012, Castro Verde (Herdade da Zibreira). Ref.

A423-7-d, 4jj, 08/05/2012, Castro Verde (Herdade da Zibreira). Ref. **A505**-3-d, 2jj, 08/05/2012, Castro Verde (Herdade do Reguengo). Ref. **A505**-5-e, 2jj, 08/05/2012, Castro Verde (Herdade do Reguengo). Ref. **A505**-6-b, 1j, 08/05/2012, Castro Verde (Herdade do Reguengo). Ref. **A527**-2-e, 2jj, 07/05/2012, Castro Verde (Herdade dos Bispos). Ref. **A527**-4-k, 2jj, 07/05/2012, Castro Verde (Herdade dos Bispos). Ref. **A527**-5-d, 3jj, 07/05/2012, Castro Verde (Herdade dos Bispos). Ref. **A527**-6-f, 2jj, 07/05/2012, Castro Verde (Herdade dos Bispos). Ref. **A527**-8-g, 4jj, 07/05/2012, Castro Verde (Herdade dos Bispos). Ref. **A527**-9-e, 2jj, 07/05/2012, Castro Verde (Herdade dos Bispos). Ref. **A527**-11-g, 2jj, 07/05/2012, Castro Verde (Herdade dos Bispos). Ref. **A999**-3-b, 1j, 09/05/2012, Castro Verde (Herdade dos Pereiros). Ref. **A999**-11-h, 1j, 09/05/2012, Castro Verde (Herdade dos Pereiros).

Mangora acalypha **(Walckenaer, 1802)**

Material examined: Ref. **73**-11-c, 1♀, 19/04/2012, Castro Verde (Herdade dos Longos). Ref. **125**-8-d, 1♂, 17/04/2012, Aljustrel (Herdade da Sobreira).

Family CHEIRACANTHIIDAE Wagner, 1887

Cheiracanthium pennatum **Simon, 1878.**

Material examined: Ref. **23**-10-k, 1j, 03/04/2012, Castro Verde (Monte do Vale das Gretas). Ref. **25**-1-c, 1j, 05/04/2012, Castro Verde (Herdade dos Touris e Rolão). Ref. **25**-10-b, 1♂, 05/04/2012, Castro Verde (Herdade dos Touris e Rolão). Ref. **43**-6-b, 1♂, 21/04/2012, Castro Verde (Monte da Comenda). Ref. **49**-3-e, 1j, 25/04/2012, Castro Verde (Herdade da Navarra). Ref. **53**-11-e, 1♂, 20/04/2012,

Castro Verde (Herdade do Reguengo). Ref. **55**-5-b, 1♂, 21/04/2012, Castro Verde (Herdade da Barrigoa). Ref. **61**-2-k, 1♂, 05/05/2012, Aljustrel (Monte da Torre). Ref. **61**-3-c, 1♀, 05/05/2012, Aljustrel (Monte da Torre). Ref. **67**-3-c, 1♀, 12/04/2012, Aljustrel (Monte da Chaiça). Ref. **67**-4-a, 1♀, 12/04/2012, Aljustrel (Monte da Chaiça). Ref. **72**-10-d, 1j, 15/04/2012, Castro Verde (Cumeada Nova). Ref. **83**-11-a, 1j, 06/05/2012, Castro Verde (Herdade do Álamo). Ref. **89**-2-c, 1j, 06/05/2012, Castro Verde (Herdade do Carapetal). Ref. **96**-5-b, 1j, 11/04/2012, Castro Verde (Herdade dos Bispos). Ref. **107**-9-a, 1j, 18/04/2012, Castro Verde (Herdade das Mestras). Ref. **109**-4-b, 1♀, 24/04/2012, Castro Verde (Monte da Achada). Ref. **123**-6-c, 1j, 20/04/2012, Castro Verde (Herdade das bicadas). Ref. **A46**-7-c1, 1♂, 10/05/2012, Aljustrel (Monte da Chaiça). Ref. **A46**-10-g, 1♀, 10/05/2012, Aljustrel (Monte da Chaiça). Ref. **A297**-7-a, 1♀, 09/05/2012, Castro Verde (Lagoa da Mó). Ref. **A299**-10-f, 1♂, 08/05/2012, Castro Verde (Lagoa da Mó).

Family CLUBIONIDAE Simon, 1878

Porrhoclubiona vegeta (Simon, 1918).

Material examined: Ref. **27**-10-d, 1♀ 5jj, 24/04/2012, Castro Verde (Herdade das Sesmarias). Ref. **86**-3-d, 4jj, 10/05/2012, Castro Verde (Herdade das Mestras). Ref. **89**-2-c, 2jj, 06/05/2012, Castro Verde (Herdade do Carapetal). Ref. **123**-6-c, 1j, 20/04/2012, Castro Verde (Herdade das bicadas). Ref. **A46**-11-g, 1j, 10/05/2012, Aljustrel (Monte da Chaiça). Ref. **A166**-8-a, 1♀, 07/05/2012, Castro Verde (Herdade dos Brunhachos). Ref. **A505**-6-b, 1j, 08/05/2012, Castro Verde (Herdade do Reguengo).

Family DICTYNIDAE O. Pickard-Cambridge, 1871

Argenna subnigra **(O.P.-Cambridge, 1861).**

Material examined: Ref. **4**-10-e, 1♀, 15/04/2012, Castro Verde (Heredade das Mouras). Ref. **37**-6-a, 1j, 09/04/2012, Castro Verde (Herdade de São Marcos). Ref. **47**-2-j, 2jj, 09/04/2012, Castro Verde (Herdade do Almarginho). Ref. **57**-5-e, 1♀, 06/04/2012, Castro Verde (Monte da Azinheira). Ref. **57**-9-b, 1♀ 1j, 06/04/2012, Castro Verde (Monte da Azinheira). Ref. **58**-1-h, 1♀, 07/04/2012, Mértola (Herdade da Benviúda). Ref. **72**-5-a, 1♀, 15/04/2012, Castro Verde (Cumeada Nova). Ref. **72**-10-d, 1♀, 15/04/2012, Castro Verde (Cumeada Nova). Ref. **109**-6-f, 1j, 24/04/2012, Castro Verde (Monte da Achada). Ref. **110**-3-k, 2♀♀, 04/05/2012, Castro Verde (Monte da Perdigoa). Ref. **110**-8-e, 2♀♀, 04/05/2012, Castro Verde (Monte da Perdigoa). Ref. **123**-2-c, 1j, 20/04/2012, Castro Verde (Herdade das bicadas). Ref. **123**-5-g, 1j, 20/04/2012, Castro Verde (Herdade das bicadas). Ref. **123**-9-b, 1♀, 20/04/2012, Castro Verde (Herdade das bicadas). Ref. **123**-11-c, 1♀, 20/04/2012, Castro Verde (Herdade das bicadas). Ref. **126**-8-f, 12jj, 05/04/2012, Castro Verde (Monte do Seixo). Ref. **126**-11-b, 1j, 05/04/2012, Castro Verde (Monte do Seixo). Ref. **126**-12-a, 1j, 05/04/2012, Castro Verde (Monte do Seixo). Ref. **127**-5-b, 1♀, 11/04/2012, Castro Verde (Herdade dos Montinhos). Ref. **127**-6-c, 1♀, 11/04/2012, Castro Verde (Herdade dos Montinhos). Ref. **127**-7-i, 1♀, 11/04/2012, Castro Verde (Herdade dos Montinhos). Ref. **A349**-8-h, 1♀, 10/05/2012, Castro Verde (Herdade das Mestras). Ref. **A482**-7-e, 1j, 09/05/2012, Castro Verde (Amendoeira Nova).

***Marilynia bicolor* (Simon, 1870)**

Material examined: Ref. **43**-5-e, 1j, 21/04/2012, Castro Verde (Monte da Comenda). Ref. **53**-8-c, 1j, 20/04/2012, Castro Verde (Herdade do Reguengo). Ref. **78**-4-b, 1j, 09/04/2012, Castro Verde (Herdade de São Marcos). Ref. **123**-6-c, 1j, 20/04/2012, Castro Verde (Herdade das bicadas). Ref. **133**-5-c, 1♀, 28/04/2012, Castro Verde (Monte da Albergaria). Ref. **133-6-g**, 1♀, 28/04/2012, Castro Verde (Monte da Albergaria). Ref. **A57**-3-h, 1♂, 08/05/2012, Aljustrel (Herdade de Corta Rabos). Ref. **A482**-3-b, 1j, 09/05/2012, Castro Verde (Amendoeira Nova).

***Nigma puella* (Simon, 1870)**

Material examined: Ref. **4**-5-j, 1♂, 15/04/2012, Castro Verde (Heredade das Mouras).

Family DYSDERIDAE C. L. Koch, 1837

***Harpactea minoccii* Ferrandez, 1982**

Material examined: Ref. **93**-8-f, 1♂, 17/04/2012, Aljustrel (Herdade da Sobreira).

Family GNAPHOSIDAE Pocock, 1898

Gnaphosidae sp.; (51 jj. indet.).

***Aphantaulax* sp.**

Material examined: Ref. **53**-5-e, 1j, 20/04/2012, Castro Verde (Herdade do Reguengo). Ref. **53**-9-b, 1j, 20/04/2012, Castro Verde (Herdade do Reguengo).

Ref. **79**-9-d, 1j, 07/04/2012, Castro Verde (Monte da Chada). Ref. **A166**-9-c, 1j, 07/05/2012, Castro Verde (Herdade dos Brunhachos). Ref. **A166**-10-b, 1j, 07/05/2012, Castro Verde (Herdade dos Brunhachos). Ref. **A999**-8-j, 1j, 09/05/2012, Castro Verde (Herdade dos Pereiros).

Civizelotes civicus **(Simon, 1878).**

Material examined: Ref. **A505**-8-c, 1♂ 1j, 08/05/2012, Castro Verde (Herdade do Reguengo).

Civizelotes ibericus **Senglet, 2012**

Material examined: Ref. **6**-5-c, 1j, 24/04/2012, Castro Verde (Herdade de A de Neves da Marinha). Ref. **23**-2-a, 1♂, 03/04/2012, Castro Verde (Monte do Vale das Gretas). Ref. **49**-7-c, 1j, 25/04/2012, Castro Verde (Herdade da Navarra). Ref. **55**-3-g, 1♂ 2jj, 21/04/2012, Castro Verde (Herdade da Barrigoa). Ref. **67**-7-f, 1♀, 12/04/2012, Aljustrel (Monte da Chaiça). Ref. **83**-3-b, 1♂ 1j, 06/05/2012, Castro Verde (Herdade do Álamo). Ref. **87**-7-g, 1j, 06/05/2012, Castro Verde (Herdade das Mestras). Ref. **89**-11-h, 1♂, 06/05/2012, Castro Verde (Herdade do Carapetal). Ref. **110**-11-b, 1♀, 04/05/2012, Castro Verde (Monte da Perdigoa). Ref. **127**-11-e, 1♂, 11/04/2012, Castro Verde (Herdade dos Montinhos). Ref. **A46**-8-c, 1j, 10/05/2012, Aljustrel (Monte da Chaiça). Ref. **A260**-11-g, 1♂, 07/05/2012, Castro Verde (Herdade de Reidias). Ref. **A297**-9-c, 1j, 09/05/2012, Castro Verde (Lagoa da Mó). Ref. **A423**-3-i, 1j, 08/05/2012, Castro Verde (Herdade da Zibreira). Ref. **A423**-5-f, 2jj, 08/05/2012, Castro Verde (Herdade da Zibreira). Ref. **A423**-10-d, 3jj, 08/05/2012, Castro Verde (Herdade da Zibreira). Ref. **A999**-10-b, 1♂, 09/05/2012, Castro Verde (Herdade dos Pereiros).

***Gnaphosa* sp.**

Material examined: Ref. **4**-11-b, 2jj, 15/04/2012, Castro Verde (Heredade das Mouras). Ref. **6**-3-c, 1j, 24/04/2012, Castro Verde (Herdade de A de Neves da Marinha). Ref. **11**-7-c, 1j, 15/04/2012, Castro Verde (Monte do Broco e Capitão). Ref. **15**-3-b, 1j, 12/04/2012, Castro Verde (Lagoa da Mó). Ref. **21**-2-a, 1j, 21/04/2012, Castro Verde (Herdade da Barrigoa). Ref. **21**-7-a, 1j, 21/04/2012, Castro Verde (Herdade da Barrigoa). Ref. **21**-9-a, 1j, 21/04/2012, Castro Verde (Herdade da Barrigoa). Ref. **23**-4-a, 1j, 03/04/2012, Castro Verde (Monte do Vale das Gretas). Ref. **23**-5-i, 3jj, 03/04/2012, Castro Verde (Monte do Vale das Gretas). Ref. **23**-11-i, 1j, 03/04/2012, Castro Verde (Monte do Vale das Gretas). Ref. **25**-3-d, 1j, 05/04/2012, Castro Verde (Herdade dos Touris e Rolão). Ref. **25**-8-a, 1j, 05/04/2012, Castro Verde (Herdade dos Touris e Rolão). Ref. **36**-9-b, 2jj, 11/04/2012, Castro Verde (Herdade dos Bispos). Ref. **37**-2-e, 1j, 09/04/2012, Castro Verde (Herdade de São Marcos). Ref. **37**-3-a, 1j, 09/04/2012, Castro Verde (Herdade de São Marcos). Ref. **37**-8-b, 2jj, 09/04/2012, Castro Verde (Herdade de São Marcos). Ref. **37**-10-e, 1j, 09/04/2012, Castro Verde (Herdade de São Marcos). Ref. **47**-3-d, 2jj, 09/04/2012, Castro Verde (Herdade do Almarginho). Ref. **50**-7-c, 1j, 16/04/2012, Castro Verde (Herdade do Torrejão). Ref. **50**-9-f, 1j, 16/04/2012, Castro Verde (Herdade do Torrejão). Ref. **53**-5-e, 1j, 20/04/2012, Castro Verde (Herdade do Reguengo). Ref. **56**-9-f, 1j, 18/04/2012, Castro Verde (Herdade da Barrigoa). Ref. **57**-1-a, 1j, 06/04/201, Castro Verde (Monte da Azinheira). Ref. **57**-3-i, 1j, 06/04/201, Castro Verde (Monte da Azinheira). Ref. **61**-3-c, 1j, 05/05/2012, Aljustrel (Monte da Torre). Ref. **67**-3-c, 1j, 12/04/2012, Aljustrel (Monte da Chaiça). Ref. **67**-5-j, 2jj, 12/04/2012, Aljustrel

(Monte da Chaiça). Ref. **67**-8-d, 1j, 12/04/2012, Aljustrel (Monte da Chaiça). Ref. **70**-3-a, 1j, 21/04/2012, Castro Verde (Monte dos Janeiros). Ref. **73**-9-j, 1j, 19/04/2012, Castro Verde (Herdade dos Longos). Ref. **73**-11-c, 1j, 19/04/2012, Castro Verde (Herdade dos Longos). Ref. **78**-4-b, 1j, 09/04/2012, Castro Verde (Herdade de São Marcos). Ref. **78**-7-b, 1j, 09/05/2012, Castro Verde (Herdade de São Marcos). Ref. **79**-11-b, 1j, 07/04/2012, Castro Verde (Monte da Chada). Ref. **86**-3-d, 1j, 10/05/2012, Castro Verde (Herdade das Mestras). Ref. **87**-3-h, 1j, 06/05/2012, Castro Verde (Herdade das Mestras). Ref. **87**-10-c, 1j, 06/05/2012, Castro Verde (Herdade das Mestras). Ref. **89**-5-k, 1j, 06/05/2012, Castro Verde (Herdade do Carapetal). Ref. **89**-8-a, 1j, 06/05/2012, Castro Verde (Herdade do Carapetal). Ref. **92**-2-c, 1j, 17/04/2012, Aljustrel (Monte do Carregueiro). Ref. **92**-4-c, 1j, 17/04/2012, Aljustrel (Monte do Carregueiro). Ref. **93**-4-i, 1j, 17/04/2012, Aljustrel (Herdade da Sobreira). Ref. **93**-6-h, 1j, 17/04/2012, Aljustrel (Herdade da Sobreira). Ref. **104**-9-d, 1j, 04/04/2012, Castro Verde (Herdade dos Touris e Rolão). Ref. **109**-3-i, 1j, 24/04/2012, Castro Verde (Monte da Achada). Ref. **110**-5-c, 1j, 04/05/2012, Castro Verde (Monte da Perdigoa). Ref. **113**-3-c, 1j, 10/04/2012, Castro Verde (Courela do Monte Novo). Ref. **114**-3-h, 2jj, 08/04/2012, Castro Verde (Herdade das Cuchilhas). Ref. **114**-10-a, 1♂, 08/04/2012, Castro Verde (Herdade das Cuchilhas). Ref. **121**-2-d, 1j, 16/04/2012, Castro Verde (Monte do Tacanho). Ref. **121**-7-e, 1j, 16/04/2012, Castro Verde (Monte do Tacanho). Ref. **122**-6-d, 2jj, 12/04/2012, Castro Verde (Herdade da Chaiça Velha). Ref. **122**-7-c, 1j, 12/04/2012, Castro Verde (Herdade da Chaiça Velha). Ref. **123**-9-b, 1j, 20/04/2012, Castro Verde (Herdade das bicadas). Ref. **125**-7-f, 2jj, 17/04/2012, Aljustrel (Herdade da Sobreira). Ref. **125**-8-d, 2jj, 17/04/2012, Aljustrel (Herdade da Sobreira). Ref. **125**-9-h, 1j, 17/04/2012,

Aljustrel (Herdade da Sobreira). Ref. **125**-10-k, 1j, 17/04/2012, Aljustrel (Herdade da Sobreira). Ref. **128**-7-d, 1j, 04/04/2012, Castro Verde (Herdade de Carriça-Viseus). Ref. **129**-6-d, 1j, 08/05/2012, Castro Verde (Herdade da Zibreira). Ref. **131**-2-f, 1j, 04/05/2012, Castro Verde (Herdade do Torrejão). Ref. **131**-7-d, 1j, 04/05/2012, Castro Verde (Herdade do Torrejão). Ref. **132**-2-k, 1j, 06/05/2012, Castro Verde (Monte das Cabeceiras). Ref. **A46**-4-j, 1j, 10/05/2012, Aljustrel (Monte da Chaiça). Ref. **A46**-7-c2, 1j, 10/05/2012, Aljustrel (Monte da Chaiça). Ref. **A57**-5-d, 1j, 08/05/2012, Aljustrel (Herdade de Corta Rabos). Ref. **A57**-10-e, 1j, 08/05/2012, Aljustrel (Herdade de Corta Rabos). Ref. **A57**-11-g, 1j, 08/05/2012, Aljustrel (Herdade de Corta Rabos). Ref. **A166**-9-c, 1j, 07/05/2012, Castro Verde (Herdade dos Brunhachos). Ref. **A297**-6-b, 2jj, 09/05/2012, Castro Verde (Lagoa da Mó). Ref. **A299**-5-f, 1j, 08/05/2012, Castro Verde (Lagoa da Mó). Ref. **A388**-9-c, 1j, 10/05/2012, Castro Verde (Monte da Achada). Ref. **A423**-4-i, 1j, 08/05/2012, Castro Verde (Herdade da Zibreira). Ref. **A527**-8-g, 1j, 07/05/2012, Castro Verde (Herdade dos Bispos). Ref. **A527**-9-e, 2jj, 07/05/2012, Castro Verde (Herdade dos Bispos). Ref. **A999**-7-g, 1j, 09/05/2012, Castro Verde (Herdade dos Pereiros). Ref. **A999**-8-j, 1j, 09/05/2012, Castro Verde (Herdade dos Pereiros). Ref. **A999**-9-g, 1j, 09/05/2012, Castro Verde (Herdade dos Pereiros). Ref. **A999**-10-b, 1j, 09/05/2012, Castro Verde (Herdade dos Pereiros).

Haplodrassus rhodanicus **(Simon, 1914)**

Material examined: Ref. **55**-11-a, 1♂ 1j, 21/04/2012, Castro Verde (Herdade da Barrigoa). Ref. **57**-7-e, 1♂, 06/04/2012, Castro Verde (Monte da Azinheira). Ref. **58**-5-c, 1♂, 07/04/2012, Mértola (Herdade da Benviúda). Ref. **58**-12-d, 1♂, 07/04/2012, Mértola (Herdade da Benviúda). Ref. **61**-3-c, 1♀, 05/05/2012,

Aljustrel (Monte da Torre). Ref. **73**-3-h, 1♀, 19/04/2012, Castro Verde (Herdade dos Longos). Ref. **83**-6-d, 1♂, 06/05/2012, Castro Verde (Herdade do Álamo). Ref. **83**-10-f, 1♀, 06/05/2012, Castro Verde (Herdade do Álamo). Ref. **86**-2-a, 1♂, 10/05/2012, Castro Verde (Herdade das Mestras). Ref. **89**-4-h, 1♂, 06/05/2012, Castro Verde (Herdade do Carapetal). Ref. **89**-11-h, 1♀, 06/05/2012, Castro Verde (Herdade do Carapetal). Ref. **93**-9-a, 1♂, 17/04/2012, Aljustrel (Herdade da Sobreira). Ref. **124**-4-i, 1j, 18/04/2012, Castro Verde (Herdade das Mestras). Ref. **125**-2-b, 1♂, 17/04/2012, Aljustrel (Herdade da Sobreira). Ref. **125**-3-k, 1j, 17/04/2012, Aljustrel (Herdade da Sobreira). Ref. **127**-8-b, 1♂, 11/04/2012, Castro Verde (Herdade dos Montinhos). Ref. **127**-11-e, 1♂, 11/04/2012, Castro Verde (Herdade dos Montinhos). Ref. **132**-4-a, 1♂, 06/05/2012, Castro Verde (Monte das Cabeceiras). Ref. **A46**-8-c, 1♂ 1j, 10/05/2012, Aljustrel (Monte da Chaiça). Ref. **A297**-8-j, 1♂, 09/05/2012, Castro Verde (Lagoa da Mó). Ref. **A299**-11R-b, 1♂, 08/05/2012, Castro Verde (Lagoa da Mó). Ref. **A505**-2-c, 1j, 08/05/2012, Castro Verde (Herdade do Reguengo). Ref. **A505**-7-f, 1♂, 08/05/2012, Castro Verde (Herdade do Reguengo). Ref. **A527**-6-f, 1♀, 07/05/2012, Castro Verde (Herdade dos Bispos). Ref. **A999**-3-b, 1♀ 1j, 09/05/2012, Castro Verde (Herdade dos Pereiros). Ref. **A999**-6-e, 1j, 09/05/2012, Castro Verde (Herdade dos Pereiros).

***Haplodrassus* sp.**

Material examined: Ref. **4**-3-j, 1j, 15/04/2012, Castro Verde (Heredade das Mouras). Ref. **34**-4-a, 1j, 16/04/2012, Castro Verde (Herdade do Tacanho e Merendeiros). Ref. **34**-7-b, 1j, 16/04/2012, Castro Verde (Herdade do Tacanho e Merendeiros). Ref. **36**-2-a, 1j, 11/04/2012, Castro Verde (Herdade dos Bispos). Ref. **36**-4-e, 1j,

11/04/2012, Castro Verde (Herdade dos Bispos). Ref. **36**-10-g, 1j, 11/04/2012, Castro Verde (Herdade dos Bispos).Ref. **43**-2-d, 1j, 21/04/2012, Castro Verde (Monte da Comenda). Ref. **49**-8-d, 1j, 25/04/2012, Castro Verde (Herdade da Navarra). Ref. **56**-8-e, 1j, 18/04/2012, Castro Verde (Herdade da Barrigoa). Ref. **67**-6-i, 1j, 12/04/2012, Aljustrel (Monte da Chaiça). Ref. **73**-7-a, 1j, 19/04/2012, Castro Verde (Herdade dos Longos). Ref. **88**-8-g, 1j, 25/04/2012, Castro Verde (Herdade da Portela). Ref. **89**-10-c, 1j, 06/05/2012, Castro Verde (Herdade do Carapetal). Ref. **93**-6-h, 1j, 17/04/2012, Aljustrel (Herdade da Sobreira). Ref. **108**-11-a, 1j, 10/04/2012, Castro Verde (Herdade dos Merendeiros). Ref. **110**-5-c, 1j, 04/05/2012, Castro Verde (Monte da Perdigoa). Ref. **113**-11-c, 1j, 10/04/2012, Castro Verde (Courela do Monte Novo). Ref. **116**-7-d, 1j, 10/04/2012, Castro Verde (Herdade dos Merendeiros). Ref. **122**-3-b, 2jj, 12/04/2012, Castro Verde (Herdade da Chaiça Velha). Ref. **126**-6-a, 1j, 05/04/2012, Castro Verde (Monte do Seixo). Ref. **129**-10-e, 1j, 08/05/2012, Castro Verde (Herdade da Zibreira). Ref. **129**-11-g, 1j, 08/05/2012, Castro Verde (Herdade da Zibreira). Ref. **131**-8-a, 1j, 04/05/2012, Castro Verde (Herdade do Torrejão). Ref. **132**-11-b, 1j, 06/05/2012, Castro Verde (Monte das Cabeceiras). Ref. **A297**-7-a, 1j, 09/05/2012, Castro Verde (Lagoa da Mó). Ref. **A299**-2-c, 1j, 08/05/2012, Castro Verde (Lagoa da Mó).

***Leptodrassus albidus* Simon, 1914**

Material examined: Ref. **11**-11-e, 1j, 15/04/2012, Castro Verde (Monte do Broco e Capitão). Ref. **21**-5-f, 1j, 21/04/2012, Castro Verde (Herdade da Barrigoa). Ref. **23**-1-a, 1j, 03/04/2012, Castro Verde (Monte do Vale das Gretas). Ref. **25**-10-b, 1j, 05/04/2012, Castro Verde (Herdade dos Touris e Rolão). Ref. **36**-2-a, 1j,

11/04/2012, Castro Verde (Herdade dos Bispos). Ref. **37**-4-d, 1♂, 09/04/2012, Castro Verde (Herdade de São Marcos). Ref. **45**-9-a, 1j, 13/04/2012, Castro Verde (Monte das Fontes Barbas Velho). Ref. **45**-10-b, 1j, 13/04/2012, Castro Verde (Monte das Fontes Barbas Velho). Ref. **50**-10-c, 1j, 16/04/2012, Castro Verde (Herdade do Torrejão). Ref. **70**-3-a, 1♂ 1j, 21/04/2012, Castro Verde (Monte dos Janeiros). Ref. **70**-7-a, 1♂, 21/04/2012, Castro Verde (Monte dos Janeiros). Ref. **73**-7-a, 1♂, 19/04/2012, Castro Verde (Herdade dos Longos). Ref. **87**-2-c, 1♂ 1j, 06/05/2012, Castro Verde (Herdade das Mestras). Ref. **121**-6-a, 1j, 16/04/2012, Castro Verde (Monte do Tacanho). Ref. **121**-10-d, 1j, 16/04/2012, Castro Verde (Monte do Tacanho). Ref. **125**-3-k, 1j, 17/04/2012, Aljustrel (Herdade da Sobreira). Ref. **125**-11-f, 1♂, 17/04/2012, Aljustrel (Herdade da Sobreira). Ref. **127**-8-b, 1j, 11/04/2012, Castro Verde (Herdade dos Montinhos). Ref. **128**-8-f, 1j, 04/04/2012, Castro Verde (Herdade de Carriça-Viseus). Ref. **129**-2-k, 2jj, 08/05/2012, Castro Verde (Herdade da Zibreira). Ref. **132**-11-b, 1♂, 06/05/2012, Castro Verde (Monte das Cabeceiras). Ref. **A46**-9-f, 1♂, 10/05/2012, Aljustrel (Monte da Chaiça). Ref. **A299**-3-i, 1♂, 08/05/2012, Castro Verde (Lagoa da Mó). Ref. **A505**-6-b, 1j, 08/05/2012, Castro Verde (Herdade do Reguengo). Ref. **A999**-9-g, 1j, 09/05/2012, Castro Verde (Herdade dos Pereiros).

***Marinarozelotes minutus* (Crespo, 2010)**

Material examined: Ref. **4**-11-b, 1♀, 15/04/2012, Castro Verde (Heredade das Mouras). Ref. **11**-3-d, 1♂, 15/04/2012, Castro Verde (Monte do Broco e Capitão). Ref. **79**-2-e, 1♂, 07/04/2012, Castro Verde (Monte da Chada). Ref. **83**-10-f, 1♂, 06/05/2012, Castro Verde (Herdade do Álamo). Ref. **89**-5-k, 1j, 06/05/2012, Castro Verde (Herdade do Carapetal). Ref. **89**-8-a, 1j, 06/05/2012, Castro Verde

(Herdade do Carapetal). Ref. **A505**-8-c, 1♀, 08/05/2012, Castro Verde (Herdade do Reguengo). Ref. **A999**-7-g, 1♀, 09/05/2012, Castro Verde (Herdade dos Pereiros). Ref. **A999**-10-b, 1♂, 09/05/2012, Castro Verde (Herdade dos Pereiros).

***Micaria* sp.**

Material examined: Ref. **49**-10-b, 1j, 25/04/2012, Castro Verde (Herdade da Navarra). Ref. **50**-4-c, 1j, 16/04/2012, Castro Verde (Herdade do Torrejão). Ref. **54**-7-g, 1j, 25/04/2012, Castro Verde (Herdade do Roncanho). Ref. **58**-8-e, 1j, 07/04/2012, Mértola (Herdade da Benviúda).

***Nomisia exornata* (C. L. Koch, 1839)**

Material examined: Ref. **6**-2-a, 1j, 24/04/2012, Castro Verde (Herdade de A de Neves da Marinha). Ref. **6**-5-c, 1j, 24/04/2012, Castro Verde (Herdade de A de Neves da Marinha). Ref. **6**-9-h, 1j, 24/04/2012, Castro Verde (Herdade de A de Neves da Marinha). Ref. **6**-10-e, 2jj, 24/04/2012, Castro Verde (Herdade de A de Neves da Marinha). Ref. **9**-6-g, 1j, 25/04/2012, Castro Verde (COSA - Herdade de São Marcos). Ref. **9**-9-d, 1j, 25/04/2012, Castro Verde (COSA - Herdade de São Marcos). Ref. **14**-6-c, 1j, 18/04/2012, Castro Verde (Lagoa da Mó). Ref. **15**-8-a, 1j, 12/04/2012, Castro Verde (Lagoa da Mó). Ref. **21**-4-f, 1j, 21/04/2012, Castro Verde (Herdade da Barrigoa). Ref. **21**-8-d, 1j, 21/04/2012, Castro Verde (Herdade da Barrigoa). Ref. **21**-11-c, 1j, 21/04/2012, Castro Verde (Herdade da Barrigoa). Ref. **23**-1-a, 1j, 03/04/2012, Castro Verde (Monte do Vale das Gretas). Ref. **23**-3-c, 1j, 03/04/2012, Castro Verde (Monte do Vale das Gretas). Ref. **25**-11-a, 1j, 05/04/2012, Castro Verde (Herdade dos Touris e Rolão). Ref. **36**-5-a, 1j, 11/04/2012, Castro Verde (Herdade dos Bispos). Ref. **36**-8-i, 1j, 11/04/2012,

Castro Verde (Herdade dos Bispos). Ref. **36**-9-b, 1j, 11/04/2012, Castro Verde (Herdade dos Bispos). Ref. **36**-10-g, 1j, 11/04/2012, Castro Verde (Herdade dos Bispos). Ref. **43**-7-b, 1j, 21/04/2012, Castro Verde (Monte da Comenda). Ref. **47**-1-a, 1j, 09/04/2012, Castro Verde (Herdade do Almarginho). Ref. **47**-12-c, 1j, 09/04/2012, Castro Verde (Herdade do Almarginho). Ref. **49**-4-b, 1j, 25/04/2012, Castro Verde (Herdade da Navarra). Ref. **49**-11-h, 1j, 25/04/2012, Castro Verde (Herdade da Navarra). Ref. **55**-2-c, 1j, 21/04/2012, Castro Verde (Herdade da Barrigoa). Ref. **55**-5-b, 1j, 21/04/2012, Castro Verde (Herdade da Barrigoa). Ref. **55**-8-b, 1j, 21/04/2012, Castro Verde (Herdade da Barrigoa). Ref. **56**-5-g, 1j, 18/04/2012, Castro Verde (Herdade da Barrigoa). Ref. **57**-3-i, 1j, 06/04/2012, Castro Verde (Monte da Azinheira). Ref. **72**-5-a, 1j, 15/04/2012, Castro Verde (Cumeada Nova). Ref. **73**-7-a, 1j, 19/04/2012, Castro Verde (Herdade dos Longos). Ref. **73**-10-h, 1j, 19/04/2012, Castro Verde (Herdade dos Longos). Ref. **79**-11-b, 1j, 07/04/2012, Castro Verde (Monte da Chada). Ref. **81**-2-h, 1j, 20/04/2012, Castro Verde (Herdade de Reidias). Ref. **81**-6-j, 1j, 20/04/2012, Castro Verde (Herdade de Reidias). Ref. **81**-9-e, 1j, 20/04/2012, Castro Verde (Herdade de Reidias). Ref. **89**-2-c, 2jj, 06/05/2012, Castro Verde (Herdade do Carapetal). Ref. **89**-4-h, 1j, 06/05/2012, Castro Verde (Herdade do Carapetal). Ref. **89**-9-h, 1j, 06/05/2012, Castro Verde (Herdade do Carapetal). Ref. **93**-5-e, 2jj, 17/04/2012, Aljustrel (Herdade da Sobreira). Ref. **96**-11-c, 1j, 11/04/2012, Castro Verde (Herdade dos Bispos). Ref. **97**-3-d, 1j, 13/04/2012, Castro Verde (Cumeada Nova). Ref. **97**-7-c, 1j, 13/04/2012, Castro Verde (Cumeada Nova). Ref. **104**-5-a, 2jj, 04/04/2012, Castro Verde (Herdade dos Touris e Rolão). Ref. **107**-3-d, 1j, 18/04/2012, Castro Verde (Herdade das Mestras). Ref. **108**-8-a, 1j, 10/04/2012, Castro Verde (Herdade dos Merendeiros). Ref. **108**-9-d, 1j,

10/04/2012, Castro Verde (Herdade dos Merendeiros). Ref. **108**-10-d, 1j, 10/04/2012, Castro Verde (Herdade dos Merendeiros). Ref. **109**-7-h, 1j, 24/04/2012, Castro Verde (Monte da Achada). Ref. **109**-8-c, 1j, 24/04/2012, Castro Verde (Monte da Achada). Ref. **110**-2-j, 1j, 04/05/2012, Castro Verde (Monte da Perdigoa). Ref. **110**-6-a, 2jj, 04/05/2012, Castro Verde (Monte da Perdigoa). Ref. **111**-7-e, 2jj, 16/04/2012, Castro Verde (Monte da Perdigoa). Ref. **111**-8-c, 1j, 16/04/2012, Castro Verde (Monte da Perdigoa). Ref. **111**-10-c, 1j, 16/04/2012, Castro Verde (Monte da Perdigoa). Ref. **114**-1-c, 1j, 08/04/2012, Castro Verde (Herdade das Cuchilhas). Ref. **114**-7-d, 1j, 08/04/2012, Castro Verde (Herdade das Cuchilhas). Ref. **114**-12-d, 1j, 08/04/2012, Castro Verde (Herdade das Cuchilhas). Ref. **116**-5-d, 1j, 10/04/2012, Castro Verde (Herdade dos Merendeiros). Ref. **116**-9-a, 1j, 10/04/2012, Castro Verde (Herdade dos Merendeiros). Ref. **121**-4-b, 1j, 16/04/2012, Castro Verde (Monte do Tacanho). Ref. **126**-3-c, 1j, 05/04/2012, Castro Verde (Monte do Seixo). Ref. **129**-6-d, 1j, 08/05/2012, Castro Verde (Herdade da Zibreira). Ref. **129**-8-b, 1j, 08/05/2012, Castro Verde (Herdade da Zibreira). Ref. **132**-3-j, 1j, 06/05/2012, Castro Verde (Monte das Cabeceiras). Ref. **132**-6-c, 1j, 06/05/2012, Castro Verde (Monte das Cabeceiras). Ref. **133**-2-b, 1j, 28/04/2012, Castro Verde (Monte da Albergaria). Ref. **133**-8-f, 1j, 28/04/2012, Castro Verde (Monte da Albergaria). Ref. **A46**-7-c2, 1♂, 10/05/2012, Aljustrel (Monte da Chaiça). Ref. **A166**-8-a, 1j, 07/05/2012, Castro Verde (Herdade dos Brunhachos). Ref. **A166**-10-b, 1j, 07/05/2012, Castro Verde (Herdade dos Brunhachos). Ref. **A299**-6-d, 1j, 08/05/2012, Castro Verde (Lagoa da Mó). Ref. **A299**-9-b, 1♂, 08/05/2012, Castro Verde (Lagoa da Mó). Ref. **A349**-4-a, 1j, 10/05/2012, Castro Verde (Herdade das Mestras). Ref. **A388**-11-a, 1j, 10/05/2012, Castro Verde (Monte da Achada). Ref. **A505**-8-c, 1j,

08/05/2012, Castro Verde (Herdade do Reguengo). Ref. **A999**-7-g, 1j, 09/05/2012, Castro Verde (Herdade dos Pereiros). Ref. **A999**-10-b, 1j, 09/05/2012, Castro Verde (Herdade dos Pereiros).

***Setaphis carmeli* (O.P.-Cambridge, 1872)**

Material examined: Ref. **4**-3-j, 1♂ 2jj, 15/04/2012, Castro Verde (Heredade das Mouras). Ref. **4**-5-j, 1♂, 15/04/2012, Castro Verde (Heredade das Mouras). Ref. **4**-6-c, 1j, 15/04/2012, Castro Verde (Heredade das Mouras). Ref. **4**-8-h, 1j, 15/04/2012, Castro Verde (Heredade das Mouras). Ref. **6**-2-a, 1j, 24/04/2012, Castro Verde (Herdade de A de Neves da Marinha). Ref. **15**-10-f, 1j, 12/04/2012, Castro Verde (Lagoa da Mó). Ref. **19**-2-c, 1♂, 26/04/2012, Castro Verde (Herdade do Torrejão). Ref. **23**-3-c, 1♂, 03/04/2012, Castro Verde (Monte do Vale das Gretas). Ref. **25**-6-i, 1♂, 05/04/2012, Castro Verde (Herdade dos Touris e Rolão). Ref. **25**-10-b, 1♂, 05/04/2012, Castro Verde (Herdade dos Touris e Rolão). Ref. **36**-2-a, 2jj, 11/04/2012, Castro Verde (Herdade dos Bispos). Ref. **36**-3-a, 1j, 11/04/2012, Castro Verde (Herdade dos Bispos). Ref. **43**-2-d, 1j, 21/04/2012, Castro Verde (Monte da Comenda). Ref. **47**-8-b, 1♂, 09/04/2012, Castro Verde (Herdade do Almarginho). Ref. **49**-9-a, 2jj, 25/04/2012, Castro Verde (Herdade da Navarra). Ref. **50**-8-c, 1♂, 16/04/2012, Castro Verde (Herdade do Torrejão). Ref. **51**-9-e, 1j, 13/04/2012, Castro Verde (Herdade do Torrejão). Ref. **56**-6-f, 1♂, 18/04/2012, Castro Verde (Herdade da Barrigoa). Ref. **57**-3-i, 1j, 06/04/2012, Castro Verde (Monte da Azinheira). Ref. **58**-4-b, 1♂, 07/04/2012, Mértola (Herdade da Benviúda). Ref. **70**-2-c, 1♂, 21/04/2012, Castro Verde (Monte dos Janeiros). Ref. **73**-10-h, 1j, 19/04/2012, Castro Verde (Herdade dos Longos). Ref. **81**-6-j, 1j, 20/04/2012, Castro Verde (Herdade de

Reidias). Ref. **87**-2-c, 1j, 06/05/2012, Castro Verde (Herdade das Mestras). Ref. **89**-3-b, 1j, 06/05/2012, Castro Verde (Herdade do Carapetal). Ref. **89**-10-c, 1j, 06/05/2012, Castro Verde (Herdade do Carapetal). Ref. **104**-3-b, 1j, 04/04/2012, Castro Verde (Herdade dos Touris e Rolão). Ref. **104**-5-a, 1♂, 04/04/2012, Castro Verde (Herdade dos Touris e Rolão). Ref. **104**-10-e, 1♂, 04/04/2012, Castro Verde (Herdade dos Touris e Rolão). Ref. **104**-12-a, 1♂ 1j, 04/04/2012, Castro Verde (Herdade dos Touris e Rolão). Ref. **107**-6-d, 3jj, 18/04/2012, Castro Verde (Herdade das Mestras). Ref. **110**-2-j, 1j, 04/05/2012, Castro Verde (Monte da Perdigoa). Ref. **114**-4-a, 1j, 08/04/2012, Castro Verde (Herdade das Cuchilhas). Ref. **121**-3-j, 1j, 16/04/2012, Castro Verde (Monte do Tacanho). Ref. **121**-7-e, 1♂, 16/04/2012, Castro Verde (Monte do Tacanho). Ref. **122**-7-c, 1j, 12/04/2012, Castro Verde (Herdade da Chaiça Velha). Ref. **122**-10-e, 1♂, 12/04/2012, Castro Verde (Herdade da Chaiça Velha). Ref. **124**-3-a, 1j, 18/04/2012, Castro Verde (Herdade das Mestras). Ref. **125**-2-b, 1♀ 1j, 17/04/2012, Aljustrel (Herdade da Sobreira). Ref. **125**-7-f, 2jj, 17/04/2012, Aljustrel (Herdade da Sobreira). Ref. **126**-12-a, 1j, 05/04/2012, Castro Verde (Monte do Seixo). Ref. **127**-5-b, 1j, 11/04/2012, Castro Verde (Herdade dos Montinhos). Ref. **127**-8-b, 2jj, 11/04/2012, Castro Verde (Herdade dos Montinhos). Ref. **127**-9-f, 1♀, 11/04/2012, Castro Verde (Herdade dos Montinhos). Ref. **128**-8-f, 1j, 04/04/2012, Castro Verde (Herdade de Carriça-Viseus). Ref. **129**-4-a, 1♂, 08/05/2012, Castro Verde (Herdade da Zibreira). Ref. **131**-2-f, 1♀, 04/05/2012, Castro Verde (Herdade do Torrejão). Ref. **132**-9-a, 1j, 06/05/2012, Castro Verde (Monte das Cabeceiras). Ref. **132**-10-g, 1j, 06/05/2012, Castro Verde (Monte das Cabeceiras). Ref. **133**-2-b, 1j, 28/04/2012, Castro Verde (Monte da Albergaria). Ref. **133**-6-g, 1♂, 28/04/2012, Castro Verde (Monte

da Albergaria). Ref. **A46**-3-h, 1♀, 10/05/2012, Aljustrel (Monte da Chaiça). Ref. **A46**-9-f, 1♀, 10/05/2012, Aljustrel (Monte da Chaiça). Ref. **A57**-2-a, 1♀, 08/05/2012, Aljustrel (Herdade de Corta Rabos). Ref. **A166**-6-b, 1j, 07/05/2012, Castro Verde (Herdade dos Brunhachos). Ref. **A297**-2-b, 1♀ 1j, 09/05/2012, Castro Verde (Lagoa da Mó). Ref. **A297**-5-a, 1j, 09/05/2012, Castro Verde (Lagoa da Mó). Ref. **A297**-10-f, 1j, 09/05/2012, Castro Verde (Lagoa da Mó). Ref. **A349**-6-k, 1♀, 10/05/2012, Castro Verde (Herdade das Mestras). Ref. **A505**-10-g, 1j, 805/2012, Castro Verde (Herdade do Reguengo). Ref. **A527**-5-d, 1j, 07/05/2012, Castro Verde (Herdade dos Bispos).

***Zelotes fulvopilosus* (Simon, 1878)**

Material examined: Ref. **70**-2-c, 1♂, 21/04/2012, Castro Verde (Monte dos Janeiros).

***Zelotes* sp.**

Material examined: Ref. **4**-9-a, 1j, 15/04/2012, Castro Verde (Heredade das Mouras). Ref. **6**-4-e, 1j, 24/04/2012, Castro Verde (Herdade de A de Neves da Marinha). Ref. **23**-1-a, 1j, 03/04/2012, Castro Verde (Monte do Vale das Gretas). Ref. **27**-10-d, 1j, 24/04/2012, Castro Verde (Herdade das Sesmarias). Ref. **34**-7-b, 1j, 16/04/2012, Castro Verde (Herdade do Tacanho e Merendeiros). Ref. **43**-2-d, 3jj, 21/04/2012, Castro Verde (Monte da Comenda). Ref. **43**-5-e, 1j, 21/04/2012, Castro Verde (Monte da Comenda). Ref. **43**-7-b, 1j, 21/04/2012, Castro Verde (Monte da Comenda). Ref. **43**-10-h, 1j, 21/04/2012, Castro Verde (Monte da Comenda). Ref. **47**-6-c, 4jj, 09/04/2012, Castro Verde (Herdade do Almarginho). Ref. **49**-7-c, 2jj, 25/04/2012, Castro Verde (Herdade da Navarra). Ref. **50**-3-f, 1j, 16/04/2012, Castro Verde (Herdade do Torrejão). Ref. **55**-8-b, 1j,

21/04/2012, Castro Verde (Herdade da Barrigoa). Ref. **55**-9-g, 1j, 21/04/2012, Castro Verde (Herdade da Barrigoa). Ref. **57**-6-b, 2jj, 06/04/2012, Castro Verde (Monte da Azinheira). Ref. **57**-8-b, 1j, 06/04/2012, Castro Verde (Monte da Azinheira). Ref. **57**-9-b, 2jj, 06/04/2012, Castro Verde (Monte da Azinheira). Ref. **57**-11-i, 1j, 06/04/2012, Castro Verde (Monte da Azinheira). Ref. **58**-3-g, 1j, 07/04/2012, Mértola (Herdade da Benviúda). Ref. **67**-5-j, 1j, 12/04/2012, Aljustrel (Monte da Chaiça). Ref. **67**-8-d, 2jj, 12/04/2012, Aljustrel (Monte da Chaiça). Ref. **67**-11-f, 1j, 12/04/2012, Aljustrel (Monte da Chaiça). Ref. **70**-5-d, 1j, 21/04/2012, Castro Verde (Monte dos Janeiros). Ref. **70**-8-f, 1j, 21/04/2012, Castro Verde (Monte dos Janeiros). Ref. **72**-6-a, 1j, 15/04/2012, Castro Verde (Cumeada Nova). Ref. **72**-11-g, 1j, 15/04/2012, Castro Verde (Cumeada Nova). Ref. **73**-5-h, 1j, 19/04/2012, Castro Verde (Herdade dos Longos). Ref. **78**-11-b, 1j, 09/04/2012, Castro Verde (Herdade de São Marcos). Ref. **79**-10-i, 1j, 07/04/2012, Castro Verde (Monte da Chada). Ref. **83**-9-a, 1j, 06/05/2012, Castro Verde (Herdade do Álamo). Ref. **83**-10-f, 2jj, 06/05/2012, Castro Verde (Herdade do Álamo). Ref. **86**-10-a, 1j, 10/05/2012, Castro Verde (Herdade das Mestras). Ref. **87**-8-c, 1j, 06/05/2012, Castro Verde (Herdade das Mestras). Ref. **89**-10-c, 1j, 06/05/2012, Castro Verde (Herdade do Carapetal). Ref. **93**-8-f, 1j, 17/04/2012, Aljustrel (Herdade da Sobreira). Ref. **109**-6-f, 1j, 24/04/2012, Castro Verde (Monte da Achada). Ref. **109**-9-e, 1j, 24/04/2012, Castro Verde (Monte da Achada). Ref. **110**-3-k, 1j, 04/05/2012, Castro Verde (Monte da Perdigoa). Ref. **116**-4-b, 1j, 10/04/2012, Castro Verde (Herdade dos Merendeiros). Ref. **121**-5-g, 1j, 16/04/2012, Castro Verde (Monte do Tacanho). Ref. **124**-2-b, 1j, 18/04/2012, Castro Verde (Herdade das Mestras). Ref. **125**-8-d, 2jj, 17/04/2012, Aljustrel (Herdade da Sobreira). Ref. **127**-9-f, 1j, 11/04/2012, Castro Verde (Herdade dos

Montinhos). Ref. **A46**-2-g, 1j, 10/05/2012, Aljustrel (Monte da Chaiça). Ref. **A166**-6-b, 1j, 07/05/2012, Castro Verde (Herdade dos Brunhachos). Ref. **A166**-7-a, 1j, 07/05/2012, Castro Verde (Herdade dos Brunhachos). Ref. **A297**-7-a, 1j, 09/05/2012, Castro Verde (Lagoa da Mó). Ref. **A388**-10-f, 1j, 10/05/201, Castro Verde (Monte da Achada). Ref. **A505**-10-g, 1j, 08/05/2012, Castro Verde (Herdade do Reguengo). Ref. **A527**-9-e, 1j, 07/05/2012, Castro Verde (Herdade dos Bispos). Ref. **A999**-7-g, 1j, 09/05/2012, Castro Verde (Herdade dos Pereiros). Ref. **A999**-8-j, 1j, 09/05/2012, Castro Verde (Herdade dos Pereiros).

***Zelotes* sp. nov.**

Material examined: Ref. **73**-2-c, 1♂ 2jj, 19/04/2012, Castro Verde (Herdade dos Longos). Ref. **A505**-5-e, 1♂ 1j, 08/05/2012, Castro Verde (Herdade do Reguengo).

Family LINYPHIIDAE Blackwall, 1859

Linyphiidae sp. (1028 jj. Indet.).

***Agyneta pseudorurestris* Wunderlich, 1980**

Material examined: Ref. **4**-2-c, 4♂♂ 3♀♀ 6jj, 15/04/2012, Castro Verde (Heredade das Mouras). Ref. **4**-3-j, 1♂ 6jj, 15/04/2012, Castro Verde (Heredade das Mouras). Ref. **4**-4-g, 2♀♀ 10jj, 15/04/2012, Castro Verde (Heredade das Mouras). Ref. **4**-5-j, 2♂♂ 3♀♀ 11jj, 15/04/2012, Castro Verde (Heredade das Mouras). Ref. **4**-6-c, 1♂ 1♀ 4jj, 15/04/2012, Castro Verde (Heredade das Mouras). Ref. **4**-7-g, 1♂ 3jj, 15/04/2012, Castro Verde (Heredade das Mouras). Ref. **4**-9-a, 2♂♂ 2♀♀

20jj, 15/04/2012, Castro Verde (Heredade das Mouras). Ref. **4**-10-e, 2♀♀ 17jj, 15/04/2012, Castro Verde (Heredade das Mouras). Ref. **4**-11-b, 1♂ 1♀ 12jj, 15/04/2012, Castro Verde (Heredade das Mouras). Ref. **6**-3-c, 1♀ 2jj, 24/04/2012, Castro Verde (Herdade de A de Neves da Marinha). Ref. **6**-5-c, 1♀, 24/04/2012, Castro Verde (Herdade de A de Neves da Marinha). Ref. **6**-8-h, 1♀ 5jj, 24/04/2012, Castro Verde (Herdade de A de Neves da Marinha). Ref. **6**-9-h, 2♂♂ 3jj, 24/04/2012, Castro Verde (Herdade de A de Neves da Marinha). Ref. **9**-2-g, 1♂ 1♀, 25/04/2012, Castro Verde (COSA - Herdade de São Marcos). Ref. **9**-6-g, 1♀, 25/04/2012, Castro Verde (COSA - Herdade de São Marcos). Ref. **9**-7-i, 1♂, 25/04/2012, Castro Verde (COSA - Herdade de São Marcos). Ref. **9**-9-d, 2♀♀, 25/04/2012, Castro Verde (COSA - Herdade de São Marcos). Ref. **11**-2-b, 2♂♂ 5jj, 15/04/2012, Castro Verde (Monte do Broco e Capitão). Ref. **11**-4-b, 1♂ 2jj, 15/04/2012, Castro Verde (Monte do Broco e Capitão). Ref. **11**-4-d, 1j, 15/04/2012, Castro Verde (Monte do Broco e Capitão). Ref. **11**-5-h, 3♂♂ 18jj, 15/04/2012, Castro Verde (Monte do Broco e Capitão). Ref. **11**-7-c, 1♂ 4jj, 15/04/2012, Castro Verde (Monte do Broco e Capitão). Ref. **11**-8-f, 1♂, 15/04/2012, Castro Verde (Monte do Broco e Capitão). Ref. **11**-10-f, 1♀, 15/04/2012, Castro Verde (Monte do Broco e Capitão). Ref. **11**-11-e, 2♀♀ 13jj, 15/04/2012, Castro Verde (Monte do Broco e Capitão). Ref. **14**-4-a, 1♂, 18/04/2012, Castro Verde (Lagoa da Mó). Ref. **14**-11-c, 1♀ 1j, 18/04/2012, Castro Verde (Lagoa da Mó). Ref. **15**-2-h, 1♀ 1j, 12/04/2012, Castro Verde (Lagoa da Mó). Ref. **15**-3-b, 1♂, 12/04/2012, Castro Verde (Lagoa da Mó). Ref. **19**-10-e, 1♂ 2jj, 26/04/2012, Castro Verde (Herdade do Torrejão). Ref. **21**-3-e, 1♂, 21/04/2012, Castro Verde (Herdade da Barrigoa). Ref. **21**-6-a, 1♂ 2jj, 21/04/2012, Castro Verde (Herdade da Barrigoa). Ref. **23**-3-c, 1♀, 03/04/2012,

Castro Verde (Monte do Vale das Gretas). Ref. **23**-7-c, 1♀ 2jj, 03/04/2012, Castro Verde (Monte do Vale das Gretas). Ref. **23**-9-a, 1♂ 9jj, 03/04/2012, Castro Verde (Monte do Vale das Gretas). Ref. **23**-10-k, 1♂ 1♀ 9jj, 03/04/2012, Castro Verde (Monte do Vale das Gretas). Ref. **23**-12-b, 1♂ 3jj, 03/04/2012, Castro Verde (Monte do Vale das Gretas). Ref. **25**-3-d, 1♀ 5jj, 05/04/2012, Castro Verde (Herdade dos Touris e Rolão). Ref. **25**-6-i, 1♂ 3jj, 05/04/2012, Castro Verde (Herdade dos Touris e Rolão). Ref. **25**-7-a, 1♂ 1♀ 4jj, 05/04/2012, Castro Verde (Herdade dos Touris e Rolão). Ref. **25**-9-c, 2♀♀ 2jj, 05/04/2012, Castro Verde (Herdade dos Touris e Rolão). Ref. **25**-11-a, 1♀ 6jj, 05/04/2012, Castro Verde (Herdade dos Touris e Rolão). Ref. **27**-3-d, 1♀, 24/04/2012, Castro Verde (Herdade das Sesmarias). Ref. **27**-4-e, 1♂ 1j, 24/04/2012, Castro Verde (Herdade das Sesmarias). Ref. **27**-6-e, 1♂ 4jj, 24/04/2012, Castro Verde (Herdade das Sesmarias). Ref. **27**-8-c, 1♂, 24/04/2012, Castro Verde (Herdade das Sesmarias). Ref. **34**-11-d, 1♂ 1j, 16/04/2012, Castro Verde (Herdade do Tacanho e Merendeiros). Ref. **35**-4-d, 1♂ 2jj, 05/04/2012, Castro Verde (Monte do Seixo). Ref. **35**-6-g, 1♀, 05/04/2012, Castro Verde (Monte do Seixo). Ref. **35**-7-d, 1♀ 4jj, 05/04/2012, Castro Verde (Monte do Seixo). Ref. **35**-8-d, 1♂ 1♀ 6jj, 05/04/2012, Castro Verde (Monte do Seixo). Ref. **36**-2-a, 2♀♀ 2jj, 11/04/2012, Castro Verde (Herdade dos Bispos). Ref. **36**-3-e, 1♂ 1♀ 2jj, 11/04/2012, Castro Verde (Herdade dos Bispos). Ref. **36**-4-e, 2♀♀ 1j, 11/04/2012, Castro Verde (Herdade dos Bispos). Ref. **36**-5-a, 1♀ 4jj, 11/04/2012, Castro Verde (Herdade dos Bispos). Ref. **36**-8-i, 1♀ 4jj, 11/04/2012, Castro Verde (Herdade dos Bispos). Ref. **36**-9-b, 1♀ 1j, 11/04/2012, Castro Verde (Herdade dos Bispos). Ref. **36**-10-g, 1♀ 5jj, 11/04/2012, Castro Verde (Herdade dos Bispos). Ref. **37**-6-a, 1♂ 1j, 09/04/2012, Castro Verde (Herdade de São Marcos). Ref. **37**-9-d, 1♀ 6jj,

09/04/2012, Castro Verde (Herdade de São Marcos). Ref. **45**-6-b, 1♂, 13/04/2012, Castro Verde (Monte das Fontes Barbas Velho). Ref. **45**-9-a, 1♀ 1j, 13/04/2012, Castro Verde (Monte das Fontes Barbas Velho). Ref. **47**-4-g, 1♂ 1j, 09/04/2012, Castro Verde (Herdade do Almarginho). Ref. **47**-5-g, 1♀ 2jj, 09/04/2012, Castro Verde (Herdade do Almarginho). Ref. **47**-6-c, 1♀ 3jj, 09/04/2012, Castro Verde (Herdade do Almarginho). Ref. **47**-10-c, 1♀ 3jj, 09/04/2012, Castro Verde (Herdade do Almarginho). Ref. **49**-2-d, 3♂♂ 11jj, 25/04/2012, Castro Verde (Herdade da Navarra). Ref. **49**-3-e, 1♂ 1♀ 4jj, 25/04/2012, Castro Verde (Herdade da Navarra). Ref. **49**-4-b, 1♀ 6jj, 25/04/2012, Castro Verde (Herdade da Navarra). Ref. **49**-5-a, 2♂♂ 2♀♀ 2jj, 25/04/2012, Castro Verde (Herdade da Navarra). Ref. **49**-7-c, 1♂ 4jj, 25/04/2012, Castro Verde (Herdade da Navarra). Ref. **49**-8-d, 3♂♂ 2♀♀ 6jj, 25/04/2012, Castro Verde (Herdade da Navarra). Ref. **49**-9-a, 1♂ 1♀ 9jj, 25/04/2012, Castro Verde (Herdade da Navarra). Ref. **49**-10-b, 3♂♂ 1♀ 14jj, 25/04/2012, Castro Verde (Herdade da Navarra). Ref. **49**-11-h, 1♂ 11jj, 25/04/2012, Castro Verde (Herdade da Navarra). Ref. **50**-2-c, 1♀ 4jj, 16/04/2012, Castro Verde (Herdade do Torrejão). Ref. **50**-11-f, 1♂ 2jj, 16/04/2012, Castro Verde (Herdade do Torrejão). Ref. **53**-5-e, 1♂ 2jj, 20/04/2012, Castro Verde (Herdade do Reguengo). Ref. **53**-6-d, 1j, 20/04/2012, Castro Verde (Herdade do Reguengo). Ref. **54**-7-g, 1♂, 25/04/2012, Castro Verde (Herdade do Roncanho). Ref. **54**-11-c, 1♂ 1j, 25/04/2012, Castro Verde (Herdade do Roncanho). Ref. **55**-2-c, 1♀ 1j, 21/04/2012, Castro Verde (Herdade da Barrigoa). Ref. **55**-3-g, 1♀ 2jj, 21/04/2012, Castro Verde (Herdade da Barrigoa). Ref. **55**-5-b, 1♂ 1j, 21/04/2012, Castro Verde (Herdade da Barrigoa). Ref. **55**-11-a, 1♀ 2jj, 21/04/2012, Castro Verde (Herdade da Barrigoa). Ref. **56**-4-b, 1♀ 1j, 18/04/2012, Castro Verde (Herdade da Barrigoa). Ref. **56**-7-

d, 1♂ 1♀ 1j, 18/04/2012, Castro Verde (Herdade da Barrigoa). Ref. **56**-8-e, 2jj, 18/04/2012, Castro Verde (Herdade da Barrigoa). Ref. **56**-10-b, 1♂ 2jj, 18/04/2012, Castro Verde (Herdade da Barrigoa). Ref. **61-3-c**, 1♂ 2♀♀ 3jj, 05/05/2012, Aljustrel (Monte da Torre). Ref. **61**-4-a, 1♀ 6jj, 05/05/2012, Aljustrel (Monte da Torre). Ref. **61**-5-h, 3♀♀ 6jj, 05/05/2012, Aljustrel (Monte da Torre). Ref. **61**-6-j, 4♀♀ 17jj, 05/05/2012, Aljustrel (Monte da Torre). Ref. **61**-7-e, 1♂ 1j, 05/05/2012, Aljustrel (Monte da Torre). Ref. **61**-8-b, 3♀♀ 4jj, 05/05/2012, Aljustrel (Monte da Torre). Ref. **61**-9-f, 1♂ 1♀ 1j, 05/05/2012, Aljustrel (Monte da Torre). Ref. **61**-10-h, 2♂♂ 2♀♀ 2jj, 05/05/2012, Aljustrel (Monte da Torre). Ref. **61**-11-e, 2♂♂ 3♀♀ 4jj, 05/05/2012, Aljustrel (Monte da Torre). Ref. **67**-2-e, 1♂ 1♀ 6jj, 12/04/2012, Aljustrel (Monte da Chaiça). Ref. **67**-5-j, 1♀ 1j, 12/04/2012, Aljustrel (Monte da Chaiça). Ref. **67**-7-f, 1♀ 3jj, 12/04/2012, Aljustrel (Monte da Chaiça). Ref. **67**-8-d, 1♂ 6jj, 12/04/2012, Aljustrel (Monte da Chaiça). Ref. **70**-3-a, 2♂♂ 7jj, 21/04/2012, Castro Verde (Monte dos Janeiros). Ref. **70**-6-e, 1♂ 1♀ 5jj, 21/04/2012, Castro Verde (Monte dos Janeiros). Ref. **70**-7-a, 2♂♂ 11jj, 21/04/2012, Castro Verde (Monte dos Janeiros). Ref. **70**-8-f, 1♂ 1♀ 8jj, 21/04/2012, Castro Verde (Monte dos Janeiros). Ref. **70**-9-f, 1♂ 2♀♀ 5jj, 21/04/2012, Castro Verde (Monte dos Janeiros). Ref. **73**-4-e, 1♂ 1j, 19/04/2012, Castro Verde (Herdade dos Longos). Ref. **78**-2-d, 1♂ 2jj, 09/04/2012, Castro Verde (Herdade de São Marcos). Ref. **79**-2-e, 1♀ 3jj, 07/04/2012, Castro Verde (Monte da Chada). Ref. **79**-6-e, 1♂ 4jj, 07/04/2012, Castro Verde (Monte da Chada). Ref. **81**-8-h, 2♀♀, 20/04/2012, Castro Verde (Herdade de Reidias). Ref. **83**-4-i, 1♀, 06/05/2012, Castro Verde (Herdade do Álamo). Ref. **83**-5-k, 1♂ 1♀, 06/05/2012, Castro Verde (Herdade do Álamo). Ref. **83**-7-h, 2♂♂ 2♀♀ 1j, 06/05/2012, Castro Verde (Herdade do Álamo). Ref. **83**-8-f, 3♂♂ 2♀♀ 2jj,

06/05/2012, Castro Verde (Herdade do Álamo). Ref. **83**-9-a, 2♂♂ 2jj, 06/05/2012, Castro Verde (Herdade do Álamo). Ref. **83**-11-a, 1♂ 2♀♀ 3jj, 06/05/2012, Castro Verde (Herdade do Álamo). Ref. **86**-4-c, 1♂ 1♀ 1j, 10/05/2012, Castro Verde (Herdade das Mestras). Ref. **86**-8-e, 1♂ 5jj, 10/05/2012, Castro Verde (Herdade das Mestras). Ref. **86**-10-a, 1♀ 4jj, 10/05/2012, Castro Verde (Herdade das Mestras). Ref. **86**-11-h, 1♂ 2♀♀ 2jj, 10/05/2012, Castro Verde (Herdade das Mestras). Ref. **87**-4-h, 1♀ 1j, 06/05/2012, Castro Verde (Herdade das Mestras). Ref. **87**-5-h, 1♂, 06/05/2012, Castro Verde (Herdade das Mestras). Ref. **87**-6-b, 1♂ 1♀, 06/05/2012, Castro Verde (Herdade das Mestras). Ref. **87**-7-g, 1♀, 06/05/2012, Castro Verde (Herdade das Mestras). Ref. **87**-9-a, 3♂♂ 1♀ 2jj, 06/05/2012, Castro Verde (Herdade das Mestras). Ref. **87**-10-c, 1♂ 1♀ 2jj, 06/05/2012, Castro Verde (Herdade das Mestras). Ref. **88**-2-o, 2♀♀ 4jj, 25/04/2012, Castro Verde (Herdade da Portela). Ref. **88**-3-d, 2♂♂ 2♀♀ 3jj, 25/04/2012, Castro Verde (Herdade da Portela). Ref. **88**-4-a, 1♂ 6jj, 25/04/2012, Castro Verde (Herdade da Portela). Ref. **88**-5-i, 1♂ 1♀ 1j, 25/04/2012, Castro Verde (Herdade da Portela). Ref. **88**-6-c, 2♂♂ 2jj, 25/04/2012, Castro Verde (Herdade da Portela). Ref. **88**-9-g, 2♀♀ 5jj, 25/04/2012, Castro Verde (Herdade da Portela). Ref. **88**-11-j, 1♂ 7jj, 25/04/2012, Castro Verde (Herdade da Portela). Ref. **89**-7-e, 1♀, 06/05/2012, Castro Verde (Herdade do Carapetal). Ref. **89**-8-a, 1♀ 1j, 06/05/2012, Castro Verde (Herdade do Carapetal). Ref. **89**-9-h, 1♂ 3jj, 06/05/2012, Castro Verde (Herdade do Carapetal). Ref. **89**-10-c, 2♀♀ 2jj, 06/05/2012, Castro Verde (Herdade do Carapetal). Ref. **92**-4-c, 1♂ 2jj, 17/04/2012, Aljustrel (Monte do Carregueiro). Ref. **92**-10-b, 1♂, 17/04/2012, Aljustrel (Monte do Carregueiro). Ref. **93**-10-a, 1♀ 2jj, 17/04/2012, Aljustrel (Herdade da Sobreira). Ref. **96**-7-b, 1♂, 11/04/2012, Castro Verde (Herdade dos

Bispos). Ref. **96**-8-a, 2♂♂ 5jj, 11/04/2012, Castro Verde (Herdade dos Bispos). Ref. **97**-3-d, 1♂, 13/04/2012, Castro Verde (Cumeada Nova). Ref. **97**-10-i, 1♂ 1j, 13/04/2012, Castro Verde (Cumeada Nova). Ref. **102**-3-g, 4jj, 15/04/2012, Castro Verde (Monte da Fonte). Ref. **104**-1-b, 1♀, 04/04/2012, Castro Verde (Herdade dos Touris e Rolão). Ref. **104**-7-h, 3jj, 04/04/2012, Castro Verde (Herdade dos Touris e Rolão). Ref. **104**-10-e, 1♂ 1♀ 2jj, 04/04/2012, Castro Verde (Herdade dos Touris e Rolão). Ref. **104**-11-c, 1♀ 1j, 04/04/2012, Castro Verde (Herdade dos Touris e Rolão). Ref. **107**-4-g, 1♂ 4jj, 18/04/2012, Castro Verde (Herdade das Mestras). Ref. **107**-6-d, 1♂, 18/04/2012, Castro Verde (Herdade das Mestras). Ref. **107**-7-f, 1♂ 1j, 18/04/2012, Castro Verde (Herdade das Mestras). Ref. **107**-8-b, 1♂ 4jj, 18/04/2012, Castro Verde (Herdade das Mestras). Ref. **107**-9-a, 1♀ 11jj, 18/04/2012, Castro Verde (Herdade das Mestras). Ref. **108**-7-g, 1j, 10/04/2012, Castro Verde (Herdade dos Merendeiros). Ref. **108**-8-a, 1♂, 10/04/2012, Castro Verde (Herdade dos Merendeiros). Ref. **108**-11-a, 1♂ 1♀ 1j, 10/04/2012, Castro Verde (Herdade dos Merendeiros). Ref. **109**-2-e, 1♀ 1j, 24/04/2012, Castro Verde (Monte da Achada). Ref. **109**-3-i, 3♂♂ 1♀ 1j, 24/04/2012, Castro Verde (Monte da Achada). Ref. **109**-4-b, 2♀♀, 24/04/2012, Castro Verde (Monte da Achada). Ref. **109**-5-c, 2♂♂ 1♀ 4jj, 24/04/2012, Castro Verde (Monte da Achada). Ref. **109**-7-h, 1♀ 1j, 24/04/2012, Castro Verde (Monte da Achada). Ref. **109**-8-c, 1♂, 24/04/2012, Castro Verde (Monte da Achada). Ref. **109**-9-e, 1♀, 24/04/2012, Castro Verde (Monte da Achada). Ref. **110**-2-j, 1♂, 04/05/2012, Castro Verde (Monte da Perdigoa). Ref. **110**-3-k, 1♂ 2jj, 04/05/2012, Castro Verde (Monte da Perdigoa). Ref. **110**-4-d, 1♂ 1♀ 4jj, 04/05/2012, Castro Verde (Monte da Perdigoa). Ref. **110**-5-c, 2♂♂ 1♀ 2jj, 04/05/2012, Castro Verde (Monte da Perdigoa). Ref. **110**-6-a, 2♂♂ 3♀♀ 4jj,

04/05/2012, Castro Verde (Monte da Perdigoa). Ref. **110**-7-f, 2♂♂, 04/05/2012, Castro Verde (Monte da Perdigoa). Ref. **110**-8-e, 2♂♂ 3♀♀ 3jj, 04/05/2012, Castro Verde (Monte da Perdigoa). Ref. **110**-10-j, 1♀ 1j, 04/05/2012, Castro Verde (Monte da Perdigoa). Ref. **111**-3-g, 1♀ 1j, 16/04/2012, Castro Verde (Monte da Perdigoa). Ref. **111**-6-d, 1♀, 16/04/2012, Castro Verde (Monte da Perdigoa). Ref. **112**-2-h, 3jj, 20/04/2012, Castro Verde (Monte do Freire). Ref. **112**-7-h, 4jj, 20/04/2012, Castro Verde (Monte do Freire). Ref. **113**-3-c, 1♀ 5jj, 10/04/2012, Castro Verde (Courela do Monte Novo). Ref. **114**-1-c, 1♂ 5jj, 08/04/2012, Castro Verde (Herdade das Cuchilhas). Ref. **114**-2-i, 1♂ 4jj, 08/04/2012, Castro Verde (Herdade das Cuchilhas). Ref. **114**-4-a, 1♂ 6jj, 08/04/2012, Castro Verde (Herdade das Cuchilhas). Ref. **116**-7-d, 1♂ 1j, 10/04/2012, Castro Verde (Herdade dos Merendeiros). Ref. **121**-8-b, 1♂ 12jj, 16/04/2012, Castro Verde (Monte do Tacanho). Ref. **122**-2-d, 1♂ 3jj, 12/04/2012, Castro Verde (Herdade da Chaiça Velha). Ref. **122**-3-b, 1♀ 1j, 12/04/2012, Castro Verde (Herdade da Chaiça Velha). Ref. **122**-4-i, 1♀ 3jj, 12/04/2012, Castro Verde (Herdade da Chaiça Velha). Ref. **122**-7-c, 1♀ 3jj, 12/04/2012, Castro Verde (Herdade da Chaiça Velha). Ref. **122**-8-d, 1♂ 5jj, 12/04/2012, Castro Verde (Herdade da Chaiça Velha). Ref. **123**-2-c, 1♂ 1♀ 1j, 20/04/2012, Castro Verde (Herdade das bicadas). Ref. **123**-3-c, 1♂ 1♀, 20/04/2012, Castro Verde (Herdade das bicadas). Ref. **123**-4-g, 1♂ 7jj, 20/04/2012, Castro Verde (Herdade das bicadas). Ref. **123**-5-g, 3♂♂ 7jj, 20/04/2012, Castro Verde (Herdade das bicadas). Ref. **123**-6-c, 1♂ 12jj, 20/04/2012, Castro Verde (Herdade das bicadas). Ref. **123**-7-d, 1♂ 1♀ 8jj, 20/04/2012, Castro Verde (Herdade das bicadas). Ref. **123**-8-h, 1♂ 1♀ 5jj, 20/04/2012, Castro Verde (Herdade das bicadas). Ref. **123**-9-b, 1♀ 11jj, 20/04/2012, Castro Verde

(Herdade das bicadas). Ref. **123**-10-a, 1♂ 1♀ 6jj, 20/04/2012, Castro Verde (Herdade das bicadas). Ref. **123**-11-c, 1♂ 7jj, 20/04/2012, Castro Verde (Herdade das bicadas). Ref. **124**-5-a, 1♀ 4jj, 18/04/2012, Castro Verde (Herdade das Mestras). Ref. **125**-4-k, 1♂ 3jj, 17/04/2012, Aljustrel (Herdade da Sobreira). Ref. **125**-6-b, 1j, 17/04/2012, Aljustrel (Herdade da Sobreira). Ref. **126**-2-b, 1♀, 05/04/2012, Castro Verde (Monte do Seixo). Ref. **127**-8-b, 1♀ 7jj, 11/04/2012, Castro Verde (erdade dos Montinhos). Ref. **127**-9-f, 2♀♀ 12jj, 11/04/2012, Castro Verde (erdade dos Montinhos). Ref. **127**-11-e, 1♂ 1j, 11/04/2012, Castro Verde (erdade dos Montinhos). Ref. **128**-2-a, 1♂ 1j, 04/04/2012, Castro Verde (Herdade de Carriça-Viseus). Ref. **129**-2-k, 1♂ 4jj, 08/05/2012, Castro Verde (Herdade da Zibreira). Ref. **129**-3-b, 1♂ 2jj, 08/05/2012, Castro Verde (Herdade da Zibreira). Ref. **129**-5-d, 1♂ 1♀ 1j, 08/05/2012, Castro Verde (Herdade da Zibreira). Ref. **129**-9-b, 1♂, 08/05/2012, Castro Verde (Herdade da Zibreira). Ref. **131**-2-f, 2♂♂ 2♀♀ 2jj, 04/05/2012, Castro Verde (Herdade do Torrejão). Ref. **131**-4-c, 1♀ 1j, 04/05/2012, Castro Verde (Herdade do Torrejão). Ref. **131**-5-f, 2♀♀, 04/05/2012, Castro Verde (Herdade do Torrejão). Ref. **131**-6-b, 4♂♂ 1♀ 2jj, 04/05/2012, Castro Verde (Herdade do Torrejão). Ref. **131-**7-d, 1♂ 1♀ 3jj, 04/05/2012, Castro Verde (Herdade do Torrejão). Ref. **131**-8-a, 1♂ 1♀ 3jj, 04/05/2012, Castro Verde (Herdade do Torrejão). Ref. **131**-9-b, 1♀, 04/05/2012, Castro Verde (Herdade do Torrejão). Ref. **131**-11-c, 1♂, 04/05/2012, Castro Verde (Herdade do Torrejão). Ref. **132**-2-k, 2jj, 06/05/2012, Castro Verde (Monte das Cabeceiras). Ref. **132**-3-j, 1♂, 06/05/2012, Castro Verde (Monte das Cabeceiras). Ref. **132**-4-a, 1♀ 2jj, 06/05/2012, Castro Verde (Monte das Cabeceiras). Ref. **132-5-g**, 1♀ 5jj, 06/05/2012, Castro Verde (Monte das Cabeceiras). Ref. **132**-6-c, 2♂♂ 3♀♀, 06/05/2012, Castro Verde (Monte das Cabeceiras). Ref. **132**-7-b, 3♂♂ 2♀♀ 2jj,

06/05/2012, Castro Verde (Monte das Cabeceiras). Ref. **132**-8-a, 1♂ 1j, 06/05/2012, Castro Verde (Monte das Cabeceiras). Ref. **132**-9-a, 1♂ 1j, 06/05/2012, Castro Verde (Monte das Cabeceiras). Ref. **132**-11-b, 2♀♀ 5jj, 06/05/2012, Castro Verde (Monte das Cabeceiras). Ref. **133**-2-b, 1♂, 28/04/2012, Castro Verde (Monte da Albergaria). Ref. **133**-3-g, 1♂ 1j, 28/04/2012, Castro Verde (Monte da Albergaria). Ref. **133**-9-j, 1♂, 28/04/2012, Castro Verde (Monte da Albergaria). Ref. **133**-10-e, 1♂ 2♀♀, 28/04/2012, Castro Verde (Monte da Albergaria). Ref. **A46**-2-g, 2♀♀ 5jj, 10/05/2012, Aljustrel (Monte da Chaiça). Ref. **A46**-4-j, 1♂ 4jj, 10/05/2012, Aljustrel (Monte da Chaiça). Ref. **A46**-7-c6, 1♂ 2♀♀, 10/05/2012, Aljustrel (Monte da Chaiça). Ref. **A46**-8-c, 1♀ 3jj, 10/05/2012, Aljustrel (Monte da Chaiça). Ref. **A46**-9-f, 1♂ 2jj, 10/05/2012, Aljustrel (Monte da Chaiça). Ref. **A46**-10-g, 1♂ 3jj, 10/05/2012, Aljustrel (Monte da Chaiça). Ref. **A57**-2-a, 1♂ 6jj, 08/05/2012, Aljustrel (Herdade de Corta Rabos). Ref. **A57**-4-f, 1♀, 08/05/2012, Aljustrel (Herdade de Corta Rabos). Ref. **A57**-4-h, 2♀♀ 3jj, 08/05/2012, Aljustrel (Herdade de Corta Rabos). Ref. **A57**-5-d, 3♂♂ 1♀ 1j, 08/05/2012, Aljustrel (Herdade de Corta Rabos). Ref. **A57**-6-c, 1♂ 3♀♀ 3jj, 08/05/2012, Aljustrel (Herdade de Corta Rabos). Ref. **A57**-7-f, 1♂ 1♀ 1j, 08/05/2012, Aljustrel (Herdade de Corta Rabos). Ref. **A57**-8-a, 2♂♂ 2♀♀ 7jj, 08/05/2012, Aljustrel (Herdade de Corta Rabos). Ref. **A57**-9-i, 1♂ 2jj, 08/05/2012, Aljustrel (Herdade de Corta Rabos). Ref. **A57**-10-e, 3♀♀ 3jj, 08/05/2012, Aljustrel (Herdade de Corta Rabos). Ref. **A57**-11-g, 2♂♂ 5jj, 08/05/2012, Aljustrel (Herdade de Corta Rabos). Ref. **A166**-2-f, 1♀, 07/05/2012, Castro Verde (Herdade dos Brunhachos). Ref. **A166**-4-a, 2♂♂, 07/05/2012, Castro Verde (Herdade dos Brunhachos). Ref. **A166**-5-b, 1♂ 5jj, 07/05/2012, Castro Verde (Herdade dos Brunhachos). Ref. **A166**-6-b, 1♂, 07/05/2012, Castro Verde

(Herdade dos Brunhachos). Ref. **A166**-7-a, 1♂ 2♀♀ 3jj, 07/05/2012, Castro Verde (Herdade dos Brunhachos). Ref. **A166**-8-a, 1♂ 2jj, 07/05/2012, Castro Verde (Herdade dos Brunhachos). Ref. **A166**-9-c, 1♂ 1j, 07/05/2012, Castro Verde (Herdade dos Brunhachos). Ref. **A166**-10-b, 4♂♂ 1♀ 1j, 07/05/2012, Castro Verde (Herdade dos Brunhachos). Ref. **A166**-11-f, 2♀♀ 3jj, 07/05/2012, Castro Verde (Herdade dos Brunhachos). Ref. **A260**-2b, 1♀, 07/05/2012, Castro Verde (Herdade de Reidias). Ref. **A260**-5-g, 2jj, 07/05/2012, Castro Verde (Herdade de Reidias). Ref. **A260**-6-g, 1♀, 07/05/2012, Castro Verde (Herdade de Reidias). Ref. **A260**-9-d, 1♀ 3jj, 07/05/2012, Castro Verde (Herdade de Reidias). Ref. **A260**-10-c, 1♂ 1j, 07/05/2012, Castro Verde (Herdade de Reidias). Ref. **A260**-11-g, 1♂ 1j, 07/05/2012, Castro Verde (Herdade de Reidias). Ref. **A297**-2-b, 1j, 09/05/2012, Castro Verde (Lagoa da Mó). Ref. **A297**-3-b, 1♀ 1j, 09/05/2012, Castro Verde (Lagoa da Mó). Ref. **A297**-5-a, 1♂ 2♀♀ 1j, 09/05/2012, Castro Verde (Lagoa da Mó). Ref. **A297**-6-b, 1♂ 3jj, 09/05/2012, Castro Verde (Lagoa da Mó). Ref. **A297**-8-j, 1♂ 2♀♀, 09/05/2012, Castro Verde (Lagoa da Mó). Ref. **A297**-10-f, 1♂ 1♀ 1j, 09/05/2012, Castro Verde (Lagoa da Mó). Ref. **A297**-11-f, 1j, 09/05/2012, Castro Verde (Lagoa da Mó). Ref. **A299**-3-i, 1♀ 1j, 08/05/2012, Castro Verde (Lagoa da Mó). Ref. **A299**-4-d, 1♂ 3jj, 08/05/2012, Castro Verde (Lagoa da Mó). Ref. **A299**-7-d, 3♀♀ 3jj, 08/05/2012, Castro Verde (Lagoa da Mó). Ref. **A299**-8-j, 2♂♂, 08/05/2012, Castro Verde (Lagoa da Mó). Ref. **A299**-9-b, 1♀, 08/05/2012, Castro Verde (Lagoa da Mó). Ref. **A299**-10-f, 2♂♂ 1♀, 08/05/2012, Castro Verde (Lagoa da Mó). Ref. **A299**-10R-k, 2♂♂ 1♀ 2jj, 08/05/2012, Castro Verde (Lagoa da Mó). Ref. **A299**-11-d, 2♀♀ 1j, 08/05/2012, Castro Verde (Lagoa da Mó). Ref. **A299**-11R-b, 1♀ 4jj, 08/05/2012, Castro Verde (Lagoa da Mó). Ref. **A349**-2-f, 2♀♀ 2jj, 10/05/2012, Castro Verde

(Herdade das Mestras). Ref. **A349**-6-k, 1♂, 10/05/2012, Castro Verde (Herdade das Mestras). Ref. **A349**-8-h, 1♂ 3jj, 10/05/2012, Castro Verde (Herdade das Mestras). Ref. **A349**-9-g, 1♂ 1♀ 2jj, 10/05/2012, Castro Verde (Herdade das Mestras). Ref. **A349**-11-d, 1♂ 1♀ 3jj, 10/05/2012, Castro Verde (Herdade das Mestras). Ref. **A388**-3-c, 1♀ 1j, 10/05/2012, Castro Verde (Monte da Achada). Ref. **A388**-5-a, 2♀♀ 2jj, 10/05/2012, Castro Verde (Monte da Achada). Ref. **A388**-6-e, 1♀ 1j, 10/05/2012, Castro Verde (Monte da Achada). Ref. **A388**-8-a, 1♀ 1j, 10/05/2012, Castro Verde (Monte da Achada). Ref. **A388**-10-f, 3♂♂ 1♀, 10/05/2012, Castro Verde (Monte da Achada). Ref. **A423**-2-j, 1♀ 2jj, 08/05/2012, Castro Verde (Herdade da Zibreira). Ref. **A423**-7-d, 1j, 08/05/2012, Castro Verde (Herdade da Zibreira). Ref. **A423**-9-a, 2jj, 08/05/2012, Castro Verde (Herdade da Zibreira). Ref. **A423**-10-d, 2♀♀ 1j, 08/05/2012, Castro Verde (Herdade da Zibreira). Ref. **A482**-2-c, 1♂ 1♀ 2jj, 09/05/2012, Castro Verde (Amendoeira Nova). Ref. **A482**-3-b, 1♂ 1♀ 1j, 09/05/2012, Castro Verde (Amendoeira Nova). Ref. **A482**-4-f, 1♂ 3jj, 09/05/2012, Castro Verde (Amendoeira Nova). Ref. **A482**-7-e, 1♀ 2jj, 09/05/2012, Castro Verde (Amendoeira Nova). Ref. **A482**-10-b, 1♀, 09/05/2012, Castro Verde (Amendoeira Nova). Ref. **A505**-2-c, 1♀ 4jj, 08/05/2012, Castro Verde (Herdade do Reguengo). Ref. **A505**-4-a, 1♀ 4jj, 08/05/2012, Castro Verde (Herdade do Reguengo). Ref. **A505**-7-f, 2♂♂ 1♀, 08/05/2012, Castro Verde (Herdade do Reguengo). Ref. **A505**-8-c, 1♂ 1j, 08/05/2012, Castro Verde (Herdade do Reguengo). Ref. **A527**-3-g, 2♀♀, 07/05/2012, Castro Verde (Herdade dos Bispos). Ref. **A527**-4-k, 2♂♂, 07/05/2012, Castro Verde (Herdade dos Bispos). Ref. **A527**-5-d, 1♀ 3jj, 07/05/2012, Castro Verde (Herdade dos Bispos). Ref. **A527**-6-f, 1♀, 07/05/2012, Castro Verde (Herdade dos Bispos). Ref. **A527**-7-c, 2♂♂, 07/05/2012, Castro Verde (Herdade dos Bispos). Ref. **A527**-8-

g, 2♂♂ 3♀♀ 2jj, 07/05/2012, Castro Verde (Herdade dos Bispos). Ref. **A527**-9-e, 1♀, 07/05/2012, Castro Verde (Herdade dos Bispos). Ref. **A527**-11-g, 1♀, 07/05/2012, Castro Verde (Herdade dos Bispos). Ref. **A999**-2-j, 2♀♀ 3jj, 09/05/2012, Castro Verde (Herdade dos Pereiros). Ref. **A999**-5-d, 1♂ 3♀♀ 1j, 09/05/2012, Castro Verde (Herdade dos Pereiros). Ref. **A999**-7-g, 1♂, 09/05/2012, Castro Verde (Herdade dos Pereiros). Ref. **A999**-8-j, 1♂ 1♀ 1j, 09/05/2012, Castro Verde (Herdade dos Pereiros). Ref. **A999**-9-g, 2♀♀ 1j, 09/05/2012, Castro Verde (Herdade dos Pereiros). Ref. **A999**-10-b, 1♀ 2jj, 09/05/2012, Castro Verde (Herdade dos Pereiros). Ref. **A999**-11-h, 1♀, 09/05/2012, Castro Verde (Herdade dos Pereiros).

***Centromerus phoceorum* Simon, 1929**

Material examined: Ref. **72**-8-e, 1♀, 15/04/2012, Castro Verde (Cumeada Nova). Ref. **123**-11-c, 1♀, 20/04/2012, Castro Verde (Herdade das bicadas).

***Diplocephalus graecus* (O.P.-Cambridge, 1873)**

Material examined: Ref. **4**-2-c, 2♂♂, 15/04/2012, Castro Verde (Heredade das Mouras). Ref. **4**-5-j, 1♂, 15/04/2012, Castro Verde (Heredade das Mouras). Ref. **4**-6-c, 1♂, 15/04/2012, Castro Verde (Heredade das Mouras). Ref. **4**-8-h, 1♂ 2♀♀ 18jj, 15/04/2012, Castro Verde (Heredade das Mouras). Ref. **4**-9-a, 1♂, 15/04/2012, Castro Verde (Heredade das Mouras). Ref. **4**-10-e, 1♂ 1♀, 15/04/2012, Castro Verde (Heredade das Mouras). Ref. **4**-11-b, 1♂, 15/04/2012, Castro Verde (Heredade das Mouras). Ref. **6**-2-a, 1♀ 1j, 24/04/2012, Castro Verde (Herdade de A de Neves da Marinha). Ref. **6**-9-h, 1♂, 24/04/2012, Castro Verde (Herdade de A de Neves da Marinha). Ref. **9**-2-g, 1♀ 3jj, 25/04/2012,

Castro Verde (COSA - Herdade de São Marcos). Ref. **9**-7-i, 1♂, 25/04/2012, Castro Verde (COSA - Herdade de São Marcos). Ref. **9**-10-c, 1♀, 25/04/2012, Castro Verde (COSA - Herdade de São Marcos). Ref. **11**-7-c, 1♂, 15/04/2012, Castro Verde (Monte do Broco e Capitão). Ref. **11**-8-f, 1♂, 15/04/2012, Castro Verde (Monte do Broco e Capitão). Ref. **11**-10-f, 1♀, 15/04/2012, Castro Verde (Monte do Broco e Capitão). Ref. **14**-3-d, 1♀ 1j, 18/04/2012, Castro Verde (Lagoa da Mó). Ref. **14**-5-b, 1♂ 1♀ 4jj, 18/04/2012, Castro Verde (Lagoa da Mó). Ref. **14**-10-j, 4jj, 18/04/2012, Castro Verde (Lagoa da Mó). Ref. **14**-11-c, 1♂, 18/04/2012, Castro Verde (Lagoa da Mó). Ref. **19**-5-c, 3♂♂ 1♀, 26/04/2012, Castro Verde (Herdade do Torrejão). Ref. **23**-4-a, 1♂ 1j, 03/04/2012, Castro Verde (Monte do Vale das Gretas). Ref. **25**-5-a, 1♂ 10jj, 05/04/2012, Castro Verde (Herdade dos Touris e Rolão). Ref. **27**-9-e, 1♂ 1♀ 2jj, 24/04/2012, Castro Verde (Herdade das Sesmarias). Ref. **34**-3-c, 1♂ 3jj, 16/04/2012, Castro Verde (Herdade do Tacanho e Merendeiros). Ref. **35**-6-g, 1♂, 05/04/2012, Castro Verde (Monte do Seixo). Ref. **35**-8-d, 1♂, 05/04/2012, Castro Verde (Monte do Seixo). Ref. **35**-10-f, 1♂ 2jj, 05/04/2012, Castro Verde (Monte do Seixo). Ref. **37**-5-b, 1♀ 1j, 09/04/2012, Castro Verde (Herdade de São Marcos). Ref. **37**-7-d, 1♂, 09/04/2012, Castro Verde (Herdade de São Marcos). Ref. **43**-2-d, 1♂ 2jj, 21/04/2012, Castro Verde (Monte da Comenda). Ref. **43-**11-h, 1♀ 1j, 21/04/2012, Castro Verde (Monte da Comenda). Ref. **47**-9-i, 1♂ 1j, 09/04/2012, Castro Verde (Herdade do Almarginho). Ref. **49**-3-e, 1♂, 25/04/2012, Castro Verde (Herdade da Navarra). Ref. **51**-7-e, 1♀, 13/04/2012, Castro Verde (Herdade do Torrejão). Ref. **54**-10a-d, 1♀, 25/04/2012, Castro Verde (Herdade do Roncanho). Ref. **55**-2-c, 1♂, 21/04/2012, Castro Verde (Herdade da Barrigoa). Ref. **55**-4-d, 1♀ 1j, 21/04/2012, Castro Verde (Herdade da Barrigoa). Ref. **56**-2-h, 1♀ 4jj, 18/04/2012,

Castro Verde (Herdade da Barrigoa). Ref. **58**-1-h, 1♀, 07/04/2012, Mértola (Herdade da Benviúda). Ref. **58**-4-b, 1♂ 1j, 07/04/2012, Mértola (Herdade da Benviúda). Ref. **61**-3-c, 1♀, 05/05/2012, Aljustrel (Monte da Torre). Ref. **61**-6-j, 1♂, 05/05/2012, Aljustrel (Monte da Torre). Ref. **61**-11-e, 1♂, 05/05/2012, Aljustrel (Monte da Torre). Ref. **67**-3-c, 2♂♂ 5jj, 12/04/2012, Aljustrel (Monte da Chaiça). Ref. **70**-9-f, 1♂, 21/04/2012, Castro Verde (Monte dos Janeiros). Ref. **70**-10-a, 1♂ 2jj, 21/04/2012, Castro Verde (Monte dos Janeiros). Ref. **79**-3-h, 1♀ 4jj, 07/04/2012, Castro Verde (Monte da Chada). Ref. **79**-7-g, 1♀ 1j, 07/04/2012, Castro Verde (Monte da Chada). Ref. **86**-7-b, 1♀ 2jj, 10/05/2012, Castro Verde (Herdade das Mestras). Ref. **89**-11-h, 1♀, 06/05/2012, Castro Verde (Herdade do Carapetal). Ref. **92**-8-e, 1♂, 17/04/2012, Aljustrel (Monte do Carregueiro). Ref. **92**-9-f, 1♀ 3jj, 17/04/2012, Aljustrel (Monte do Carregueiro). Ref. **92**-10-b, 1♀ 1j, 17/04/2012, Aljustrel (Monte do Carregueiro). **93**-5-e, 1♀ 4jj, 17/04/2012, Aljustrel (Herdade da Sobreira). Ref. **104**-1-b, 1♀ 2jj, 04/04/2012, Castro Verde (Herdade dos Touris e Rolão). Ref. **104**-3-b, 2♀♀ 1j, 04/04/2012, Castro Verde (Herdade dos Touris e Rolão). Ref. **104**-5-a, 1♂ 3jj, 04/04/2012, Castro Verde (Herdade dos Touris e Rolão). Ref. **104**-6-c, 2♀♀, 04/04/2012, Castro Verde (Herdade dos Touris e Rolão). Ref. **104**-11-c, 1♂, 04/04/2012, Castro Verde (Herdade dos Touris e Rolão). Ref. **107**-2-j, 1♂, 18/04/2012, Castro Verde (Herdade das Mestras). Ref. **107**-4-g, 1♀, 18/04/2012, Castro Verde (Herdade das Mestras). Ref. **107**-5-e, 1♂ 1j, 18/04/2012, Castro Verde (Herdade das Mestras). Ref. **107**-6-d, 1♂, 18/04/2012, Castro Verde (Herdade das Mestras). Ref. **107**-8-b, 1♂, 18/04/2012, Castro Verde (Herdade das Mestras). Ref. **107**-11-f, 1♀ 2jj, 18/04/2012, Castro Verde (Herdade das Mestras). Ref. **109**-3-i, 1♀, 24/04/2012, Castro Verde (Monte da Achada). Ref. **109**-8-c, 1♀, 24/04/2012, Castro Verde

(Monte da Achada). Ref. **114**-4-a, 1♂, 08/04/2012, Castro Verde (Herdade das Cuchilhas). Ref. **114**-10-a, 1♂ 3jj, 08/04/2012, Castro Verde (Herdade das Cuchilhas). Ref. **114**-12-d, 1♀ 4jj, 08/04/2012, Castro Verde (Herdade das Cuchilhas). Ref. **122**-4-i, 2♀♀, 12/04/2012, Castro Verde (Herdade da Chaiça Velha). Ref. **122**-5-f, 1♀ 1j, 12/04/2012, Castro Verde (Herdade da Chaiça Velha). Ref. **122**-7-c, 1♂ 2jj, 12/04/2012, Castro Verde (Herdade da Chaiça Velha). Ref. **122**-9-b, 1♂ 2jj, 12/04/2012, Castro Verde (Herdade da Chaiça Velha). Ref. **122**-11-a, 1♀, 12/04/2012, Castro Verde (Herdade da Chaiça Velha). Ref. **123**-3-c, 1♀, 20/04/2012, Castro Verde (Herdade das bicadas). Ref. **123**-5-g, 1♂ 1♀, 20/04/2012, Castro Verde (Herdade das bicadas). Ref. **123**-7-d, 2♂♂, 20/04/2012, Castro Verde (Herdade das bicadas). Ref. **123**-9-b, 1♂ 1♀, 20/04/2012, Castro Verde (Herdade das bicadas). Ref. **123**-11-c, 1♀, 20/04/2012, Castro Verde (Herdade das bicadas). Ref. **124**-8-d, 1♂ 1♀ 2jj, 18/04/2012, Castro Verde (Herdade das Mestras). Ref. **127**-8-b, 131-3-c, 11/04/2012, Castro Verde (Herdade dos Montinhos). Ref. **128**-6-e, 1♂, 04/04/2012, Castro Verde (Herdade de Carriça-Viseus). Ref. **128**-9-a, 1♂, 04/04/2012, Castro Verde (Herdade de Carriça-Viseus). Ref. **131**-3-c, 1♀ 2jj, 04/05/2012, Castro Verde (Herdade do Torrejão). Ref. **131**-11-c, 1♀ 2jj, 04/05/2012, Castro Verde (Herdade do Torrejão). Ref. **133**-10-e, 1♂, 28/04/2012, Castro Verde (Monte da Albergaria). Ref. **A57**-6-c, 1♂, 08/05/2012, Aljustrel (Herdade de Corta Rabos). Ref. **A57**-8-a, 2♂♂ 2♀♀, 08/05/2012, Aljustrel (Herdade de Corta Rabos). Ref. **A57**-11-g, 1♂, 08/05/2012, Aljustrel (Herdade de Corta Rabos). Ref. **A260**-11-g, 1♂, 07/05/2012, Castro Verde (Herdade de Reidias). Ref. **A423**-11-f, 1♂, 08/05/2012, Castro Verde (Herdade da Zibreira). Ref. **A482**-2-c, 1♀, 09/05/2012, Castro Verde (Amendoeira Nova).

***Diplocephalus marijae* Bosmans, 2010**

Material examined: Ref. **92**-6-h, 1♀, 17/04/2012, Aljustrel (Monte do Carregueiro). Ref. **97**-8-c, 1♂ 1♀, 13/04/2012, Castro Verde (Cumeada Nova). Ref. **97**-10-i, 1♂, 13/04/2012, Castro Verde (Cumeada Nova).

***Erigone dentipalpis* (Wider, 1834)**

Material examined: Ref. **A388**-6-e, 1♂, 10/05/2012, Castro Verde (Monte da Achada).

***Microctenonyx subitaneus* (O.P.-Cambridge, 1875)**

Material examined: Ref. **67**-8-d, 1♀, 12/04/2012, Aljustrel (Monte da Chaiça). Ref. **73**-3-h, 1j, 19/04/2012, Castro Verde (Herdade dos Longos). Ref. **104**-3-b, 1♀, 04/04/2012, Castro Verde (Herdade dos Touris e Rolão). Ref. **110**-9-f, 1♀ 2jj, 04/05/2012, Castro Verde (Monte da Perdigoa). Ref. **111**-10-c, 1♂, 16/04/2012, Castro Verde (Monte da Perdigoa). Ref. **121**-2-d, 1♂, 16/04/2012, Castro Verde (Monte do Tacanho). Ref. **A297**-2-b, 1j, 09/05/2012, Castro Verde (Lagoa da Mó).

***Oedothorax fuscus* (Blackwall, 1834)**

Material examined: Ref. **87**-2-c, 1♂ 2♀♀, 06/05/2012, Castro Verde (Herdade das Mestras). Ref. **A423**-9-a, 1♂, 08/05/2012, Castro Verde (Herdade da Zibreira).

***Ouedia rufithorax* (Simon, 1881)**

Material examined: Ref. **36**-3-a, 1♀, 11/04/2012, Castro Verde (Herdade dos Bispos). Ref. **125**-4-k, 1♀, 17/04/2012, Aljustrel (Herdade da Sobreira). Ref. **125**-7-f, 1♂ 1♀, 17/04/2012, Aljustrel (Herdade da Sobreira).

***Pallidiphantes stygius* (Simon, 1884)**

Material examined: Ref. **70**-10-a, 1♂, 21/04/2012, Castro Verde (Monte dos Janeiros).

***Pelecopsis bucephala* (O.P.-Cambridge, 1875)**

Material examined: Ref. **70**-6-e, 2♀♀ 1j, 21/04/2012, Castro Verde (Monte dos Janeiros).

***Pelecopsis inedita* (O.P.-Cambridge, 1875)**

Material examined: Ref. **6**-9-h, 2jj, 24/04/2012, Castro Verde (Herdade de A de Neves da Marinha). Ref. **9**-2-g, 1♂, 25/04/2012, Castro Verde (COSA - Herdade de São Marcos). Ref. **9**-5-a, 4jj, 25/04/2012, Castro Verde (COSA - Herdade de São Marcos). Ref. **9**-6-g, 2jj, 25/04/2012, Castro Verde (COSA - Herdade de São Marcos). Ref. **9**-9-d, 2jj, 25/04/2012, Castro Verde (COSA - Herdade de São Marcos). Ref. **14**-11-c, 1♀, 18/04/2012, Castro Verde (Lagoa da Mó). Ref. **15**-3-b, 1♂, 12/04/2012, Castro Verde (Lagoa da Mó). Ref. **21**-2-a, 1j, 21/04/2012, Castro Verde (Herdade da Barrigoa). Ref. **21**-10-a, 1♀, 21/04/2012, Castro Verde (Herdade da Barrigoa). Ref. **23**-4-a, 1♂, 03/04/2012, Castro Verde (Monte do Vale das Gretas). Ref. **23**-7-c, 1♂, 03/04/2012, Castro Verde (Monte do Vale das Gretas). Ref. **47**-3-d, 1♀, 09/04/2012, Castro Verde (Herdade do Almarginho). Ref. **47**-4-g, 1♀, 09/04/2012, Castro Verde (Herdade do Almarginho). Ref. **61**-

10-h, 1♂, 05/05/2012, Aljustrel (Monte da Torre). Ref. **61**-11-e, 1♂, 05/05/2012, Aljustrel (Monte da Torre). Ref. **67**-8-d, 1♂, 12/04/2012, Aljustrel (Monte da Chaiça). Ref. **67**-9-c, 1♂, 12/04/2012, Aljustrel (Monte da Chaiça). Ref. **78**-1-b, 1♂, 09/04/2012, Castro Verde (Herdade de São Marcos). Ref. **78**-5-b, 1♂, 09/04/2012, Castro Verde (Herdade de São Marcos). Ref. **78**-10-c, 1j, 09/04/2012, Castro Verde (Herdade de São Marcos). Ref. **83**-4-i, 1j, 06/05/2012, Castro Verde (Herdade do Álamo). Ref. **83**-7-h, 1j, 06/05/2012, Castro Verde (Herdade do Álamo). Ref. **83**-11-a, 1♂, 06/05/2012, Castro Verde (Herdade do Álamo). Ref. **86**-3-d, 1♀, 10/05/2012, Castro Verde (Herdade das Mestras). Ref. **86**-6-g, 1♂, 10/05/2012, Castro Verde (Herdade das Mestras). Ref. **86**-7-b, 1♀ 1j, 10/05/2012, Castro Verde (Herdade das Mestras). Ref. **86**-9-h, 1♂ 6jj, 10/05/2012, Castro Verde (Herdade das Mestras). Ref. **87**-7-g, 1♂ 3jj, 06/05/2012, Castro Verde (Herdade das Mestras). Ref. **88**-5-i, 2jj, 25/04/2012, Castro Verde (Herdade da Portela). Ref. **88**-6-c, 1♀, 25/04/2012, Castro Verde (Herdade da Portela). Ref. **88**-7-e, 1♂, 25/04/2012, Castro Verde (Herdade da Portela). Ref. **88**-11-j, 1♂ 1♀, 25/04/2012, Castro Verde (Herdade da Portela). Ref. **109**-2-e, 1j, 24/04/2012, Castro Verde (Monte da Achada). Ref. **109**-6-f, 1♀ 3jj, 24/04/2012, Castro Verde (Monte da Achada). Ref. **109**-7-h, 1♂, 24/04/2012, Castro Verde (Monte da Achada). Ref. **111**-6-d, 1♂ 1j, 16/04/2012, Castro Verde (Monte da Perdigoa). Ref. **112**-8-i, 1♂ 1j, 20/04/2012, Castro Verde (Monte do Freire). Ref. **121**-4-b, 1♂ 2jj, 16/04/2012, Castro Verde (Monte do Tacanho). Ref. **124**-2-b, 1♀, 18/04/2012, Castro Verde (Herdade das Mestras). Ref. **124**-7-i, 1♀ 1j, 18/04/2012, Castro Verde (Herdade das Mestras). Ref. **124**-10-b, 1j, 18/04/2012, Castro Verde (Herdade das Mestras). Ref. **126**-4-c, 1♀, 05/04/2012, Castro Verde (Monte do Seixo). Ref. **126**-6-a, 1j, 05/04/2012, Castro Verde

(Monte do Seixo). Ref. **128**-2-a, 1♀, 04/04/2012, Castro Verde (Herdade de Carriça-Viseus). Ref. **A166**-3-g, 1♀, 07/05/2012, Castro Verde (Herdade dos Brunhachos). Ref. **A166**-4-a, 1♂, 07/05/2012, Castro Verde (Herdade dos Brunhachos). Ref. **A166**-6-b, 2♂♂ 1♀ 4jj, 07/05/2012, Castro Verde (Herdade dos Brunhachos). Ref. **A260**-2b, 1♂, 07/05/2012, Castro Verde (Herdade de Reidias). Ref. **A260**-7-d, 1♀ 1j, 07/05/2012, Castro Verde (Herdade de Reidias). Ref. **A260**-10-c, 1♀, 07/05/2012, Castro Verde (Herdade de Reidias). Ref. **A297**-2-b, 2♂♂ 1♀ 2jj, 09/05/2012, Castro Verde (Lagoa da Mó). Ref. **A297**-8-j, 2♂♂ 2♀♀, 09/05/2012, Castro Verde (Lagoa da Mó). Ref. **A349**-6-k, 2♀♀ 2jj, 10/05/2012, Castro Verde (Herdade das Mestras). Ref. **A388**-4-b, 2♀♀, 10/05/2012, Castro Verde (Monte da Achada). Ref. **A388**-11-a, 1♀ 1j, 10/05/2012, Castro Verde (Monte da Achada). Ref. **A482**-4-f, 1♂, 09/05/2012, Castro Verde (Amendoeira Nova). Ref. **A527**-11-g, 1♂, 07/05/2012, Castro Verde (Herdade dos Bispos).

Prinerigone vagans **(Audouin, 1826)**

Material examined: Ref. **125**-5-e, 1♀, 17/04/2012, Aljustrel (Herdade da Sobreira). Ref. **125**-10-k, 1♀, 17/04/2012, Aljustrel (Herdade da Sobreira). Ref. **A57**-3-h, 1♂, 08/05/2012, Aljustrel (Herdade de Corta Rabos). Ref. **A57**-6-c, 1♂, 08/05/2012, Aljustrel (Herdade de Corta Rabos). Ref. **A527**-7-c, 1♂, 07/05/2012, Castro Verde (Herdade dos Bispos).

Styloctetor romanus **(O.P.-Cambridge, 1873)**

Material examined: Ref. **111**-9-g, 1♀ 1j, 16/04/2012, Castro Verde (Monte da Perdigoa).

***Tapinocyba algirica* Bosmans, 2007**

Material examined: Ref. **35**-7-d, 1♂, 05/04/2012, Castro Verde (Monte do Seixo). Ref. **49**-9-a, 1♂, 25/04/2012, Castro Verde (Herdade da Navarra). Ref. **51**-5-b, 1♂, 13/04/2012, Castro Verde (Herdade do Torrejão). Ref. **61**-5-h, 2♂♂, 05/05/2012, Aljustrel (Monte da Torre). Ref. **73**-8-c, 1♂ 1j, 19/04/2012, Castro Verde (Herdade dos Longos). Ref. **A260**-8-e, 1♂ 1♀, 07/05/2012, Castro Verde (Herdade de Reidias). Ref. **A260**-9-d, 1♂, 07/05/2012, Castro Verde (Herdade de Reidias). Ref. **A260**-10-c, 1♀, 07/05/2012, Castro Verde (Herdade de Reidias). Ref. **A297**-3-b, 1j, 09/05/2012, Castro Verde (Lagoa da Mó).

***Tenuiphantes* sp.**

Material examined: Ref. **4**-2-c, 1j, 15/04/2012, Castro Verde (Heredade das Mouras). Ref. **49**-4-b, 1j, 25/04/2012, Castro Verde (Herdade da Navarra). Ref. **50**-3-f, 1j, 16/04/2012, Castro Verde (Herdade do Torrejão). Ref. **A166**-9-c, 1j, 07/05/2012, Castro Verde (Herdade dos Brunhachos). Ref. **A297**-10-f, 1j, 09/05/2012, Castro Verde (Lagoa da Mó). Ref. **A999**-11-h, 2jj, 09/05/2012, Castro Verde (Herdade dos Pereiros).

***Walckenaeria cucullata* (C. L. Koch, 1836)**

Material examined: Ref. **131**-11-c, 1♂, 04/05/2012, Castro Verde (Herdade do Torrejão).

Family LIOCRANIDAE Simon, 1897

***Agraecina lineata* (Simon, 1878)**

Material examined: Ref. **87**-2-c, 1♂, 06/05/2012, Castro Verde (Herdade das Mestras).

Family LYCOSIDAE Sundevall, 1833

Lycosidae sp.; (189 jj. indet.).

***Alopecosa albofasciata* (Brullé, 1832)**

Material examined: Ref. **11**-4-b, 1♂, 15/04/2012, Castro Verde (Monte do Broco e Capitão). Ref. **36**-6-h, 1♂ 1j, 11/04/2012, Castro Verde (Herdade dos Bispos). Ref. **113**-11-c, 1j, 10/04/2012, Castro Verde (Courela do Monte Novo). Ref. **121**-8-b, 1♂, 16/04/2012, Castro Verde (Monte do Tacanho).

***Hogna radiata* (Latreille, 1817)**

Material examined: Ref. **21**-9-a, 3jj, 21/04/2012, Castro Verde (Herdade da Barrigoa). Ref. **25**-8-a, 1j, 05/04/2012, Castro Verde (Herdade dos Touris e Rolão). Ref. **51**-8-c, 1j, 13/04/2012, Castro Verde (Herdade do Torrejão). Ref. **70**-11-g, 3jj, 21/04/2012, Castro Verde (Monte dos Janeiros). Ref. **112**-11-d, 1j, 20/04/2012, Castro Verde (Monte do Freire). Ref. **116**-5-d, 1j, 10/04/2012, Castro Verde (Herdade dos Merendeiros). Ref. **122**-11-a, 1j, 12/04/2012, Castro Verde (Herdade da Chaiça Velha). Ref. **124**-5-a, 1j, 18/04/2012, Castro Verde (Herdade das Mestras). Ref. **124**-8-d, 1j, 18/04/2012, Castro Verde (Herdade das Mestras). Ref. **127**-4-a, 1j, 11/04/2012, Castro Verde (Herdade dos Montinhos). Ref. **A423**-

8-a, 1j, 08/05/2012, Castro Verde (Herdade da Zibreira). Ref. **A423**-10-d, 1j, 08/05/2012, Castro Verde (Herdade da Zibreira).

Pardosa proxima **(C. L. Koch, 1847)**

Material examined: Ref. **34**-3-c, 1♀, 16/04/2012, Castro Verde (Herdade do Tacanho e Merendeiros). Ref. **88**-9-g, 1j, 25/04/2012, Castro Verde (Herdade da Portela). Ref. **107**-4-g, 1j, 18/04/2012, Castro Verde (Herdade das Mestras). Ref. **108**-7-g, 1♀, 10/04/2012, Castro Verde (Herdade dos Merendeiros). Ref. **109**-9-e, 1♀, 24/04/2012, Castro Verde (Monte da Achada). Ref. **110**-2-j, 1j, 04/05/2012, Castro Verde (Monte da Perdigoa). Ref. **110**-3-k, 1j, 04/05/2012, Castro Verde (Monte da Perdigoa). Ref. **111**-10-c, 1j, 16/04/2012, Castro Verde (Monte da Perdigoa). Ref. **112**-3-b, 1♀ 1j, 20/04/2012, Castro Verde (Monte do Freire). Ref. **112**-6-g, 2jj, 20/04/2012, Castro Verde (Monte do Freire). Ref. **113**-10-c, 1j, 10/04/2012, Castro Verde (Courela do Monte Novo). Ref. **121**-2-d, 1j, 16/04/2012, Castro Verde (Monte do Tacanho). Ref. **129**-9-b, 1j, 08/05/2012, Castro Verde (Herdade da Zibreira). Ref. **132**-7-b, 2jj, 06/05/2012, Castro Verde (Monte das Cabeceiras). Ref. **A57**-4-h 1♀ 1j, 08/05/2012, Aljustrel (Herdade de Corta Rabos). Ref. **A297**-10-f, 1j, 09/05/2012, Castro Verde (Lagoa da Mó).

Family MIMETIDAE Simon, 1881

Ero aphana **(Walckenaer, 1802)**

Material examined: Ref. **6**-7-f, 1♂, 24/04/2012, Castro Verde (Herdade de A de Neves da Marinha). Ref. **6**-11-b, 1♂, 24/04/2012, Castro Verde (Herdade de A de Neves da Marinha). Ref. **50**-11-f, 1♂, 16/04/2012, Castro Verde (Herdade do

Torrejão). Ref. **61**-3-c, 1♀, 05/05/2012, Aljustrel (Monte da Torre). Ref. **A166**-9-c, 1♂, 07/05/2012, Castro Verde (Herdade dos Brunhachos). Ref. **A299**-3-i, 1♀, 08/05/2012, Castro Verde (Lagoa da Mó).

Family MITURGIDAE Simon, 1886

***Zora silvestris* Kulczyński, 1897**

Material examined: Ref. **43**-7-b, 1♀, 21/04/2012, Castro Verde (Monte da Comenda). Ref. **A999**-11-h, 1♀, 09/05/2012, Castro Verde (Herdade dos Pereiros).

Family NEMESIIDAE Simon, 1889

***Iberesia* sp.**

Material examined: Ref. **133**-5-c, 1j, 28/04/2012, Castro Verde (Monte da Albergaria).

Family OONOPIDAE Simon, 1890

***Oonops tubulatus* Dalmas, 1916**

Material examined: Ref. **43**-9-d, 1♀, 21/04/2012, Castro Verde (Monte da Comenda). Ref. **70**-11-g, 1♀, 21/04/2012, Castro Verde (Monte dos Janeiros). Ref. **121**-10-d, 1♀, 16/04/2012, Castro Verde (Monte do Tacanho). Ref. **127**-11-e, 1♀, 11/04/2012, Castro Verde (Herdade dos Montinhos).

Silhouettella loricatula **(Roewer, 1942)**

Material examined: Ref. **57**-11-i, 1♂, 06/04/2012, Castro Verde (Monte da Azinheira). Ref. **A423**-4-i, 2♂♂, 08/05/2012, Castro Verde (Herdade da Zibreira).

Family OXYOPIDAE Thorell, 1870

Oxyopes heterophthalmus **(Latreille, 1804)**

Material examined: Ref. **4**-6-c, 1j, 15/04/2012, Castro Verde (Heredade das Mouras). Ref. **4**-11-b, 1j, 15/04/2012, Castro Verde (Heredade das Mouras). Ref. **6**-5-c, 1j, 24/04/2012, Castro Verde (Herdade de A de Neves da Marinha). Ref. **6**-6-a, 1♀ 1j, 24/04/2012, Castro Verde (Herdade de A de Neves da Marinha). Ref. **6**-7-f, 2jj, 24/04/2012, Castro Verde (Herdade de A de Neves da Marinha). Ref. **6**-9-h, 1j, 24/04/2012, Castro Verde (Herdade de A de Neves da Marinha). Ref. **6**-10-e, 1j, 24/04/2012, Castro Verde (Herdade de A de Neves da Marinha). Ref. **9**-6-g, 1j, 25/04/2012, Castro Verde (COSA - Herdade de São Marcos). Ref. **11**-5-h, 1j, 15/04/2012, Castro Verde (Monte do Broco e Capitão). Ref. **15**-5-b, 1j, 12/04/2012, Castro Verde (Lagoa da Mó). Ref. **15**-9-a, 1j, 12/04/2012, Castro Verde (Lagoa da Mó). Ref. **19**-6-a, 1j, 26/04/2012, Castro Verde (Herdade do Torrejão). Ref. **21**-6-a, 1♂, 21/04/2012, Castro Verde (Herdade da Barrigoa). Ref. **23**-2-a, 1j, 03/04/2012, Castro Verde (Monte do Vale das Gretas). Ref. **23**-5-i, 4jj, 03/04/2012, Castro Verde (Monte do Vale das Gretas). Ref. **23**-7-c, 2jj, 03/04/2012, Castro Verde (Monte do Vale das Gretas). Ref. **23**-9-a, 1j, 03/04/2012, Castro Verde (Monte do Vale das Gretas). Ref. **23**-11-i, 1j, 03/04/2012, Castro Verde (Monte do Vale das Gretas). Ref. **23**-12-b, 3jj, 03/04/2012, Castro Verde (Monte do Vale das Gretas). Ref. **25**-1-c, 1j,

05/04/2012, Castro Verde (Herdade dos Touris e Rolão). Ref. **25**-11-a, 1j, 05/04/2012, Castro Verde (Herdade dos Touris e Rolão). Ref. **27**-2-c, 2jj, 24/04/2012, Castro Verde (Herdade das Sesmarias). Ref. **27**-8-c, 1j, 24/04/2012, Castro Verde (Herdade das Sesmarias). Ref. **34**-4-a, 1j, 16/04/2012, Castro Verde (Herdade do Tacanho e Merendeiros). Ref. **35**-5-f, 1j, 05/04/2012, Castro Verde (Monte do Seixo). Ref. **36**-3-a, 4jj, 11/04/2012, Castro Verde (Herdade dos Bispos). Ref. **37**-3-a, 1j, 09/04/2012, Castro Verde (Herdade de São Marcos). Ref. **37**-12-d, 1j, 09/04/2012, Castro Verde (Herdade de São Marcos). Ref. **43**-4-d, 2jj, 21/04/2012, Castro Verde (Monte da Comenda). Ref. **45**-2-c, 1j, 13/04/2012, Castro Verde (Monte das Fontes Barbas Velho). Ref. **47**-1-a, 1j, 09/04/2012, Castro Verde (Herdade do Almarginho). Ref. **47**-6-c, 2jj, 09/04/2012, Castro Verde (Herdade do Almarginho). Ref. **49**-2-d, 1j, 25/04/2012, Castro Verde (Herdade da Navarra). Ref. **49**-3-e, 1j, 25/04/2012, Castro Verde (Herdade da Navarra). Ref. **49**-5-a, 3jj, 25/04/2012, Castro Verde (Herdade da Navarra). Ref. **53**-4-b, 1j, 20/04/2012, Castro Verde (Herdade do Reguengo). Ref. **53**-6-d, 1j, 20/04/2012, Castro Verde (Herdade do Reguengo). Ref. **53**-8-c, 1j, 20/04/2012, Castro Verde (Herdade do Reguengo). Ref. **53**-9-b, 3jj, 20/04/2012, Castro Verde (Herdade do Reguengo). Ref. **53**-11-e, 2jj, 20/04/2012, Castro Verde (Herdade do Reguengo). Ref. **55**-5-b, 1j, 21/04/2012, Castro Verde (Herdade da Barrigoa). Ref. **57**-5-e, 1j, 06/04/2012, Castro Verde (Monte da Azinheira). Ref. **57**-9-b, 1j, 06/04/2012, Castro Verde (Monte da Azinheira). Ref. **67**-4-a, 1j, 12/04/2012, Aljustrel (Monte da Chaiça). Ref. **67**-10-f, 2jj, 12/04/2012, Aljustrel (Monte da Chaiça). Ref. **78**-7-b, 1j, 09/04/2012, Castro Verde (Herdade de São Marcos). Ref. **79**-3-h, 1j, 07/04/2012, Castro Verde (Monte da Chada). Ref. **79**-7-g, 1j, 07/04/2012, Castro Verde (Monte da Chada). Ref. **81**-11-g, 1j,

20/04/2012, Castro Verde (Herdade de Reidias). Ref. **83**-2-g, 1j, 06/05/2012, Castro Verde (Herdade do Álamo). Ref. **83**-5-k, 1j, 06/05/2012, Castro Verde (Herdade do Álamo). Ref. **83**-6-d, 1j, 06/05/2012, Castro Verde (Herdade do Álamo). Ref. **83**-7-h, 1j, 06/05/2012, Castro Verde (Herdade do Álamo). Ref. **83**-9-a, 1j, 06/05/2012, Castro Verde (Herdade do Álamo). Ref. **86**-2-a, 1j, 10/05/2012, Castro Verde (Herdade das Mestras). Ref. **87**-8-c, 1j, 06/05/2012, Castro Verde (Herdade das Mestras). Ref. **87**-9-a, 1j, 06/05/2012, Castro Verde (Herdade das Mestras). Ref. **89**-4-h, 1j, 06/05/2012, Castro Verde (Herdade do Carapetal). Ref. **89**-6-g, 1j, 06/05/2012, Castro Verde (Herdade do Carapetal). Ref. **89**-11-h, 1j, 06/05/2012, Castro Verde (Herdade do Carapetal). Ref. **93**-5-e, 1♂ 1j, 17/04/2012, Aljustrel (Herdade da Sobreira). Ref. **93**-9-a, 2jj, 17/04/2012, Aljustrel (Herdade da Sobreira). Ref. **96**-2-e, 1j, 11/04/2012, Castro Verde (Herdade dos Bispos). Ref. **96**-6-b, 1j, 11/04/2012, Castro Verde (Herdade dos Bispos). Ref. **96**-9-i, 1j, 11/04/2012, Castro Verde (Herdade dos Bispos). Ref. **96**-10-i, 1j, 11/04/2012, Castro Verde (Herdade dos Bispos). Ref. **97**-7-c, 1j, 13/04/2012, Castro Verde (Cumeada Nova). Ref. **104**-4-c, 3jj, 04/04/2012, Castro Verde (Herdade dos Touris e Rolão). Ref. **108**-4-a, 1j, 10/04/2012, Castro Verde (Herdade dos Merendeiros). Ref. **108**-6-e, 2jj, 10/04/2012, Castro Verde (Herdade dos Merendeiros). Ref. **108**-10-d, 1j, 10/04/2012, Castro Verde (Herdade dos Merendeiros). Ref. **109**-6-f, 2jj, 24/04/2012, Castro Verde (Monte da Achada). Ref. **109**-11-h, 1j, 24/04/2012, Castro Verde (Monte da Achada). Ref. **110**-5-c, 1j, 04/05/2012, Castro Verde (Monte da Perdigoa). Ref. **110**-8-e, 2jj, 04/05/2012, Castro Verde (Monte da Perdigoa). Ref. **110**-10-j, 2jj, 04/05/2012, Castro Verde (Monte da Perdigoa). Ref. **110**-11-b, 1♀, 04/05/2012, Castro Verde (Monte da Perdigoa). Ref. **114**-3-h, 3jj, 08/04/2012, Castro Verde (Herdade das

Cuchilhas). Ref. **114**-7-d, 1j, 08/04/2012, Castro Verde (Herdade das Cuchilhas). Ref. **114**-11-d, 1j, 08/04/2012, Castro Verde (Herdade das Cuchilhas). Ref. **114**-12-d, 2jj, 08/04/2012, Castro Verde (Herdade das Cuchilhas). Ref. **116**-5-d, 2jj, 10/04/2012, Castro Verde (Herdade dos Merendeiros). Ref. **116**-6-b, 1j, 10/04/2012, Castro Verde (Herdade dos Merendeiros). Ref. **116**-7-d, 1j, 10/04/2012, Castro Verde (Herdade dos Merendeiros). Ref. **121**-6-a, 1j, 16/04/2012, Castro Verde (Monte do Tacanho). Ref. **121**-9-b, 1j, 16/04/2012, Castro Verde (Monte do Tacanho). Ref. **122**-2-d, 1j, 12/04/2012, Castro Verde (Herdade da Chaiça Velha). Ref. **123**-2-c, 1j, 20/04/2012, Castro Verde (Herdade das bicadas). Ref. **123**-6-c, 1♂, 20/04/2012, Castro Verde (Herdade das bicadas). Ref. **124**-7-i, 1j, 18/04/2012, Castro Verde (Herdade das Mestras). Ref. **124**-10-b, 1j, 18/04/2012, Castro Verde (Herdade das Mestras). Ref. **125**-3-k, 1j, 17/04/2012, Aljustrel (Herdade da Sobreira). Ref. **125**-6-b, 1j, 17/04/2012, Aljustrel (Herdade da Sobreira). Ref. **125**-8-d, 1j, 17/04/2012, Aljustrel (Herdade da Sobreira). Ref. **125**-10-k, 2jj, 17/04/2012, Aljustrel (Herdade da Sobreira). Ref. **126**-1-f, 2jj, 05/04/2012, Castro Verde (Monte do Seixo). Ref. **126**-2-b, 1j, 05/04/2012, Castro Verde (Monte do Seixo). Ref. **126**-8-f, 3jj, 05/04/2012, Castro Verde (Monte do Seixo). Ref. **126**-11-b, 1♂ 1j, 05/04/2012, Castro Verde (Monte do Seixo). Ref. **126**-12-a, 3jj, 05/04/2012, Castro Verde (Monte do Seixo). Ref. **127**-2-b, 1j, 11/04/2012, Castro Verde (Herdade dos Montinhos). Ref. **127**-4-a, 3jj, 11/04/2012, Castro Verde (Herdade dos Montinhos). Ref. **127**-7-i, 1♂, 11/04/2012, Castro Verde (Herdade dos Montinhos). Ref. **128**-1-b, 3jj, 04/04/2012, Castro Verde (Herdade de Carriça-Viseus). Ref. **128**-2-a, 7jj, 04/04/2012, Castro Verde (Herdade de Carriça-Viseus). Ref. **128**-3-b, 3jj, 04/04/2012, Castro Verde (Herdade de Carriça-Viseus). Ref. **128**-4-b, 2jj,

04/04/2012, Castro Verde (Herdade de Carriça-Viseus). Ref. **128**-5-b, 1j, 04/04/2012, Castro Verde (Herdade de Carriça-Viseus). Ref. **128**-8-f, 2jj, 04/04/2012, Castro Verde (Herdade de Carriça-Viseus). Ref. **128**-11-d, 1j, 04/04/2012, Castro Verde (Herdade de Carriça-Viseus). Ref. **128**-12-a, 2jj, 04/04/2012, Castro Verde (Herdade de Carriça-Viseus). Ref. **132**-6-c, 1j, 06/05/2012, Castro Verde (Monte das Cabeceiras). Ref. **133**-4-b, 1j, 28/04/2012, Castro Verde (Monte da Albergaria). Ref. **133**-6-g, 1j, 28/04/2012, Castro Verde (Monte da Albergaria). Ref. **A46**-6-e, 2jj, 10/05/2012, Aljustrel (Monte da Chaiça). Ref. **A57**-7-f, 1j, 08/05/2012, Aljustrel (Herdade de Corta Rabos). Ref. **A57**-8-a, 1♂, 08/05/2012, Aljustrel (Herdade de Corta Rabos). Ref. **A57**-11-g, 1j, 08/05/2012, Aljustrel (Herdade de Corta Rabos). Ref. **A166**-3-g, 2jj, 07/05/2012, Castro Verde (Herdade dos Brunhachos). Ref. **A166**-4-a, 1j, 07/05/2012, Castro Verde (Herdade dos Brunhachos). Ref. **A166**-5-b, 1♂ 4jj, 07/05/2012, Castro Verde (Herdade dos Brunhachos). Ref. **A166**-7-a, 3jj, 07/05/2012, Castro Verde (Herdade dos Brunhachos). Ref. **A166**-8-a, 5jj, 07/05/2012, Castro Verde (Herdade dos Brunhachos). Ref. **A166**-9-c, 2jj, 07/05/2012, Castro Verde (Herdade dos Brunhachos). Ref. **A166**-10-b, 2jj, 07/05/2012, Castro Verde (Herdade dos Brunhachos). Ref. **A166**-11-f, 2jj, 07/05/2012, Castro Verde (Herdade dos Brunhachos). Ref. **A260**-10-c, 1j, 07/05/2012, Castro Verde (Herdade de Reidias). Ref. **A297**-5-a, 4jj, 09/05/2012, Castro Verde (Lagoa da Mó). Ref. **A297**-7-a, 1j, 09/05/2012, Castro Verde (Lagoa da Mó). Ref. **A297**-8-j, 1j, 09/05/2012, Castro Verde (Lagoa da Mó). Ref. **A297**-9-c, 1j, 09/05/2012, Castro Verde (Lagoa da Mó). Ref. **A297**-11-f, 2jj, 09/05/2012, Castro Verde (Lagoa da Mó). Ref. **A299**-4-d, 2jj, 08/05/2012, Castro Verde (Lagoa da Mó). Ref. **A299**-5-f, 1j, 08/05/2012, Castro Verde (Lagoa da Mó). Ref. **A299**-8-j, 3jj,

08/05/2012, Castro Verde (Lagoa da Mó). Ref. **A299**-10-f, 1j, 08/05/2012, Castro Verde (Lagoa da Mó). Ref. **A299**-11-d, 1j, 08/05/2012, Castro Verde (Lagoa da Mó). Ref. **A299**-11R-b, 1j, 08/05/2012, Castro Verde (Lagoa da Mó). Ref. **A423**-2-j, 1j, 08/05/2012, Castro Verde (Herdade da Zibreira). Ref. **A423**-3-i, 1j, 08/05/2012, Castro Verde (Herdade da Zibreira). Ref. **A423**-4-i, 1j, 08/05/2012, Castro Verde (Herdade da Zibreira). Ref. **A423**-5-f, 1j, 08/05/2012, Castro Verde (Herdade da Zibreira). Ref. **A423**-6-d, 1j, 08/05/2012, Castro Verde (Herdade da Zibreira). Ref. **A423**-8-b, 3jj, 08/05/2012, Castro Verde (Herdade da Zibreira). Ref. **A423**-9-a, 2jj, 08/05/2012, Castro Verde (Herdade da Zibreira). Ref. **A482**-9-e, 1j, 09/05/2012, Castro Verde (Amendoeira Nova). Ref. **A505**-6-b, 1j, 08/05/2012, Castro Verde (Herdade do Reguengo). Ref. **A505**-11-c, 2jj, 08/05/2012, Castro Verde (Herdade do Reguengo). Ref. **A999**-3-b, 1j, 09/05/2012, Castro Verde (Herdade dos Pereiros). Ref. **A999**-6-e, 1j, 09/05/2012, Castro Verde (Herdade dos Pereiros). Ref. **A999**-10-b, 1j, 09/05/2012, Castro Verde (Herdade dos Pereiros).

***Oxyopes* sp.**

Material examined: Ref. **36**-3-a, 1j, 11/04/2012, Castro Verde (Herdade dos Bispos). Ref. **45**-4-f, 1j, 13/04/2012, Castro Verde (Monte das Fontes Barbas Velho). Ref. **47**-4-g, 1j, 09/04/2012, Castro Verde (Herdade do Almarginho). Ref. **47**-5-g, 1j, 09/04/2012, Castro Verde (Herdade do Almarginho). Ref. **53**-2-d, 3jj, 20/04/2012, Castro Verde (Herdade do Reguengo). Ref. **61**-5-h, 1j, 05/05/2012, Aljustrel (Monte da Torre). Ref. **67**-3-c, 1j, 12/04/2012, Aljustrel (Monte da Chaiça). Ref. **67**-7-f, 1j, 12/04/2012, Aljustrel (Monte da Chaiça). Ref. **67**-11-f, 1j, 12/04/2012, Aljustrel (Monte da Chaiça). Ref. **73**-4-e, 1j, 19/04/2012, Castro Verde (Herdade

dos Longos). Ref. **83**-5-k, 3jj, 06/05/2012, Castro Verde (Herdade do Álamo). Ref. **83**-8-f, 1j, 06/05/2012, Castro Verde (Herdade do Álamo). Ref. **86**-3-d, 1j, 10/05/2012, Castro Verde (Herdade das Mestras). Ref. **122**-7-c, 1j, 12/04/2012, Castro Verde (Herdade da Chaiça Velha). Ref. **124**-4-i, 1j, 18/04/2012, Castro Verde (Herdade das Mestras). Ref. **125**-4-k, 1j, 17/04/2012, Aljustrel (Herdade da Sobreira). Ref. **129**-11-g, 1j, 08/05/2012, Castro Verde (Herdade da Zibreira).

Family PHILODROMIDAE Thorell, 1870

Pulchellodromus pulchellus **(Lucas, 1846)**

Material examined: Ref. **15**-3-b, 1j, 12/04/2012, Castro Verde (Lagoa da Mó). Ref. **23**-10-k, 1j, 03/04/2012, Castro Verde (Monte do Vale das Gretas). Ref. **23**-11-i, 2jj, 03/04/2012, Castro Verde (Monte do Vale das Gretas). Ref. **25**-11-a, 1♂, 05/04/2012, Castro Verde (Herdade dos Touris e Rolão). Ref. **37**-5-b, 1j, 09/04/2012, Castro Verde (Herdade de São Marcos). Ref. **43**-4-d, 1j, 21/04/2012, Castro Verde (Monte da Comenda). Ref. **43**-11-h, 1j, 21/04/2012, Castro Verde (Monte da Comenda). Ref. **47**-2-j, 1j, 09/04/2012, Castro Verde (Herdade do Almarginho). Ref. **53**-2-d, 1♂, 20/04/2012, Castro Verde (Herdade do Reguengo). Ref. **53**-5-e, 1j, 20/04/2012, Castro Verde (Herdade do Reguengo). Ref. **55**-4-d, 1j, 21/04/2012, Castro Verde (Herdade da Barrigoa). Ref. **92**-5-e, 1j, 17/04/2012, Aljustrel (Monte do Carregueiro). Ref. **113**-3-c, 1j, 10/04/2012, Castro Verde (Courela do Monte Novo). Ref. **114**-7-d, 1♂, 08/04/2012, Castro Verde (Herdade das Cuchilhas). Ref. **114**-10-a, 1♂, 08/04/2012, Castro Verde (Herdade das Cuchilhas). Ref. **124**-2-b, 1♂, 18/04/2012, Castro Verde (Herdade das Mestras). Ref. **131**-8-a, 1j, 04/05/2012, Castro Verde (Herdade do Torrejão). Ref. **A297**-11-

f, 1♀, 09/05/2012, Castro Verde (Lagoa da Mó). Ref. **A299**-6-d, 1♂, 08/05/2012, Castro Verde (Lagoa da Mó). Ref. **A482**-11-d, 1j, 09/05/2012, Castro Verde (Amendoeira Nova).

Thanatus vulgaris **Simon, 1870**

Material examined: Ref. **4**-2-c, 1j, 15/04/2012, Castro Verde (Heredade das Mouras). Ref. **4**-9-a, 1j, 15/04/2012, Castro Verde (Heredade das Mouras). Ref. **6**-10-e, 2jj, 24/04/2012, Castro Verde (Herdade de A de Neves da Marinha). Ref. **14**-7-e, 1j, 18/04/2012, Castro Verde (Lagoa da Mó). Ref. **14**-9-h, 1j, 18/04/2012, Castro Verde (Lagoa da Mó). Ref. **15**-4-e, 1j, 12/04/2012, Castro Verde (Lagoa da Mó). Ref. **15**-7-e, 1j, 12/04/2012, Castro Verde (Lagoa da Mó). Ref. **15**-11-c, 2jj, 12/04/2012, Castro Verde (Lagoa da Mó). Ref. **19**-5-c, 1j, 26/04/2012, Castro Verde (Herdade do Torrejão). Ref. **21**-2-a, 1j, 21/04/2012, Castro Verde (Herdade da Barrigoa). Ref. **21**-7-a, 1j, 21/04/2012, Castro Verde (Herdade da Barrigoa). Ref. **21**-9-a, 1j, 21/04/2012, Castro Verde (Herdade da Barrigoa). Ref. **23**-1-a, 1j, 03/04/2012, Castro Verde (Monte do Vale das Gretas). Ref. **23**-3-c, 1j, 03/04/2012, Castro Verde (Monte do Vale das Gretas). Ref. **23**-4-a, 1j, 03/04/2012, Castro Verde (Monte do Vale das Gretas). Ref. **23**-9-a, 2jj, 03/04/2012, Castro Verde (Monte do Vale das Gretas). Ref. **23**-10-k, 2jj, 03/04/2012, Castro Verde (Monte do Vale das Gretas). Ref. **23**-11-i, 1j, 03/04/2012, Castro Verde (Monte do Vale das Gretas). Ref. **25**-2-d, 1j, 05/04/2012, Castro Verde (Herdade dos Touris e Rolão). Ref. **27**-3-d, 1j, 24/04/2012, Castro Verde (Herdade das Sesmarias). Ref. **27**-7-d, 1j, 24/04/2012, Castro Verde (Herdade das Sesmarias). Ref. **27**-9-e, 1j, 24/04/2012, Castro Verde (Herdade das Sesmarias). Ref. **27**-10-d, 1j, 24/04/2012, Castro Verde

(Herdade das Sesmarias). Ref. **27**-11-c, 1j, 24/04/2012, Castro Verde (Herdade das Sesmarias). Ref. **34**-3-c, 1j, 16/04/2012, Castro Verde (Herdade do Tacanho e Merendeiros). Ref. **36**-2-a, 1j, 11/04/2012, Castro Verde (Herdade dos Bispos). Ref. **36**-3-e, 1j, 11/04/2012, Castro Verde (Herdade dos Bispos). Ref. **36**-4-e, 1j, 11/04/2012, Castro Verde (Herdade dos Bispos). Ref. **36**-5-a, 1j, 11/04/2012, Castro Verde (Herdade dos Bispos). Ref. **36**-10-g, 1j, 11/04/2012, Castro Verde (Herdade dos Bispos). Ref. **45**-9-a, 1j, 13/04/2012, Castro Verde (Monte das Fontes Barbas Velho). Ref. **47**-1-a, 1j, 09/04/2012, Castro Verde (Herdade do Almarginho). Ref. **47**-2-j, 1j, 09/04/2012, Castro Verde (Herdade do Almarginho). Ref. **47**-6-c, 2jj, 09/04/2012, Castro Verde (Herdade do Almarginho). Ref. **49**-2-d, 2jj, 25/04/2012, Castro Verde (Herdade da Navarra). Ref. **49**-4-b, 2jj, 25/04/2012, Castro Verde (Herdade da Navarra). Ref. **49**-5-a, 1j, 25/04/2012, Castro Verde (Herdade da Navarra). Ref. **49**-8-d, 1j, 25/04/2012, Castro Verde (Herdade da Navarra). Ref. **49**-9-a, 1♂ 1j, 25/04/2012, Castro Verde (Herdade da Navarra). Ref. **49**-10-b, 3jj, 25/04/2012, Castro Verde (Herdade da Navarra). Ref. **50**-3-f, 1j, 16/04/2012, Castro Verde (Herdade do Torrejão). Ref. **50**-4-c, 1j, 16/04/2012, Castro Verde (Herdade do Torrejão). Ref. **51**-2-a, 1j, 13/04/2012, Castro Verde (Herdade do Torrejão). Ref. **53**-2-d, 1j, 20/04/2012, Castro Verde (Herdade do Reguengo). Ref. **53**-5-e, 1j, 20/04/2012, Castro Verde (Herdade do Reguengo). Ref. **55**-4-d, 1j, 21/04/2012, Castro Verde (Herdade da Barrigoa). Ref. **55**-11-a, 1j, 21/04/2012, Castro Verde (Herdade da Barrigoa). Ref. **56**-9-f, 1j, 18/04/2012, Castro Verde (Herdade da Barrigoa). Ref. **56**-11-b, 1j, 18/04/2012, Castro Verde (Herdade da Barrigoa). Ref. **57**-8-b, 1j, 06/04/2012, Castro Verde (Monte da Azinheira). Ref. **61**-3-c, 1j, 05/05/2012, Aljustrel (Monte da Torre). Ref. **61**-6-j, 1j, 05/05/2012, Aljustrel (Monte da Torre). Ref. **61**-7-ej, 1j, 05/05/2012,

Aljustrel (Monte da Torre). Ref. **67**-3-c, 1j, 12/04/2012, Aljustrel (Monte da Chaiça). Ref. **67**-5-j, 1j, 12/04/2012, Aljustrel (Monte da Chaiça). Ref. **67**-7-f, 2jj, 12/04/2012, Aljustrel (Monte da Chaiça). Ref. **72**-6-a, 1j, 15/04/2012, Castro Verde (Cumeada Nova). Ref. **72**-7-f, 1j, 15/04/2012, Castro Verde (Cumeada Nova). Ref. **73**-9-j, 1j, 19/04/2012, Castro Verde (Herdade dos Longos). Ref. **73**-10-h, 1j, 19/04/2012, Castro Verde (Herdade dos Longos). Ref. **78**-10-c, 1j, 09/04/2012, Castro Verde (Herdade de São Marcos). Ref. **79**-12-e, 1j, 07/04/2012, Castro Verde (Monte da Chada). Ref. **81**-11-g, 1j, 20/04/2012, Castro Verde (Herdade de Reidias). Ref. **83**-2-g, 1j, 06/05/2012, Castro Verde (Herdade do Álamo). Ref. **83**-3-b, 2jj, 06/05/2012, Castro Verde (Herdade do Álamo). Ref. **83**-11-a, 1j, 06/05/2012, Castro Verde (Herdade do Álamo). Ref. **86**-4-c, 1j, 10/05/2012, Castro Verde (Herdade das Mestras). Ref. **86**-6-g, 1j, 10/05/2012, Castro Verde (Herdade das Mestras). Ref. **86**-7-b, 2jj, 10/05/2012, Castro Verde (Herdade das Mestras). Ref. **86**-10-a, 1j, 10/05/2012, Castro Verde (Herdade das Mestras). Ref. **87**-7-g, 1j, 06/05/2012, Castro Verde (Herdade das Mestras). Ref. **93**-4-i, 1j, 17/04/2012, Aljustrel (Herdade da Sobreira). Ref. **96**-9-i, 1j, 11/04/2012, Castro Verde (Herdade dos Bispos). Ref. **96**-10-i, 1j, 11/04/2012, Castro Verde (Herdade dos Bispos). Ref. **97**-10-i, 1j, 13/04/2012, Castro Verde (Cumeada Nova). Ref. **104**-6-c, 1j, 04/04/2012, Castro Verde (Herdade dos Touris e Rolão). Ref. **104**-8-g, 1j, 04/04/2012, Castro Verde (Herdade dos Touris e Rolão). Ref. **104**-9-d, 1j, 04/04/2012, Castro Verde (Herdade dos Touris e Rolão). Ref. **104**-12-a, 2jj, 04/04/2012, Castro Verde (Herdade dos Touris e Rolão). Ref. **107**-3-d, 1j, 18/04/2012, Castro Verde (Herdade das Mestras). Ref. **107**-6-d, 1j, 18/04/2012, Castro Verde (Herdade das Mestras). Ref. **107**-9-a, 1j, 18/04/2012, Castro Verde (Herdade das Mestras). Ref. **108**-10-d, 1j, 10/04/2012,

Castro Verde (Herdade dos Merendeiros). Ref. **108**-11-a, 1j, 10/04/2012, Castro Verde (Herdade dos Merendeiros). Ref. **109**-3-i, 1j, 24/04/2012, Castro Verde (Monte da Achada). Ref. **109**-4-b, 1j, 24/04/2012, Castro Verde (Monte da Achada). Ref. **109**-5-c, 2jj, 24/04/2012, Castro Verde (Monte da Achada). Ref. **109**-8-c, 1j, 24/04/2012, Castro Verde (Monte da Achada). Ref. **109**-11-h, 1j, 24/04/2012, Castro Verde (Monte da Achada). Ref. **110**-2-j, 1j, 04/05/2012, Castro Verde (Monte da Perdigoa). Ref. **110**-4-d, 1j, 04/05/2012, Castro Verde (Monte da Perdigoa). Ref. **112**-8-i, 1j, 20/04/2012, Castro Verde (Monte do Freire). Ref. **112**-11-d, 1j, 20/04/2012, Castro Verde (Monte do Freire). Ref. **114**-1-c, 1j, 08/04/2012, Castro Verde (Herdade das Cuchilhas). Ref. **114**-5-f, 1j, 08/04/2012, Castro Verde (Herdade das Cuchilhas). Ref. **121**-9-b, 1j, 16/04/2012, Castro Verde (Monte do Tacanho). Ref. **122**-5-f, 1j, 12/04/2012, Castro Verde (Herdade da Chaiça Velha). Ref. **122**-7-c, 1j, 12/04/2012, Castro Verde (Herdade da Chaiça Velha). Ref. **122**-9-b, 1j, 12/04/2012, Castro Verde (Herdade da Chaiça Velha). Ref. **122**-11-a, 1j, 12/04/2012, Castro Verde (Herdade da Chaiça Velha). Ref. **123**-2-c, 2jj, 20/04/2012, Castro Verde (Herdade das bicadas). Ref. **123**-10-a, 1j, 20/04/2012, Castro Verde (Herdade das bicadas). Ref. **125**-6-b, 1j, 17/04/2012, Aljustrel (Herdade da Sobreira). Ref. **125**-7-f, 1j, 17/04/2012, Aljustrel (Herdade da Sobreira). Ref. **125**-10-k, 1j, 17/04/2012, Aljustrel (Herdade da Sobreira). Ref. **126**-6-a, 1j, 05/04/2012, Castro Verde (Monte do Seixo). Ref. **126**-8-f, 1j, 05/04/2012, Castro Verde (Monte do Seixo). Ref. **126**-9-a, 1j, 05/04/2012, Castro Verde (Monte do Seixo). Ref. **126**-11-b, 1j, 05/04/2012, Castro Verde (Monte do Seixo). Ref. **127**-5-b, 2jj, 11/04/2012, Castro Verde (Herdade dos Montinhos). Ref. **128**-4-b, 1j, 04/04/2012, Castro Verde (Herdade de Carriça-Viseus). Ref. **128**-9-a, 1j, 04/04/2012, Castro Verde (Herdade de

Carriça-Viseus). Ref. **129**-2-k, 1j, 08/05/2012, Castro Verde (Herdade da Zibreira). Ref. **129**-11-g, 1j, 08/05/2012, Castro Verde (Herdade da Zibreira). Ref. **131**-4-c, 1j, 04/05/2012, Castro Verde (Herdade do Torrejão). Ref. **131**-10-g, 1j, 04/05/2012, Castro Verde (Herdade do Torrejão). Ref. **132**-6-c, 1j, 06/05/2012, Castro Verde (Monte das Cabeceiras). Ref. **133**-9-j, 1j, 28/04/2012, Castro Verde (Monte da Albergaria). Ref. **A46**-3-h, 1j, 10/05/2012, Aljustrel (Monte da Chaiça). Ref. **A46**-7-c3, 1j, 10/05/2012, Aljustrel (Monte da Chaiça). Ref. **A166**-4-a, 1j, 07/05/2012, Castro Verde (Herdade dos Brunhachos). Ref. **A166**-5-b, 1j, 07/05/2012, Castro Verde (Herdade dos Brunhachos). Ref. **A166**-6-b, 1j, 07/05/2012, Castro Verde (Herdade dos Brunhachos). Ref. **A166**-9-c, 1j, 07/05/2012, Castro Verde (Herdade dos Brunhachos). Ref. **A166**-11-f, 1j, 07/05/2012, Castro Verde (Herdade dos Brunhachos). Ref. **A260**-5-g, 2jj, 07/05/2012, Castro Verde (Herdade de Reidias). Ref. **A260**-10-c, 1♀, 07/05/2012, Castro Verde (Herdade de Reidias). Ref. **A297**-10-f, 1j, 09/05/2012, Castro Verde (Lagoa da Mó). Ref. **A299**-4-d, 1j, 08/05/2012, Castro Verde (Lagoa da Mó). Ref. **A299**-8-j, 1j, 08/05/2012, Castro Verde (Lagoa da Mó). Ref. **A299**-9-b, 1j, 08/05/2012, Castro Verde (Lagoa da Mó). Ref. **A299**-11-d, 2jj, 08/05/2012, Castro Verde (Lagoa da Mó). Ref. **A349**-5-a, 1j, 10/05/2012, Castro Verde (Herdade das Mestras). Ref. **A349**-7-a, 1j, 10/05/2012, Castro Verde (Herdade das Mestras). Ref. **A505**-8-c, 1j, 08/05/2012, Castro Verde (Herdade do Reguengo). Ref. **A527**-2-e, 1j, 07/05/2012, Castro Verde (Herdade dos Bispos). Ref. **A527**-4-k, 1j, 07/05/2012, Castro Verde (Herdade dos Bispos). Ref. **A527**-8-g, 1j, 07/05/2012, Castro Verde (Herdade dos Bispos).

***Tibellus macellus* Simon, 1875**

Material examined: Ref. **61**-4-a, 1♀, 05/05/2012, Aljustrel (Monte da Torre).

Family PISAURIDAE Simon, 1890

Pisaura mirabilis **(Clerck, 1757)**

Material examined: Ref. **57**-6-a, 1♀, 06/04/2012, Castro Verde (Monte da Azinheira). Ref. **112**-5-b, 1j, 20/04/2012, Castro Verde (Monte do Freire). Ref. **125**-2-b, 1j, 17/04/2012, Aljustrel (Herdade da Sobreira).

Family SALTICIDAE Blackwall, 1841

Chalcoscirtus infimus **(Simon, 1868)**

Material examined: Ref. **4**-4-g, 1j, 15/04/2012, Castro Verde (Heredade das Mouras). Ref. **6**-7-f, 1j, 24/04/2012, Castro Verde (Herdade de A de Neves da Marinha). Ref. **9**-2-g, 1j, 25/04/2012, Castro Verde (COSA - Herdade de São Marcos). Ref. **9**-8-b, 1♂ 1j, 25/04/2012, Castro Verde (COSA - Herdade de São Marcos). Ref. **11**-2-b, 1j, 15/04/2012, Castro Verde (Monte do Broco e Capitão). Ref. **11**-3-d, 1j, 15/04/2012, Castro Verde (Monte do Broco e Capitão). Ref. **11**-7-c, 1j, 15/04/2012, Castro Verde (Monte do Broco e Capitão). Ref. **14**-2-c, 1j, 18/04/2012, Castro Verde (Lagoa da Mó). Ref. **14**-5-b, 1j, 18/04/2012, Castro Verde (Lagoa da Mó). Ref. **15**-11-c, 1j, 12/04/2012, Castro Verde (Lagoa da Mó). Ref. **19**-10-e, 1♂, 26/04/2012, Castro Verde (Herdade do Torrejão). Ref. **23**-2-a, 1♂, 03/04/2012, Castro Verde (Monte do Vale das Gretas). Ref. **27**-2-c, 2jj, 24/04/2012, Castro Verde (Herdade das Sesmarias). Ref. **27**-3-d, 1j, 24/04/2012, Castro Verde (Herdade das Sesmarias). Ref. **27**-4-e, 1j, 24/04/2012, Castro

Verde (Herdade das Sesmarias). Ref. **27**-11-c, 1j, 24/04/2012, Castro Verde (Herdade das Sesmarias). Ref. **34**-4-a, 1j, 16/04/2012, Castro Verde (Herdade do Tacanho e Merendeiros). Ref. **35**-5-f, 1j, 05/04/2012, Castro Verde (Monte do Seixo). Ref. **35**-9-g, 1j, 05/04/2012, Castro Verde (Monte do Seixo). Ref. **36**-3-e, 1j, 11/04/2012, Castro Verde (Herdade dos Bispos). Ref. **36**-6-h, 2jj, 11/04/2012, Castro Verde (Herdade dos Bispos). Ref. **36**-10-g, 1j, 11/04/2012, Castro Verde (Herdade dos Bispos). Ref. **36**-11-d, 2jj, 11/04/2012, Castro Verde (Herdade dos Bispos). Ref. **37**-4-d, 1j, 09/04/2012, Castro Verde (Herdade de São Marcos). Ref. **37**-6-a, 1j, 09/04/2012, Castro Verde (Herdade de São Marcos). Ref. **37**-10-e, 1j, 09/04/2012, Castro Verde (Herdade de São Marcos). Ref. **37**-11-c, 2jj, 09/04/2012, Castro Verde (Herdade de São Marcos). Ref. **43**-5-e, 1j, 21/04/2012, Castro Verde (Monte da Comenda). Ref. **47**-1-a, 3jj, 09/04/2012, Castro Verde (Herdade do Almarginho). Ref. **47**-2-j, 1j, 09/04/2012, Castro Verde (Herdade do Almarginho). Ref. **47**-7-e, 2jj, 09/04/2012, Castro Verde (Herdade do Almarginho). Ref. **47**-9-i, 1j, 09/04/2012, Castro Verde (Herdade do Almarginho). Ref. **47**-11-h, 1j, 09/04/2012, Castro Verde (Herdade do Almarginho). Ref. **49**-10-b, 1j, 25/04/2012, Castro Verde (Herdade da Navarra). Ref. **49**-11-h, 1j, 25/04/2012, Castro Verde (Herdade da Navarra). Ref. **51**-2-a, 1j, 13/04/2012, Castro Verde (Herdade do Torrejão). Ref. **54**-4-a, 1j, 25/04/2012, Castro Verde (Herdade do Roncanho). Ref. **54**-6-f, 1♂, 25/04/2012, Castro Verde (Herdade do Roncanho). Ref. **54**-8a-a, 1j, 25/04/2012, Castro Verde (Herdade do Roncanho). Ref. **54**-8b-d, 1♂, 25/04/2012, Castro Verde (Herdade do Roncanho). Ref. **55**-4-d, 2jj, 21/04/2012, Castro Verde (Herdade da Barrigoa). Ref. **55**-7-b, 1j, 21/04/2012, Castro Verde (Herdade da Barrigoa). Ref. **55**-10-b, 1j, 21/04/2012, Castro Verde (Herdade da Barrigoa). Ref. **56**-9-f, 1j, 18/04/2012, Castro Verde (Herdade da

Barrigoa). Ref. **56**-10-b, 1j, 18/04/2012, Castro Verde (Herdade da Barrigoa). Ref. **57**-1-a, 1j, 06/04/2012, Castro Verde (Monte da Azinheira). Ref. **57**-7-e, 3jj, 06/04/2012, Castro Verde (Monte da Azinheira). Ref. **57**-8-b, 1j, 06/04/2012, Castro Verde (Monte da Azinheira). Ref. **57**-9-b, 2jj, 06/04/2012, Castro Verde (Monte da Azinheira). Ref. **58**-4R-f, 1j, 07/04/2012, Mértola (Herdade da Benviúda). Ref. **58**-5-c, 1j, 07/04/2012, Mértola (Herdade da Benviúda). Ref. **58**-9-d, 1j, 07/04/2012, Mértola (Herdade da Benviúda). Ref. **58**-12-d, 2jj, 07/04/2012, Mértola (Herdade da Benviúda). Ref. **61**-10-h, 1j, 05/05/2012, Aljustrel (Monte da Torre). Ref. **67**-6-i, 1j, 12/04/2012, Aljustrel (Monte da Chaiça). Ref. **67**-8-d, 1j, 12/04/2012, Aljustrel (Monte da Chaiça). Ref. **67**-9-c, 1j, 12/04/2012, Aljustrel (Monte da Chaiça). Ref. **70**-7-a, 1j, 21/04/2012, Castro Verde (Monte dos Janeiros). **72**-10-d, 1j, 15/04/2012, Castro Verde (Cumeada Nova). Ref. **73**-5-h, 1j, 19/04/2012, Castro Verde (Herdade dos Longos). Ref. **73**-6-c, 1j, 19/04/2012, Castro Verde (Herdade dos Longos). Ref. Ref. **78**-7-b, 1j, 09/04/2012, Castro Verde (Herdade de São Marcos). Ref. **78**-10-c, 1j, 09/04/2012, Castro Verde (Herdade de São Marcos). Ref. **79**-1-c, 1j, 07/04/2012, Castro Verde (Monte da Chada). Ref. **79**-2-e, 2jj, 07/04/2012, Castro Verde (Monte da Chada). Ref. **79**-3-h, 1j, 07/04/2012, Castro Verde (Monte da Chada). Ref. **79**-5-d, 1j, 07/04/2012, Castro Verde (Monte da Chada). Ref. **79**-6-e, 1j, 07/04/2012, Castro Verde (Monte da Chada). Ref. **81**-4-h, 2jj, 20/04/2012, Castro Verde (Herdade de Reidias). Ref. **81**-8-h, 2♂♂, 20/04/2012, Castro Verde (Herdade de Reidias). Ref. **83**-6-d, 1♂, 06/05/2012, Castro Verde (Herdade do Álamo). Ref. **83**-8-f, 1♂, 06/05/2012, Castro Verde (Herdade do Álamo). Ref. **83**-9-a, 1♂, 06/05/2012, Castro Verde (Herdade do Álamo). Ref. **86**-7-b, 2jj, 10/05/2012, Castro Verde (Herdade das Mestras). Ref. **87**-2-c, 1j, 06/05/2012, Castro Verde (Herdade das

Mestras). Ref. **87**-7-g, 1♂, 06/05/2012, Castro Verde (Herdade das Mestras). Ref. **87**-9-a, 1j, 06/05/2012, Castro Verde (Herdade das Mestras). Ref. **87**-11-a, 1♂, 06/05/2012, Castro Verde (Herdade das Mestras). Ref. **88**-7-e, 1j, 25/04/2012, Castro Verde (Herdade da Portela). Ref. **88**-11-j, 1♂, 25/04/2012, Castro Verde (Herdade da Portela). Ref. **89**-9-h, 1j, 06/05/2012, Castro Verde (Herdade do Carapetal). Ref. **89**-10-c, 1♂, 06/05/2012, Castro Verde (Herdade do Carapetal). Ref. **92**-3-g, 1♂, 17/04/2012, Aljustrel (Monte do Carregueiro). Ref. **92**-7-e, 1j, 17/04/2012, Aljustrel (Monte do Carregueiro). Ref. **93**-4-i, 1♂ 1j, 17/04/2012, Aljustrel (Herdade da Sobreira). Ref. **93**-6-h, 1j, 17/04/2012, Aljustrel (Herdade da Sobreira). Ref. **93**-11-c, 1j, 17/04/2012, Aljustrel (Herdade da Sobreira). Ref. **96**-6-b, 1j, 11/04/2012, Castro Verde (Herdade dos Bispos). Ref. **96**-10-i, 1j, 11/04/2012, Castro Verde (Herdade dos Bispos). Ref. **97**-5-c, 1j, 13/04/2012, Castro Verde (Cumeada Nova). Ref. **97**-7-c, 1j, 13/04/2012, Castro Verde (Cumeada Nova). Ref. **102**-2-c, 1♂, 15/04/2012, Castro Verde (Monte da Fonte). Ref. **102**-7-f, 1♂, 15/04/2012, Castro Verde (Monte da Fonte). Ref. **104**-1-b, 1j, 04/04/2012, Castro Verde (Herdade dos Touris e Rolão). Ref. **104**-2-d, 1j, 04/04/2012, Castro Verde (Herdade dos Touris e Rolão). Ref. **104**-9-d, 1j, 04/04/2012, Castro Verde (Herdade dos Touris e Rolão). Ref. **104**-12-a, 1j, 04/04/2012, Castro Verde (Herdade dos Touris e Rolão). Ref. **107**-6-d, 1j, 18/04/2012, Castro Verde (Herdade das Mestras). Ref. **107**-11-f, 1♂, 18/04/2012, Castro Verde (Herdade das Mestras). Ref. **108**-2-d, 2jj, 10/04/2012, Castro Verde (Herdade dos Merendeiros). Ref. **108**-4-a, 1j, 10/04/2012, Castro Verde (Herdade dos Merendeiros). Ref. **108**-5-a, 1j, 10/04/2012, Castro Verde (Herdade dos Merendeiros). Ref. **108**-9-d, 1j, 10/04/2012, Castro Verde (Herdade dos Merendeiros). Ref. **108**-11-a, 1j, 10/04/2012, Castro Verde

(Herdade dos Merendeiros). Ref. **109**-6-f, 1j, 24/04/2012, Castro Verde (Monte da Achada). Ref. **110**-10-j, 1j, 04/05/2012, Castro Verde (Monte da Perdigoa). Ref. **110**-11-b, 2♂♂, 04/05/2012, Castro Verde (Monte da Perdigoa). Ref. **112**-5-b, 1♂, 20/04/2012, Castro Verde (Monte do Freire). Ref. **112**-7-h, 1j, 20/04/2012, Castro Verde (Monte do Freire). Ref. **113**-2-b, 1♂, 10/04/2012, Castro Verde (Courela do Monte Novo). Ref. **113**-4-j, 1j, 10/04/2012, Castro Verde (Courela do Monte Novo). Ref. **113**-6-a, 1j, 10/04/2012, Castro Verde (Courela do Monte Novo). Ref. **113**-7-e, 1j, 10/04/2012, Castro Verde (Courela do Monte Novo). Ref. **114**-2-i, 1j, 08/04/2012, Castro Verde (Herdade das Cuchilhas). Ref. **114**-4-a, 2jj, 08/04/2012, Castro Verde (Herdade das Cuchilhas). Ref. **114**-5-f, 1j, 08/04/2012, Castro Verde (Herdade das Cuchilhas). Ref. **114**-8-c, 2jj, 08/04/2012, Castro Verde (Herdade das Cuchilhas). Ref. **114**-9-h, 1j, 08/04/2012, Castro Verde (Herdade das Cuchilhas). Ref. **114**-11-d, 1j, 08/04/2012, Castro Verde (Herdade das Cuchilhas). Ref. **114**-12-d, 2jj, 08/04/2012, Castro Verde (Herdade das Cuchilhas). Ref. **116**-3-c, 1j, 10/04/2012, Castro Verde (Herdade dos Merendeiros). Ref. **116**-6-b, 1j, 10/04/2012, Castro Verde (Herdade dos Merendeiros). Ref. **121**-9-b, 1j, 16/04/2012, Castro Verde (Monte do Tacanho). Ref. **122**-2-d, 1j, 12/04/2012, Castro Verde (Herdade da Chaiça Velha). Ref. **122**-11-a, 1j, 12/04/2012, Castro Verde (Herdade da Chaiça Velha). Ref. **123**-3-c, 2jj, 20/04/2012, Castro Verde (Herdade das bicadas). Ref. **123**-7-d, 1♂ 1j, 20/04/2012, Castro Verde (Herdade das bicadas). Ref. **124**-2-b, 1j, 18/04/2012, Castro Verde (Herdade das Mestras). Ref. **124**-11-f, 1j, 18/04/2012, Castro Verde (Herdade das Mestras). Ref. **125**-8-d, 1j, 17/04/2012, Aljustrel (Herdade da Sobreira). Ref. **125**-10-k, 1j, 17/04/2012, Aljustrel (Herdade da Sobreira). Ref. **126**-8-f, 1j, 05/04/2012, Castro Verde (Monte do Seixo). Ref. **126**-11-b, 1j,

05/04/2012, Castro Verde (Monte do Seixo). Ref. **127**-4-a, 1j, 11/04/2012, Castro Verde (Herdade dos Montinhos). Ref. **127**-3-d, 1j, 11/04/2012, Castro Verde (Herdade dos Montinhos). Ref. **128**-4-b, 2jj, 04/04/2012, Castro Verde (Herdade de Carriça-Viseus). Ref. **128**-8-f, 1j, 04/04/2012, Castro Verde (Herdade de Carriça-Viseus). Ref. **128**-12-a, 1j, 04/04/2012, Castro Verde (Herdade de Carriça-Viseus). Ref. **129**-8-b, 1j, 08/05/2012, Castro Verde (Herdade da Zibreira). Ref. **129**-9-b, 1j, 08/05/2012, Castro Verde (Herdade da Zibreira). Ref. **131**-2-f, 2jj, 04/05/2012, Castro Verde (Herdade do Torrejão). Ref. **131**-7-d, 1j, 04/05/2012, Castro Verde (Herdade do Torrejão). Ref. **131**-8-a, 1j, 04/05/2012, Castro Verde (Herdade do Torrejão). Ref. **131**-9-b, 1j, 04/05/2012, Castro Verde (Herdade do Torrejão). Ref. **132**-2-k, 1♂, 06/05/2012, Castro Verde (Monte das Cabeceiras). Ref. **132**-4-a, 1j, 06/05/2012, Castro Verde (Monte das Cabeceiras). Ref. **132**-7-b, 1j, 06/05/2012, Castro Verde (Monte das Cabeceiras). Ref. **132**-8-a, 1♂, 06/05/2012, Castro Verde (Monte das Cabeceiras). Ref. **132**-10-g, 1j, 06/05/2012, Castro Verde (Monte das Cabeceiras). Ref. **133**-10-e, 1j, 28/04/2012, Castro Verde (Monte da Albergaria). Ref. **A46**-2-g, 1j, 10/05/2012, Aljustrel (Monte da Chaiça). Ref. **A46**-6-e, 1j, 10/05/2012, Aljustrel (Monte da Chaiça). Ref. **A57**-3-h, 1♂, 08/05/2012, Aljustrel (Herdade de Corta Rabos). Ref. **A57**-4-h, 1j, 08/05/2012, Aljustrel (Herdade de Corta Rabos). Ref. **A57**-8-a, 1♂, 08/05/2012, Aljustrel (Herdade de Corta Rabos). Ref. **A57**-9-i, 1j, 08/05/2012, Aljustrel (Herdade de Corta Rabos). Ref. **A166**-3-g, 1♂ 1j, 07/05/2012, Castro Verde (Herdade dos Brunhachos). Ref. **A166**-11-f, 2jj, 07/05/2012, Castro Verde (Herdade dos Brunhachos). Ref. **A260**-6-g, 1j, 07/05/2012, Castro Verde (Herdade de Reidias). Ref. **A260**-10-c, 1♂ 1j, 07/05/2012, Castro Verde (Herdade de Reidias). Ref. **A297**-3-b, 1♂, 09/05/2012, Castro Verde (Lagoa da

Mó). Ref. **A297**-4-a, 2jj, 09/05/2012, Castro Verde (Lagoa da Mó). Ref. **A299**-2-c, 1j, 08/05/2012, Castro Verde (Lagoa da Mó). Ref. **A299**-4-d, 1♂ 1j, 08/05/2012, Castro Verde (Lagoa da Mó). Ref. **A299**-5-f, 1♂, 08/05/2012, Castro Verde (Lagoa da Mó). Ref. **A299**-6-d, 1j, 08/05/2012, Castro Verde (Lagoa da Mó). Ref. **A349**-6-k, 1♂ 1j, 10/05/2012, Castro Verde (Herdade das Mestras). Ref. **A349**-9-g, 1j, 10/05/2012, Castro Verde (Herdade das Mestras). Ref. **A388**-5-a, 1♂ 1j, 10/05/2012, Castro Verde (Monte da Achada). Ref. **A388**-11-a, 1j, 10/05/2012, Castro Verde (Monte da Achada). Ref. **A423**-4-i, 1j, 08/05/2012, Castro Verde (Herdade da Zibreira). Ref. **A423**-6-d, 1j, 08/05/2012, Castro Verde (Herdade da Zibreira). Ref. **A423**-9-a, 3♂♂, 08/05/2012, Castro Verde (Herdade da Zibreira). Ref. **A423**-10-d, 2jj, 08/05/2012, Castro Verde (Herdade da Zibreira). Ref. **A482**-11-d, 1j, 09/05/2012, Castro Verde (Amendoeira Nova). Ref. **A505**-9-k, 1♂, 08/05/2012, Castro Verde (Herdade do Reguengo). Ref. **A527**-8-g, 1♂, 07/05/2012, Castro Verde (Herdade dos Bispos). Ref. **A999**-3-b, 1j, 09/05/2012, Castro Verde (Herdade dos Pereiros). Ref. **A999**-4-f, 1♂, 09/05/2012, Castro Verde (Herdade dos Pereiros). Ref. **A999**-9-g, 2♂♂, 09/05/2012, Castro Verde (Herdade dos Pereiros).

Euophrys herbigarda **(Simon, 1871)**

Material examined: Ref. **6**-4-e, 1j, 24/04/2012, Castro Verde (Herdade de A de Neves da Marinha). Ref. **9**-5-a, 2♂♂ 1j, 25/04/2012, Castro Verde (COSA - Herdade de São Marcos). Ref. **9**-10-c, 1♂, 25/04/2012, Castro Verde (COSA - Herdade de São Marcos). Ref. **11**-11-e, 1j, 09/05/2012, Castro Verde (Monte do Broco e Capitão). Ref. **15**-6-a, 1j, 12/04/2012, Castro Verde (Lagoa da Mó). Ref. **15**-8-a, 1♂, 12/04/2012, Castro Verde (Lagoa da Mó). Ref. **21**-7-a, 1♂,

21/04/2012, Castro Verde (Herdade da Barrigoa). Ref. **36**-5-a, 1j, 11/04/2012, Castro Verde (Herdade dos Bispos). Ref. **36**-6-h, 1♂ 1j, 11/04/2012, Castro Verde (Herdade dos Bispos). Ref. **43**-5-e, 1j, 21/04/2012, Castro Verde (Monte da Comenda). Ref. **47**-5-g, 1♂, 09/04/2012, Castro Verde (Monte das Fontes Barbas Velho). Ref. **47**-12-c, 1j, 09/04/2012, Castro Verde (Herdade do Almarginho). Ref. **49**-4-b, 1♀, 25/04/2012, Castro Verde (Herdade da Navarra). Ref. **49-**7-c, 2♀♀ 1j, 25/04/2012, Castro Verde (Herdade da Navarra). Ref. **55**-5-b, 1j, 21/04/2012, Castro Verde (Herdade da Barrigoa). Ref. **55**-11-a, 1♀, 21/04/2012, Castro Verde (Herdade da Barrigoa). Ref. **56**-10-b, 1♂, 18/04/2012, Castro Verde (Herdade da Barrigoa). Ref. **58**-3-g, 1♂, 07/04/2012, Mértola (Herdade da Benviúda). Ref. **70**-9-f, 1j, 21/04/2012, Castro Verde (Monte dos Janeiros). Ref. **73**-9-j, 1♂, 19/04/2012, Castro Verde (Herdade dos Longos). Ref. **73**-10-h, 1j, 19/04/2012, Castro Verde (Herdade dos Longos). Ref. **79**-2-e, 1j, 07/04/2012, Castro Verde (Monte da Chada). Ref. **79**-5-d, 1♂, 07/04/2012, Castro Verde (Monte da Chada). Ref. **79**-8-c, 2jj, 07/04/2012, Castro Verde (Monte da Chada). Ref. **83**-5-k, 2♂♂, 06/05/2012, Castro Verde (Herdade do Álamo). Ref. **83**-7-h, 1♂, 06/05/2012, Castro Verde (Herdade do Álamo). Ref. **83**-8-f, 1♂, 06/05/2012, Castro Verde (Herdade do Álamo). Ref. **86**-4-c, 1♀, 10/05/2012, Castro Verde (Herdade das Mestras). Ref. **86**-5-b, 1♂, 10/05/2012, Castro Verde (Herdade das Mestras). Ref. **86**-8-e, 1♀, 10/05/2012, Castro Verde (Herdade das Mestras). Ref. **87**-11-a, 1♂ 1♀, 06/05/2012, Castro Verde (Herdade das Mestras). Ref. **88**-8-g, 1♀, 25/04/2012, Castro Verde (Herdade da Portela). Ref. **89**-2-c, 1j, 06/05/2012, Castro Verde (Herdade do Carapetal). Ref. **89**-3-b, 1♀, 06/05/2012, Castro Verde (Herdade do Carapetal). Ref. **89**-4-h, 1♂ 1♀, 06/05/2012, Castro Verde (Herdade do Carapetal). Ref. **93**-8-f, 1♂,

17/04/2012, Aljustrel (Herdade da Sobreira). Ref. **93**-11-c, 1j, 17/04/2012, Aljustrel (Herdade da Sobreira). Ref. **97**-8-c, 1j, 13/04/2012, Castro Verde (Cumeada Nova). Ref. **110**-5-c, 1♂, 04/05/2012, Castro Verde (Monte da Perdigoa). Ref. **113**-3-c, 1j, 10/04/2012, Castro Verde (Courela do Monte Novo). Ref. **113**-9-b, 1♂, 10/04/2012, Castro Verde (Courela do Monte Novo). Ref. **116**-5-d, 1j, 10/04/2012, Castro Verde (Herdade dos Merendeiros). Ref. **121**-7-e, 1♀, 16/04/2012, Castro Verde (Monte do Tacanho). Ref. **123**-2-c, 1j, 20/04/2012, Castro Verde (Herdade das bicadas). Ref. **123**-9-b, 1j, 20/04/2012, Castro Verde (Herdade das bicadas). Ref. **124**-5-a, 1♀, 18/04/2012, Castro Verde (Herdade das Mestras). Ref. **124-6-j**, 1♀, 18/04/2012, Castro Verde (Herdade das Mestras). Ref. **124**-9-g, 1♂, 18/04/2012, Castro Verde (Herdade das Mestras). Ref. **124**-11-f, 1j, 18/04/2012, Castro Verde (Herdade das Mestras). Ref. **125**-8-d, 1♀, 17/04/2012, Aljustrel (Herdade da Sobreira). Ref. **127**-3-d, 1♂, 11/04/2012, Castro Verde (Herdade dos Montinhos). Ref. **127**-8-b, 1j, 11/04/2012, Castro Verde (Herdade dos Montinhos). Ref. **131**-2-f, 1♂, 04/05/2012, Castro Verde (Herdade do Torrejão). Ref. **131**-10-g, 1♂, 04/05/2012, Castro Verde (Herdade do Torrejão). Ref. **132**-10-g, 1♂, 06/05/2012, Castro Verde (Monte das Cabeceiras). Ref. **133**-11-h, 1♂, 28/04/2012, Castro Verde (Monte da Albergaria). Ref. **A46**-11-g, 1♂, 10/05/2012, Aljustrel (Monte da Chaiça). Ref. **A57**-6-c, 1♂, 08/05/2012, Aljustrel (Herdade de Corta Rabos). Ref. **A57**-11-g, 1j, 08/05/2012, Aljustrel (Herdade de Corta Rabos). Ref. **A166**-3-g, 1j, 07/05/2012, Castro Verde (Herdade dos Brunhachos). Ref. **A297**-3-b, 1j, 09/05/2012, Castro Verde (Lagoa da Mó). Ref. **A297**-4-a, 1♂, 09/05/2012, Castro Verde (Lagoa da Mó). Ref. **A297**-7-a, 1♂, 09/05/2012, Castro Verde (Lagoa da Mó). Ref. **A297**-11-f, 1j, 09/05/2012, Castro Verde (Lagoa da Mó). Ref. **A299**-8-j, 1♂ 1j, 08/05/2012, Castro Verde

(Lagoa da Mó). Ref. **A299**-10-f, 1j, 08/05/2012, Castro Verde (Lagoa da Mó). Ref. **A388**-7-g, 1j, 10/05/2012, Castro Verde (Monte da Achada). Ref. **A423**-6-d, 1♀, 08/05/2012, Castro Verde (Herdade da Zibreira). Ref. **A505**-7-f, 2♂♂ 2♀♀, 08/05/2012, Castro Verde (Herdade do Reguengo). Ref. **A527**-4-k, 1j, 07/05/2012, Castro Verde (Herdade dos Bispos). Ref. **A527**-8-g, 1♂, 07/05/2012, Castro Verde (Herdade dos Bispos). Ref. **A527**-10-g, 1♀, 07/05/2012, Castro Verde (Herdade dos Bispos). Ref. **A999**-6-e, 1j, 09/05/2012, Castro Verde (Herdade dos Pereiros). Ref. **A999**-10-b, 1j, 09/05/2012, Castro Verde (Herdade dos Pereiros).

Heliophanus lineiventris **Simon, 1868**

Material examined: Ref. **50**-7-c, 1♀, 16/04/2012, Castro Verde (Herdade do Torrejão); Ref. **55**-5-b, 1♀, 21/04/2012, Castro Verde (Herdade da Barrigoa); Ref. **58**-2-a, 1♂, 07/04/2012, Mértola (Herdade da Benviúda); Ref. **A527**-10-g, 1j, 07/04/2012, Castro Verde (Herdade dos Bispos).

Pellenes nigrociliatus **(Simon, 1875)**

Material examined: Ref. **47**-2-j, 1♀, 09/04/2012, Castro Verde (Herdade do Almarginho); Ref. **A388**-6-e, 1♀, 10/05/2012, Castro Verde (Monte da Achada).

Phlegra bresnieri **(Lucas, 1846)**

Material examined: Ref. **6**-8-h, 1♀, 24/04/2012, Castro Verde (Herdade de A de Neves da Marinha). Ref. **15**-3-b, 1♀, 12/04/2012, Castro Verde (Lagoa da Mó). Ref. **19**-7-d, 1♂, 26/04/2012, Castro Verde (Herdade do Torrejão). Ref. **21**-11-c, 1♂, 21/04/2012, Castro Verde (Herdade da Barrigoa). Ref. **23**-2-a, 1♀,

03/04/2012, Castro Verde (Monte do Vale das Gretas). Ref. **23**-4-a, 1♂, 03/04/2012, Castro Verde (Monte do Vale das Gretas). Ref. **23**-8-c, 1♀, 03/04/2012, Castro Verde (Monte do Vale das Gretas). Ref. **23**-11-i, 1♂, 03/04/2012, Castro Verde (Monte do Vale das Gretas). Ref. **25**-5-a, 1♂, 05/04/2012, Castro Verde (Herdade dos Touris e Rolão). Ref. **34**-6-c, 1♂, 16/04/2012, Castro Verde (Herdade do Tacanho e Merendeiros). Ref. **34**-8-c, 1♀, 16/04/2012, Castro Verde (Herdade do Tacanho e Merendeiros). Ref. **34**-11-d, 1♀, 16/04/2012, Castro Verde (Herdade do Tacanho e Merendeiros). Ref. **43**-3-c, 1♀, 21/04/2012, Castro Verde (Monte da Comenda). Ref. **45**-2-c, 1♀, 13/04/2012, Castro Verde (Monte das Fontes Barbas Velho). Ref. **45**-3-b, 2♀♀, 13/04/2012, Castro Verde (Monte das Fontes Barbas Velho). Ref. **45**-6-b, 1♀, 13/04/2012, Castro Verde (Monte das Fontes Barbas Velho). Ref. **47**-2-j, 1♀, 09/04/2012, Castro Verde (Herdade do Almarginho). Ref. **47**-3-d, 1♀, 09/04/2012, Castro Verde (Herdade do Almarginho). Ref. **49**-3-e, 2♀♀, 25/04/2012, Castro Verde (Herdade da Navarra). Ref. **50**-8-c, 1♂, 16/04/2012, Castro Verde (Herdade do Torrejão). Ref. **51**-2-a, 2♀♀, 13/04/2012, Castro Verde (Herdade do Torrejão). Ref. **51**-11-b, 1♀, 13/04/2012, Castro Verde (Herdade do Torrejão). Ref. **54**-5-c, 1♂, 25/04/2012, Castro Verde (Herdade do Roncanho). Ref. **55**-9-g, 1♀, 21/04/2012, Castro Verde (Herdade da Barrigoa). Ref. **56**-7-d, 1♂, 18/04/2012, Castro Verde (Herdade da Barrigoa). Ref. **57**-1-a, 1♂ 1♀, 06/04/2012, Castro Verde (Monte da Azinheira). Ref. **57**-4-c, 1♀, 06/04/2012, Castro Verde (Monte da Azinheira). Ref. **57**-8-b, 1♀, 06/04/2012, Castro Verde (Monte da Azinheira). Ref. **57**-12-c, 1♂ 1♀, 06/04/2012, Castro Verde (Monte da Azinheira). Ref. **70**-4-a, 1♀, 21/04/2012, Castro Verde (Monte dos Janeiros). Ref. **72**-3-b, 1♀, 15/04/2012, Castro Verde (Cumeada Nova). Ref. **78**-1-b, 1♂,

09/04/2012, Castro Verde (Herdade de São Marcos). Ref. **78**-5-b, 1♀, 09/04/2012, Castro Verde (Herdade de São Marcos). Ref. **78**-9-d, 1♀, 09/04/2012, Castro Verde (Herdade de São Marcos). Ref. **83**-7-h, 1♀, 06/05/2012, Castro Verde (Herdade do Álamo). Ref. **83**-11-a, 1♀, 06/05/2012, Castro Verde (Herdade do Álamo). Ref. **86**-10-a, 1j, 10/05/2012, Castro Verde (Herdade das Mestras). Ref. **88**-4-a, 1♂, 25/04/2012, Castro Verde (Herdade da Portela). Ref. **92**-2-c, 2♀♀, 17/04/2012, Aljustrel (Monte do Carregueiro). Ref. **92**-7-e, 1♀, 17/04/2012, Aljustrel (Monte do Carregueiro). Ref. **92**-9-f, 1♂, 17/04/2012, Aljustrel (Monte do Carregueiro). Ref. **96**-2-e, 1♂, 11/04/2012, Castro Verde (Herdade dos Bispos). Ref. **97**-3-d, 1j, 13/04/2012, Castro Verde (Cumeada Nova). Ref. **97**-9-e, 1♀, 13/04/2012, Castro Verde (Cumeada Nova). Ref. **102**-11-e, 1♀, 15/04/2012, Castro Verde (Monte da Fonte). Ref. **107**-9-a, 1♂, 18/04/2012, Castro Verde (Herdade das Mestras). Ref. **107**-11-f, 1♂, 18/04/2012, Castro Verde (Herdade das Mestras). Ref. **111**-8-c, 1♀, 16/04/2012, Castro Verde (Monte da Perdigoa). Ref. **112**-2-h, 1♀, 20/04/2012, Castro Verde (Monte do Freire). Ref. **112**-5-b, 1♂, 20/04/2012, Castro Verde (Monte do Freire). Ref. **112**-8-**i**, 1♀, 20/04/2012, Castro Verde (Monte do Freire). Ref. **113**-8-b, 1j, 10/04/2012, Castro Verde (Courela do Monte Novo). Ref. **114**-2-i, 1♀, 08/04/2012, Castro Verde (Herdade das Cuchilhas). Ref. **114**-10-a, 1♂, 08/04/2012, Castro Verde (Herdade das Cuchilhas). Ref. **121**-7-e, 1♀, 16/04/2012, Castro Verde (Monte do Tacanho). Ref. **121**-11-a, 1♀, 16/04/2012, Castro Verde (Monte do Tacanho). Ref. **122**-8-d, 1♂, 12/04/2012, Castro Verde (Herdade da Chaiça Velha). Ref. **123**-5-g, 1♂, 20/04/2012, Castro Verde (Herdade das bicadas). Ref. **123**-9-b, 1♀, 20/04/2012, Castro Verde (Herdade das bicadas). Ref. **124**-11-f, 1♂, 18/04/2012, Castro Verde (Herdade das

Mestras). Ref. **125**-3-k, 1♀, 17/04/2012, Aljustrel (Herdade da Sobreira). Ref. **132**-3-j, 1♀, 06/05/2012, Castro Verde (Monte das Cabeceiras). Ref. **132**-8-a, 1♀, 06/05/2012, Castro Verde (Monte das Cabeceiras). Ref. **A57**-10-e, 1♀, 08/05/2012, Aljustrel (Herdade de Corta Rabos). Ref. **A166**-3-g, 1♀, 07/05/2012, Castro Verde (Herdade dos Brunhachos). Ref. **A297**-10-f, 1♂, 09/05/2012, Castro Verde (Lagoa da Mó). Ref. **A299**-11-d, 1♀, 08/05/2012, Castro Verde (Lagoa da Mó). Ref. **A388**-6-e, 1♂, 10/05/2012, Castro Verde (Monte da Achada). Ref. **A423**-8-b, 1♀, 08/05/2012, Castro Verde (Herdade da Zibreira). Ref. **A423**-10-d, 1♀, 08/05/2012, Castro Verde (Herdade da Zibreira).

***Salticus propinquus* Lucas, 1846**

<u>Material examined</u>: Ref. **114**-8-c, 1♀, 08/04/2012, Castro Verde (Herdade das Cuchilhas).

***Talavera petrensis* (C. L. Koch, 1837)**

<u>Material examined</u>: Ref. **43**-8-a, 1♂, 21/04/2012, Castro Verde (Monte da Comenda).

Family SCYTODIDAE Blackwall, 1864

***Scytodes* sp.**

<u>Material examined</u>: Ref. **116**-3c, 1j, 10/04/2012, Castro Verde (Herdade dos Merendeiros).

Family SYNAPHRIDAE Wunderlich, 1986

Synaphris saphrynis Lopardo, Hormiga & Melic, 2007

Material examined: Ref. **4**-10e, 1♂, 15/04/2012, Castro Verde (Heredade das Mouras). Ref. **4**-11b, 1♂, 15/04/2012, Castro Verde (Heredade das Mouras). Ref. **11**-8f, 1♀, 15/04/2012, Castro Verde (Monte do Broco e Capitão). Ref. **14**-10j, 1♀, 18/04/2012, Castro Verde (Lagoa da Mó). Ref. **45**-5a, 1♂, 13/04/2012, Castro Verde (Monte das Fontes Barbas Velho). Ref. **50**-11f, 1♀, 16/04/2012, Castro Verde (Herdade do Torrejão). Ref. **53**-4b, 1♂, 20/04/2012, Castro Verde (Herdade do Reguengo). Ref. **53**-8c, 1♀, 20/04/2012, Castro Verde (Herdade do Reguengo). Ref. **55**-3g, 2♂♂ 1♀, 21/04/2012, Castro Verde (Herdade da Barrigoa). Ref. **55**-5b, 1♀, 21/04/2012, Castro Verde (Herdade da Barrigoa). Ref. **55**-8b, 1♂, 21/04/2012, Castro Verde (Herdade da Barrigoa). Ref. **55**-9g 3♂♂, 21/04/2012, Castro Verde (Herdade da Barrigoa). Ref. **55**-10m1, 1♀, 21/04/2012, Castro Verde (Herdade da Barrigoa). Ref. **56**-4b, 1♂, 18/04/2012, Castro Verde (Herdade da Barrigoa). Ref. **57**-12c, 1♀, 06/04/2012, Castro Verde (Monte da Azinheira). Ref. **61**-2k, 2jj, 05/05/2012, Aljustrel (Monte da Torre). Ref. **61**-3c, 1♂, 05/05/2012, Aljustrel (Monte da Torre). Ref. **61**-7e, 1♂, 05/05/2012, Aljustrel (Monte da Torre). Ref. **70**-5d, 1j, 21/04/2012, Castro Verde (Monte dos Janeiros). Ref. **72**-6a, 1♀, 15/04/2012, Castro Verde (Cumeada Nova) Ref. **72-**7f, 1♀, 15/04/2012, Castro Verde (Cumeada Nova). Ref. **73**-3h, 1♀, 19/04/2012, Castro Verde (Herdade dos Longos). Ref **73**-6c, 1♀, 19/04/2012 Castro Verde (Herdade dos Longos). Ref. **79-**6e, 1♂, 07/04/2012 Castro Verde (Monte da Chada). Ref. **83**-2-g, 1♀, 06/05/2012, Castro Verde (Herdade do Álamo). Ref.**83**-3b, 1♂, 06/05/2012, Castro Verde (Herdade do Álamo). Ref. **83**-4i, 1♂ 1♀, 06/05/2012,

Castro Verde (Herdade do Álamo). Ref. **83**-7h, 1♂, 06/05/2012, Castro Verde (Herdade do Álamo); Ref. **83**-9a, 1♀, 06/05/2012, Castro Verde (Herdade do Álamo). Ref. **87**-5h, 1♀, 06/05/2012, Castro Verde (Herdade das Mestras). Ref. **87**-10c, 1j, 06/05/2012, Castro Verde (Herdade das Mestras). Ref. **89**-3b, 1♀, 06/05/2012, Castro Verde (Herdade do Carapetal). Ref. **89**-5k, 3♂♂ 1♀, 06/05/2012, Castro Verde (Herdade do Carapetal). Ref. **89**-5p1, 1♀, 06/05/2012, Castro Verde (Herdade do Carapetal). Ref. **89**-11h, 1♂ 1♀, 06/05/2012, Castro Verde (Herdade do Carapetal). Ref. **92**-2c, 1♂, 17/04/2012, Aljustrel (Monte do Carregueiro). Ref. **93**-2a, 2♀♀, 17/04/2012, Aljustrel (Herdade da Sobreira). Ref. **93**-5e, 1♀, 17/04/2012, Aljustrel (Herdade da Sobreira). Ref. **93**-6h, 1♀, 17/04/2012, Aljustrel (Herdade da Sobreira). Ref. **93**-7d, 1♂, 17/04/2012, Aljustrel (Herdade da Sobreira). Ref. **93**-9a, 1♂ 1♀, 17/04/2012, Aljustrel (Herdade da Sobreira). Ref. **97**-3d, 1♀, 13/04/2012, Castro Verde (Cumeada Nova). Ref. **102**-3g, 1♂, 15/04/2012, Castro Verde (Monte da Fonte). Ref. **102**-8-h, 1♀, 15/04/2012, Castro Verde (Monte da Fonte). Ref. **110**-2j, 2♂♂ 3jj, 04/05/2012, Castro Verde (Monte da Perdigoa). Ref. **110**-5c, 1♂ 04/05/2012, Castro Verde (Monte da Perdigoa). Ref. **110**-11b, 1♂ 1♀ 04/05/2012, Castro Verde (Monte da Perdigoa). Ref. **125**-2b, 2♂♂, 17/04/2012, Aljustrel (Herdade da Sobreira). Ref. **125**-4k, 2♂♂ 1♀, 17/04/2012, Aljustrel e (Herdade da Sobreira). Ref. **125**-9h, 2♂♂, 17/04/2012, Aljustrel e (Herdade da Sobreira). Ref. **A46**-7c2, 1j, 10/05/2012, Aljustrel (Monte da Chaiça). Ref. **A46**-10g, 1♂, 10/05/2012, Aljustrel (Monte da Chaiça). Ref. **A297**-5a, 1♀, Castro Verde (Lagoa da Mó). Ref. **A297**-7a, 1♂, Castro Verde (Lagoa da Mó). Ref. **A297**-10f, 1♂ 1j, Castro Verde (Lagoa da Mó). Ref. **A299**-2c, 2jj, 08/05/2012, Castro Verde (Lagoa da Mó). Ref. **A299**-4d, 1j, 08/05/2012, Castro Verde (Lagoa da Mó). Ref. **A299**-7d, 1j,

08/05/2012, Castro Verde (Lagoa da Mó). Ref. **A349**-11d, 1♂, 10/05/2012, Castro Verde (Herdade das Mestras). Ref. **A388**-9c, 1♀, 10/05/2012, Castro Verde (Monte da Achada). Ref. **A423**-2j, 1j, 08/05/2012, Castro Verde (Herdade da Zibreira). Ref. **A423**-4i, 2jj, 08/05/2012, Castro Verde (Herdade da Zibreira). Ref. **A423**-7d, 2jj, 08/05/2012, Castro Verde (Herdade da Zibreira). Ref. **A423**-9a, 1j, 08/05/2012, Castro Verde (Herdade da Zibreira). Ref.**A423**-11f, 1j, 08/05/2012, Castro Verde (Herdade da Zibreira). Ref. **A999**-3b, 1♂ 1♀, 09/05/2012, Castro Verde (Herdade dos Pereiros); Ref. **A999**-8j, 1♂, 09/05/2012, Castro Verde (Herdade dos Pereiros); Ref. **A999**-10b, 1♂, 09/05/2012, Castro Verde (Herdade dos Pereiros).

Family TETRAGNATHIDAE Menge, 1866

Tetragnatha intermedia **Kulczyński, 1891**

Material examined: Ref. **36**-3a, 1♂, 1♀, 11/04/2012, Castro Verde (Herdade dos Bispos).

Family THERIDIIDAE Sundevall, 1833

Theridiidae sp.; (7 jj. indet.).

Asagena phalerata **(Panzer, 1801)**

Material examined: Ref. **6**-8h, 1♀, 24/04/2012, Castro Verde (Herdade de A de Neves da Marinha). Ref. **27**-4e, 1♀, 24/04/2012, Castro Verde (Herdade das Sesmarias). Ref. **27**-8c, 1♀, 24/04/2012, Castro Verde (Herdade das Sesmarias).

Ref. **47**-1a, 1♀, 09/04/2012, Castro Verde (Herdade do Almarginho). Ref. **47**-2j, 1♀, 09/04/2012, Castro Verde (Herdade do Almarginho). Ref. **47**-6c 1j, 09/04/2012, Castro Verde (Herdade do Almarginho). Ref. **47**-10c, 1♀, 09/04/2012, Castro Verde (Herdade do Almarginho). Ref. **50**-5d, 1♀, 16/04/2012, Castro Verde (Herdade do Torrejão). Ref. **73**-7a, 1j, 19/04/2012, Castro Verde (Herdade dos Longos). Ref. **86**-4c, 1♀, 10/05/2012, Castro Verde (Herdade das Mestras). Ref. **87**-7g, 1♂, 06/05/2012, Castro Verde (Herdade das Mestras). Ref. **96**-10i, 1j, 11/04/2012, Castro Verde (Herdade dos Bispos). Ref. **97**-3d, 1♀, 13/04/2012, Castro Verde (Cumeada Nova). Ref. **122**-9b, 1♀, 12/04/2012, Castro Verde (Herdade da Chaiça Velha). Ref. **124**-4i, 1♀, 18/04/2012, Castro Verde (Herdade das Mestras). Ref. **124**-5a, 1♀, 18/04/2012, Castro Verde (Herdade das Mestras). Ref. **124**-6j, 1♀, 18/04/2012, Castro Verde (Herdade das Mestras). Ref. **125**-8d, 1j, 17/04/2012, Aljustrel (Herdade da Sobreira). Ref. **125**-11f, 1♂, 1♀, 17/04/2012, Aljustrel (Herdade da Sobreira). Ref. **128**-4b, 1♀, 04/04/2012, Castro Verde (Herdade de Carriça-Viseus). Ref. **128**-12a, 1♀, 04/04/2012, Castro Verde (Herdade de Carriça-Viseus). Ref. **129**-9b, 1♀, 08/05/2012, Castro Verde (Herdade da Zibreira). Ref. **A46**-5c, 1♀, 10/05/2012, Aljustrel (Monte da Chaiça). Ref. **A46**-9f, 1♂, 10/05/2012, Aljustrel (Monte da Chaiça). Ref. **A297**-4a, 1♀, 09/05/2012, Castro Verde (Lagoa da Mó). Ref. **A297**-9c, 2♂♂, 2♀♀, 1j, 09/05/2012, Castro Verde (Lagoa da Mó). Ref. **A388**-6e, 1♀, 10/05/2012, Castro Verde (Monte da Achada). Ref. **A423**-10d, 1♀, 08/05/2012, Castro Verde (Herdade da Zibreira).

***Dipoena umbratilis* (Simon, 1873)**

Material examined: Ref. **11**-11e, 1♂, 15/04/2012, Castro Verde (Monte do Broco e Capitão). Ref. **35**-8d, 1j, 05/04/2012, Castro Verde (Monte do Seixo). Ref. **43**-10h, 1j, 21/04/2012, Castro Verde (Monte da Comenda). Ref. **50**-10c, 1j, 16/04/2012, Castro Verde (Herdade do Torrejão). Ref. **112**-8i, 1♂, 20/04/2012, Castro Verde (Monte do Freire). Ref. **128**-1b, 1j, 04/04/2012, Castro Verde (Herdade de Carriça-Viseus).

***Enoplognatha diversa* (Blackwall, 1859)**

Material examined: Ref. **25**-10b, 1♀, 05/04/2012, Castro Verde (Herdade dos Touris e Rolão). Ref. **47**-2j, 1♀, 09/04/2012, Castro Verde (Herdade do Almarginho). Ref. **53**-11e, 1♂, 20/04/2012, Castro Verde (Herdade do Reguengo). Ref. **56**-11b, 1♂, 1♀, 18/04/2012, Castro Verde (Herdade da Barrigoa). Ref. **129**-4a, 1♀, 08/05/2012, Castro Verde (Herdade da Zibreira).

***Euryopis episinoides* (Walckenaer, 1847)**

Material examined: Ref. **54**-11c, 1j, 25/04/2012, Castro Verde (Herdade do Roncanho). Ref. **83**-5k, 1j, 06/05/2012, Castro Verde (Herdade do Álamo). Ref. **127**-8b, 1♂, 1j, 11/04/2012, Castro Verde (Herdade dos Montinhos).

***Neottiura uncinata* (Lucas, 1846)**

Material examined: Ref. **4**-6-c, 1j, 15/04/2012, Castro Verde (Heredade das Mouras). Ref. **6**-3-c, 2jj, 24/04/2012, Castro Verde (Herdade de A de Neves da Marinha). Ref. **6**-4-e, 1j, 24/04/2012, Castro Verde (Herdade de A de Neves da Marinha). Ref. **6**-8-h, 4jj, 24/04/2012, Castro Verde (Herdade de A de Neves da Marinha). Ref. **6**-9-h, 2jj, 24/04/2012, Castro Verde (Herdade de A de Neves da Marinha).

Ref. **6**-10-e, 1j, 24/04/2012, Castro Verde (Herdade de A de Neves da Marinha). Ref. **6**-11-b, 1j, 24/04/2012, Castro Verde (Herdade de A de Neves da Marinha). Ref. **9**-9-d, 1j, 25/04/2012, Castro Verde (COSA - Herdade de São Marcos). Ref. **11**-7-c, 2jj, 15/04/2012, Castro Verde (Monte do Broco e Capitão). Ref. **11**-11-e, 2jj, 15/04/2012, Castro Verde (Monte do Broco e Capitão). Ref. **14**-8-c, 1♂, 18/04/2012, Castro Verde (Lagoa da Mó). Ref. **14**-10-j, 1j, 18/04/2012, Castro Verde (Lagoa da Mó). Ref. **15**-2-h, 1j, 12/04/2012, Castro Verde (Lagoa da Mó). Ref. **15**-3-b, 6jj, 12/04/2012, Castro Verde (Lagoa da Mó). Ref. **15**-4-e, 2jj, 12/04/2012, Castro Verde (Lagoa da Mó). Ref. **15**-5-b, 3jj, 12/04/2012, Castro Verde (Lagoa da Mó). Ref. **15**-7-e, 1j, 12/04/2012, Castro Verde (Lagoa da Mó). Ref. **15**-8-a, 3jj, 12/04/2012, Castro Verde (Lagoa da Mó). Ref. **19**-4-d, 1j, 26/04/2012, Castro Verde (Herdade do Torrejão). Ref. **21**-4-f, 1j, 21/04/2012, Castro Verde (Herdade da Barrigoa). Ref. **21**-5-f, 1j, 21/04/2012, Castro Verde (Herdade da Barrigoa). Ref. **21**-10-a, 1j, 21/04/2012, Castro Verde (Herdade da Barrigoa). Ref. **23**-1-a, 3jj, 03/04/2012, Castro Verde (Monte do Vale das Gretas). Ref. **23**-3-c, 1j, 03/04/2012, Castro Verde (Monte do Vale das Gretas). Ref. **23**-10-k, 1j, 03/04/2012, Castro Verde (Monte do Vale das Gretas). Ref. **23**-11-i, 1j, 03/04/2012, Castro Verde (Monte do Vale das Gretas). Ref. **25**-2-d, 1j, 05/04/2012, Castro Verde (Herdade dos Touris e Rolão). Ref. **25**-6-i, 1j, 05/04/2012, Castro Verde (Herdade dos Touris e Rolão). Ref. **25**-7-a, 1j, 05/04/2012, Castro Verde (Herdade dos Touris e Rolão). Ref. **25**-9-c, 1j, 05/04/2012, Castro Verde (Herdade dos Touris e Rolão). Ref. **25**-11-a, 1j, 05/04/2012, Castro Verde (Herdade dos Touris e Rolão). Ref. **27**-2-c, 1j, 24/04/2012, Castro Verde (Herdade das Sesmarias). Ref. **27**-3-d, 1♂ 4jj, 24/04/2012, Castro Verde (Herdade das Sesmarias). Ref. **27**-4-e, 2jj, 24/04/2012,

Castro Verde (Herdade das Sesmarias). Ref. **27**-5-d, 4jj, 24/04/2012, Castro Verde (Herdade das Sesmarias). Ref. **27**-6-e, 1j, 24/04/2012, Castro Verde (Herdade das Sesmarias). Ref. **27**-7-d, 2jj, 24/04/2012, Castro Verde (Herdade das Sesmarias). Ref. **27**-10-d, 3jj, 24/04/2012, Castro Verde (Herdade das Sesmarias). Ref. **35**-5-f, 1j, 05/04/2012, Castro Verde (Monte do Seixo). Ref. **36**-4-e, 3jj, 11/04/2012, Castro Verde (Herdade dos Bispos). Ref. **36**-5-a, 3jj, 11/04/2012, Castro Verde (Herdade dos Bispos). Ref. **36**-6-h, 4jj, 11/04/2012, Castro Verde (Herdade dos Bispos). Ref. **36**-7-d, 1j, 11/04/2012, Castro Verde (Herdade dos Bispos). Ref. **36**-8-i, 3jj, 11/04/2012, Castro Verde (Herdade dos Bispos). Ref. **36**-10-g, 1j, 11/04/2012, Castro Verde (Herdade dos Bispos). Ref. **37**-7-d, 1j, 09/04/2012, Castro Verde (Herdade de São Marcos). Ref. **43**-2-d, 3jj, 21/04/2012, Castro Verde (Monte da Comenda). Ref. **43**-3-c, 1j, 21/04/2012, Castro Verde (Monte da Comenda). Ref. **43**-4-d, 4jj, 21/04/2012, Castro Verde (Monte da Comenda). Ref. **43**-5-e, 2jj, 21/04/2012, Castro Verde (Monte da Comenda). Ref. **43**-6-b, 1♂ 3jj, 21/04/2012, Castro Verde (Monte da Comenda). Ref. **43**-7-b, 2jj, 21/04/2012, Castro Verde (Monte da Comenda). Ref. **43**-10-h, 2jj, 21/04/2012, Castro Verde (Monte da Comenda). Ref. **43**-11-h, 1j, 21/04/2012, Castro Verde (Monte da Comenda). Ref. **45**-2-c, 1j, 13/04/2012, Castro Verde (Monte das Fontes Barbas Velho). Ref. **45**-3-b, 2jj, 13/04/2012, Castro Verde (Monte das Fontes Barbas Velho). Ref. **45**-5-a, 1♀, 13/04/2012, Castro Verde (Monte das Fontes Barbas Velho). Ref. **45**-8-b, 13/04/2012, Castro Verde (Monte das Fontes Barbas Velho). Ref. **47**-1-a, 4jj, 09/04/2012, Castro Verde (Herdade do Almarginho). Ref. **47**-2-j, 1j, 09/04/2012, Castro Verde (Herdade do Almarginho). Ref. **47**-9-i, 2jj, 09/04/2012, Castro Verde (Herdade do Almarginho). Ref. **47**-12-c, 1j, 09/04/2012, Castro Verde (Herdade do Almarginho). Ref. **49**-5-

a, 1♂ 1♀, 25/04/2012, Castro Verde (Herdade da Navarra). Ref. **49**-6-a, 1j, 25/04/2012, Castro Verde (Herdade da Navarra). Ref. **49**-7-c, 1j, 25/04/2012, Castro Verde (Herdade da Navarra). Ref. **49**-8-d, 1♂ 1♀, 25/04/2012, Castro Verde (Herdade da Navarra). Ref. **49**-9-a, 1♀, 25/04/2012, Castro Verde (Herdade da Navarra). Ref. **49**-10-b, 1♂ 1♀, 25/04/2012, Castro Verde (Herdade da Navarra). Ref. **50**-3-f, 1j, 16/04/2012, Castro Verde (Herdade do Torrejão). Ref. **50**-6-c, 1j, 16/04/2012, Castro Verde (Herdade do Torrejão). Ref. **50**-10-c, 3jj, 16/04/2012, Castro Verde (Herdade do Torrejão). Ref. **51**-8-c, 1j, 13/04/2012, Castro Verde (Herdade do Torrejão). Ref. **51**-10-c, 2jj, 13/04/2012, Castro Verde (Herdade do Torrejão). Ref. **53**-2-d, 1j, 20/04/2012, Castro Verde (Herdade do Reguengo). Ref. **53**-4-b, 2jj, 20/04/2012, Castro Verde (Herdade do Reguengo). Ref. **53**-6-d, 2jj, 20/04/2012, Castro Verde (Herdade do Reguengo). Ref. **53**-8-c, 2jj, 20/04/2012, Castro Verde (Herdade do Reguengo). Ref. **53**-9-b, 1j, 20/04/2012, Castro Verde (Herdade do Reguengo). Ref. **53**-11-e, 1j, 20/04/2012, Castro Verde (Herdade do Reguengo). Ref. **55**-3-g, 3jj, 21/04/2012, Castro Verde (Herdade da Barrigoa). Ref. **55**-5-b, 2jj, 21/04/2012, Castro Verde (Herdade da Barrigoa). Ref. **55**-6-e, 2jj, 21/04/2012, Castro Verde (Herdade da Barrigoa). Ref. **55**-7-b, 3jj, 21/04/2012, Castro Verde (Herdade da Barrigoa). Ref. **55**-9-g, 1j, 21/04/2012, Castro Verde (Herdade da Barrigoa). Ref. **55**-11-a, 2jj, 21/04/2012, Castro Verde (Herdade da Barrigoa). Ref. **56**-2-h, 1j, 18/04/2012, Castro Verde (Herdade da Barrigoa). Ref. **56**-4-b, 1j, 18/04/2012, Castro Verde (Herdade da Barrigoa). Ref. **56**-9-f, 1j, 18/04/2012, Castro Verde (Herdade da Barrigoa). Ref. **56**-10-b, 4jj, 18/04/2012, Castro Verde (Herdade da Barrigoa). Ref. **57-6-b**, 1j, 06/04/2012, Castro Verde (Monte da Azinheira). Ref. **61**-2-k, 1♂ 1j, 05/05/2012, Aljustrel (Monte da Torre). Ref. **61**-3-c, 1j, 05/05/2012, Aljustrel (Monte da Torre).

Ref. **61**-7-e, 1♂ 1♀, 05/05/2012, Aljustrel (Monte da Torre). Ref. **61**-9-f, 1j, 05/05/2012, Aljustrel (Monte da Torre). Ref. **67**-6-i, 1j, 12/04/2012, Aljustrel (Monte da Chaiça). Ref. **67**-9-c, 1j, 12/04/2012, Aljustrel (Monte da Chaiça). Ref. **70**-6-e, 1j, 21/04/2012, Castro Verde (Monte dos Janeiros). Ref. **70**-7-a, 2jj, 21/04/2012, Castro Verde (Monte dos Janeiros). Ref. **70**-8-f, 1j, 21/04/2012, Castro Verde (Monte dos Janeiros). Ref. **70**-10-a, 1j, 21/04/2012, Castro Verde (Monte dos Janeiros). Ref. **70**-11-g, 2jj, 21/04/2012, Castro Verde (Monte dos Janeiros). Ref. **72**-2-e, 4jj, 15/04/2012, Castro Verde (Cumeada Nova). Ref. **72**-4-c, 2jj, 15/04/2012, Castro Verde (Cumeada Nova). Ref. **72**-10-d, 1j, 15/04/2012, Castro Verde (Cumeada Nova). Ref. **73**-2-c, 2jj, 19/04/2012, Castro Verde (Herdade dos Longos). Ref. **73**-3-h, 1j, 19/04/2012, Castro Verde (Herdade dos Longos). Ref. **73**-7-a, 1j, 19/04/2012, Castro Verde (Herdade dos Longos). Ref. **73**-9-j, 2jj, 19/04/2012, Castro Verde (Herdade dos Longos). Ref. **73**-10-h, 1j, 19/04/2012, Castro Verde (Herdade dos Longos). Ref. **78**-10-c, 1j, 09/04/2012, Castro Verde (Herdade de São Marcos). Ref. **79**-3-h, 1j, 07/04/2012, Castro Verde (Monte da Chada). Ref. **79**-7-g, 1j, 07/04/2012, Castro Verde (Monte da Chada). Ref. **79**-8-c, 1j, 07/04/2012, Castro Verde (Monte da Chada). Ref. **83**-4-i, 1♀, 06/05/2012, Castro Verde (Herdade do Álamo). Ref. **83**-6-d, 1♂ 1♀, 06/05/2012, Castro Verde (Herdade do Álamo). Ref. **83**-9-a, 1♂ 1♀, 06/05/2012, Castro Verde (Herdade do Álamo). Ref. **83**-10-f, 2♀♀, 06/05/2012, Castro Verde (Herdade do Álamo). Ref. **86**-2-a, 1j, 10/05/2012, Castro Verde (Herdade das Mestras). Ref. **86**-4-c, 2♀♀, 10/05/2012, Castro Verde (Herdade das Mestras). Ref. **86**-6-g, 1♀ 1j, 10/05/2012, Castro Verde (Herdade das Mestras). Ref. **86**-9-h, 1j, 10/05/2012, Castro Verde (Herdade das Mestras). Ref. **87**-4-h, 3jj, 06/05/2012, Castro Verde (Herdade das Mestras). Ref. **87**-9-a, 1j, 06/05/2012,

Castro Verde (Herdade das Mestras). Ref. **87**-11-a, 1j, 06/05/2012, Castro Verde (Herdade das Mestras). Ref. **88**-10-g, 1j, 25/04/2012, Castro Verde (Herdade da Portela). Ref. **89**-4-h, 1j, 06/05/2012, Castro Verde (Herdade do Carapetal). Ref. **89**-5-k, 1♂, 06/05/2012, Castro Verde (Herdade do Carapetal). Ref. **89**-6-g, 1♀, 06/05/2012, Castro Verde (Herdade do Carapetal). Ref. **89**-7-e, 1j, 06/05/2012, Castro Verde (Herdade do Carapetal). Ref. **89**-9-h, 2♀♀, 06/05/2012, Castro Verde (Herdade do Carapetal). Ref. **89**-11-h, 2jj, 06/05/2012, Castro Verde (Herdade do Carapetal). Ref. **93**-4-i, 1j, 17/04/2012, Aljustrel (Herdade da Sobreira). Ref. **93**-5-e, 2jj, 17/04/2012, Aljustrel (Herdade da Sobreira). Ref. **93**-10-a, 1j, 17/04/2012, Aljustrel (Herdade da Sobreira). Ref. **96**-2-e, 2jj, 09/05/2012, Castro Verde (Herdade dos Bispos). Ref. **96**-4-c, 1j, 09/05/2012, Castro Verde (Herdade dos Bispos). Ref. **96**-6-b, 1j, 09/05/2012, Castro Verde Herdade dos Bispos). Ref. **96**-8-a, 3jj, 09/05/2012, Castro Verde (Herdade dos Bispos). Ref. **96**-10-i, 1j, 09/05/2012, Castro Verde (Herdade dos Bispos). Ref. **96**-11-c, 6jj, 09/05/2012, Castro Verde (Herdade dos Bispos). Ref. **97**-2-g, 1j, 13/04/2012, Castro Verde (Cumeada Nova). Ref. **97**-5-c, 2jj, 13/04/2012, Castro Verde (Cumeada Nova). Ref. **97**-7-c, 1j, 13/04/2012, Castro Verde (Cumeada Nova). Ref. **97**-8-c, 4jj, 13/04/2012, Castro Verde (Cumeada Nova). Ref. **97**-9-e, 1j, 13/04/2012, Castro Verde (Cumeada Nova). Ref. **97**-10-i, 1j, 13/04/2012, Castro Verde (Cumeada Nova). Ref. **97**-11-a, 1j, 13/04/2012, Castro Verde (Cumeada Nova). Ref. **104**-4-c, 1j, 04/04/2012, Castro Verde (Herdade dos Touris e Rolão). Ref. **104**-9-d, 1j, 09/05/2012, Castro Verde (Herdade dos Touris e Rolão). Ref. **104**-10-e, 1j, 09/05/2012, Castro Verde (Herdade dos Touris e Rolão). Ref. **104**-12-a, 1j, 09/05/2012, Castro Verde (Herdade dos Touris e Rolão). Ref. **108**-2-d, 1j, 10/04/2012, Castro Verde (Herdade dos Merendeiros). Ref. **108**-3-l, 2jj,

10/04/2012, Castro Verde (Herdade dos Merendeiros). Ref. **108**-5-a, 2jj, 10/04/2012, Castro Verde (Herdade dos Merendeiros). Ref. **108**-6-e, 2jj, 10/04/2012, Castro Verde (Herdade dos Merendeiros). Ref. **108**-7-g, 1j, 10/04/2012, Castro Verde (Herdade dos Merendeiros). Ref. **108**-11-a, 2jj, 10/04/2012, Castro Verde (Herdade dos Merendeiros). Ref. **109**-3-i, 1j, 24/04/2012, Castro Verde (Monte da Achada). Ref. **109**-4-b, 1j, 24/04/2012, Castro Verde Monte da Achada). Ref. **109**-9-e, 2♂♂, 24/04/2012, Castro Verde (Monte da Achada). Ref. **109**-10-g, 1♂, 24/04/2012, Castro Verde (Monte da Achada). Ref. **109**-11-h, 1j, 24/04/2012, Castro Verde (Monte da Achada). Ref. **110**-3-k, 1♂, 04/05/2012, Castro Verde (Monte da Perdigoa). Ref. **110**-5-c, 1♀, 04/05/2012, Castro Verde (Monte da Perdigoa). Ref. **110**-6-a, 1j, 04/05/2012, Castro Verde (Monte da Perdigoa). Ref. **110**-8-e, 2♀♀, 04/05/2012, Castro Verde (Monte da Perdigoa). Ref. **110**-11-b, 1j, 04/05/2012, Castro Verde (Monte da Perdigoa). Ref. **112**-8-i, 1j, 20/04/2012, Castro Verde (Monte do Freire). Ref. **112**-9-k, 1j, 20/04/2012, Castro Verde (Monte do Freire). Ref. **112**-10-g, 1j, 20/04/2012, Castro Verde (Monte do Freire). Ref. **113**-4-j, 1♀, 10/04/2012, Castro Verde (Courela do Monte Novo). Ref. **113**-5-f, 1j, 10/04/2012, Castro Verde (Courela do Monte Novo). Ref. **114**-1-c, 1j, 08/04/2012, Castro Verde (Herdade das Cuchilhas). Ref. **114**-10-a, 1j, 08/04/2012, Castro Verde (Herdade das Cuchilhas). Ref. **114**-11-d, 1j, 08/04/2012, Castro Verde (Herdade das Cuchilhas). Ref. **116**-5-d, 1j, 10/04/2012, Castro Verde (Herdade dos Merendeiros). Ref. **121**-2-d, 1j, 16/04/2012, Castro Verde (Monte do Tacanho). Ref. **121**-3-j, 1j, 16/04/2012, Castro Verde (Monte do Tacanho). Ref. **121**-7-e, 1j, 16/04/2012, Castro Verde (Monte do Tacanho). Ref. **121**-8-b, 1j, 16/04/2012, Castro Verde (Monte do Tacanho). Ref. **121**-10-a, 1♂ 1j, 16/04/2012, Castro

Verde (Monte do Tacanho). Ref. **122**-2-d, 1j, 12/04/2012, Castro Verde (Herdade da Chaiça Velha). Ref. **122**-4-i, 2jj, 12/04/2012, Castro Verde (Herdade da Chaiça Velha). Ref. **122**-6-d, 4jj, 12/04/2012, Castro Verde (Herdade da Chaiça Velha). Ref. **122**-7-c, 1j, 12/04/2012, Castro Verde (Herdade da Chaiça Velha). Ref. **122**-10-e, 1j, 12/04/2012, Castro Verde (Herdade da Chaiça Velha). Ref. **122**-11-a, 1j, 12/04/2012, Castro Verde (Herdade da Chaiça Velha). Ref. **123**-5-g, 2jj, 20/04/2012, Castro Verde (Herdade das bicadas). Ref. **123**-6-c, 1j, 20/04/2012, Castro Verde (Herdade das bicadas). Ref. **123**-8-h, 2jj, 20/04/2012, Castro Verde (Herdade das bicadas). Ref. **123**-9-b, 1j, 20/04/2012, Castro Verde (Herdade das bicadas). Ref. **123**-10-a, 1♂ 1j, 20/04/2012, Castro Verde (Herdade das bicadas). Ref. **123**-11-c, 1j, 20/04/2012, Castro Verde (Herdade das bicadas). Ref. **124**-2-b, 2jj, 18/04/2012, Castro Verde (Herdade das Mestras). Ref. **124**-3-a, 1j, 18/04/2012, Castro Verde (Herdade das Mestras). Ref. **124**-4-i, 1j, 18/04/2012, Castro Verde (Herdade das Mestras). Ref. **124**-5-a, 2jj, 18/04/2012, Castro Verde (Herdade das Mestras). Ref. **124**-6-j, 1j, 18/04/2012, Castro Verde (Herdade das Mestras). Ref. **124**-7-i, 2jj, 18/04/2012, Castro Verde (Herdade das Mestras). Ref. **124**-8-d, 1♂ 2jj, 18/04/2012, Castro Verde (Herdade das Mestras). Ref. **124**-9-g, 2jj, 18/04/2012, Castro Verde (Herdade das Mestras). Ref. **124**-10-b, 4jj, 18/04/2012, Castro Verde (Herdade das Mestras). Ref. **124**-11-f, 4jj, 18/04/2012, Castro Verde (Herdade das Mestras). Ref. **125**-4-k, 2jj, 17/04/2012, Aljustrel (Herdade da Sobreira). Ref. **125**-7-f, 3jj, 17/04/2012, Aljustrel (Herdade da Sobreira). Ref. **125**-8-d, 2jj, 17/04/2012, Aljustrel (Herdade da Sobreira). Ref. **125**-10-k, 1j, 17/04/2012, Aljustrel (Herdade da Sobreira). Ref. **126**-1-f, 1j, 05/04/2012, Castro Verde (Monte do Seixo). Ref. **126**-6-a, 1j, 05/04/2012, Castro Verde (Monte do Seixo). Ref. **126**-7-b, 1j, 05/04/2012, Castro

Verde (Monte do Seixo). Ref. **126**-8-f, 1j, 05/04/2012, Castro Verde (Monte do Seixo). Ref. **127**-3-d, 6jj, 11/04/2012, Castro Verde (Herdade dos Montinhos). Ref. **127**-4-a, 4jj, 11/04/2012, Castro Verde (Herdade dos Montinhos). Ref. **127**-5-b, 3jj, 11/04/2012, Castro Verde (Herdade dos Montinhos). Ref. **127**-7-i, 1j, 11/04/2012, Castro Verde (Herdade dos Montinhos). Ref. **127**-8-b, 2♂♂ 4jj, 11/04/2012, Castro Verde (Herdade dos Montinhos). Ref. **127**-9-f, 3jj, 11/04/2012, Castro Verde (Herdade dos Montinhos). Ref. **127**-10-a, 1j, 11/04/2012, Castro Verde (Herdade dos Montinhos). Ref. **127**-11-e, 1j, 11/04/2012, Castro Verde (Herdade dos Montinhos). Ref. **128**-1-b, 1j, 04/04/2012, Castro Verde (Herdade de Carriça-Viseus). Ref. **128**-3-b, 2jj, 04/04/2012, Castro Verde (Herdade de Carriça-Viseus). Ref. **128**-5-b, 1j, 04/04/2012, Castro Verde (Herdade de Carriça-Viseus). Ref. **128**-8-f, 6jj, 04/04/2012, Castro Verde (Herdade de Carriça-Viseus). Ref. **128**-12-a, 2jj, 04/04/2012, Castro Verde (Herdade de Carriça-Viseus). Ref. **129**-6-d, 1j, 08/05/2012, Castro Verde (Herdade da Zibreira). Ref. **129**-10-e, 1j, 08/05/2012, Castro Verde (Herdade da Zibreira). Ref. **129**-11-g, 1♀, 08/05/2012, Castro Verde (Herdade da Zibreira). Ref. **132**-6-c, 1♀, 06/05/2012, Castro Verde (Monte das Cabeceiras). Ref. **132**-7-b, 2♀♀, 06/05/2012, Castro Verde (Monte das Cabeceiras). Ref. **132**-8-a, 1♂ 2♀♀, 06/05/2012, Castro Verde (Monte das Cabeceiras). Ref. **132**-11-b, 1♀, 06/05/2012, Castro Verde (Monte das Cabeceiras). Ref. **A46**-3-h, 1♂, 10/05/2012, Aljustrel (Monte da Chaiça). Ref. **A46**-9-f, 1♀, 10/05/2012, Aljustrel (Monte da Chaiça). Ref. **A57**-4-h, 1♀, 08/05/2012, Aljustrel (Herdade de Corta Rabos). Ref. **A57**-5-d, 2♀♀, 08/05/2012, Aljustrel (Herdade de Corta Rabos). Ref. **A57**-11-g, 1♂, 08/05/2012, Aljustrel (Herdade de Corta Rabos). Ref. **A166**-3-g, 1♀, 07/05/2012, Castro Verde (Herdade dos Brunhachos). Ref. **A166**-6-b, 1♂ 1♀, 07/05/2012, Castro Verde

(Herdade dos Brunhachos). Ref. **A166**-9-c, 1♀, 07/05/2012, Castro Verde (Herdade dos Brunhachos). Ref. **A166**-10-b, 1♀, 07/05/2012, Castro Verde (Herdade dos Brunhachos). Ref. **A260**-6-g, 1♀, 07/05/2012, Castro Verde (Herdade de Reidias). Ref. **A260**-11-g, 1♂ 1♀, 07/05/2012, Castro Verde (Herdade de Reidias). Ref. **A297**-5-a, 2♀♀, 09/05/2012, Castro Verde (Lagoa da Mó). Ref. **A297**-7-a, 1♀, 09/05/2012, Castro Verde (Lagoa da Mó). Ref. **A297**-8-j, 1♂ 2♀♀ 1j, 09/05/2012, Castro Verde (Lagoa da Mó). Ref. **A297**-9-c, 1♂ 2♀♀ 1j, 09/05/2012, Castro Verde (Lagoa da Mó). Ref. **A297**-10-f, 1♀, 09/05/2012, Castro Verde (Lagoa da Mó). Ref. **A297**-11-f, 1♂, 09/05/2012, Castro Verde (Lagoa da Mó). Ref. **A299**-2-c, 1♀, 08/05/2012, Castro Verde (Lagoa da Mó). Ref. **A299**-3-i, 1♂ 1♀, 08/05/2012, Castro Verde (Lagoa da Mó). Ref. **A299**-4-d, 2♀♀, 08/05/2012, Castro Verde (Lagoa da Mó). Ref. **A299**-6-d, 1♂ 2♀♀, 08/05/2012, Castro Verde (Lagoa da Mó). Ref. **A299**-7-d, 1♂ 1♀ 1j, 08/05/2012, Castro Verde (Lagoa da Mó). Ref. **A299**-8-j, 1j, 08/05/2012, Castro Verde (Lagoa da Mó). Ref. **A299**-9-b, 1♀, 08/05/2012, Castro Verde (Lagoa da Mó). Ref. **A299**-11-d, 2jj, 08/05/2012, Castro Verde (Lagoa da Mó). Ref. **A349**-5-a, 1♂ 1j, 10/05/2012, Castro Verde (Herdade das Mestras). Ref. **A349**-7-a, 1♀, 10/05/2012, Castro Verde (Herdade das Mestras). Ref. **A349**-9-g, 1♀, 10/05/2012, Castro Verde (Herdade das Mestras). Ref. **A349**-10-d, 1j, 10/05/2012, Castro Verde (Herdade das Mestras). Ref. **A388**-3-c, 1♀, 10/05/2012, Castro Verde (Monte da Achada). Ref. **A423**-2-j, 2♀♀, 08/05/2012, Castro Verde (Herdade da Zibreira). Ref. **A423**-3-i, 1♀, 08/05/2012, Castro Verde (Herdade da Zibreira). Ref. **A423**-4-i, 2♂♂ 1♀, 08/05/2012, Castro Verde (Herdade da Zibreira). Ref. **A423**-6-d, 1♂ 2♀♀ 1j, 08/05/2012, Castro Verde (Herdade da Zibreira). Ref. **A423**-7-d, 2♀♀ 1j, 08/05/2012, Castro Verde

(Herdade da Zibreira). Ref. **A423**-9-a, 1♂ 1♀, 08/05/2012, Castro Verde (Herdade da Zibreira). Ref. **A423**-10-d, 1j, 08/05/2012, Castro Verde (Herdade da Zibreira). Ref. **A505**-5-e, 1j, 08/05/2012, Castro Verde (Herdade do Reguengo). Ref. **A505**-6-b, 1j, 08/05/2012, Castro Verde (Herdade do Reguengo). Ref. **A505**-7-f, 2♂♂ 2jj, 08/05/2012, Castro Verde (Herdade do Reguengo). Ref. **A505**-10-g, 1♂, 08/05/2012, Castro Verde (Herdade do Reguengo). Ref. **A505**-11-c, 1♂ 1j, 08/05/2012, Castro Verde (Herdade do Reguengo). Ref. **A527**-2-e, 1♀, 07/05/2012, Castro Verde (Herdade dos Bispos). Ref. **A527**-8-g, 1♂ 1j, 07/05/2012, Castro Verde (Herdade dos Bispos). Ref. **A527**-10-g, 1j, 07/05/2012, Castro Verde (Herdade dos Bispos). Ref. **A999**-2-j, 1♂, 09/05/2012, Castro Verde (Herdade dos Pereiros). Ref. **A999**-5-d, 1j♂, 09/05/2012, Castro Verde (Herdade dos Pereiros). Ref. **A999**-11-h, 1j, 09/05/2012, Castro Verde (Herdade dos Pereiros).

Paidiscura pallens **(Blackwall, 1834)**

Material examined: Ref. **122**-9b, 1♂, 12/04/2012, Castro Verde (Herdade da Chaiça Velha).

Phylloneta impressa **(L. Koch, 1881)**

Material examined: Ref. **127**-6c, 1j, 11/04/2012, Castro Verde (Herdade dos Montinhos). Nos hemos guiado de la facies pigmentaria dorsal del opistosoma como criterio para su identificación.

Ruborridion musivum **(Simon, 1873)**

Material examined: Ref. **128**-8f, 1♀, 04/04/2012 Castro Verde (Herdade de Carriça-Viseus).

***Simitidion simile* (C. L. Koch, 1836)**

Material examined: Ref. **121**-10a, 1j, 16/04/2012, Castro Verde (Monte do Tacanho). Se ha identificado como *S. simile* por su característico patrón pigmentario opistosómico.

***Steatoda albomaculata* (De Geer, 1778)**

Material examined: Refs. **19**-3a, 1j, 26/04/2012, Castro Verde (Herdade do Torrejão). Ref. **19**-9e, 1j, 26/04/2012, Castro Verde (Herdade do Torrejão). Ref. **127**-6c, 1j, 11/04/2012, Castro Verde (Herdade dos Montinhos). Ref. **A482**-3b, 1j, 09/05/2012, Castro Verde (Amendoeira Nova). Ref. **A505**-4a, 1j, 08/05/2012, Castro Verde (Herdade do Reguengo). **A505**-8c, 1j, 08/05/2012, Castro Verde (Herdade do Reguengo). Ref. **A527**-3g, 1j, 07/05/2012, Castro Verde (Herdade dos Bispos). Hemos asimilado a *S. albomaculata* estos 7 juveniles por su facies pigmentaria y por haber sido citada previamente de Castro Verde.

***Theridion pinastri* L. Koch, 1872**

Material examined: Ref. **6**-4-e, 1j, 24/04/2012, Castro Verde (Herdade de A de Neves da Marinha). Ref. **6**-8-h, 1♀, 24/04/2012, Castro Verde (Herdade de A de Neves da Marinha). Ref. **6**-10-e, 1♀, 24/04/2012, Castro Verde (Herdade de A de Neves da Marinha). Ref. **14**-7-e, 1j, 18/04/2012, Castro Verde (Lagoa da Mó). Ref. **15**-2-h, 1j, 12/04/2012, Castro Verde (Lagoa da Mó). Ref. **15**-4-e, 1j, 12/04/2012, Castro Verde (Lagoa da Mó). Ref. **21**-6-a, 1♀ 1j, 21/04/2012, Castro

Verde (Herdade da Barrigoa). Ref. **21**-11-c, 1j, 21/04/2012, Castro Verde (Herdade da Barrigoa). Ref. **25**-1-c, 1♀, 05/04/2012, Castro Verde (Herdade dos Touris e Rolão). Ref. **25**-11-a, 1j, 05/04/2012, Castro Verde (Herdade dos Touris e Rolão). Ref. **27**-5-d, 1j, 24/04/2012, Castro Verde (Herdade das Sesmarias). Ref. **27**-8-c, 1♀, 24/04/2012, Castro Verde (Herdade das Sesmarias). Ref. **27**-9-e, 1♀, 24/04/2012, Castro Verde (Herdade das Sesmarias). Ref. **27**-11-c, 1♀, 24/04/2012, Castro Verde (Herdade das Sesmarias). Ref. **36**-7-k1, 1j, 11/04/2012, Castro Verde (Herdade dos Bispos). Ref. **37**-7-d, 1j, 09/04/2012, Castro Verde (Herdade de São Marcos). Ref. **47**-9-i, 1j, 09/04/2012, Castro Verde (Herdade do Almarginho). Ref. **53**-11-e, 1j, 20/04/2012, Castro Verde (Herdade do Reguengo). Ref. **54**-8b-d, 1j, 25/04/2012, Castro Verde (Herdade do Roncanho). Ref. **55**-3-g, 1j, 21/04/2012, Castro Verde (Herdade da Barrigoa). Ref. **57**-9-b, 1♀, 06/04/2012, Castro Verde (Monte da Azinheira). Ref. **70**-2-c, 1j, 21/04/2012, Castro Verde (Monte dos Janeiros). Ref. **79**-2-e, 2jj, 07/04/2012, Castro Verde (Monte da Chada). Ref. **81**-8-h, 1j, 20/04/2012, Castro Verde (Herdade de Reidias). Ref. **86**-8-e, 1j, 10/05/2012, Castro Verde (Herdade das Mestras). Ref. **93**-10-a, 1♀, 17/04/2012, Aljustrel (Herdade da Sobreira). Ref. **97**-8-c, 1j, 13/04/2012, Castro Verde (Cumeada Nova). Ref. **108**-3-l, 1♂, 10/04/2012, Castro Verde (Herdade dos Merendeiros). Ref. **116**-3-c, 1j, 10/04/2012, Castro Verde (Herdade dos Merendeiros). Ref. **123**-2-c, 1j, 20/04/2012, Castro Verde (Herdade das bicadas). Ref. **126**-1-f, 1j, 05/04/2012, Castro Verde (Monte do Seixo). Ref. **126**-4-c, 1♀, 05/04/2012, Castro Verde (Monte do Seixo). Ref. **126**-8-f, 1♀, 05/04/2012, Castro Verde (Monte do Seixo). Ref. **126**-9-a, 1♀, 05/04/2012, Castro Verde (Monte do Seixo). Ref. **127**-4-a, 1j, 11/04/2012, Castro Verde (Herdade dos Montinhos). Ref. **128**-2-a, 1j, 04/04/2012, Castro Verde

(Herdade de Carriça-Viseus). Ref. **128**-7-d, 1j, 04/04/2012, Castro Verde (Herdade de Carriça-Viseus). Ref. **128**-12-a, 1♂, 04/04/2012, Castro Verde (Herdade de Carriça-Viseus). Ref. **132**-3-j, 1♀, 06/05/2012, Castro Verde (Monte das Cabeceiras). Ref. **A57**-7-f, 1j, 08/05/2012, Aljustrel (Herdade de Corta Rabos). Ref. **A166**-3-g, 1j, 07/05/2012, Castro Verde (Herdade dos Brunhachos). Ref. **A166**-5-b, 1♂, 07/05/2012, Castro Verde (Herdade dos Brunhachos). Ref. **A297**-10-f, 1♂, 09/05/2012, Castro Verde (Lagoa da Mó). Ref. **A299**-7-d, 1♀, 08/05/2012, Castro Verde (Lagoa da Mó). Ref. **A299**-9-b, 1♀, 08/05/2012, Castro Verde (Lagoa da Mó). Ref. **A505**-3-d, 1j, 08/05/2012, Castro Verde (Herdade do Reguengo).

Family THOMISIDAE Sundevall, 1833

Thomisidae sp.: (3339 jj. indet. *Xysticus/Ozyptila*)

***Bassaniodes bliteus* (Simon, 1875)**

Material examined: Ref. **49**-11h, 1♀, 25/04/2012, Castro Verde (Herdade da Navarra); Ref. **70**-5d, 1♀, 21/04/2012. Castro Verde (Monte dos Janeiros).

***Ozyptila pauxilla* (Simon, 1870)**

Material examined: Ref. **4**-2-c, 1♀, 15/04/2012, Castro Verde (Heredade das Mouras). Ref. **4**-5-j, 1♂, 15/04/2012, Castro Verde (Heredade das Mouras). Ref. **4**-6-c, 1♂, 15/04/2012, Castro Verde (Heredade das Mouras). Ref. **4**-10-e, 1♀, 15/04/2012, Castro Verde (Heredade das Mouras). Ref. **6**-3-c, 1♀, 24/04/2012, Castro Verde (Herdade de A de Neves da Marinha). Ref. **6**-11-b, 1♂ 1♀,

24/04/2012, Castro Verde (Herdade de A de Neves da Marinha). Ref. **14**-2-c, 1♀, 18/04/2012, Castro Verde (Lagoa da Mó). Ref. **15**-5-b, 1♂ 1♀, 12/04/2012, Castro Verde (Lagoa da Mó). Ref. **15**-6-a, 1♂, 12/04/2012, Castro Verde (Lagoa da Mó). Ref. **19**-6-a, 1♂, 26/04/2012, Castro Verde (Herdade do Torrejão). Ref. **21**-11-c, 1♂, 21/04/2012, Castro Verde (Herdade da Barrigoa). Ref. **23**-3-c, 1♂, 03/04/2012, Castro Verde (Monte do Vale das Gretas). Ref. **23**-12-b, 1♂, 03/04/2012, Castro Verde (Monte do Vale das Gretas). Ref. **25**-1-c, 1♀, 05/04/2012, Castro Verde (Herdade dos Touris e Rolão). Ref. **25**-2-d, 1♂, 05/04/2012, Castro Verde (Herdade dos Touris e Rolão). Ref. **25**-10-b, 1♂, 05/04/2012, Castro Verde (Herdade dos Touris e Rolão). Ref. **25**-12-e, 2♂♂, 05/04/2012, Castro Verde (Herdade dos Touris e Rolão). Ref. **27**-2-c, 1♂, 24/04/2012, Castro Verde (Herdade das Sesmarias). Ref. **27**-5-d, 1♂, 24/04/2012, Castro Verde (Herdade das Sesmarias). Ref. **27**-8-c, 1♂, 24/04/2012, Castro Verde (Herdade das Sesmarias). Ref. **34**-6-c, 1♀, 16/04/2012, Castro Verde (Herdade do Tacanho e Merendeiros). Ref. **34**-8-c, 1♂, 16/04/2012, Castro Verde (Herdade do Tacanho e Merendeiros). Ref. **34**-11-d, 1♂, 16/04/2012, Castro Verde (Herdade do Tacanho e Merendeiros). Ref. **35**-4-d, 1♀, 05/04/2012, Castro Verde (Monte do Seixo). Ref. **35**-7-d, 1♂, 05/04/2012, Castro Verde (Monte do Seixo). Ref. **37**-9-d, 1♀, 09/04/2012, Castro Verde (Herdade de São Marcos). Ref. **45**-2-c, 1♂, 13/04/2012, Castro Verde (Monte das Fontes Barbas Velho). Ref. **45**-4-f, 1♀, 13/04/2012, Castro Verde (Monte das Fontes Barbas Velho). Ref. **47**-12-c, 1♀, 09/04/2012, Castro Verde (Herdade do Almarginho). Ref. **49**-5-a, 1♀, 25/04/2012, Castro Verde (Herdade da Navarra). Ref. **50**-10-c, 1♂, 16/04/2012, Castro Verde (Herdade do Torrejão). Ref. **50**-11-f, 1♂, 16/04/2012, Castro Verde (Herdade do Torrejão). Ref. **51**-7-e, 2♂♂ 1♀,

13/04/2012, Castro Verde (Herdade do Torrejão). Ref. **53**-4-b, 2jj, 20/04/2012, Castro Verde (Herdade do Reguengo). Ref. **53**-9-b, 1♂, 20/04/2012, Castro Verde (Herdade do Reguengo). Ref. **54**-2-f, 1j, 25/04/2012, Castro Verde (Herdade do Roncanho). Ref. **54**-3-e, 2jj, 25/04/2012, Castro Verde (Herdade do Roncanho). Ref. **54**-6-f, 1j, 25/04/2012, Castro Verde (Herdade do Roncanho). Ref. **54**-9-b, 1♂ 5jj, 25/04/2012, Castro Verde (Herdade do Roncanho). Ref. **55**-10-b, 4jj, 21/04/2012, Castro Verde (Herdade da Barrigoa). Ref. **55**-11-a, 1♂, 21/04/2012, Castro Verde (Herdade da Barrigoa). Ref. **56**-2-h, 1♀, 18/04/2012, Castro Verde (Herdade da Barrigoa). Ref. **56**-4-b, 3jj, 18/04/2012, Castro Verde (Herdade da Barrigoa). Ref. **56**-6-f, 1♀ 1j, 18/04/2012, Castro Verde (Herdade da Barrigoa). Ref. **57**-5-e, 2jj, 06/04/2012, Castro Verde (Monte da Azinheira). Ref. **57**-7-e, 3jj, 06/04/2012, Castro Verde (Monte da Azinheira). Ref. **57**-10-g, 2jj, 06/04/2012, Castro Verde (Monte da Azinheira). Ref. **57**-11-i, 7jj, 06/04/2012, Castro Verde (Monte da Azinheira). Ref. **58**-4-b, 1♂, 07/04/2012, Mértola (Herdade da Benviúda). Ref. **58**-7-e, 1♂ 1♀, 07/04/2012, Mértola (Herdade da Benviúda). Ref. **58**-10-f, 1♀, 07/04/2012, Mértola (Herdade da Benviúda). Ref. **58**-12-d, 2jj, 07/04/2012, Mértola (Herdade da Benviúda). Ref. **61**-4-a, 3jj, 05/05/2012, Aljustrel (Monte da Torre). Ref. **61**-7-e, 1j, 05/05/2012, Aljustrel (Monte da Torre). Ref. **61**-8-b, 2jj, 05/05/2012, Aljustrel (Monte da Torre). Ref. **61**-9-f, 1♂ 1j, 05/05/2012, Aljustrel (Monte da Torre). Ref. **67**-3-c, 1♂, 12/04/2012, Aljustrel (Monte da Chaiça). Ref. **67**-4-a, 1j, 12/04/2012, Aljustrel (Monte da Chaiça). Ref. **70**-10-a, 1♂, 21/04/2012, Castro Verde (Monte dos Janeiros). Ref. **72**-5-a, 1♀ 1j, 15/04/2012, Castro Verde (Cumeada Nova). Ref. **72**-9-a, 1♂, 15/04/2012, Castro Verde (Cumeada Nova). Ref. **73**-6-c, 1♀, 19/04/2012, Castro Verde (Herdade dos Longos). Ref. **73**-10-h, 1♂, 19/04/2012, Castro Verde

(Herdade dos Longos). Ref. **79**-10-i, 1♂, 07/04/2012, Castro Verde (Monte da Chada). Ref. **79**-12-e, 1♂, 07/04/2012, Castro Verde (Monte da Chada). Ref. **81**-9-e, 2jj, 20/04/2012, Castro Verde (Herdade de Reidias). Ref. **83**-4-i, 2♀♀, 06/05/2012, Castro Verde (Herdade do Álamo). Ref. **83**-6-d, 1♀, 06/05/2012, Castro Verde (Herdade do Álamo). Ref. **83**-7-h, 3jj, 06/05/2012, Castro Verde (Herdade do Álamo). Ref. **83**-8-f, 1♀, 06/05/2012, Castro Verde (Herdade do Álamo). Ref. **86**-2-a, 1♂, 10/05/2012, Castro Verde (Herdade das Mestras). Ref. **87**-2-c, 1♂, 06/05/2012, Castro Verde (Herdade das Mestras). Ref. **89**-2-c, 1♂, 06/05/2012, Castro Verde (Herdade do Carapetal). Ref. **92**-4-c, 1♂, 17/04/2012, Aljustrel (Monte do Carregueiro). Ref. **97**-5-c, 1♂, 13/04/2012, Castro Verde (Cumeada Nova). Ref. **97**-9-e, 1♂, 13/04/2012, Castro Verde (Cumeada Nova). Ref. **102**-7-f, 1♂, 15/04/2012, Castro Verde (Monte da Fonte). Ref. **104**-1-b, 1♂, 04/04/2012, Castro Verde (Herdade dos Touris e Rolão). Ref. **104**-7-h, 1♂, 04/04/2012, Castro Verde (Herdade dos Touris e Rolão). Ref. **104**-9-d, 1♂, 04/04/2012, Castro Verde (Herdade dos Touris e Rolão). Ref. **104**-10-e, 1j, 04/04/2012, Castro Verde (Herdade dos Touris e Rolão). Ref. **104**-11-c, 1♂, 04/04/2012, Castro Verde (Herdade dos Touris e Rolão). Ref. **107**-5-e, 1♀, 18/04/2012, Castro Verde (Herdade das Mestras). Ref. **107**-6-d, 1♀, 18/04/2012, Castro Verde (Herdade das Mestras). Ref. **107**-8-b, 1♂, 18/04/2012, Castro Verde (Herdade das Mestras). Ref. **107**-11-f, 1♀, 18/04/2012, Castro Verde (Herdade das Mestras). Ref. **108**-11-a, 1♂, 10/04/2012, Castro Verde (Herdade dos Merendeiros). Ref. **109**-5-c, 1♀, 24/04/2012, Castro Verde (Monte da Achada). Ref. **111**-2-d, 1♀, 16/04/2012, Castro Verde (Monte da Perdigoa). Ref. **111**-3-g, 1♀, 16/04/2012, Castro Verde (Monte da Perdigoa). Ref. **111**-11-e, 1♂, 16/04/2012, Castro Verde (Monte da Perdigoa). Ref. **112**-10-g, 1♂, 20/04/2012,

Castro Verde (Monte do Freire). Ref. **112**-11-d, 1♂, 20/04/2012, Castro Verde (Monte do Freire). Ref. **113**-3-c, 1♂ 1♀, 10/04/2012, Castro Verde (Courela do Monte Novo). Ref. **113**-4-j, 1♂, 10/04/2012, Castro Verde (Courela do Monte Novo). Ref. **113**-11-c, 1♀, 10/04/2012, Castro Verde (Courela do Monte Novo). Ref. **114**-10-a, 1♂, 08/04/2012, Castro Verde (Herdade das Cuchilhas). Ref. **122**-2-d, 1♂, 12/04/2012, Castro Verde (Herdade da Chaiça Velha). Ref. **122**-6-d, 2♂♂, 12/04/2012, Castro Verde (Herdade da Chaiça Velha). Ref. **122**-8-d, 3♂♂, 12/04/2012, Castro Verde (Herdade da Chaiça Velha). Ref. **122**-10-e, 1♀, 12/04/2012, Castro Verde (Herdade da Chaiça Velha). Ref. **122**-11-a, 1♂, 12/04/2012, Castro Verde (Herdade da Chaiça Velha). Ref. **123**-7-d, 1♂, 20/04/2012, Castro Verde (Herdade das bicadas). Ref. **124**-3-a, 1♂, 18/04/2012, Castro Verde (Herdade das Mestras). Ref. **126**-5-b, 1j, 05/04/2012, Castro Verde (Monte do Seixo). Ref. **128**-8-f, 1♀, 04/04/2012, Castro Verde (Herdade de Carriça-Viseus). Ref. **129**-5-d, 1♀, 08/05/2012, Castro Verde (Herdade da Zibreira). Ref. **129**-6-d, 1♀, 08/05/2012, Castro Verde (Herdade da Zibreira). Ref. **129**-7-g, 1♀, 08/05/2012, Castro Verde (Herdade da Zibreira). Ref. **131**-2-f, 1j, 04/05/2012, Castro Verde (Herdade do Torrejão). Ref. **131**-8-a, 1♀, 04/05/2012, Castro Verde (Herdade do Torrejão). Ref. **132**-2-k, 1♂, 06/05/2012, Castro Verde (Monte das Cabeceiras). Ref. **132**-8-a, 1♀, 06/05/2012, Castro Verde (Monte das Cabeceiras). Ref. **A166**-5-b, 1♂, 07/05/2012, Castro Verde (Herdade dos Brunhachos). Ref. **A349**-4-a, 1♂, 10/05/2012, Castro Verde (Herdade das Mestras). Ref. **A388**-8-a, 1♂, 10/05/2012, Castro Verde (Monte da Achada). Ref. **A482**-9-e, 2♂♂, 09/05/2012, Castro Verde (Amendoeira Nova). Ref. **A999**-3-b, 1♀, 09/05/2012, Castro Verde (Herdade dos Pereiros).

***Runcinia grammica* (C. L. Koch, 1837)**

Material examined: Aunque se trata de individuos inmaduros, los hemos asimilado a *R. grammica* por su facies pigmentaria y por estar haber sido citada previamente de la provincia de Beja. Ref. **6**-8-h, 1j, 24/04/2012, Castro Verde (Herdade de A de Neves da Marinha). Ref. **14**-10-j, 1j, 18/04/2012, Castro Verde (Lagoa da Mó). Ref. **15**-5-b, 1j, 12/04/2012, Castro Verde (Lagoa da Mó). Ref. **15**-11-c, 1j, 12/04/2012, Castro Verde (Lagoa da Mó). Ref. **23**-10-k, 1j, 03/04/2012, Castro Verde (Monte do Vale das Gretas). Ref. **27**-10-d, 1j, 24/04/2012, Castro Verde (Herdade das Sesmarias). Ref. **35**-1-d, 1j, 05/04/2012, Castro Verde (Monte do Seixo). Ref. **36**-6-h, 1j, 11/04/2012, Castro Verde (Herdade dos Bispos). Ref. **36**-10-g, 1j, 11/04/2012, Castro Verde (Herdade dos Bispos). Ref. **36**-11-d, 1j, 11/04/2012, Castro Verde (Herdade dos Bispos). Ref. **37**-2-e, 1j, 09/04/2012, Castro Verde (Herdade de São Marcos). Ref. **45**-9-a, 1j, 13/04/2012, Castro Verde (Monte das Fontes Barbas Velho). Ref. **47**-1-a, 1j, 09/04/2012, Castro Verde (Herdade do Almarginho). Ref. **47**-6-c, 2jj, 09/04/2012, Castro Verde (Herdade do Almarginho). Ref. **49**-2-d, 1j, 25/04/2012, Castro Verde (Herdade da Navarra). Ref. **49**-5-a, 3jj, 25/04/2012, Castro Verde (Herdade da Navarra). Ref. **49**-6-a, 1j, 25/04/2012, Castro Verde (Herdade da Navarra). Ref. **49**-8-d, 1j, 25/04/2012, Castro Verde (Herdade da Navarra). Ref. **49**-9-a, 1j, 25/04/2012, Castro Verde (Herdade da Navarra). Ref. **55**-11-a, 2jj, 21/04/2012, Castro Verde (Herdade da Barrigoa). Ref. **56**-10-b, 1j, 18/04/2012, Castro Verde (Herdade da Barrigoa). Ref. **57**-7-e, 1j, 06/04/2012, Castro Verde (Monte da Azinheira). Ref. **67**-5-j, 1j, 12/04/2012, Aljustrel (Monte da Chaiça). Ref. **70**-7-a, 1j, 21/04/2012, Castro Verde (Monte dos Janeiros). Ref. **83**-4-i, 1j, 06/05/2012, Castro Verde (Herdade do Álamo). Ref. **83**-7-h, 1j, 06/05/2012,

Castro Verde (Herdade do Álamo). Ref. **88**-11-j, 1j, 25/04/2012, Castro Verde (Herdade da Portela). Ref. **104**-3-b, 1j, 04/04/2012, Castro Verde (Herdade dos Touris e Rolão). Ref. **104**-7-h, 1j, 04/04/2012, Castro Verde (Herdade dos Touris e Rolão). Ref. **104**-10-e, 2jj, 04/04/2012, Castro Verde (Herdade dos Touris e Rolão). Ref. **107**-3-d, 1j, 18/04/2012, Castro Verde (Herdade das Mestras). Ref. **107**-9-a, 1j, 18/04/2012, Castro Verde (Herdade das Mestras). Ref. **107**-10-e, 1j, 18/04/2012, Castro Verde (Herdade das Mestras). Ref. **108**-3-l, 1j, 10/04/2012, Castro Verde (Herdade dos Merendeiros). Ref. **108**-9-d, 1j, 10/04/2012, Castro Verde (Herdade dos Merendeiros). Ref. **109**-2-e, 1j, 24/04/2012, Castro Verde (Monte da Achada). Ref. **109**-7-h, 3jj, 24/04/2012, Castro Verde (Monte da Achada). Ref. **109**-9-e, 2jj, 24/04/2012, Castro Verde (Monte da Achada). Ref. **109**-10-g, 2jj, 24/04/2012, Castro Verde (Monte da Achada). Ref. **111**-10-c, 1j, 16/04/2012, Castro Verde (Monte da Perdigoa). Ref. **114**-12-d, 1j, 08/04/2012, Castro Verde (Herdade das Cuchilhas). Ref. **121**-7-e, 1j, 16/04/2012, Castro Verde (Monte do Tacanho). Ref. **121**-10-d, 1j, 16/04/2012, Castro Verde (Monte do Tacanho). Ref. **122**-11-a, 1j, 12/04/2012, Castro Verde (Herdade da Chaiça Velha). Ref. **123**-5-g, 1j, 20/04/2012, Castro Verde (Herdade das bicadas). Ref. **123**-9-b, 1j, 20/04/2012, Castro Verde (Herdade das bicadas). Ref. **124**-10-b, 1j, 18/04/2012, Castro Verde (Herdade das Mestras). Ref. **124**-11-f, 1j, 18/04/2012, Castro Verde (Herdade das Mestras). Ref. **125**-7-f, 1j, 17/04/2012, Aljustrel (Herdade da Sobreira). Ref. **126**-3-c, 1j, 05/04/2012, Castro Verde (Monte do Seixo). Ref. **126**-7-b, 1j, 05/04/2012, Castro Verde (Monte do Seixo). Ref. **128**-2-a, 1j, 04/04/2012, Castro Verde (Herdade de Carriça-Viseus). Ref. **128**-7-d, 2jj, 04/04/2012, Castro Verde (Herdade de Carriça-Viseus). Ref. **132**-11-b, 2jj, 06/05/2012, Castro Verde (Monte das Cabeceiras). Ref. **A297**-3-b, 1j,

09/05/2012, Castro Verde (Lagoa da Mó). Ref. **A297**-4-a, 1j, 09/05/2012, Castro Verde (Lagoa da Mó). Ref. **A297**-6-b, 1j, 09/05/2012, Castro Verde (Lagoa da Mó). Ref. **A297**-7-a, 1j, 09/05/2012, Castro Verde (Lagoa da Mó). Ref. **A297**-9-c, 1j, 09/05/2012, Castro Verde (Lagoa da Mó). Ref. **A297**-10-f, 2jj, 09/05/2012, Castro Verde (Lagoa da Mó). Ref. **A297**-11-f, 2jj, 09/05/2012, Castro Verde (Lagoa da Mó). Ref. **A299**-2-c, 1j, 08/05/2012, Castro Verde (Lagoa da Mó). Ref. **A299**-3-i, 1j, 08/05/2012, Castro Verde (Lagoa da Mó). Ref. **A299**-8-j, 1j, 08/05/2012, Castro Verde (Lagoa da Mó). Ref. **A299**-11R-b, 1j, 08/05/2012, Castro Verde (Lagoa da Mó). Ref. **A388**-9-c, 1j, 10/05/2012, Castro Verde (Monte da Achada). Ref. **A423**-7-d, 1j, 08/05/2012, Castro Verde (Herdade da Zibreira). Ref. **A999**-3-b, 1j, 09/05/2012, Castro Verde (Herdade dos Pereiros).

***Thomisus onustus* Walckenaer, 1805**

Material examined: Ref. **35**-7d, 1j, 05/04/2012, Castro Verde (Monte do Seixo). Ref. **104**-11c, 1j, 04/04/2012. Castro Verde (Herdade dos Touris e Rolão). Ref. **108**-6e, 1j, 10/04/2012, Castro Verde (Herdade dos Merendeiros). Refs. **110**-6a, 1♂, 04/05/2012, Castro Verde (Monte da Perdigoa). Ref. **110**-11b, 1♂, 04/05/2012, Castro Verde (Monte da Perdigoa). Ref. **111**-6d, 1j, 16/04/2012, Castro Verde (Monte da Perdigoa). Ref. **114**-12d, 1♂, 08/04/2012, Castro Verde (Herdade das Cuchilhas). Ref. **121**-11a, 1j, 16/04/2012, Castro Verde (Monte do Tacanho). Ref. **A-505**-4a, 1♀, 08/05/2012, Castro Verde (Herdade do Reguengo).

***Xysticus grallator* Simon, 1932**

Material examined: Ref. **124**-5a, 1♀, 18/04/2012, Castro Verde (Herdade das Mestras). Ref. **127**-10a, 1♀, 11/04/2012, Castro Verde (Herdade dos Montinhos).

Xysticus nubilus **Simon, 1875**

Material examined: Ref. **27**-8c, 1♀, 24/04/2012, Castro Verde (Herdade das Sesmarias). Ref. **49**-10b, 1♀, 25/04/2012, Castro Verde (Herdade da Navarra). Ref. **70**-2c, 1♀, 21/04/2012, Castro Verde (Monte dos Janeiros). Ref. **83**-10f, 1♀, 06/05/2012, Castro Verde (Herdade do Álamo). Ref. **109**-7h, 1♀, 1j, 24/04/2012, Castro Verde (Monte da Achada). Ref. **123**-2c, 1♀, 20/04/2012, Castro Verde (Herdade das bicadas). Ref. **132**-8a, 1♀, 06/05/2012, Castro Verde (Monte das Cabeceiras). Ref. **A423**-9a, 1♀, 08/05/2012, Castro Verde (Herdade da Zibreira). Ref. **A527**-7c, 1♀, 07/05/2012, Castro Verde (Herdade dos Bispos).

Family ZODARIIDAE Thorell, 1881

Zodarion jozefienae **Bosmans, 1994**

Material examined: Ref. **4**-6-c, 1♂, 15/04/2012, Castro Verde (Heredade das Mouras). Ref. **37**-3-a, 1♂, 09/04/2012, Castro Verde (Herdade de São Marcos). Ref. **43**-10-h, 1♂, 21/04/2012, Castro Verde (Monte da Comenda). Ref. **54**-11-c, 1♂, 25/04/2012, Castro Verde (Herdade do Roncanho). Ref. **67**-2-e, 1♂, 12/04/2012, Aljustrel (Monte da Chaiça). Ref. **67**-7-f, 1♂, 12/04/2012, Aljustrel (Monte da Chaiça). Ref. **67**-9-c, 1♂, 12/04/2012, Aljustrel (Monte da Chaiça). Ref. **70**-2-c, 1♂, 21/04/2012, Castro Verde (Monte dos Janeiros). Ref. **70**-7-a, 1♂, 21/04/2012, Castro Verde (Monte dos Janeiros). Ref. **78**-3-f, 1♂, 09/04/2012, Castro Verde (Herdade de São Marcos). Ref. **79**-12-e, 1♂, 07/04/2012, Castro Verde (Monte da Chada). Ref. **83**-2-g, 1♂, 06/05/2012, Castro Verde (Herdade do Álamo). Ref. **87**-7-g, 1♂, 06/05/2012, Castro Verde (Herdade das Mestras).

Ref. **92**-10-b, 1j, 17/04/2012, Aljustrel (Monte do Carregueiro). Ref. **96**-5-b, 1♂, 11/04/2012, Castro Verde (Herdade dos Bispos). Ref. **107**-6-d, 1♂, 18/04/2012, Castro Verde (Herdade das Mestras). Ref. **116**-2-c, 1♂, 10/04/2012, Castro Verde (Herdade dos Merendeiros). Ref. **124**-8-d, 1♂, 18/04/2012, Castro Verde (Herdade das Mestras). Ref. **124**-9-g, 1♂, 18/04/2012, Castro Verde (Herdade das Mestras). Ref. **125**-11-f, 1♂, 17/04/2012, Aljustrel (Herdade da Sobreira). Ref. **127**-10-a, 1♂, 11/04/2012, Castro Verde (Herdade dos Montinhos). Ref. **131**-4-c, 1♂, 04/05/2012, Castro Verde (Herdade do Torrejão). Ref. **A46**-8-c, 1♂, 10/05/2012, Aljustrel (Monte da Chaiça). Ref. **A57**-6-c, 1♂, 08/05/2012, Aljustrel (Herdade de Corta Rabos). Ref. **A388**-2-b, 1♂, 10/05/2012, Castro Verde (Monte da Achada). Ref. **A423**-3-i, 1♂, 08/05/2012, Castro Verde (Herdade da Zibreira). Ref. **A423**-10-d, 1♂, 08/05/2012, Castro Verde (Herdade da Zibreira).

Order OPILIONES

Family PHALANGIIDAE Latreille, 1802

Dasylobus ibericus **(Rambla, 1967)** (2♂♂)

Material examined: Ref. **79**-1-a, 1♂, 07/04/2012, Castro Verde (Monte da Chada). Ref. **133**-9-a, 1♂, 09/04/2012, Castro Verde (Monte da Albergaria).

Family SCLEROSOMATHIDAE Simon, 1879

Homalenotus buchneri **(Schenkel, 1936)**; (14♂♂, 6♀♀, 57 jj.)

Material examined: Ref. **11**-11-a, 1♂, 15/04/2012, Castro Verde (Monte do Broco e Capitão). Ref. **23**-4-l, 1j, 03/04/2012, Castro Verde (Monte do Vale das Gretas). Ref. **23**-7-a, 1j, 03/04/2012, Castro Verde (Monte do Vale das Gretas). Ref. **23**-11-h, 1j, 03/04/2012, Castro Verde (Monte do Vale das Gretas). Ref. **36**-6-b, 1j, 11/04/2012, Castro Verde (Herdade dos Bispos). Ref. **36**-10-h, 1j, 11/04/2012, Castro Verde (Herdade dos Bispos). Ref. **43**-3-a, 1j, 21/04/2012, Castro Verde (Monte da Comenda). Ref. **43**-5-l, 1j, 21/04/2012, Castro Verde (Monte da Comenda). Ref. **45**-7-g, 1j, 13/04/2012, Castro Verde (Monte das Fontes Barbas Velho). Ref. **50**-3-b, 1♀ 2jj, 16/04/2012, Castro Verde (Herdade do Torrejão). Ref. **53**-4-l, 1j, 20/04/2012, Castro Verde (Herdade do Reguengo). Ref. **53**-5-j, 1j, 20/04/2012, Castro Verde (Herdade do Reguengo). Ref. **53**-9-c, 1j, 20/04/2012, Castro Verde (Herdade do Reguengo). Ref. **55**-6-n, 1j, 21/04/2012, Castro Verde (Herdade da Barrigoa). Ref. **55**-7-h, 1j, 21/04/2012, Castro Verde (Herdade da Barrigoa). Ref. **55**-10-a, 2jj, 21/04/2012, Castro Verde (Herdade da Barrigoa). Ref. **55**-11-m, 1j, 21/04/2012, Castro Verde (Herdade da Barrigoa). Ref. **56**-9-e, 1♂, 18/04/2012, Castro Verde (Herdade da Barrigoa). Ref. **57**-6-c, 1♀, 06/04/2012, Castro Verde (Monte da Azinheira). Ref. **57**-12-f, 1j, 06/04/2012, Castro Verde (Monte da Azinheira). Ref. **70**-5-i, 1j, 21/04/2012, Castro Verde (Monte dos Janeiros). Ref. **70**-9-e, 1♂, 21/04/2012, Castro Verde (Monte dos Janeiros). Ref. **70**-11-l, 1j, 21/04/2012, Castro Verde (Monte dos Janeiros). Ref. **72**-7-a, 2jj, 15/04/2012, Castro Verde (Cumeada Nova). Ref. **73**-10-g, 1j, 19/04/2012, Castro Verde (Herdade dos Longos). Ref. **87**-2-k, 1j, 06/05/2012, Castro Verde (Herdade das Mestras). Ref. **87**-3-a, 2♂♂, 06/05/2012, Castro Verde (Herdade das Mestras). Ref. **87**-7-b, 2jj, 06/05/2012, Castro Verde (Herdade das Mestras). Ref. **89**-6-h, 1j, 06/05/2012, Castro Verde (Herdade do

Carapetal). Ref. **89**-10-h, 1j, 06/05/2012, Castro Verde (Herdade do Carapetal). Ref. **96**-8-e, 1♀ 1j, 11/04/2012, Castro Verde (Herdade dos Bispos). Ref. **96**-10-b, 1j, 11/04/2012, Castro Verde (Herdade dos Bispos). Ref. **97**-7-d, 1j, 13/04/2012, Castro Verde (Cumeada Nova). Ref. **97**-10-l, 1j, 13/04/2012, Castro Verde (Cumeada Nova). Ref. **108**-7-b, 2jj, 10/04/2012, Castro Verde (Herdade dos Merendeiros). Ref. **108**-9-h, 1j, 10/04/2012, Castro Verde (Herdade dos Merendeiros). Ref. **112**-3-a, 1♂ 2jj, 20/04/2012, Castro Verde (Monte do Freire). Ref. **112**-5-**c**, 1j, 20/04/2012, Castro Verde (Monte do Freire). Ref. **112**-7-d, 1j, 20/04/2012, Castro Verde (Monte do Freire). Ref. **112**-8-d, 1j, 20/04/2012, Castro Verde (Monte do Freire). Ref. **112**-9-d, 1j, 20/04/2012, Castro Verde (Monte do Freire). Ref. **123**-11-c, 1j, 20/04/2012, Castro Verde (Herdade das bicadas). Ref. **124**-4-d, 1j, 18/04/2012, Castro Verde (Herdade das Mestras). Ref. **125**-3-a, 2♂♂, 17/04/2012, Aljustrel (Herdade da Sobreira). Ref. **125**-7-e, 1j, 17/04/2012, Aljustrel (Herdade da Sobreira). Ref. **127**-3-g, 1j, 11/04/2012, Castro Verde (Herdade dos Montinhos). Ref. **127**-4-c, 2jj, 11/04/2012, Castro Verde (Herdade dos Montinhos). Ref. **127**-11-f, 2jj, 11/04/2012, Castro Verde (Herdade dos Montinhos). Ref. **128**-2-b, 1j, 04/04/2012, Castro Verde (Herdade de Carriça-Viseus). Ref. **128**-3-a, 1j, 04/04/2012, Castro Verde (Herdade de Carriça-Viseus). Ref. **129**-2-i, 1♂, 08/05/2012, Castro Verde (Herdade da Zibreira). Ref. **129**-9-a, 1♀, 08/05/2012, Castro Verde (Herdade da Zibreira). Ref. **132**-5-k, 1♂, 06/05/2012, Castro Verde (Monte das Cabeceiras). Ref. **A46**-3-g, 1j, 10/05/2012, Aljustrel (Monte da Chaiça). Ref. **A423**-2-i, 1♂ 1♀ 1j, 08/05/2012, Castro Verde (Herdade da Zibreira). Ref. **A423**-4-d, 1♀, 08/05/2012, Castro Verde (Herdade da Zibreira). Ref. **A505**-5-c, 2♂♂, 08/05/2012, Castro Verde (Herdade do Reguengo). Ref. **A505**-7-g, 1j, 08/05/2012, Castro Verde (Herdade do

Reguengo). Ref. **A505**-9-g, 1j, 08/05/2012, Castro Verde (Herdade do Reguengo). Ref. **A505**-10-f, 1♂, 08/05/2012, Castro Verde (Herdade do Reguengo). Ref. **A527**-7-g, 1j, 07/05/2012, Castro Verde (Herdade dos Bispos). Ref. **A527**-7-i, 1j, 07/05/2012, Castro Verde (Herdade dos Bispos).

Leiobunum sp. (2 jj. indet.)

<u>Material examined</u>: Ref. **43**-6-c, 2jj, 21/04/2012, Castro Verde (Monte da Comenda).
